# Elimination of detrimental grain boundary segregation in garnets

**DOI:** 10.1038/s41467-026-74887-z

**Published:** 2026-06-29

**Authors:** Kai Yao, Kwangnam Kim, Dylan Jennings, Jan Dippell, Lei Jin, Meng Ma, Xingyu Liu, Qianli Ma, Walter Sebastian Scheld, Christoph Roitzheim, Yuan Zeng, Timo Danner, Olivier Guillon, Mark Huijben, Johan E. ten Elshof, Liwen F. Wan, Arnulf Latz, Brandon C. Wood, Martin Finsterbusch, Dina Fattakhova-Rohlfing

**Affiliations:** 1 Forschungszentrum Jülich GmbH, Institute of Energy Materials and Devices: Materials Synthesis and Processing (IMD-2), Jülich, Germany; 2 University of Duisburg-Essen, Faculty of Engineering and Center for Nanointegration Duisburg-Essen CENIDE, Duisburg, Germany; 3 Laboratory for Energy Applications for the Future, Lawrence Livermore National Laboratory, Livermore, CA USA; 4 Forschungszentrum Jülich GmbH, Ernst-Ruska Centre for Microscopy and Spectroscopy with Electrons (ER-C), Jülich, Germany; 5 Faculty of Physics and Astronomy, Advanced Transmission Electron Microscopy, Ruhr University Bochum, Bochum, Germany; 6 German Aerospace Center, Institute of Engineering Thermodynamics, Stuttgart, Germany; 7 Helmholtz Institute Ulm for Electrochemical Energy Storage, Ulm, Germany; 8 State Key Laboratory of Flexible Electronics (LOFE), Institute of Flexible Electronics, Northwestern Polytechnical University, Xi’an, PR China; 9 Forschungszentrum Jülich GmbH, Institute of Energy Materials and Devices: Structure and Function of Materials (IMD-1), Jülich, Germany; 10 Helmholtz Institute Münster: Ionics in Energy Storage (IMD-4), Forschungszentrum Jülich GmbH, Münster, Germany; 11 Jülich-Aachen Research Alliance: JARA-Energy, Jülich, Germany; 12 MESA+ Institute for Nanotechnology, University of Twente, Enschede, AE The Netherlands; 13 Ulm University, Institute of Electrochemistry, Ulm, Germany

**Keywords:** Batteries, Molecular dynamics, Batteries

## Abstract

Garnet Li_7_La_3_Zr_2_O_12_ electrolyte is considered a key enabler of solid-state batteries with Li metal electrodes, but the grain boundaries impair its performance. To date, the understanding of grain boundary structures and its impact on performance remains elusive. Here, we show that element segregation at Li_7_La_3_Zr_2_O_12_ grain boundaries critically governs Li transport and nucleation. During conventional sintering, Al, Ta, and La segregate at grain boundaries, locally depleting Li and creating space-charge layers that lower total ionic conductivity. Simultaneously, this segregation leads to higher electronic conductivity along grain boundaries, which promotes Li nucleation at grain boundary edges with increased risk of dendrite formation. The underlying mechanism of segregation is governed by both thermodynamic driving forces and diffusion kinetics. Building on this understanding, we develop a strategy to achieve segregation-free grain boundaries through a rapid sintering protocol that utilizes the onset of solid-state softening. This approach yields transparent, polycrystalline Li_7_La_3_Zr_2_O_12_ with negligible grain boundary impedance and enhanced dendrite tolerance. By elucidating the structural origins and electrochemical consequences of grain boundary segregation, this work provides a guidance for the rational optimization of solid electrolytes.

## Abstract (web summary)

Understanding the origin of grain boundary (GB) structures and their impact remains a challenge. Here, the authors unravel the origin of LLZO GB segregation and create segregation-free GBs by a fast-sintering strategy to mitigate its detrimental effects on Li conductivity and dendrite formation.

## Introduction

Owing to its chemical and electrochemical stability, sufficiently high ionic conductivity, and non-flammability, the garnet-type electrolyte Li_7_La_3_Zr_2_O_12_ (LLZO) has emerged as an important electrolyte for reliable and safe solid-state batteries (SSBs) employing a Li metal electrode^1–5^. Since LLZO is a ceramic material, high-temperature sintering of LLZO-based components is required to fuse the particles and form mechanically robust and dense structures^6,7^. While many relevant processes already exist on an industrial scale^8^, the formation mechanisms and resulting structures of grain boundaries (GBs) in polycrystalline LLZO have a profound impact on cell performance but remain poorly understood^9,10^. The GB resistance defines the upper limit of ionic conductivity for a given composition^11,12^, whereas GB structural characteristics are closely correlated with Li dendrite initiation^13^. In addition, GBs deteriorate interfacial stability with positive electrode active materials by facilitating interdiffusion during sintering^14^.

Given the crucial role of GBs in determining the performance of LLZO-based components, governing conductivity and Li nucleation, considerable efforts have been devoted to unraveling structure-property correlations that dictate their electrochemical behavior^10,15,16^. Theoretical and experimental studies reveal that GBs with disordered structure increase the energy barrier for Li-ion transport^12,17^, thereby lowering the total ionic conductivity. Similarly, GBs exhibit a narrower band gap and thus higher electronic conductivity than the bulk phase^13,18^, which is believed to promote the formation of Li nuclei along the GBs in LLZO and their subsequent interconnection into dendritic filaments. In situ observations further reveal preferential Li nucleation along GBs on the LLZO surface^19,20^, which compromise the uniformity of Li plating and stripping at the interface to negative electrode, and thus accelerate Li dendrite propagation from negative electrode side^21^.

Structural disorder at GBs is accompanied by compositional variations with steep concentration gradients, typically referred to as elemental segregation^22^. Such segregation phenomena are common in ceramic materials and have been well understood for a select few systems, where they dramatically alter the local structure and electrostatic environment of GBs^23,24^. In particular, in LLZO, segregation also strongly influences conductivity and Li nucleation behavior. For example, the GB segregation of Ta and Al exceeding their solute limit deteriorates ionic conductivity through the formation of secondary oxide phases^25,26^. Conversely, certain secondary phases, such as ZrO_2_, can be leveraged to tune the local electronic structure, making GBs less prone to electron trapping and thereby suppressing Li nucleation^27^. The local chemical segregation has also been identified as a key factor contributing to dendrite propagation, although the precise mechanisms governing Li growth initiation under the influence of segregation remain elusive^28–30^.

Despite advances in understanding GBs in LLZO, they remain a long-standing scientific puzzle due to experimental challenges of resolving intrinsic chemistry within nanometer-thick layers and the theoretical difficulties of modeling GBs across multiple length scales in an unbiased yet accurate manner^13,18^. In particular, a fundamental understanding of segregation effects and their impact on ionic conductivity and Li nucleation is still lacking. The absence of such mechanistic insight hampers the rational design of sintering protocols and the optimization of separator performance. So far, conventional sintering requires prolonged processing times to compact LLZO powders, which increases relative density but degrades GB properties due to increased Li loss and uncontrollable elemental segregation^31,32^. Although pressure-assisted and field-assisted sintering techniques can effectively increase relative density, optimizing GB properties remains challenging due to limited understanding of sintering dynamics at the atomic level^12,33^. Furthermore, ensuring chemical homogeneity at the microscale is particularly difficult, as this calls for a predictive comprehension of the mechanisms governing GB segregation.

To address the above mechanistic shortcomings and facilitate the fabrication of advanced LLZO separators, we employ multiscale computational methodology integrating density functional theory (DFT), large-scale molecular dynamics (MD) simulations with accurate machine-learning force fields (MLFFs), and continuum-level modeling for space-charge layers (SCLs) to investigate segregation behavior and predict its potential impacts on intrinsic LLZO functionalities. The results show that the segregation is a result of the thermodynamic and kinetic factors and that innovations in sintering technology, especially those enabling rapid densification, are crucial under kinetically dominated conditions. Based on in-depth understanding of the segregation mechanism, we developed a fast-sintering strategy to fabricate a nearly fully dense, segregation-suppressed LLZO by understanding sintering dynamics through real-time tracking of sample displacement and gas evolution. The resulting LLZO pellets exhibit negligible GB resistance and significantly improved tolerance to dendrite formation. Combined theoretical and experimental investigations of the structural characteristics of LLZO GBs offer opportunities to elucidate how segregation governs GB ionic conductivity and to uncover the origin of Li nucleation mediated by segregation chemistry by resolving GB structures across atomic to nanoscale dimensions.

## Results and discussions

### Element segregation in LLZO

Doping with various elements such as Al, Ta, Ga, Nb, Hf, and Mo has been extensively used as an effective means to stabilize the cubic phase and improve the performance of LLZO in different parts of the cell^4,5^. Among the commonly used dopants, only Ta- and Ga-doped LLZO have achieved ionic conductivity above 1 mS cm^−1^. However, Ga-doped LLZO is known to be unstable in contact with Li metal and thermodynamically unstable during co-sintering with positive electrode active materials (CAMs)^34,35^. In contrast to Ga, Ta-doped LLZO exhibits a better reduction stability towards Li metal as well as good thermodynamic stability with various CAMs, including LiCoO_2_ and NCM, making it suitable as an anolyte, catholyte, and separator material in various cell designs. Based on these considerations, this work focuses primarily on Ta-doped LLZO (Li_6.45_Al_0.05_La_3_Zr_1.6_Ta_0.4_O_12_), with a small amount of Al additionally introduced to promote densification during sintering^36^, which was shown to be the most promising composition for the use in practical all-solid-state oxide batteries.

**Fig. 1:**
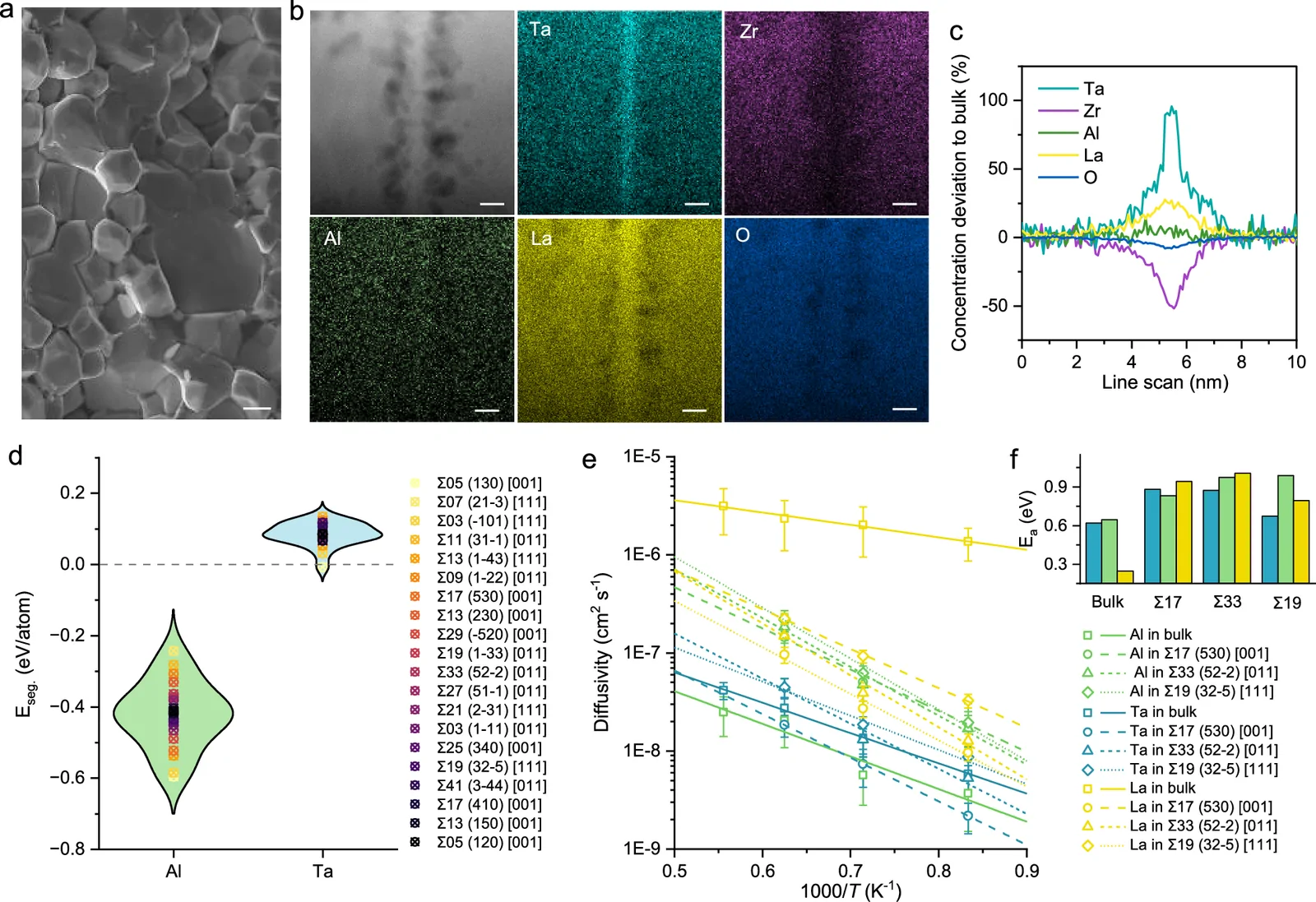
Element segregation in LLZO. **a** Representative SEM image of a cross-section of S-LLZO. The scale bars are 5 µm. **b**, **c** Representative HAADF-STEM image and STEM-EDS mappings (**b**), and line scan analysis (**c**) of the GB of S-LLZO. The scale bars are 10 nm. **d** Statistical results of MLFF-MD simulation on segregation energies of Al and Ta at 20 GBs. Each segregation energy was calculated across 50 randomly doped structures using the formula: *E*_seg_ = (*E*_doped gb_–*E*_doped bulk_)/*N*_dopant_. **e**, **f** Arrhenius plots of Ta, Al, and La by MLFF-MD over a temperature range of 1200–1800 K in bulk, GB Σ17 (530) [001], Σ33 (52-2) [011] and Σ19 (32-5) [111] (**e**) (Each data point with error bar is presented as the mean value of 10 independent simulations +/− SD), and activation energies comparison (**f**).

Conventional sintering of LLZO requires 10 h to achieve a relative density of about 94%, which is the upper limit in this study. Prolonged sintering yields diminishing returns in density and inevitably leads to excessive Li loss, which impairs ionic conductivity and phase stability (Supplementary Fig. 1a)^37^. A scanning electron microscopy (SEM) image of a mechanically fractured cross-section of LLZO produced by conventional sintering for 10 h shows a polycrystalline microstructure with micrometer-sized grains fractured along the GBs (Fig. 1a). The inhomogeneous element distribution observed in the energy-dispersive X-ray spectroscopy (EDS) of the cross-section is primarily due to localized element segregation at the GBs during sintering (Supplementary Fig. 1b). High-angle annular dark field scanning transmission electron microscopy (HAADF-STEM) shows the microstructure of a representative GB with a distinct GB core and dark contrast spots along the edges (Fig. 1b). These dark regions are assumed to be Li compounds (see discussion in a later section). STEM-EDS mapping qualitatively confirms significant Ta segregation and moderate to slight La and Al segregation at the GB. In contrast, Zr is strongly depleted, and O shows slight depletion (Fig. 1b). This sample is referred to as segregated LLZO (S-LLZO) in the following. Since Ta and Al substitute Zr and Li sites, respectively, their segregation reduces the concentrations of Zr and Li at the GBs. Li depletion may be particularly pronounced due to charge compensation mechanisms, given the higher oxidation states of the dopants. The detailed concentration variations of each element across the GB were further semi-quantified by line-scan analysis (Fig. 1c). Segregation and depletion are strongest at the GB core, reaching up to ~50% variation, and gradually decay on both sides over ~2.5 nm, forming a concentration gradient. Such compositional inhomogeneity could significantly impair separator quality and the overall performance of SSBs, underscoring the need to understand its causes.

In general, the known elemental segregation processes, such as sintering-driven segregation^38^, non-equilibrium segregation during annealing^39^, and radiation-induced segregation^40^, are invariably governed by kinetic and/or thermodynamic determinants. Among these, minimization of the system’s free energy, as described by Gibbs’ segregation theory, is one of the most widely accepted thermodynamic mechanisms^41^. To assess this possibility in LLZO, the segregation energies of Al and Ta in LLZO GBs were evaluated by MLFF-driven atomistic simulations, defined as the difference in energies between systems with dopants at GB regions and in bulk regions. To avoid the sampling bias inherent to single-GB simulations, where unrepresentative atomic configurations can distort segregation tendencies, we modeled 20 large symmetric tilt GBs up to 30 k atoms distributed along three tilt axes of [001], [011], and [111] (Supplementary Fig. 2a and Supplementary Data 1). The segregation energies exhibit similar normal distributions across all GBs (Fig. 1d), suggesting that the thermodynamic preference for Al and Ta segregation into GBs is largely independent of the tilt axis and misorientation angle (16.3° to 70.5°; Σ≤ 41). This consistency underscores the validity and broader relevance of localized GB characterizations using techniques such as electron microscopy and scanning probe microscopy. Across all GBs, Al shows negative segregation energies with an average of −0.42 eV/atom (Fig. 1d), indicating its thermodynamic preference for residing in GBs over Li sites in cubic LLZO. In contrast, Ta demonstrates only marginal segregation preference, with the majority of GBs exhibiting slightly positive segregation energies averaging 0.08 eV/atom; only the Σ05 (130) [001] GB shows a negative value. Since GBs are generally disordered, while the bulk remains crystalline, we further compared the defect formation energies of Al and Ta in amorphous and cubic LLZO using DFT (Supplementary Table 1). In the amorphous model, Al exhibits a defect formation energy that is 0.95 eV lower than at the 24 d Li site in the cubic structure, indicating its preference for disordered environments. Ta, on the other hand, has a formation energy in the amorphous model that is 0.12 eV higher than at the cubic Zr site, suggesting a weak preference for crystalline order. These DFT results align with the MLFF predictions and confirm the energetic trends driving segregation. Furthermore, we have found that La exhibits an energetically unfavorable segregation tendency in disordered environments, as the formation energy of La vacancies at the 24c cation sites of cubic LLZO is 2.50 eV higher than in the amorphous phase (Supplementary Table 1). Therefore, it is not sufficient to interpret the segregation behaviors of Al, Ta, and La solely from a thermodynamic perspective, and additional kinetic contributions must be taken into account.

Kinetic factors can dominate segregation when the thermodynamic driving forces are weak or when highly mobile species are involved^39^. For example, our previous work has shown diffusion of Co ions along Li-ion channels and segregation at GBs in LLZO^14^. Given that Al occupies the same Li sites as Co in LLZO and has high diffusivity during solid-state synthesis, it is likely that it takes the same migration pathway during sintering^42^. Similarly, Ta and La could reach GBs via Li channels if they form defects at Li sites and can migrate along the Li sites. To validate this hypothesis, we first simulated the formation energies of Ta and La Frenkel defects at Li sites in cubic LLZO. Ta ions prefer the Li-96 h site with a formation energy of 2.72 eV over the Li-24d site (3.75 eV), possibly due to the preference for octahedral coordination (Supplementary Fig. 2b). Although this energy is high, it can be overcome by the thermal energy supplied during sintering^43^. The results suggest a strong energetic preference for Ta to reside at GBs rather than Li sites, as deduced from the 2.64 eV difference between the Frenkel defect formation energy and the 0.08 eV energetic preference for Zr sites over GBs. Thus, when Ta ions diffuse along Li sites, they can ultimately return to Zr vacancies or segregate into GBs. The formation energies of La Frenkel defects at tetrahedral and octahedral Li sites in cubic LLZO are 2.45 and 2.48 eV, respectively (Supplementary Fig. 2b). As with Ta, these energies could be overcome during sintering. However, considering energetic preference of La for the crystalline phase over amorphous configurations of 2.50 eV, La exhibits negligible thermodynamic preference between GBs and Li sites in the bulk. Therefore, La could only segregate to GBs with the help of strong kinetic factors and relatively fast diffusivity along the Li migration channels.

The transport properties of Al, Ta and La in cubic LLZO were further studied by large-scale machine-learning force field-assisted molecular dynamics (MLFF-MD) simulations using three large representative GB models, Σ17 (530) [001], Σ33 (52-2) [011], and Σ19 (32-5) [111], as well as a cubic LLZO model (Supplementary Fig. 2a). Each GB model extends 15 nm along the *c*-axis (normal to the GB plane), thus approximating the actual segregation scale as closely as possible. The results show that Al, Ta, and La can diffuse through Li-ion channels in the cubic LLZO lattice at high temperatures close to the sintering environment (Fig. 1e and Supplementary Fig. 2c, d), with activation energies of 0.619 eV for Al, 0.645 eV for Ta, and an unexpectedly low value of 0.248 eV for La (Fig. 1f and Supplementary Table 2). It should be noted that similar La diffusivities in cubic LLZO were also determined from ab-initio molecular dynamics (AIMD) simulations (Supplementary Fig. 2e). The low activation energy of La can be attributed to its similar energetic preference for the Li-24d and 96 h sites in cubic LLZO, as indicated by nearly identical La defect formation energies at these sites (with a difference of only 0.02 eV), which could enable easy hopping between Li sites without site preference. In contrast, Ta and Al preferentially occupy the Li 96 h and 24 d sites, with differences in formation energies of 1.03 eV (as discussed) and 1.38 eV^44^, respectively, creating an energetic barrier to site-to-site hopping. The predicted diffusivities of Al and Ta at 1000–1300 °C are on the order of 10^−8^ cm^2^ s^−1^ in cubic LLZO, comparable to those of Li at 25 °C^45^, while La exhibits even higher diffusivities exceeding 10^−6^ cm^2^ s^−1^ in the same temperature range (Fig. 1e). These results imply that all three elements diffuse rapidly during sintering and can reach the GB regions. In GBs, the predicted activation energies can be higher than 0.9 eV, with an increase of up to 0.24 eV for Ta, 0.37 eV for Al, and 0.76 eV for La compared to cubic LLZO (Fig. 1f and Supplementary Table 2). The diffusivities of Al and La are about 10^−7^ cm^2^ s^−1^, and that of Ta is on the order of 10^−8^ cm^2^ s^−1^ in the GBs, suggesting a spatiotemporal evolution of GB configurations with fast mobilities of the elements at high temperatures. Due to the high activation energies, the diffusivities of the dopants in GBs are in the range of 10^−16^–10^−20^ cm^2^ s^−1^ at 25 °C, so that the GB structure is frozen and the dopants can be kinetically trapped at the GB regions after cooling at the end of sintering.

In our experiments, strong, moderate, and slight segregation of Ta, La, and Al ions, respectively, was observed (Fig. 1b, c). Based on our simulation results, this trend can be explained by a combination of thermodynamic and kinetic factors: Ta has a strong thermodynamic driving force of 2.64 eV and a fast diffusivity of about 10^−8^ cm^2^ s^−1^ along the Li channel in cubic LLZO, which promotes significant segregation at the GBs. Al has a much lower thermodynamic driving force of 0.42 eV and a similar diffusivity to Ta, resulting in slight segregation in the GB regions. Although La has a negligible thermodynamic driving force, its diffusivity through the Li-ion channels is more than two orders of magnitude faster than that of Ta and Al, which significantly increases the probability of La reaching GBs and leads to moderate segregation. These results suggest that elemental segregation at GBs in LLZO is governed by both thermodynamic and kinetic contributions. Our conventional sintering experiments combined with atomistic simulations using DFT and MLFF clarify the mechanisms of elemental segregation and underscore the critical role of kinetic factors. In particular, longer sintering durations provide sufficient time for Al, Ta, and La to migrate over longer distances within LLZO and for GBs to evolve, thereby increasing the likelihood of their segregation at thick and disordered GBs. Therefore, sintering strategies that enable effective densification within shorter timeframes are essential for minimizing elemental segregation.

### Sintering of high-density and segregation-free LLZO

**Fig. 2:**
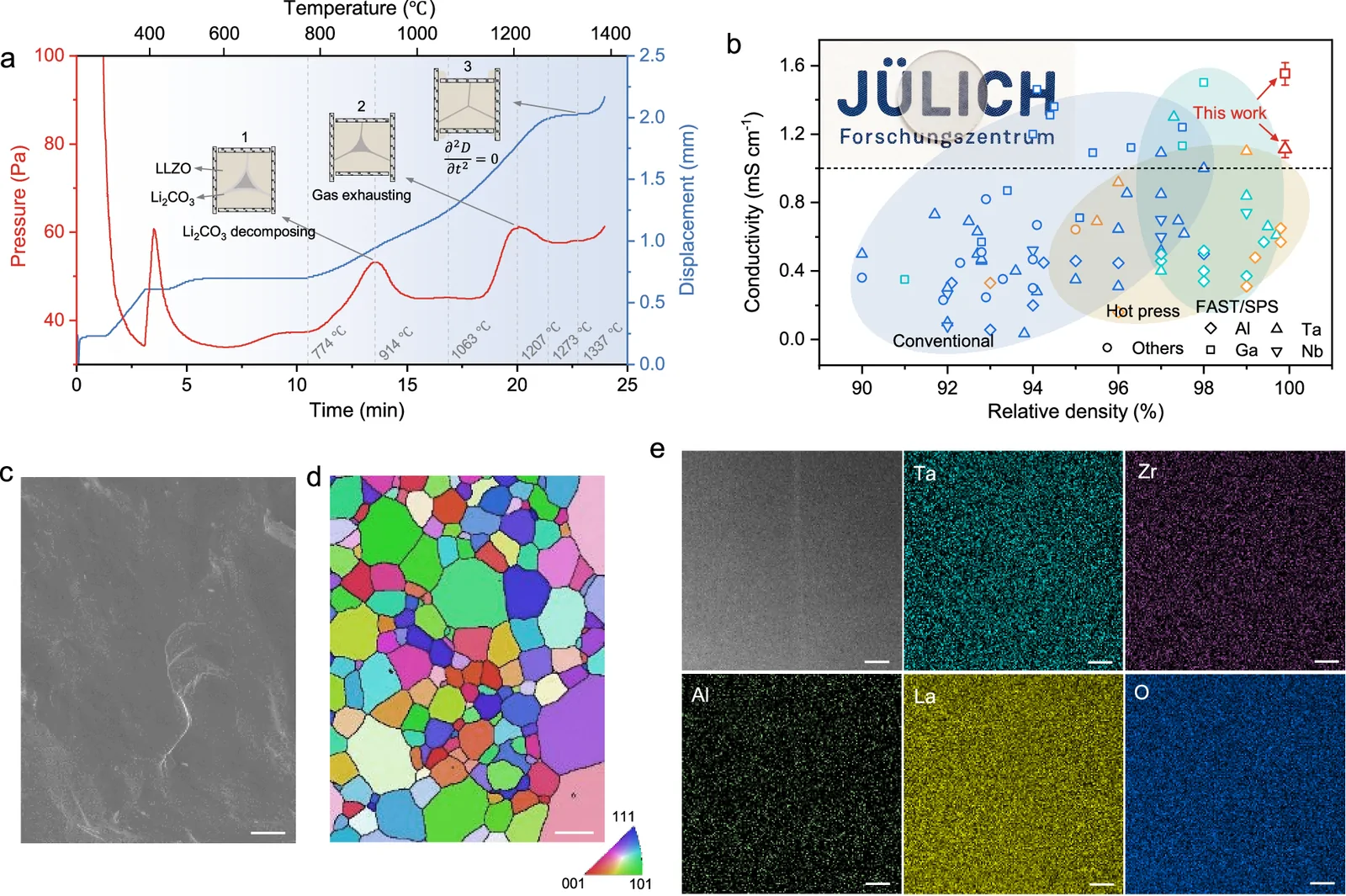
Sintering of high-density and segregation-free LLZO. **a** Displacement and chamber pressure records during FAST/SPS process. The insets are schematic diagrams of LLZO sintering dynamics. **b** Conductivities plotted against relative densities comparing various doped LLZO synthesized via conventional sintering, hot press, FAST/SPS, and this work with error bar. Conductivity data are presented as mean values of 5 separately sintered pellets +/− SD. The source of the literature data shown in this figure can be found in Supplementary Table 3. **c**, **d** Representative SEM (**c**) and EBSD images (**d**) of SF-LLZO. The scale bars are 20 µm. **e** Representative HAADF-STEM image and STEM-EDS mappings of the GB of SF-LLZO. The scale bars are 50 nm.

Here, we have developed a rapid and controllable sintering strategy to produce segregation-free LLZO (SF-LLZO) using Field Assisted Sintering Technology/Spark Plasma Sintering (FAST/SPS) (details in Supplementary Fig. 3a, b). During the sintering process, the chamber pressure and sample displacement curves were recorded to monitor gas release from the sample and track its deformation (Fig. 2a). The results show that powder densification occurs in two main steps: In the first step, from 774 to 1063 °C, the chamber pressure reaches a peak due to the decomposition of Li_2_CO_3_ and the release of CO_2_, while the displacement curve shows a gradual increase (inset 1). The second step begins above 1063 °C, where the displacement slope becomes significantly steeper, coinciding with a second pressure peak at 1207 °C (inset 2) caused by particle deformation and gas ejection between the particles. During this phase, rapid densification occurs as displacement increases steadily up to 1273 °C. Beyond this point, the displacement rate decreases, indicating that the relative density of the sample is close to the theoretical density. It is noteworthy that the rate increases again at temperatures above 1337 °C when LLZO softens and is partially pressed out of the die (Inset 3). The process exhibits a mathematically identifiable inflection point (${\partial }^{2}\varDelta D/\partial {t}^{2}=0$, where *D* is the displacement) after the gas has completely escaped. This point represents the critical softening point of the solid phase and can be determined individually for different ceramic powders with different compositions (Supplementary Fig. 3c). In our sintering protocol, the inflection point is used as a practical indicator of the optimal endpoint, and the sintering process is terminated immediately after this point to obtain nearly full dense pellets. The entire process was completed within 23 min, with only 3 min above 1200 °C, thereby effectively minimizing long-range migration of elements and Li loss, and preserving ~6.6 Li atoms per formula unit.

The pellets sintered at the densification inflection point exhibit high optical transparency, as shown in the inset in Fig. 2b, indicating their high density with minimal defects. A review of the existing literature shows that conventional sintering achieves a wide range of relative densities between 90 and 98% (Fig. 2b and Supplementary Table 3 for detail data). The application of pressure during sintering generally improves densification, with hot pressing yielding pellets with relative densities of 95 to 99.5%, while FAST/SPS produces the highest average relative density of approximately 98.5%. The data show that the conductivities in samples with a given composition correlate positively with the relative densities, with Ga-LLZO and Ta-LLZO exhibiting high conductivities of over 1 mS cm^−1^ at 25 °C (Fig. 2b). In comparison, all of our samples produced at the inflection point exhibit relative densities of over 99.9%, resulting in ionic conductivities that are among the highest reported for LLZO with various dopants. We have produced samples with a total ionic conductivity at 25 °C of 1.55 mS cm^−1^ for Ga-doped LLZO and 1.11 mS cm^−1^ for Al and Ta-doped LLZO. Unlike the conventional method, which relies on fixed times and temperatures, the sintering behavior in the FAST/SPS procedure can be monitored using sample displacement and gas exhaust curves, enabling real-time intervention at the optimal point (inflection point) to produce high-quality LLZO. We have successfully used this process to produce various high-density oxide ceramics, not only for LLZO with different dopants, but also for other CEs (Supplementary Fig. 3c), confirming the broad applicability of this protocol for a wide range of oxide ceramics that achieve near-complete density with minimal microstructural defects.

The SF-LLZO (doped with Al and Ta) processed using the inflection point-guided FAST/SPS method with the same powder as in the conventional sintering described above exhibits a highly crystalline cubic phase (space group Ia-3d) with a refined lattice parameter of 12.97(3) Å, which was confirmed by synchrotron XRD (Supplementary Fig. 4a)^46^. We also found no influence of the slightly reducing conditions of FAST/SPS (in contrast to the oxidizing atmosphere during conventional sintering in air) on the chemical environment of the constituent elements, as the X-ray photoelectron spectra (XPS) of SF-LLZO and S-LLZO are virtually identical (Supplementary Fig. 4b). SEM imaging shows a transgranular fracture morphology with a smooth, dense surface, which contrasts sharply with the intergranular fracture observed in S-LLZO, suggesting that the GBs in SF-LLZO have a mechanical strength comparable to that of the grain interior (Fig. 2c). Electron backscatter diffraction (EBSD) analysis on polished cross-sections confirms the polycrystalline nature of the sample with an average grain size of ~10 μm, which corresponds to the particle size distribution of the starting powder (Fig. 2d and Supplementary Fig. 4c). This consistency implies that grain growth via GB migration was suppressed during FAST/SPS, supporting a sintering mechanism dominated by grain deformation and pore elimination. Uniform element distribution in SF-LLZO was observed by SEM-EDS (Supplementary Fig. 4d). HAADF-STEM reveals a thin and clean GB (Fig. 2e and Supplementary Fig. 4e–h), and the corresponding STEM-EDS mapping further confirms that no statistically significant segregation is detected within the measurement accuracy (Supplementary Table 4). When the SF-LLZO sample is annealed again in a conventional sintering process, slight Ta segregation can be observed, confirming the kinetic control of element segregation (Supplementary Fig. 4i). In addition, the sample synthesized by FAST/SPS under a slower heating rate (10 K min^−1^) and a prolonged holding time (1 h) produced a secondary phase, which can be distinguished by the distinctly dark regions in the HAADF image (Supplementary Fig. 4j).

### Effect of GB segregation on Li-ion conductivity

**Fig. 3:**
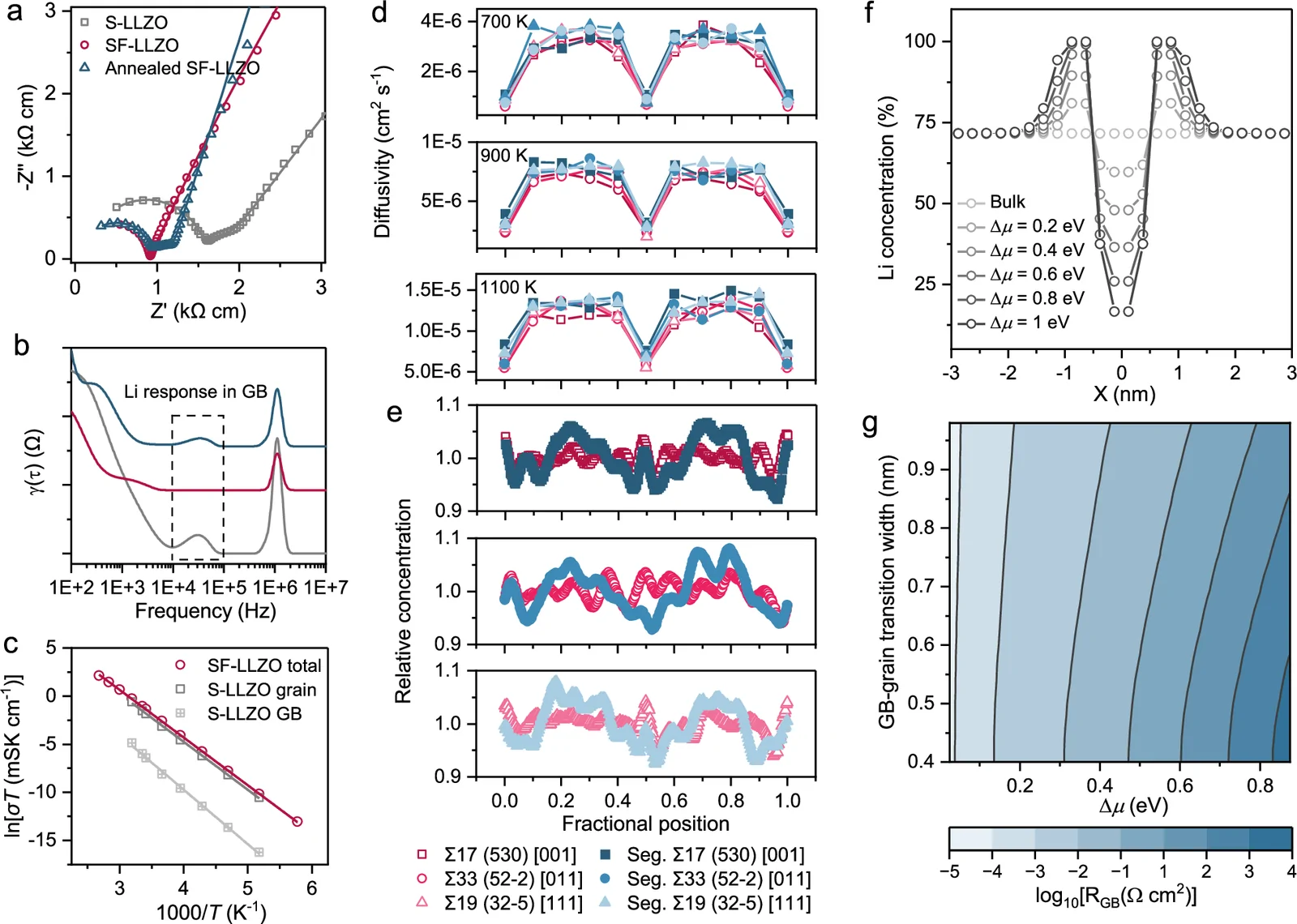
Effect of GB segregation on Li-ion conductivity. **a**, **b** EIS (**a**) and corresponding DRT analysis (**b**) of S-, SF-, and annealed SF-LLZO measured from 7 MHz to 1 Hz at 25 °C. **c** Arrhenius plots of S-LLZO and SF-LLZO over the temperature range from −100 to 100 °C. The GB plot is fitted based on the specific resistance. **d**, **e** MLFF-MD for Li diffusivities at 700, 900, and 1100 K (**d**) and Li concentrations (**e**) for Ta-segregated and non-segregated GB Σ17 (530) [001], Σ33 (52-2) [011], and Σ19 (32-5) [111]. 10 simulations were performed at each temperature. **f**, **g** Calculated Li concentration as a function of Li chemical potentials (**f**) and resistance map as a function of Li chemical potentials and GB-grain transition width (**g**).

The influence of elemental segregation on Li-ion transport properties in S-LLZO and SF-LLZO was quantitatively evaluated using electrochemical impedance spectroscopy (EIS) (Fig. 3a and Supplementary Table 5). The S-LLZO sample exhibits two distinct arcs, a characteristic feature of garnet-type electrolytes^12^. The high-frequency arc (~4 MHz) corresponds to Li-ion transport within the grains with a fitted capacitance of 8.05 × 10^−11^ F, while the mid-frequency arc (~60 kHz) is attributable to Li-ion conduction across the GBs, with a fitted capacitance of 2.1 × 10^−8^ F. In contrast, the SF-LLZO shows only a single arc in the high-frequency region (~7 MHz), which corresponds to grain impedance, with no discernible contribution from the GBs. Distribution of relaxation time (DRT) analysis further confirms the absence of peaks in the range of 10^4^–10^5^ Hz for SF-LLZO (Fig. 3b)^42^, indicating negligible GB resistance. It is noteworthy that when SF-LLZO is annealed in air, an additional arc reappears in the impedance spectra, accompanied by a corresponding peak in the DRT analysis (Fig. 3a, b). This is attributed to the evolution of GB with Ta segregation during annealing (Supplementary Fig. 4e).

Fitting the Arrhenius plots over a temperature range of −100 to 100 °C shows that the total activation energy in SF-LLZO is 0.427 eV, which is almost identical to the grain activation energy in S-LLZO (0.429 eV), while the presence of GBs in S-LLZO increases the activation energy to 0.487 eV (Fig. 3c). This highlights the negligible GB impedance in SF-LLZO with thin GBs without segregation.

Elemental segregation can affect Li-ion conductivity by increasing the activation energy and/or altering the Li concentration, as described by the following equation^47^:

1 $$\sigma=\gamma \left(N{q}^{2}/T{c}_{\max }^{2}\right)c\left({c}_{\max }-c\right){a}^{2}{\nu }_{0}exp \left({E}_{a}/{k}_{B}T\right)$$

where γ is the correlation factor, N is the number density of available hopping sites, q is the ionic charge, T is the temperature, c is the ionic concentration, ${c}_{\max }$ is the maximum ionic concentration, a is the hop distance, ${\nu }_{0}$ is the attempt frequency, ${E}_{a}$ is the activation energy of charge carrier hopping and ${k}_{B}$ is the Boltzmann constant. To evaluate the influence of segregation on Li-ion conductivity, Li-ion mobility was investigated using MLFF-MD simulations using the three large GBs (Σ17 (530) [001], Σ33 (52-2) [011], and Σ19 (32-5) [111]) with and without Ta segregation. Certain frames during simulation process are provided in Supplementary Data 2. In the non-segregated models, Ta is distributed homogeneously throughout the grain interiors and GB regions, while in the segregated models, the Ta concentration is enriched at the GBs, corresponding to the experimental concentration profiles observed in transmission electron microscopy (TEM) (Fig. 1c and Supplementary Fig. 5a–c). The Li-ion self-diffusivities in the GB models were analyzed as a function of the *z*-axis fractional coordinates, as shown in Fig. 3d. The results show no significant difference in Li-ion diffusivities between Ta-segregated and non-segregated GB models, although both exhibit significantly lower diffusivity in the GB regions compared to the grain interiors. The calculated activation energies of Li-ion transport in the GB models are 0.341 eV (segregated) and 0.336 eV (non-segregated), averaged over three GBs, with both values being higher than the bulk value of 0.229 eV (Supplementary Fig. 5d and Table 6). Furthermore, the Li positional probability density maps at 1100 K confirm similar migration behavior in both models (Supplementary Fig. 5a–c). The results suggest that Ta segregation itself does not directly impede Li-ion transport at GBs. Thus, the observed reduction in Li conductivity would be attributable to disordered structural features and possibly to segregation-induced structural evolution. Accordingly, the mid-frequency arc observed in S-LLZO (Fig. 3a) is not necessarily a direct consequence of element segregation, but is more likely attributed to morphological evolution, in particular the formation of thicker and more disordered GBs during conventional sintering compared to the thinner GBs in SF-LLZO.

Although Ta segregation does not significantly affect Li-ion self-diffusivity and activation energy, it can reduce Li-ion concentration and thus affect Li-ion conductivity at GBs. MLFF-MD simulations reveal that, after reaching equilibrium, the relative Li-ion concentration in the presence of Ta segregation is lower at the GBs than within the grains (Fig. 3e), in contrast to the distribution of Ta (Supplementary Fig. 5a–c). This reduction is attributed to the higher oxidation state of Ta compared to Zr, which requires a lower Li-ion concentration for charge compensation. This trend is further supported by the radial distribution function (RDF) analysis (Supplementary Fig. 5e), showing that the average coordination number of Li around Ta within 3.05 Å is 2.11 (the first minimum point in RDF) and thus lower than the Zr–Li coordination number of 2.86. Therefore, according to equation (1), a reduction in carrier concentration c within a certain range can lead to a corresponding decrease in Li-ion conductivity at the GBs. Although this simulation focuses on Ta, Al segregation is expected to produce the same effect. Since Al preferentially occupies Li sites in the LLZO framework and carries a charge state of 3+, it can substitute up to three Li-ions to maintain charge neutrality, further exacerbating Li-ion depletion at the GBs.

The increased resistance in segregated GBs cannot be attributed solely to the difference in activation energies between GB and grain interior, as shown by the MLFF-MD results (Fig. 3d). Quantitative estimates based on a GB width of ~2 nm (Fig. 1c) and a grain size of ~10 μm (Supplementary Fig. 4c) further show that activation energy differences contribute less than 1% to the total GB resistance. One possible explanation for the increased resistance is concentration variations, i.e., space charge layers (SCLs)^48,49^. SCLs can arise from the difference in chemical potential between Li ions at the GBs (${\mu }_{{GB}}$) and the bulk phase (${\mu }_{B}$), which is due to different site configurations and site energies in the two phases, both of which may be influenced by dopants. We define a chemical potential difference $\varDelta \mu$ as the difference between ${\mu }_{{GB}}$ and ${\mu }_{B}$. If Li can move between the GB and the bulk phase, the Li-ions will equilibrate, resulting in space charges arising from either the negatively charged background lattice or the displacement of positively charged Li ions. The local electric field and chemical potential are balanced in equilibrium state. The enrichment or depletion of Li affects the GB resistance and can increase the probability of metal Li nucleation or the formation of Li compounds at the GB edges, as it changes the local number of Li-ions. To model the Li distribution across the GBs in equilibrium, we use a continuum simulation framework based on the Poisson equation and a diffusion-drift equation (see the “Method” Section for the simulation framework and Supplementary Table 7 for model parameters). Assuming that only the activation energy and concentration affect the difference in resistance between the bulk and GB, we identified $\varDelta \mu$ required to achieve the expected GB resistance by performing a parameter study for $\varDelta \mu$ and the GB-grain transition width (Fig. 3f, g). The transition width in our simulations is the width over which $\varDelta \mu$ changes smoothly from the GB core to the bulk. Figure 3f shows the Li concentration as a function of positions normal to GB plane as a function of $\varDelta \mu$, suggesting that and an increase in $\varDelta \mu$ leads to Li enrichment at the GB edges and depletion in the GB core. This implies the formation of SCL with Li segregations at the GB edges during GB evolution, which is expected to be more pronounced in S-LLZO than in SF-LLZO, thereby increasing the GB resistance. It should be noted that the elevated concentrations outside the GB core would contribute substantially to the overall resistance, as the limited number of available sites restricts ion hopping (Eq. (1)). Figure 3g shows the relationships between the transition width, the $\varDelta \mu$, and the resistance. Based on literature data^50^, our EIS measurements, and the grain size distribution, a single GB in conventionally sintered LLZO has a resistance of 0.1–0.2 Ω cm^2^. This resistance corresponds to the $\varDelta \mu$ value of 0.47–0.62 eV in a transition width range between 0.4 and 1.0 nm (Fig. 3g). Our DFT calculations with cubic and amorphous LLZO phases suggest a large $\varDelta \mu$ value of 1.3 eV (Supplementary Fig. 5f, g). The simulation also shows that the GB resistance drops sharply with decreasing $\varDelta \mu$. The segregation-free GBs in SF-LLZO are likely to have a small $\varDelta \mu$ value, as the differences in structural properties between the clean GB and bulk LLZO are minimized due to limited structural evolution at the GBs during the short sintering time. This explains the negligible GB resistance observed in our experiments.

### Effect of GB segregation on Li nucleation

Li-ions in LLZO exhibit high chemical reactivity: exposure to ambient air easily leads to Li^+^/H^+^ exchange^51^, and Li-ions can be reduced to metallic Li under electron beam irradiation on the LLZO surfaces (Supplementary Fig. 6a)^52^. STEM characterizations of conventionally sintered LLZO doped with Al, Ta, or Ga reveal the presence of dark spots along the GB edges in HAADF images (Fig. 1b and Supplementary Fig. 6b, c). We hypothesize that during specimen preparation (e.g., ion milling), surface charging of the sample promotes the precipitation of Li on the LLZO surface. The deposited Li is then likely to be rapidly oxidized, forming Li-containing compounds (e.g., Li_2_O or Li_2_CO_3_) during sample transfer. These Li containing compounds consist of relatively light elements, or Li precipitation may induce local structural collapse and nano porosity, both of which would lead to reduced HAADF contrast and thus appear as dark spots in the images. The emergence of Li compounds correlates strongly with local structural features: they preferentially form in grains oriented along the 〈111〉 zone axis (Supplementary Fig. 6d–f) and are consistently located bilaterally adjacent to the GB cores within a narrow region of ~2–3 nm, but not within the GB core itself (Fig. 1b and Supplementary Fig. 6b, c). In contrast, no such secondary phases were observed in STEM analyses of segregation-free GBs (Fig. 2e).

**Fig. 4:**
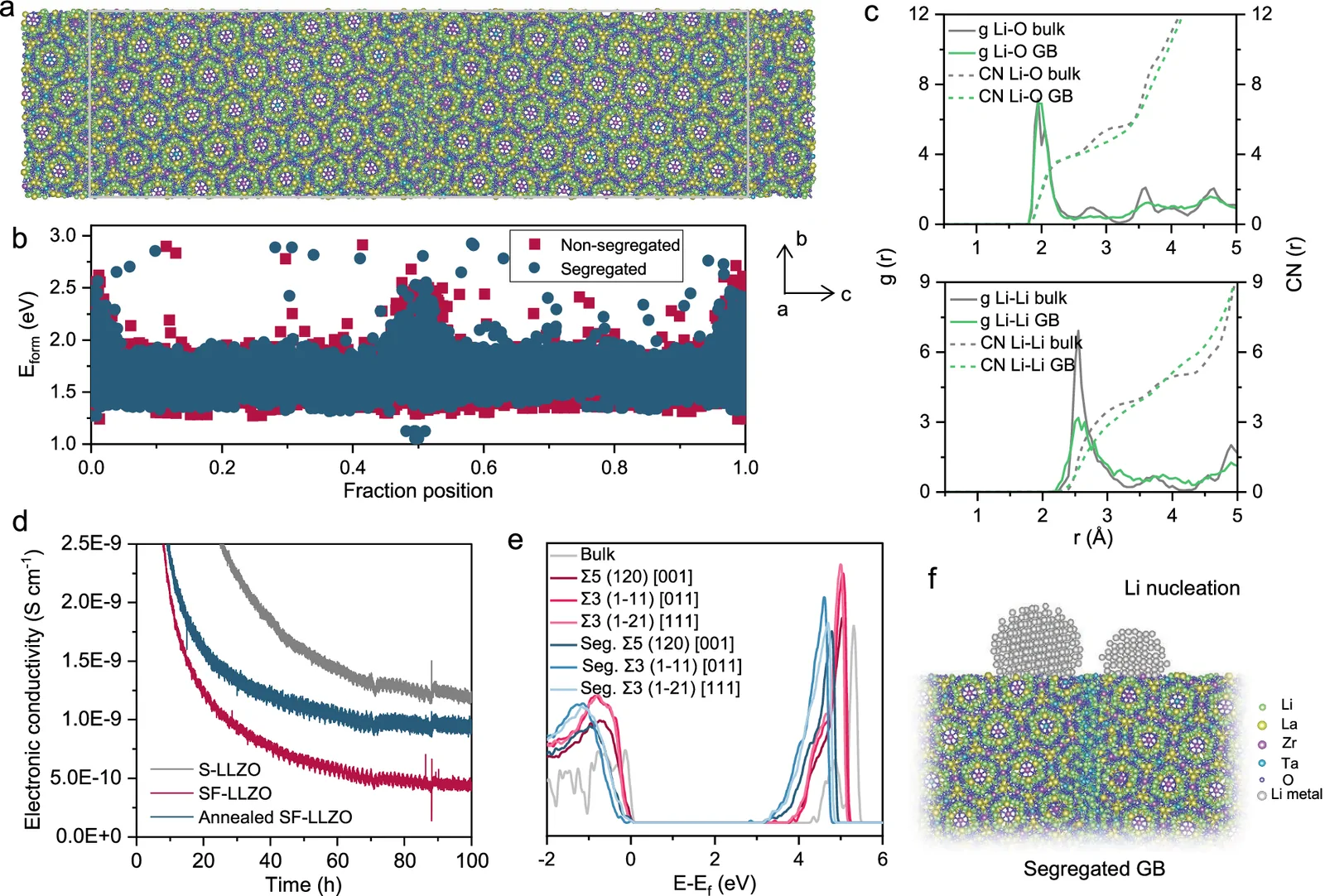
Effect of GB segregation on Li nucleation. **a**, **b** GB Σ19 (32-5) [111] (**a**) and calculated Li vacancy formation energies (**b**) with and without Ta segregation. **c** RDFs of Li–O and Li–Li in the bulk and the GB core in segregated GB Σ19 (32-5) [111]. **d** DC polarization measurements for S-, SF-, and annealed SF-LLZO pellets tested at 25 °C with a 0.2 V bias. **e** Band structures of bulk LLZO and Ta-segregated and non-segregated GB Σ5 (120) [001], Σ3 (1-11) [011], and Σ3 (1-21) [111]. In segregated models, about 40% of Zr are replaced by Ta, with 10 randomly-doped models generated and the case for each GB with the smallest band gap plotted. **f** Schematic diagram illustrating Li nucleation. Green, yellow, cyan, purple, and blue spheres denote Li, La, Ta, Zr, and O in LLZO, respectively, while gray represents metallic Li.

The local structure-dependent reactivity of Li-ions is closely related to their electrochemical nucleation behavior, which can provide microscopic insight into how GB segregation influences Li plating and dendrite formation. The formation energies of Li vacancies, in conjunction with local electronic properties, synergistically serve as key mechanistic intermediaries, as the feasibility of Li extraction and subsequent reduction to initiate nucleation is determined by the energy required to form Li vacancies and the availability of electrons^13,18^. Therefore, we calculated the Li vacancy formation energy for the three representative GBs (Fig. 4a and Supplementary Fig. 7a, d) with and without Ta segregations using the developed MLFF. The vacancy formation energy was calculated using the energy of bcc Li metal as a reference, thereby also taking into account the thermodynamic cost of Li metal formation. The results demonstrate that the formation energy at the GB is consistently higher than in the bulk region, regardless of segregation (Fig. 4b and Supplementary Fig. 7b, e). In the bulk region, the formation energy generally remains below 2 eV, while in the GB core with a width of ~1.5–2.0 nm, it can reach up to 2.5 eV and exhibits a peak-like distribution. These results suggest that the increased formation energy of Li vacancies originates from the intrinsic structural characteristics of the GB core rather than from segregation. Therefore, Li extraction and nucleation at the GB core are more difficult than in the grain interior and at the GB edges, which are thermodynamically more favorable.

To reveal the structural properties that determine the formation energy of Li vacancies at GB cores, we performed radial distribution function (RDF) analyses for cation-oxygen and cation-cation pairs to compare the local coordination environments in the GB and bulk regions with and without Ta segregation (Fig. 4c and Supplementary Fig. 7c, f, g). The comparison between RDFs with and without Ta segregation shows minimal differences in the local coordination structures across all three GBs, which explains the negligible variation in Li vacancy formation energies observed between segregated and non-segregated GBs (Fig. 4b and Supplementary Fig. 7b, e). This also explains the negligible influence of Ta segregation on Li diffusion at GBs, as shown in Fig. 3d. Detailed analysis of the RDFs suggests that the increased Li vacancy formation energy at GB cores originates from two primary structural factors: reduced electrostatic repulsion between cations and increased Li–O bond strength at GBs compared to the bulk. The absence of a second peak in the RDF of Li–O pairs at GBs suggests that most Li-ions at GBs adopt a tetrahedral coordination, in contrast to the mixed tetrahedral and octahedral coordination observed in the bulk (Fig. 4c). Since tetrahedral Li–O bonds are shorter and stronger than octahedral ones, this implies a higher average Li–O bond strength at GBs. Furthermore, the RDFs of Li–Li, La–Li, and Zr–Li pairs indicate lower first-neighbor coordination numbers at GBs, suggesting weaker cationic repulsion acting on Li-ions compared to the bulk (Fig. 4 and Supplementary Fig. 7c, f, g). Therefore, the reduced electrostatic repulsion and enhanced Li–O interactions deepen the potential energy landscape at GB cores, making the removal of Li-ion energetically more difficult. Consequently, the formation energy of Li vacancies at GB cores is increased, suppressing the thermodynamic driving force for Li metal nucleation.

In addition to the formation energy, the space charge regions calculated above influence the reactivity of Li near GBs. The number of Li-ions in the GB is lower in the GB core and higher at the GB edges (Fig. 3f). This leads to a higher reaction probability at the GB edges with higher Li-ion populations than in the GB cores, promoting the formation of Li compounds or Li nucleation. The result is directly consistent with our observation by HAADF-STEM (Fig. 1b), which shows Li compounds at the GB edges in S-LLZO with increasing $\varDelta \mu$. Therefore, the formation of SCLs drives the preferential Li nucleation at the GB edges.

Previous theoretical and experimental studies have shown that GBs with higher electronic conductivity facilitate dendrite initiation and propagation^13,18^. To evaluate the influence of elemental segregation on electronic conductivity, a 100-h DC polarization experiment was performed (Fig. 4d). The results show that SF-LLZO exhibits electronic conductivity that is approximately one order of magnitude lower than that of segregated samples, including S-LLZO and air-annealed SF-LLZO, and that the presence of GBs actually correlates with increased electronic conductivity of polycrystalline LLZO at the macroscopic level. The reported experimental results of other groups, such as Liu et al., show that LLZO GBs can have a markedly reduced bandgap (1–3 eV), in sharp contrast to the grain interior (5.7–5.8 eV)^13^. To evaluate how GB segregation affects the bandgap, we performed DFT calculations for three GB models using the generalized gradient approximation (GGA) level of theory. The GB models are provided in Supplementary Data 3. As shown in Fig. 4e, bulk LLZO has a band gap of 4.35 eV, while the band gap at GBs is about 0.6–0.9 eV lower. With Ta segregation, the average band gap decreases further by 0.2–0.3 eV, with the maximum decrease reaching 0.6 eV. Therefore, our calculations support experimental observation by Liu et al. regarding the higher electronic conductivity of GBs. In addition to dopant segregation, other GB effects that were not explicitly considered in our DFT calculations may also contribute to the observed difference in electronic conductivity. For example, differences in GB thickness, such as the thinner GBs in SF-LLZO compared to S-LLZO, could influence the local electronic structure and therefore affect the electronic conductivity. The higher electronic conductivity of GBs, together with the higher concentration of Li-ions at the GB edges, will fertilize the reduction of Li-ions and dendrite formation along the GBs. It should be noted that the present DFT analysis is intended to provide a qualitative assessment within a consistent computational framework and to offer mechanistic insights into GB electronic conductivity, rather than to quantitatively predict absolute bandgap values, given the intrinsic structural complexity of GBs and the limitations associated with the choice of exchange-correlation functional.

By integrating experimental observations and theoretical calculations, a comprehensive picture of the Li reaction in LLZO is proposed (Fig. 4f). This model not only accounts the TEM observations, but also suggests a possible microscopic mechanism for electrochemical Li nucleation. The increased Li vacancy formation energy at the GB core relative to the bulk reduces the tendency of Li-ions at the core to participate in reactions. Meanwhile, GBs act as pathways for electronic conduction, allowing nearby electrons to participate in Li reduction. Consequently, Li preferentially nucleates along the GB edges during electrochemical cycling or post-processing. Ta segregation further promotes this nucleation behavior by enhancing the electronic conductivity of the GB edges. These results suggest the mechanism Li nucleation at microscale during battery operation: Li clusters could initiate a few nanometers away from the GB core and then grow and coalesce into Li metal grains (Fig. 4f). This leads to electrochemical plating of Li along the GBs as observed in operando SEM observations at the mesoscale^20^, and suggests that Li stripping can occur in the vicinity of GBs, consistent with observations by operando TEM^19^. Elemental segregation at the GBs disrupts the local balance of Li-ion flux, leading to Li depletion at the Li|solid electrolyte interface, the formation of voids, and localized Li accumulation. This can induce the formation of dendrites from the LLZO surface, which penetrate the separator along the GBs^21^. Alternatively, segregation may promote Li nucleation within the polycrystalline LLZO microstructures, where isolated clusters can interconnect into dendrites, ultimately causing soft or hard short-circuits. These results align with reports suggesting that dendrite formation results from a combination of surface-initiated and bulk-mediated mechanisms^13,21^. Notably, the SF-LLZO, characterized by its high density and segregation-free GBs with improved electronic insulation, holds promise as a separator for battery applications by enabling more uniform Li plating/stripping, improved Li transport, and increased dendrite tolerance.

### Dendrite tolerance assessment

**Fig. 5:**
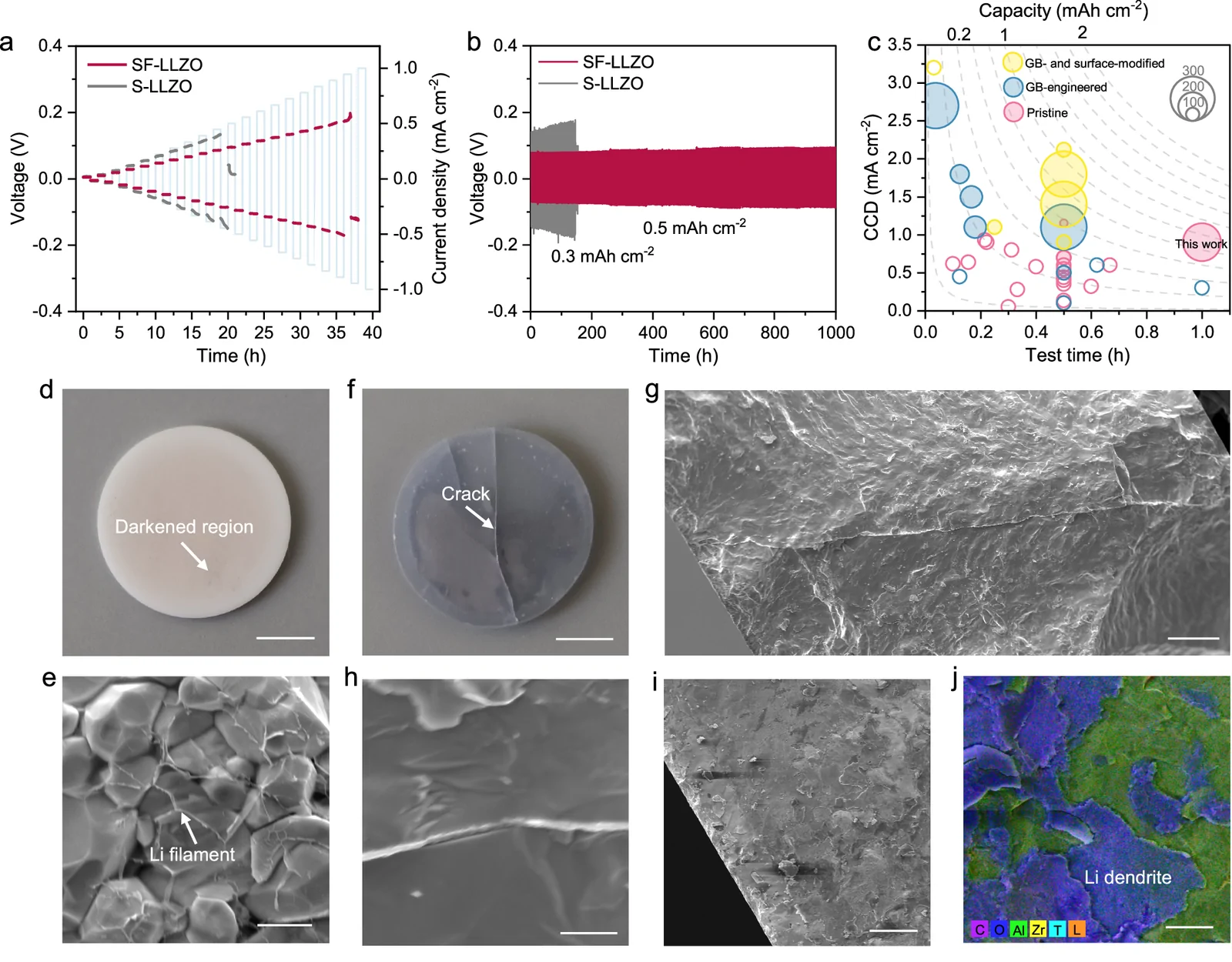
Dendrite tolerance assessment. **a**, **b** CCD (**a**) and galvanostatic cycling (**b**) measurements of S-LLZO at 0.3 mA cm^−2^ and SF-LLZO at 0.5 mA cm^−2^ at 25 °C. **c**, Comparison of reported CCDs and CCD capacities of LLZOs, together with total accumulated capacities during galvanostatic cycling (bubbles). Pink, blue, and yellow bubbles denote pristine, GB-engineered, and both GB- and surface-modified LLZO, respectively. Filled bubbles indicate accumulated capacities and open bubbles indicate <50 mAh cm^−2^ or not provided. Current densities are normalized to the effective Li/LLZO contact area. The source of the literature data shown in this figure can be found in Supplementary Table 8. **d**, **e** Post-mort**e**m analysis after dendrite penetrating S-LLZO (removing one Li electrode), shown by an optical top-view image (**d**) and local cross-sectional SEM images at dark regions (**e**). Scale bars are 3 mm (**d**), 5 μm (**e**). **f**–**j** Post-mortem analysis after dendrite penetrating SF-LLZO (removing one Li electrode), shown by an optical top-view image (**f**), local cross-sectional (**g**, **h**), and fractured SEM image and EDS overlay mapping at cracks (**i**, **j**). Scale bars are 3 mm (**f**), 100 μm (**g**), 5 μm (**h**), 50 μm (**i**), and 10 μm (**j**). Batteries for post-mortem analysis were disassembled after 500 plating/stripping cycles with 2 h per cycle at current densities of 0.5 mA cm^−2^ for S-LLZO and 1 mA cm^−2^ for SF-LLZO at 25 °C.

The dendrite resistance of segregation-free, nearly fully dense (99.9%) SF-LLZO pellets was evaluated in symmetrical Li cells at 25 °C, with S-LLZO pellets as a reference. Critical current density (CCD) tests show that SF-LLZO can tolerate currents up to 0.9 mA cm^−2^, which is twice the limit of 0.45 mA cm^−2^ observed for S-LLZO (Fig. 5a). Upon reaching their respective CCD thresholds, both cells exhibit soft short-circuit behavior, indicative of initial dendrite formation without complete penetration. Galvanostatic cycling demonstrates the stability of SF-LLZO over 1000 h, in stark contrast to S-LLZO, which fails after 150 h, accompanied by increasing overpotentials indicating uneven Li plating/stripping (Fig. 5b). Symmetrical cells with SF-LLZO separators consistently exhibit lower polarization than cells with S-LLZO, primarily due to the higher ionic conductivity of SF-LLZO pellets and thus greatly reduced ohmic losses, resulting in an ohmic voltage drop of approximately 0.05 V. In addition, the denser surface morphology of SF-LLZO pellets likely allows for more effective contact with the Li metal, reducing interfacial resistance and resulting in more homogeneous Li plating/stripping. In CCD tests, SF-LLZO delivers a maximum single cycle capacity of 0.9 mAh g^−1^, with a cumulative capacity exceeding 250 mAh g^−1^ over long-term cycling. Compared to the state of the art, SF-LLZO outperforms most comparable polycrystalline LLZO pellets without GB modification or interface modification (Fig. 5c and Supplementary Table 8). Higher CCDs are only achieved by LLZO pellets that contain electronically insulating phases at GBs (e.g., LiF or ZrO_2_) introduced by GB engineering or that use lithiophilic interlayers to modify the Li|LLZO interface (Fig. 5c). It is noteworthy that SF-LLZO itself exhibits promising CCD capacity and cumulative capacity during galvanostatic cycling (Fig. 5c), even when benchmarked against these advanced modification strategies. It is worth mentioning that the fabrication of SF-LLZO offers clear advantages in terms of energy cost and process simplification, as it is produced in short high-temperature process without the introduction of additional modification materials.

Post-mortem analysis of the cells after a hard short circuit also shows that segregated and segregation-free LLZO pellets exhibit differences in Li penetration mechanisms. In S-LLZO pellets, dark areas can be observed on the surface where dendrites form^21^ (Fig. 5d). Local cross-sectional SEM images of this area show that Li grows along the GBs and forms interconnected filaments (Fig. 5e). This is consistent with the reported bulk-mediated dendrite growth mode, where Li nucleation and interconnection occur along the GBs^13^. In contrast, SF-LLZO exhibits a distinctly different dendrite growth behavior, in which Li dendrites penetration is accompanied by crack formation (Fig. 5f, g). The cracks run either slanted or almost perpendicular to the surface through the entire pellet thickness without following the microstructure of the LLZO grains. The cracks are filled with lithium, which can be seen along the crack length and along the fractured surface (Fig. 5h–j and Supplementary Fig. 8), as confirmed by EDS mapping, where C and O are enriched (Li was oxidized to Li_2_CO_3_ during sample transfer), while Zr and La (LLZO) are absent (Supplementary Fig. 8). This behavior is very similar to the performance reported for single-crystal LLZO^53^, suggesting a surface-initiated dendrite growth mechanism in which dendrites propagate from one electrode to the other through SF-LLZO separator, while the high stress concentrated at the dendrite tips promotes crack initiation and development^54,55^. The distinctly different dendrite propagation behavior can be attributed to the differences in the structure of segregated and segregation-free GB discussed above, including differences in the local distribution of Li-ions and electronic conductivity. We therefore propose that the dendrite tolerance of polycrystalline LLZO separators is mainly determined by the morphology (relative density) and electronic conductivity of the GBs, the latter being strongly influenced by the dopants and the element segregation. After eliminating element segregation in SF-LLZO, the dendrite tolerance is largely determined by the mechanical properties (mainly fracture toughness) of the LLZO crystal structure and is close to maximum value expected for this material. A further increase in CCD would primarily require optimization of the mechanical properties of the polycrystalline LLZO scaffold and improving its ability to relief the stress occurring during Li plating, with research directions including optimization of particle size/particle size distribution and GB modification to improve the total ductility.

The promising performance of SF-LLZO was further validated in a quasi-solid-state coin cell at 25 °C employing high-voltage LiCoO_2_ as the positive electrode, with a drop of liquid electrolyte added at the electrode|LLZO pellet interface to improve contact and Li foil attached at the opposite side of the LLZO pellet as the negative electrode. Comparable rate performance at low current densities (<0.5 C) is observed for both S-LLZO- and SF-LLZO-based batteries (Supplementary Fig. 9a, b). However, at higher rates of 2 C, the S-LLZO cell suffers from increasing polarization, diminished discharge plateaus, and capacity loss, delivering only 86.9 mAh g^−1^, while the SF-LLZO cell maintains 146.9 mAh g^−1^, demonstrating its higher ionic conductivity. Extended cycling further highlights the durability of SF-LLZO under high-voltage operation. At 0.1 C between 3.0 and 4.45 V (vs. Li/Li^+^), the cell exhibits a negligible capacity decay from 172.3 mAh g^−1^ in the 5th cycle to 172.1 mAh g^−1^ in the 100th cycle (Supplementary Fig. 9c). Under a more aggressive 1 C cycling between 3.0 and 4.3 V, the cell delivers 143.7 mAh g^−1^ in the 5th cycle and retains 93.0% of its capacity after 300 cycles and 77.2% after 500 cycles. These results underscore that segregation-free, highly dense LLZO electrolytes enable improved electrochemical performance by minimizing GB resistance for uniform Li-ion flux and suppressing electronic conduction at the GBs to prevent dendrite-induced short-circuits.

Optimizing the performance of LLZO separators requires not only improving densification in the mesoscale range, but also controlling chemical homogeneity at the GBs in the nanoscale range. While the former requires a solid understanding of sintering dynamics, controlling the chemistry at the GBs necessitates fundamental insights into the mechanisms that govern element segregation. To elucidate these underlying principles and guide process optimization, we conducted a combined theoretical–experimental investigation of the segregation behavior of Al, Ta, and La in LLZO GBs during sintering, leveraging a multiscale modeling framework that integrates DFT- and MLFF-driven atomistic simulations and continuum-level modeling. Atomistic simulations show that these segregation phenomena arise from the thermodynamic and kinetic contributions: Upon the formation of Frenkel defects involving dopants at Li sites in cubic LLZO, Ta, Al, and La can diffuse along Li channels with distinctly different diffusivities and thermodynamic driving forces, ultimately leading to GB segregation. Given the strong kinetic dependence of segregation, a time-constrained sintering regime that ensures effective densification is essential to minimize elemental inhomogeneity.

In this work, we have developed a fast and controllable sintering protocol to synthesize high-density (99.9%) and segregation-free LLZO separators based on a mechanistic understanding of particle sintering dynamics obtained from the analysis of densification behavior. The critical softening point of the solid phase, marked by an inflection in the densification profile, is key to this optimization. The decisive influence of elemental segregation on the functional properties of LLZO is evidenced by the fact that SF-LLZO with suppressed elemental segregation exhibits negligible GB resistance, improved dendrite tolerance, and more than an order of magnitude lower electronic conductivity compared to its segregated counterpart (S-LLZO). Our experimental evidence and multiscale simulations show that while element segregation does not alter Li-ion diffusivity or the activation energy of Li-ion transfer, it does affect the ionic conductivity of LLZO through an inhomogeneous redistribution of Li ions, leading to the formation of SCLs with increased resistance. Contrary to currently accepted models, we have found that GBs have an intricate structure consisting of a GB core and edges with different chemical environments and Li concentrations. Segregation leads to a narrowing of the band gap at GB edges, increasing the risk of electronic leakage in S-LLZO. The increase in electronic conductivity, combined with the high formation energy of Li vacancies at the GB cores, explains the observed Li compounds at the GB edges and provides microscopic insights into Li plating along the GBs and a possible dendrite growth pathway.

Overall, this study reveals the mechanisms of elemental segregation in LLZO, its influence on intrinsic material properties, and its implications for the sintering process. The optimized sintering approach results in highly dense, segregation-free LLZO ceramic that serves as an ideal polycrystalline electrolyte for SSB applications. These results underscore the critical role of processing-microstructure-property relationships in the design of high-performance CEs. The developed sintering strategy suppresses detrimental GB characteristics while enabling dense, cost-effective, and reproducible LLZO separators, and can be extended to other ceramic ion conductors. Beyond LLZO, these insights into GB segregation and sintering behavior provide a general framework for the rational design of polycrystalline electrolytes for next-generation SSBs.

## Methods

### Materials synthesis

Synthesis of Li_6.45_Al_0.05_La_3_Zr_1.6_Ta_0.4_O_12_ powder utilized the following starting materials: LiOH∙H_2_O (99%, AppliChem GmbH, Darmstadt, Germany), La_2_O_3_ (99.9%, Merck KGaA, Darmstadt, Germany, pre-dried at 900 °C for 10 h), ZrO_2_ (99.5%, Alfa Aesar GmbH & Co KG, Karlsruhe, Germany), Ta_2_O_5_ (Inframat, 99.95%), and Al_2_O_3_ (99.9%, Inframat Advanced Materials LLC, Manchester, CT, USA). The materials were weighed in 100 × *g* batches according to their stoichiometric ratios with 20% LiOH∙H_2_O excess, homogenized using an electrical mortar grinder (RM 200, Retsch GmbH, Haan, Germany) for 1 h, and subsequently used to fabricate pellets. The pellets were pressed using a uniaxial press with a 45 mm diameter at a pressure of 20 MPa for 2 min. They underwent two calcination steps in Al_2_O_3_ crucibles: initially at 850 °C and then at 1000 °C for 20 h each. After each calcination step, the pellets were ground into powder and repressed. S-LLZO was prepared by pressing the synthesized powder into 13 mm diameter pellets at a pressure of 150 MPa for 2 min and sintering them in an Al_2_O_3_ crucible on a powder bed at 1175 °C for a dwell time of 10 h. All the sintering processes mentioned above are carried out in air with a heating ramp of 10 °C min^−1^, and the sintering is completed by natural cooling. For SF-LLZO preparation, an HPD5 FAST/SPS device (FCT Systeme) was used. In each experiment, 3 × *g* of prepared powder were loaded into graphite die with a diameter of 12 mm and pre-pressed at 50 MPa for 2 min to compact the powder. The die was then placed into the FAST/SPS chamber to run the program. The pressure in the chamber was evacuated to below 100 Pa. A pressure of 53 MPa was then applied to the sample. The sample is subsequently heated to 400 °C within seconds as the starting temperature. Afterwards, the sample was heated linearly with 45 °C min^−1^ until inflection point on the displacement curve observed. Upon detecting the inflection point on the sample displacement curve, the program was promptly stopped, and the pressure on the sample was released. The circulating cooling water rapidly cools the components in contact with the graphite mold, thereby allowing the sample to cool down quickly, reaching a temperature below 100 °C within 20 min.

### Material characterization

Relative density was measured by the Archimedean method. Quantitative determination of elements was analyzed using inductively coupled plasma-optical emission (ICP-OES) spectrometry with a Thermo Scientific iCAP7600 instrument (Thermo Fischer Scientific, Waltham, MA, USA). SEM and SEM-EDS and Backscattered Electron Detector (BSD) images were obtained using a Zeiss EVO 15 or Zeiss Gemini 450 instrument (Carl Zeiss Microscopy Deutschland GmbH, Oberkochen, Germany) at 20 kV coupled with an energy-dispersive X-ray spectroscopy detector Ultim® Max 100 or Ultim® Max 170, respectively (Oxford Instruments plc, Abingdon, UK). For EBSD, Zeiss SUPRA 50VP was utilized. SXRD measurements were conducted at KARA using a wavelength of 0.4592 Å, with powders obtained by grinding the sintered pellets. The resulting data were analyzed utilizing GSAS Ⅱ. XPS was tested employing Axis Ultra spectrometer with Al (Mono) Kα X-ray source, 1486.6 eV. The particle size distribution of mother powder was examined through laser diffraction in ethanol using a LA950 (Horiba Scientific) equipped with 650 and 405 nm laser sources. Prior to particle size measurement, samples underwent 5 min of ultrasonic treatment to ensure deagglomeration. The particle size distribution of sintered pellets was analyzed by ImageJ based on BSD pictures. Samples for STEM analysis were prepared by polishing, dimple grinding, and finally ion milling to electron beam transparency. Ion milling of samples was done with a Gatan PIPS II, with a final milling energy of 0.2 keV. STEM analysis, including high-angle annular dark field (HAADF) imaging and energy-dispersive X-ray spectroscopy (EDS) mapping was done with a Hitachi HF5000 S/TEM. High-resolution HAADF imaging of select samples was done using an FEI Titan G2 80–200 ChemiSTEM. Air exposure between ion milling and sample insertion into vacuum was on the order of several minutes.

### Electrochemical characterization

Impedance, DC polarization, CCD, and symmetrical battery cycling were conducted in a Swagelok cell using a BioLogic VMP-300 multipotentiostat at 25 °C in climate chamber. Impedance was tested with blocking electrodes, achieved by sputtering gold onto the fresh surface. The frequency range spanned from 7 MHz to 1 Hz, in a potentiostatic mode with voltage amplitude of 20 mV. The number of data points was 10 points per frequency decade. Prior to each EIS measurement, the working electrode was allowed to stabilize at the open-circuit potential for 15 min to reach a quasi-stationary condition. The EIS measurement was then performed at the stabilized open-circuit potential. For temperature-dependent impedance measurements, electrochemical systems (Keysight E4991B and Novocontrol Technologies Alpha-A) covering an AC frequency range from 10 MHz to 1 Hz were employed, using an alternating voltage amplitude of 20 mV. Temperature-dependent impedance was recorded between 100 and −100 °C within a temperature-controlled chamber (Novocontrol Technologies BDS1100). Data fitting and DRT analysis were performed using RelaxIS 3 (rhd Instruments). All graphs were normalized to the thickness and area of the samples. The ionic conductivity was calculated using the formula

2 $$\sigma=\frac{L}{{RA}}$$

and the real GB conductivity was calculated using the formula

3 $${\sigma }_{{GB}}=\frac{L}{{R}_{{GB}}A}\left(\frac{{C}_{{bulk}}}{{C}_{{GB}}}\right)$$

where R is the resistance, L is the sample thickness, A is the sample area, and ${C}_{{bulk}}$ and ${C}_{{GB}}$ are fitted capacitances of bulk and GB, respectively. The activation energy ${E}_{a}$ was determined from a linear fit of the Arrhenius plot according to the Arrhenius equation

4 $$\sigma T={\sigma }^{0}{e}^{-\left(\frac{{E}_{a}}{{kT}}\right)}$$

where σ is ionic conductivity, ${\sigma }^{0}$ is the pre-exponential factor, T is absolute temperature, ${E}_{a}$ is the activation energy and k is the Boltzmann constant. DC polarization was tested by applied 0.2 V bias to LLZO pellets and recorded the current. Electronic conductivity was calculated as

5 $$\sigma=\frac{{IA}}{{VL}}$$

where I is the steady current at applied voltage, A is the area of the LLZO pellet, V is the applied voltage, and L is the thickness of the LLZO pellets. The CCD was tested in a symmetric cell assembled within a glovebox. The surface of the LLZO pellets was polished, followed by attaching fresh Li foils on both sides. The fresh Li foils were prepared by removing the oxidized surface layer of the Li ingots (purity > 99.9%, Aladdin), and manually rolling them into thin foils with 0.5 ± 0.1 mm in thickness using glass tools in a glovebox (O_2_ < 0.5 ppm, H_2_O < 0.1 ppm) at temperature of 25 ± 5 °C. The pellets with Li foils were then heated to 300 °C to melt the Li, and subsequently cooled to 25 °C. The full battery performance was tested in a coin cell assembled within a glovebox. Prepared Li metal foils were used as the negative electrode, applied to one side of the LLZO pellet following the method described earlier. The positive electrode consisted of commercial high-voltage LiCoO_2_ (Canrd) (areal density: 10.7 mg cm^−2^; loading: 96.4%; compacted density: 3.5 g cm^−3^). To ensure good contact, a drop (20 ± 5 μL) of liquid electrolyte (1.0 M LiPF_6_ in EC:DMC:EMC = 1:1:1 vol% with 1.0 wt.% VC) was used on the positive electrode side. Assembled batteries were tested at 25 °C using above mentioned equipment. The batteries used for post-mortem analysis were disassembled in a glovebox, and the Li metal on both sides of the LLZO pellets was scraped off. The pellets were then crushed or broken apart along the cracks. Subsequently, samples were prepared inside the glovebox and quickly transferred to the SEM chamber under ambient conditions for further observation.

### Computation

The MLFFs for Al or Ta-doped LLZO were developed based on the neural-network models with the Behler-Parrinello symmetry functions, using n2p2 code^56–58^. We used the same approach for training the models shown in our previous work for undoped LLZO^59^, though only one type of angular symmetry function (so called narrow-angular symmetry function) was used in this work to reduce the model complexity. The training data was generated by collecting atomic structures of Al and Ta-doped LLZO and their energies and atomic forces from first-principles simulations by density functional theory (DFT) and ab-initio molecular dynamics (AIMD). (See below for details of first-principles simulations). The total number of data is 17,086 and 17,546 for Al and Ta-doped LLZO, respectively, which are cubic and amorphous LLZO structures at 300–6000 K, using various doping levels with* x* = 0, 0.25, 0.50, and 0.75 in [Li_7-3x_Al_x_]La_3_Zr_2_O_12_ and Li_7-x_La_3_[Zr_2-x_Ta_x_]O_12_. The charge states of dopants were assumed as Al^3+^ (replacing three Li^+^) and Ta^5+^ (replacing one Zr^4+^ and one Li^+^ removal). Note that amorphous LLZO models were adopted from our previous work^60^, which were generated using the melting-quenching approach. The models were validated by benchmarking energies and forces against DFT calculations for atomic structures sampled every 1 ps from MD simulations at 2000 K for 100 ps (Supplementary Fig. 10). Supplementary Table 9 summarizes the RMSEs for energies and forces for Al and Ta-doped cubic and amorphous LLZO with four doping levels *x* = 0, 0.25, 0.50, and 0.75, showing that the energy RMSEs are at most 6.1 meV/atom with *R*^2^ values higher than 0.99 (*R*^2^ measures the similarity between prediction and observation^61^; see ref. ^59^ for the evaluation method). The force RMSEs are also low in a range of 0.11–0.15 eV/Å for cubic systems and 0.15–0.18 eV/Å for amorphous systems, with *R*^2^ values higher than 0.98. The energy and force validation tests show that the developed ML potentials can predict energies and forces close to DFT calculations.

DFT calculations were performed using the Vienna ab-initio Simulation Package (VASP)^62^. Exchange-correlation effects were treated within the generalized gradient approximation (GGA), as parameterized by Perdew, Burke, and Ernzerhof (PBE)^63^. Interactions between core and valence electrons were treated using the projector augmented wave (PAW) method^64,65^. A plane-wave basis set with cutoff energy of 500 eV was used for the plane wave basis and a Γ-centered single k-point sampling was used. The energy of the electronic ground state was converged to 10^−5^ eV, and the force criterion for atomic relaxations was set to 0.01 eV/Å. Ab-initio molecular dynamics (AIMD) simulations were performed using the NVT ensemble with the Nosé–Hoover thermostat with a 0.5 fs time step. The diffusivities of La from AIMD were estimated using trajectories longer than 30 ps at 1200, 1400, 1600, 1800, and 2000 K. Large-scale MD simulations with MLFFs were performed using *LAMMPS* software^66^, also with the Nosé–Hoover style thermostat and barostat for NVT and NPT simulations, respectively, with a time step of 0.5 fs for the bulk LLZO systems and 1 fs for GB models. The GB models were constructed following the same methods as shown in our previous works^14,45^, which were equilibrated at 1300 K under the NPT ensemble for 2.5 ns followed by the full degree of freedom relaxations. The diffusivities of Li were evaluated using 100 ps MLFF-MD trajectories at 700, 900, and 1100 K and those of dopants were calculated using trajectories longer than 2.7 ns at 1200, 1400, 1600, and 1800 K, using the NVT ensemble. Small GB models for DFT calculations, i.e., Σ5 (120) [001] (960 atoms), Σ3 (1-11) [011] (1152 atoms), and Σ3 (1-21) [111] (1152 atoms), used for calculating electronic density of states, were modeled using the same procedure as for the large GB models. A (100)-oriented surface slab model, based on the conventional unit cell with Li-terminated surface compositions, was used to calculate the surface segregation behavior of Ta using DFT.

The ion diffusivities were calculated using NVT MD trajectories by the mean squared displacement as

6 $$D=\frac{1}{6{tN}}{\sum }_{j}^{N}{\left|{{{{\bf{r}}}}}_{j}\left(t+{t}_{0}\right)-{{{{\bf{r}}}}}_{j}\left({t}_{0}\right)\right|}^{2}$$

where N is number of atoms, ${{{{\bf{r}}}}}_{j}$ is the position of atom j, and ${t}_{0}$ is the initial (reference) time. Then, the activation energy ${E}_{a}$ was estimated by fitting the diffusivity–temperature data to the Arrhenius relation as

7 $$D\left(T\right)={D}_{0}\exp \left[-\frac{{E}_{a}}{{k}_{{{{\rm{B}}}}}T}\right]$$

where ${D}_{0}$ is a prefactor, ${k}_{{{{\rm{B}}}}}$ is the Boltzmann constant, and T is temperature. The transport of Al and Ta at GBs was studied by placing them only at the GB areas; about 3.6% of Li were replaced by Al with two additional Li removal per Al for charge balance, and about 50% of Zr were replaced by Ta with one Li removal per Ta, based on the experimental observation.

The segregation energies of Al and Ta with large GB models and MLFFs were calculated by

8 $${E}_{{{{\rm{seg}}}}}=\frac{\left({E}_{d{{{\rm{oping}}}},{{{\rm{GB\; area}}}}}-{E}_{{{{\rm{doping}}}},{{{\rm{bulk\; area}}}}}\right)}{{N}_{{{{\rm{dopant}}}}}}$$

where ${E}_{{{{\rm{doping}}}}}$ is the total energy of the GB model with dopants at either GB areas or bulk areas and ${N}_{{{{\rm{dopant}}}}}$ is the number of dopants in the GB model. We used 50 randomly-doped GB models to obtain the averaged ${E}_{d{{{\rm{oping}}}},{{{\rm{GB\; area}}}}}$ and ${E}_{{{{\rm{doping}}}},{{{\rm{bulk\; area}}}}}$ values for statistical reliability. The defect formation energies of Al, Ta, and La in cubic and amorphous LLZO by DFT were calculated by

9 $${E}_{{{{\rm{form}}}}}={E}_{{{{\rm{defect}}}}}-\left({E}_{{{{\rm{pureLLZO}}}}}+{\sum}_{i}{n}_{i}{\mu }_{i}\right)$$

where ${E}_{{{{\rm{defect}}}}}$ and ${E}_{{{{\rm{pureLLZO}}}}}$ are the total energies of the defected and pristine LLZO structures, respectively, and ${n}_{i}$ and ${\mu }_{i}$ are the number of elements *i* removed or added in the system and ${\mu }_{i}$ is the chemical potential of the element *i* using elemental metal system as its reference. In total, 10 randomly doped structures, which are defected structures with one Al replacing three Li or one Ta replacing one Zr and one Li, were used to get the averaged for ${E}_{{{{\rm{defect}}}}}$ for statistics. Note that Al was places at 24 d Li site. The average formation energy of La vacancy was calculated by considering all individual 24 La ions in cubic and amorphous LLZO models. The Frenkel defect formation energies for Ta and La were calculated by comparing total energies between systems before and after swapping site occupancies between the dopant and Li. Both the tetrahedral and octahedral Li sites were tested. Note that a Li-ion occupies Ta/La vacancy to lower the total energy of the system (i.e., Li–Ta and Li–La site exchange for the Frenkel defect formation).

The radial distribution functions (RDFs) ${g}_{\alpha \beta }\left(r\right)$ for species α and β as a function of distance *r* were calculated by

10 $${g}_{\alpha \beta }\left(r\right)=\frac{1}{{\rho }_{\beta }{N}_{\alpha }}\langle {\sum }_{j}^{{N}_{\alpha }}{\sum }_{k\ne j}^{{N}_{\beta }}\delta \left(r-{r}_{{jk}}\right)\rangle$$

where ${\rho }_{\beta }$ is the number density of species β, ${N}_{\alpha {{{\rm{or}}}}\beta }$ is the number of species α or β, and δ is the Dirac delta function with the distance ${r}_{{jk}}$ between atom j of species α and atom k of species β. The coordination number (CN) can be calculated by

11 $${{CN}}_{\alpha \beta }\left(r\right)=4\pi {\rho }_{\beta }{\int }_{o}^{r}{r}^{{\prime} 2}{g}_{\alpha \beta }\left({r}^{{\prime} }\right)d{r}^{{\prime} }$$

The positional probability density was evaluated by subdividing the simulation cell into a grid of 0.25 × 0.25 × 0.25 Å^3^ cubes. Position densities for a given grid were determined from the number of time steps for which each mesh was occupied by an atom (normalized by the total counts and divided by the grid volume) using MD trajectories.

Continuum simulation framework was employed to model the SCLs using the Poisson equation and a diffusion-drift equation:

12 $$\varDelta \varPhi=-{qc}/{\varepsilon }_{0}{\varepsilon }_{r}$$

13 $${dc}/{dt}=\nabla \left(D\nabla c+\sigma \left(c\right)\left(\nabla \varPhi+\nabla {\mu }_{0}/F\right)\right)$$

with Φ the electric potential, ${\varepsilon }_{0}$ the vacuum permittivity, ${\varepsilon }_{r}$ the relative permittivity, ${\mu }_{0}$ the chemical reference potential and F the Faraday constant. The resistance of space charges comes from the concentration dependence of σ, inhibiting both high and low concentration transport because the site-to-site jump probability is lowered. Note that D is independent of the concentration in our simplified model of non-interacting particles and no energy difference between octahedral and tetrahedral Li sites and depends on the conductivity in a generalized Nernst-Einstein equation. For simplicity, we assume that the GB core would be charge neutral apart from Li-ion displacements, i.e., there are no trapped ions. The chemical reference potential may be higher in the GB compared to the rest of the grain, both due to higher local site energies and different electronic Fermi energy levels that are compensated by Li-ion charges. The parameters used can be found in Supplementary Table 7. Note that the GB width has a negligible influence on GB resistance, and the main factor remains the influence of the space charge accumulation. We used a maximum Li concentration of 7.5 per unit cell and a site-to-site distance of 0.2 nm^50^, which approximately corresponds to the average distance between tetrahedral and octahedral sites. Note that this distance is not an exact value because we simplify the garnet lattice by using a 1D model. We included the influence of different activation energies in GB and bulk by multiplication at the end with the assumption that in the narrow region of greatest space charge resistance, and the changes in activation energy are negligible.

## Supplementary information

Supplementary Information

Description of Additional Supplementary Files

Supplementary Data 1-3

Transparent Peer Review file

## Source data

Source data

## Data Availability

The data that support the findings of this study are available from the corresponding author upon request. Dataset for training MLFFs and atomic configurations for atomistic modeling and simulations can be found in the Zenodo repository: Kim, K., Wan, L., & Wood, B. Elimination of Detrimental Grain Boundary Segregation in Garnets, *Zenodo*, 10.5281/zenodo.20618849 (2026)^67^. Source data are provided with this paper.

## Code availability

Results described in this manuscript have been obtained with publicly available software packages such as VASP, Lammps (https://docs.lammps.org), and n2p2 (https://compphysvienna.github.io/n2p2/).
