# Supplementary material for: Elimination of detrimental grain boundary segregation in garnets
Source: Nat Commun. 2026 Jun 29;17:8667. doi: 10.1038/s41467-026-74887-z (PMC13490533; doi:10.1038/s41467-026-74887-z)
Supplement: Supplementary file 1 [file 41467_2026_74887_MOESM1_ESM.pdf]

**Supplementary Information**

# Elimination of Detrimental Grain Boundary Segregation in Garnets

Kai Yao[1,2], Kwangnam Kim[3]*, Dylan Jennings[4,5], Jan Dippell[6,7], Lei Jin[4], Meng Ma[8], Xingyu Liu[9], Qianli Ma[1], Walter Sebastian Scheld[1], Christoph Roitzheim[1], Yuan Zeng[1], Timo Danner[6,7], Olivier Guillon[1,10,11], Mark Huijben[12], Johan E. ten Elshof[12], Liwen F. Wan[3], Arnulf Latz[6,7,13], Brandon C. Wood[3], Martin Finsterbusch[1,10] and Dina Fattakhova-Rohlfing[1,2,10,11]*

Corresponding authors*: Kwangnam Kim (kim109@llnl.gov), Dina Fattakhova-Rohlfing (d.fattakhova-rohlfing@fz-juelich.de)

1. Forschungszentrum Jülich GmbH, Institute of Energy Materials and Devices: Materials Synthesis and Processing (IMD-2), Wilhelm-Johnen-Straße, 52428 Jülich, Germany

2. University of Duisburg-Essen, Faculty of Engineering and Center for Nanointegration Duisburg-Essen CENIDE, Lotharstraße 1, 47057 Duisburg, Germany

3. Laboratory for Energy Applications for the Future, Lawrence Livermore National Laboratory 7000 East Ave, Livermore, CA 94550, USA

4. Forschungszentrum Jülich GmbH, Ernst-Ruska Centre for Microscopy and Spectroscopy with Electrons (ER-C), Wilhelm-Johnen-Straße, 52428 Jülich, Germany

5. Faculty of Physics and Astronomy, Advanced Transmission Electron Microscopy, Ruhr University Bochum, Bochum, 44801, Germany

6. German Aerospace Center, Institute of Engineering Thermodynamics, Pfaffenwaldring 38-40, Stuttgart, 70569, Germany

7. Helmholtz Institute Ulm for Electrochemical Energy Storage, Helmholtzstraße 11, Ulm, 89081, Germany

8. State Key Laboratory of Flexible Electronics (LOFE), Institute of Flexible Electronics, Northwestern Polytechnical University, Xi'an 710072, PR China

9. Forschungszentrum Jülich GmbH, Institute of Energy Materials and Devices: Structure and Function of Materials (IMD-1), Wilhelm-Johnen-Straße, 52428 Jülich, Germany

10. Helmholtz Institute Münster: Ionics in Energy Storage (IMD-4), Forschungszentrum Jülich GmbH, Corrensstr. 46, 48149 Münster, Germany

11. Jülich-Aachen Research Alliance: JARA-Energy, 52425, Jülich, Germany

12. MESA+ Institute for Nanotechnology, University of Twente, PO Box 217, 7500 AE

Enschede, the Netherlands

13. Ulm University, Institute of Electrochemistry, Albert-Einstein-Allee 47, Ulm, 89081, Germany

**Supplementary Fig. 1 | Conventional sintering of LLZO. a**, Relative densities and Li concentrations of LLZO as a function of sintering time. Density and Li concentration with error bars are presented as mean values of 5 separately sintered pellets +/- SD. **b**, SEM-EDS images of the cross-section of S-LLZO. The scale bars are 5 μm. The elemental distributions of Ta, Al and La exhibit local inhomogeneities in the images.

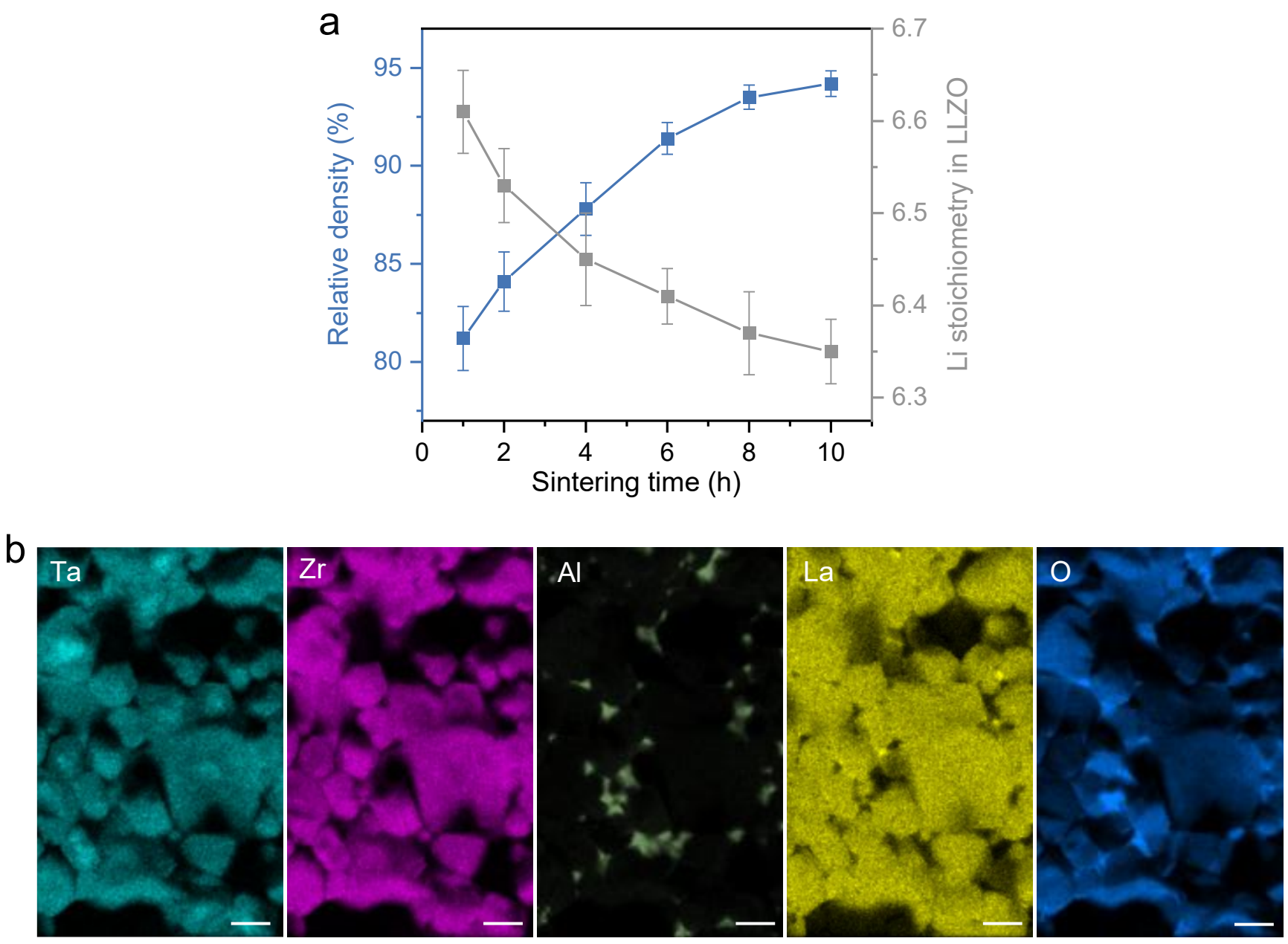

**Supplementary Fig. 2 | MLFF-MD for Ta, Al and La migration. a**, All GBs for simulations. All GB models were constructed based on the [001], [011], and [111] orientations. For charge balance, each Ta substitution for Zr compensated by removing one Li, and each Al substitution for Li compensated by removing two Li atoms. The GB models were equilibrated at 1100 K using NPT for 10 ns, followed by relaxation using the MLFF. Green, yellow, cyan, purple, and blue spheres represent Li, La, Ta, Zr, and O, respectively. **b**, Formation energy calculations of Ta and La at Li 24d and 96h sites. For Ta and La substitution, closest Li are removed to account for charge compensation. Note that a Li ion occupies Ta/La vacancy to lower the total energy of the system (i.e., Li-Ta and Li-La site exchange for the Frenkel defect formation). **c**, MSD of Al, Ta and La. MLFF-MD simulations were conducted on bulk LLZO and three GBs, with doping ratios set at approximately 0.5 for Al and Ta per formula unit. For each result, the MSD was averaged across 10 randomly generated doping configurations. **d**, Positional probability density of Al diffusion in $Li_{5.5}Al_{0.5}La_3Zr_2O_{12}$, Ta diffusion in $Li_{6.5}La_3Zr_{1.5}Ta_{0.5}O_{12}$ and La diffusion in $Li_{6.25}La_{3.25}Zr_2O_{12}$ at 1800 K. Their isosurfaces level are $1*10^{-5}$. **e**, MSD and Arrhenius plot of La from AIMD simulations. Each data point with error bar is presented as mean values of 10 independent AIMD simulations +/- SD.

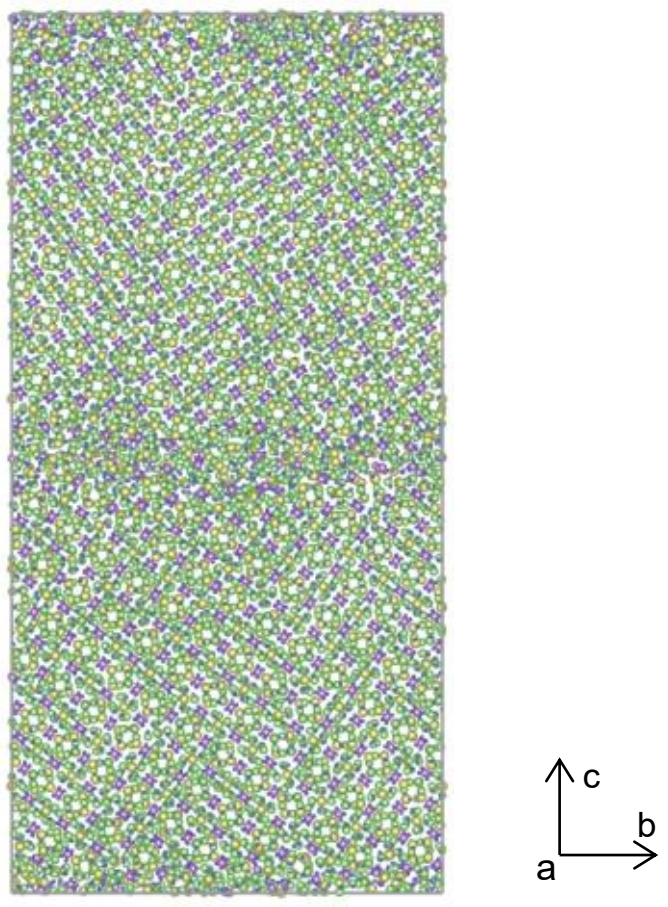

Σ25 (340) [001] tilt angle16.26deg

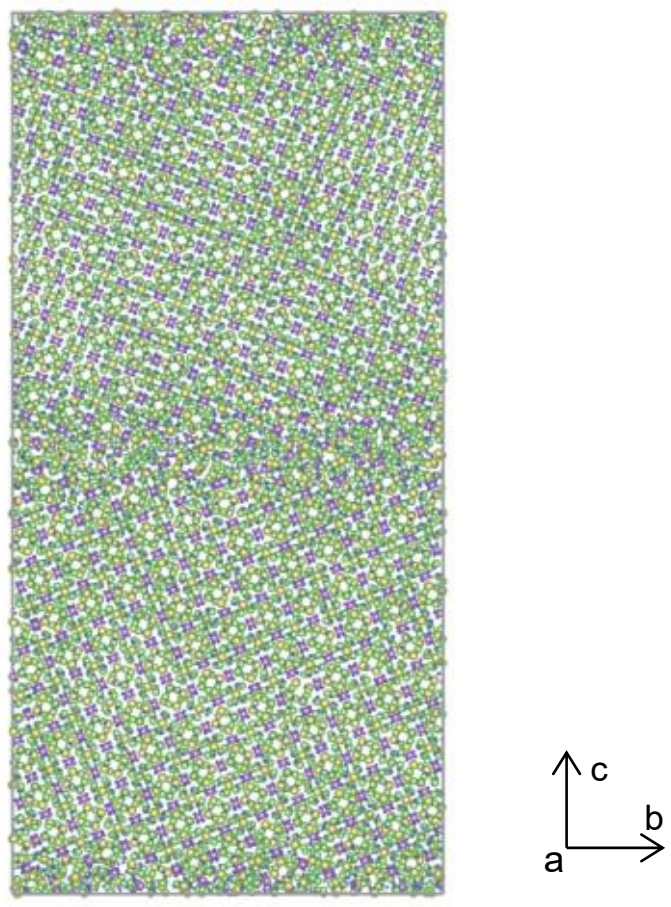

Σ29 (-520) [001] tilt angle46.4deg

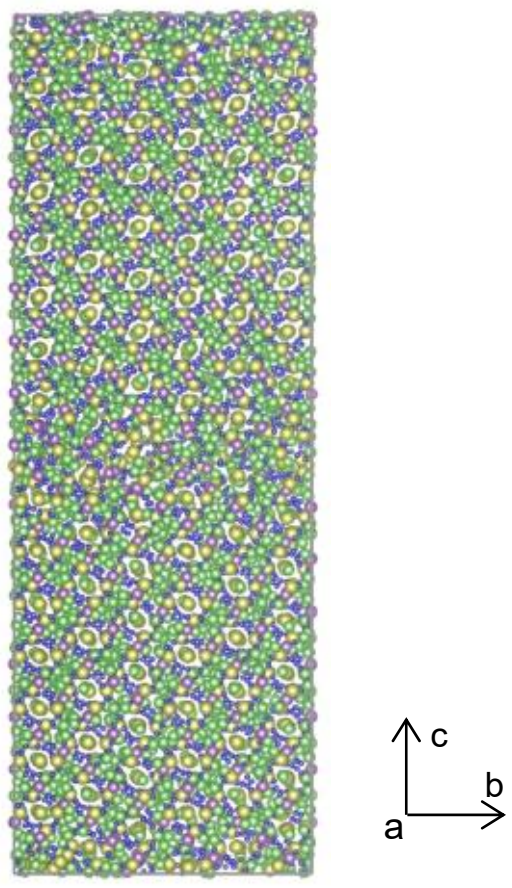

Σ03 (1-11) [011]tilt angle70.53deg

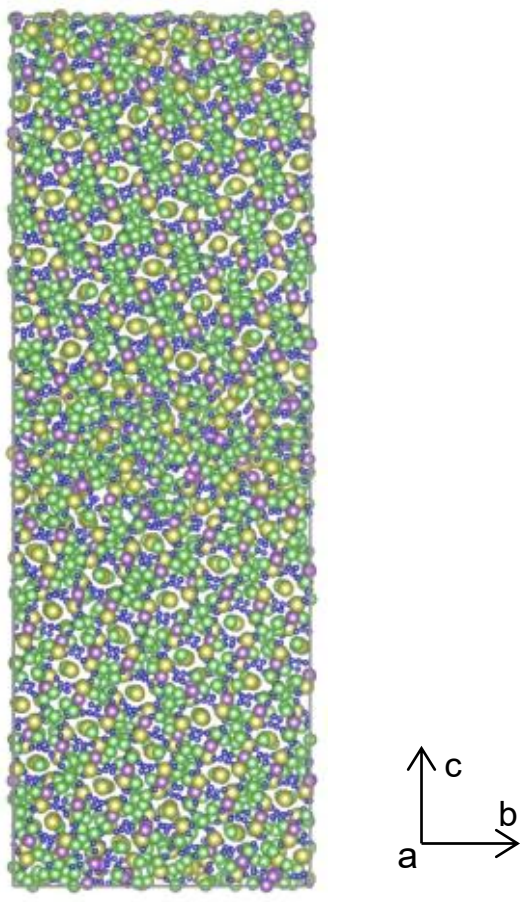

Σ09 (1-22) [011] tilt angle38.94deg

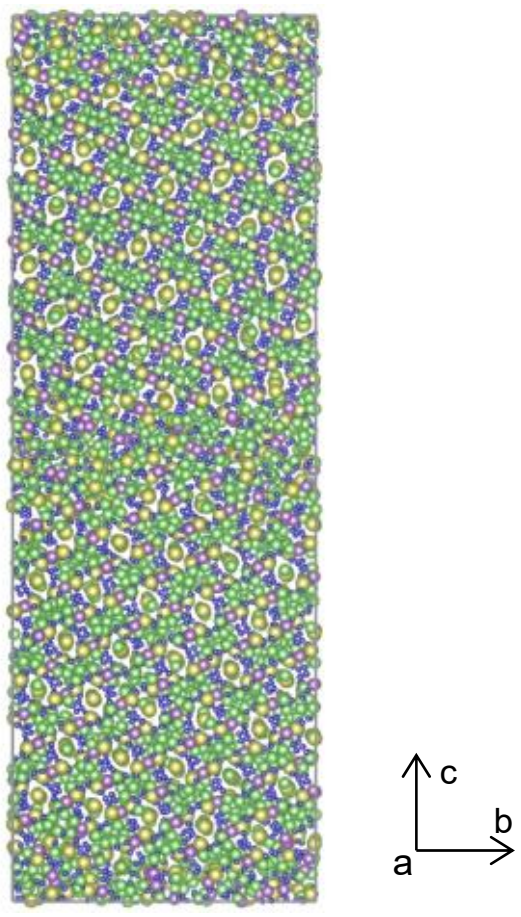

Σ11 (31-1) [011] tilt angle50.48deg

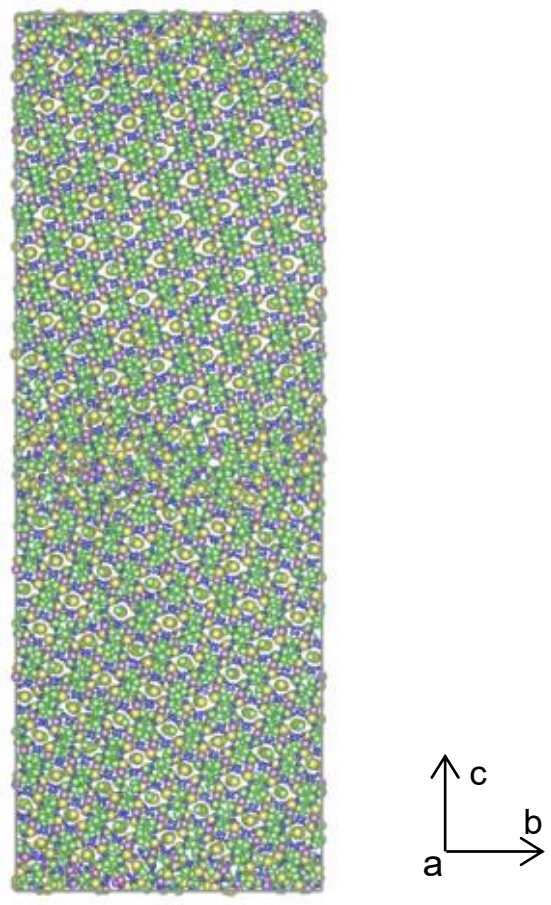

Σ19 (1-33) [011] tilt angle26.53deg

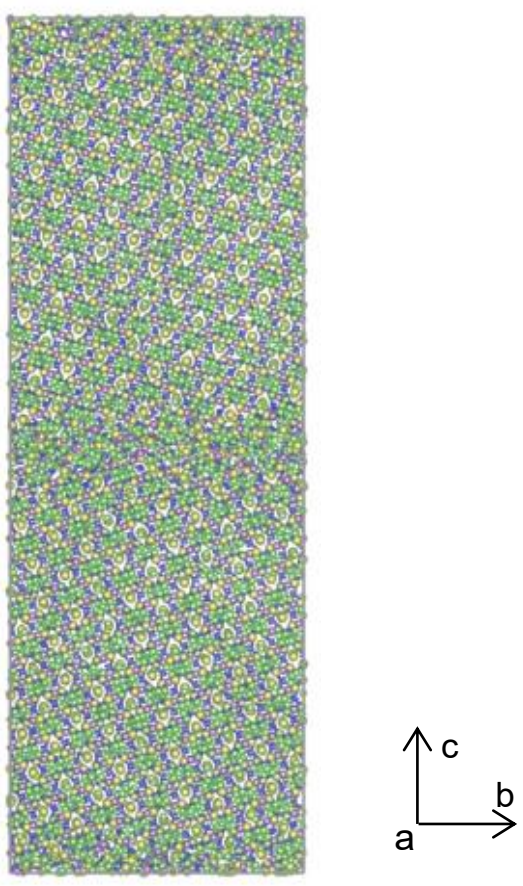

Σ27 (51-1) [011] tilt angle31.59deg

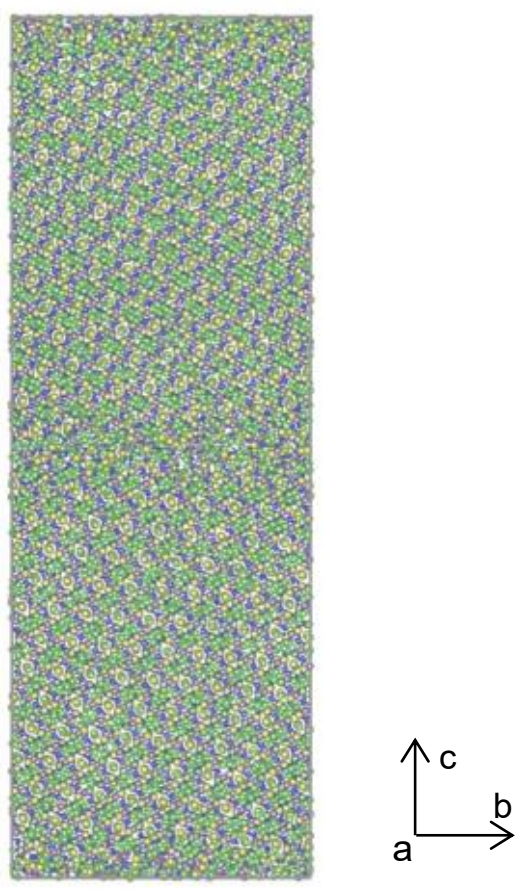

Σ33 (52-2) [011] tilt angle58.99deg

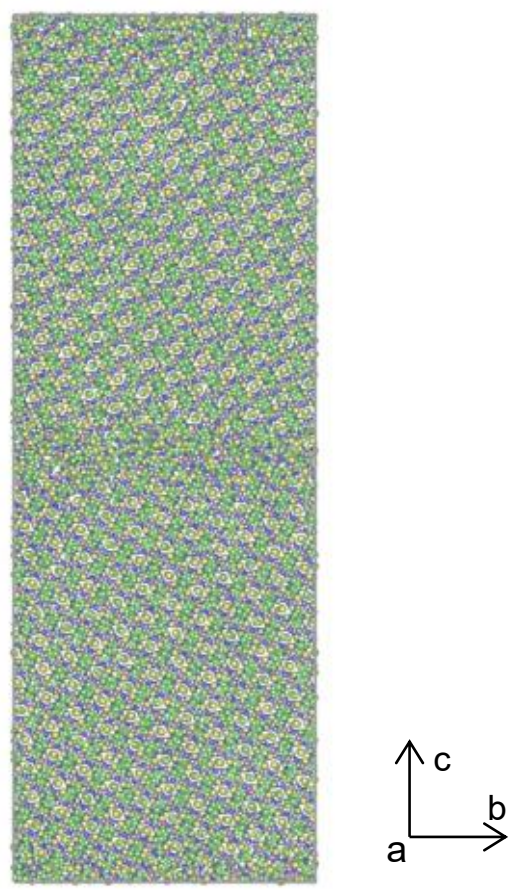

Σ41 (3-44) [011] tilt angle55.88deg

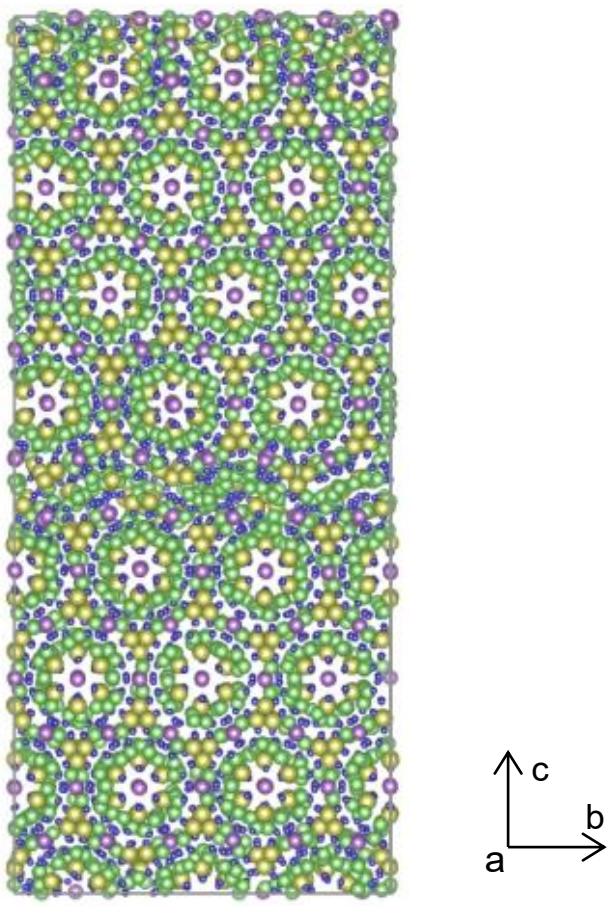


Σ03 (-101) [111] tilt angle60.0deg

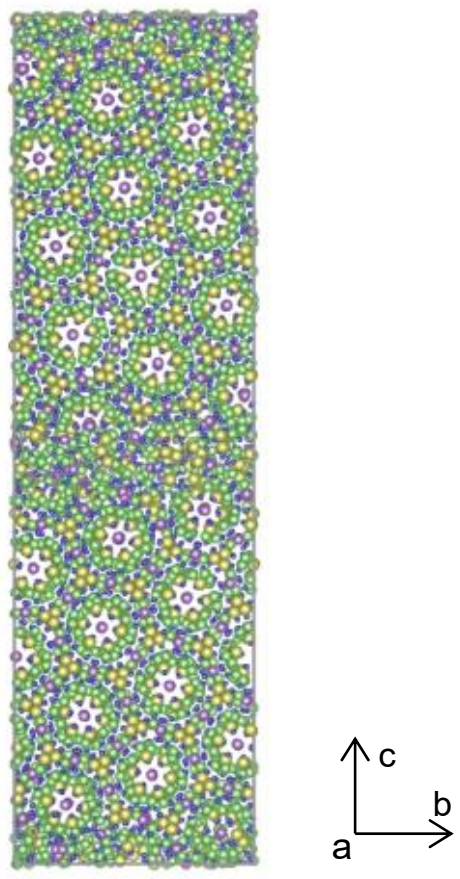


Σ07 (21-3) [111] tilt angle38.21deg

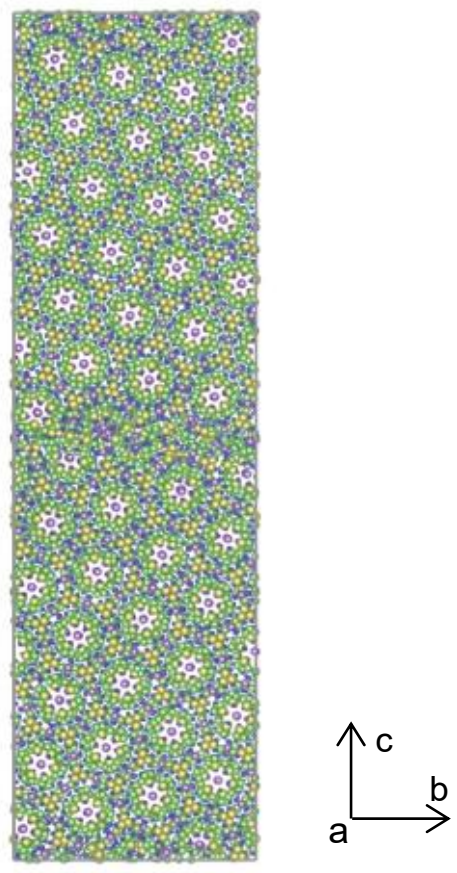


Σ13 (1-43) [111] tilt angle27.8deg

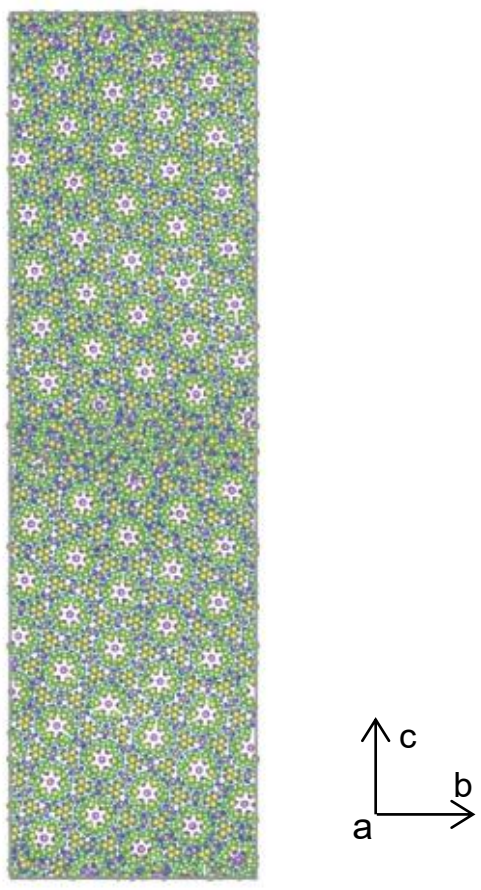


Σ19 (32-5) [111] tilt angle46.83deg

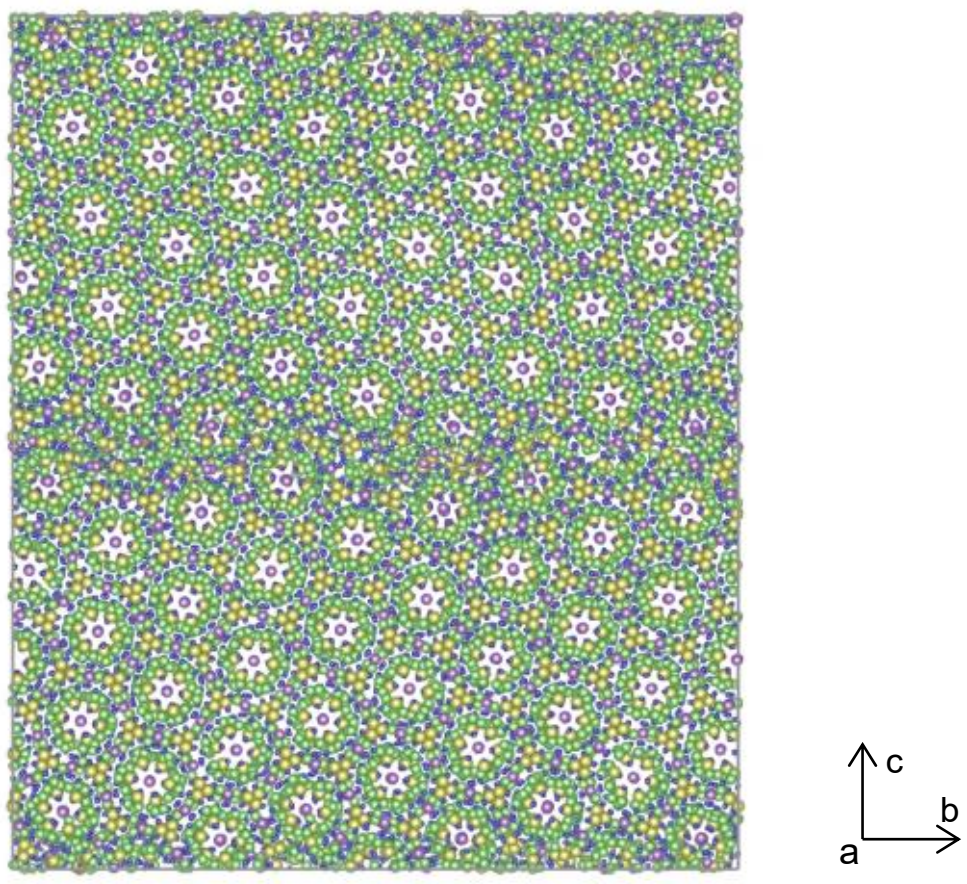


Σ21 (2-31) [111] tilt angle21.79deg

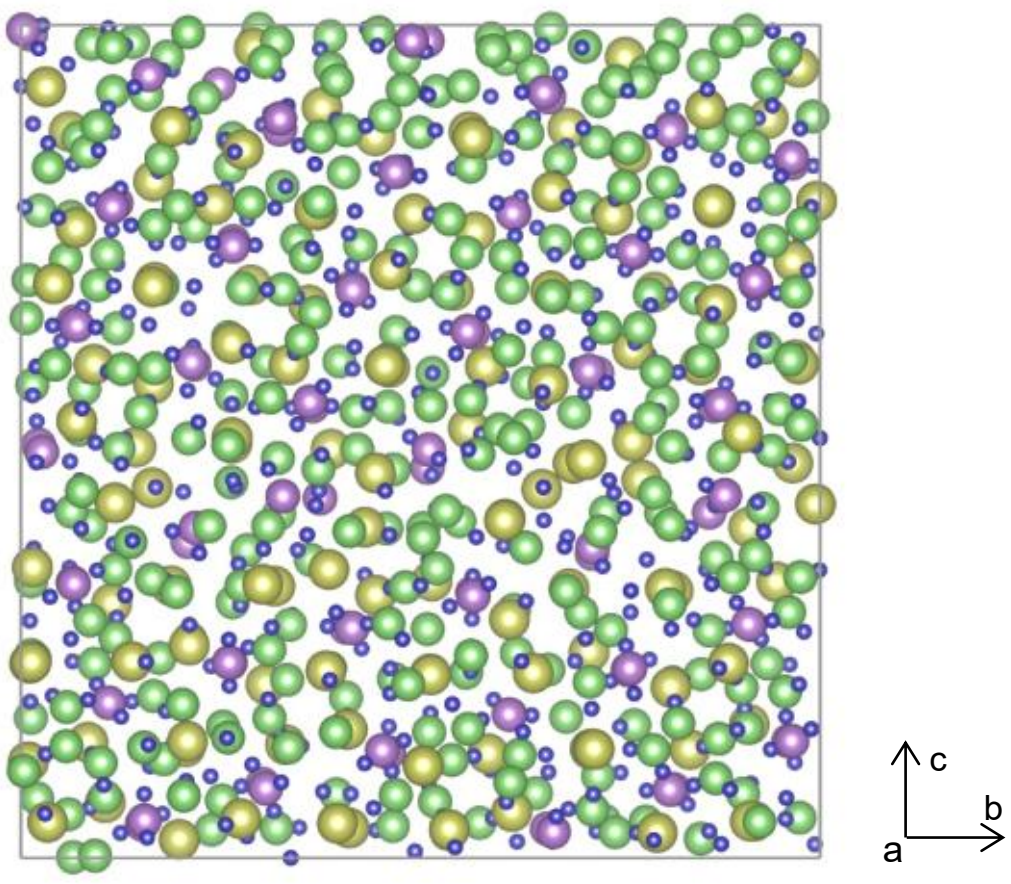


Σ5 (120) [001] tilt angle 36.87deg

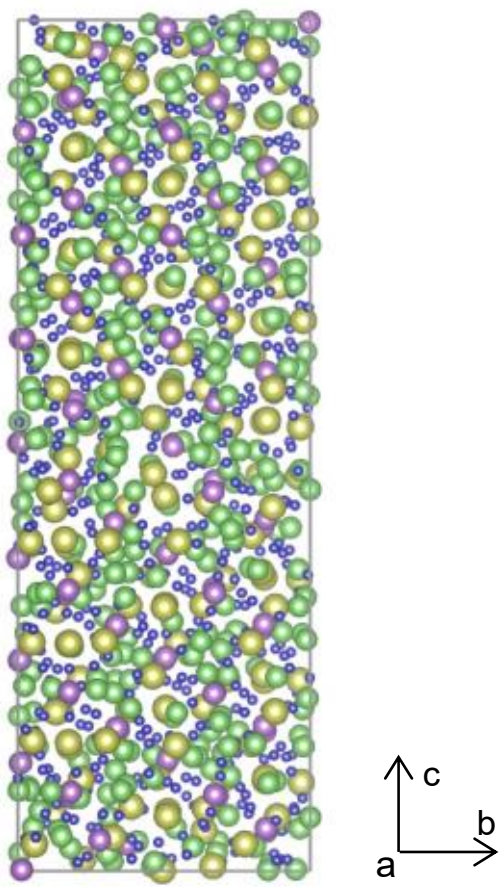


Σ3 (1-11) [011] tilt angle 70.53deg

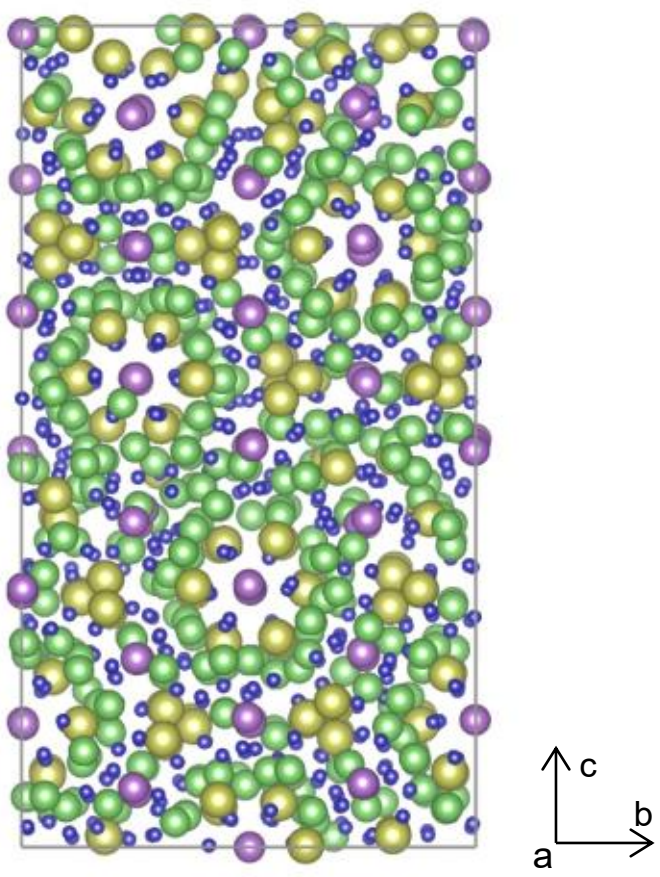


Σ3 (1-21) [111] tilt angle 60.00deg

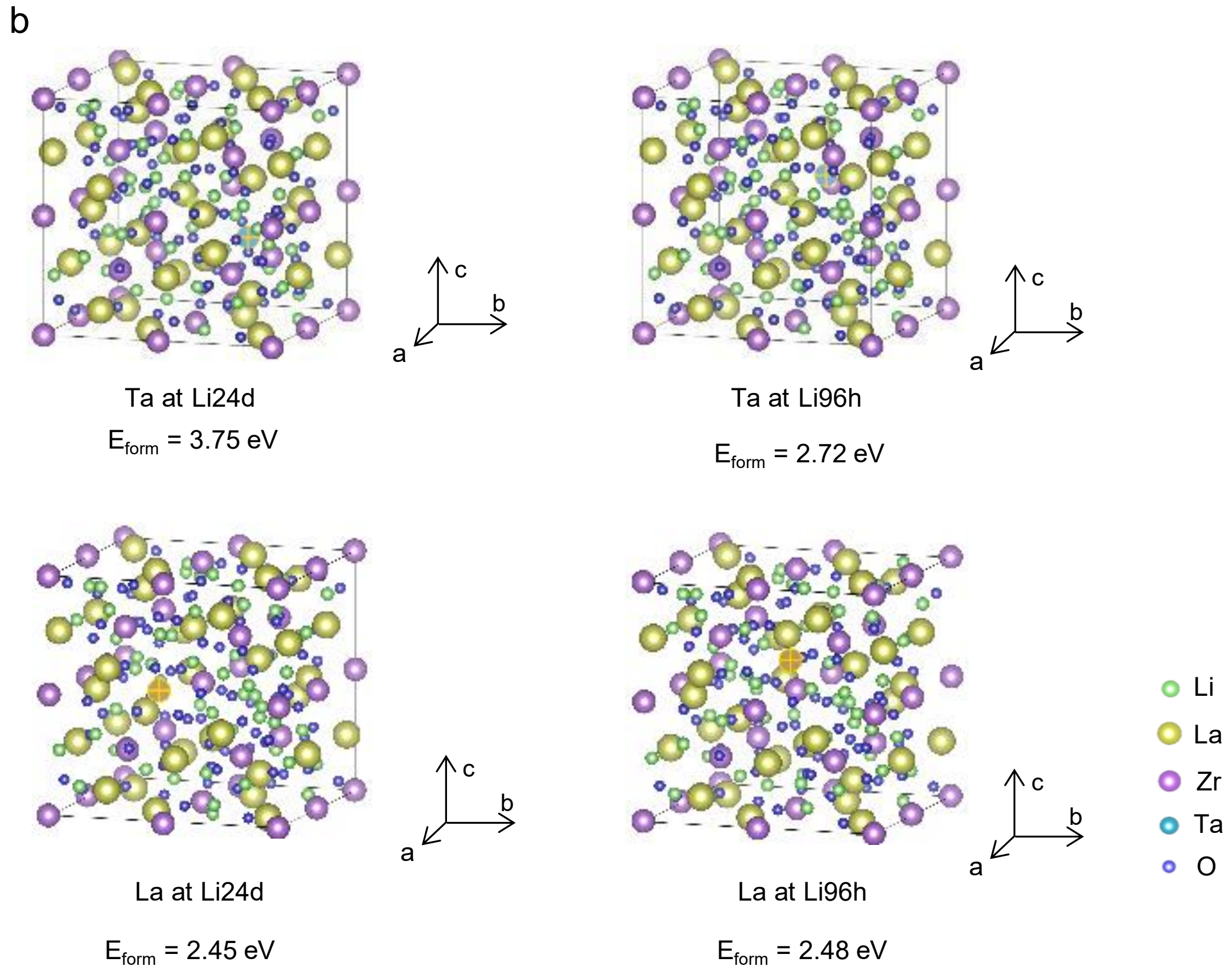
b
c
b
a
Ta at Li24d
E_form = 3.75 eV
Ta at Li96h
E_form = 2.72 eV
La at Li24d
E_form = 2.45 eV
La at Li96h
E_form = 2.48 eV
Li
La
Zr
Ta
O

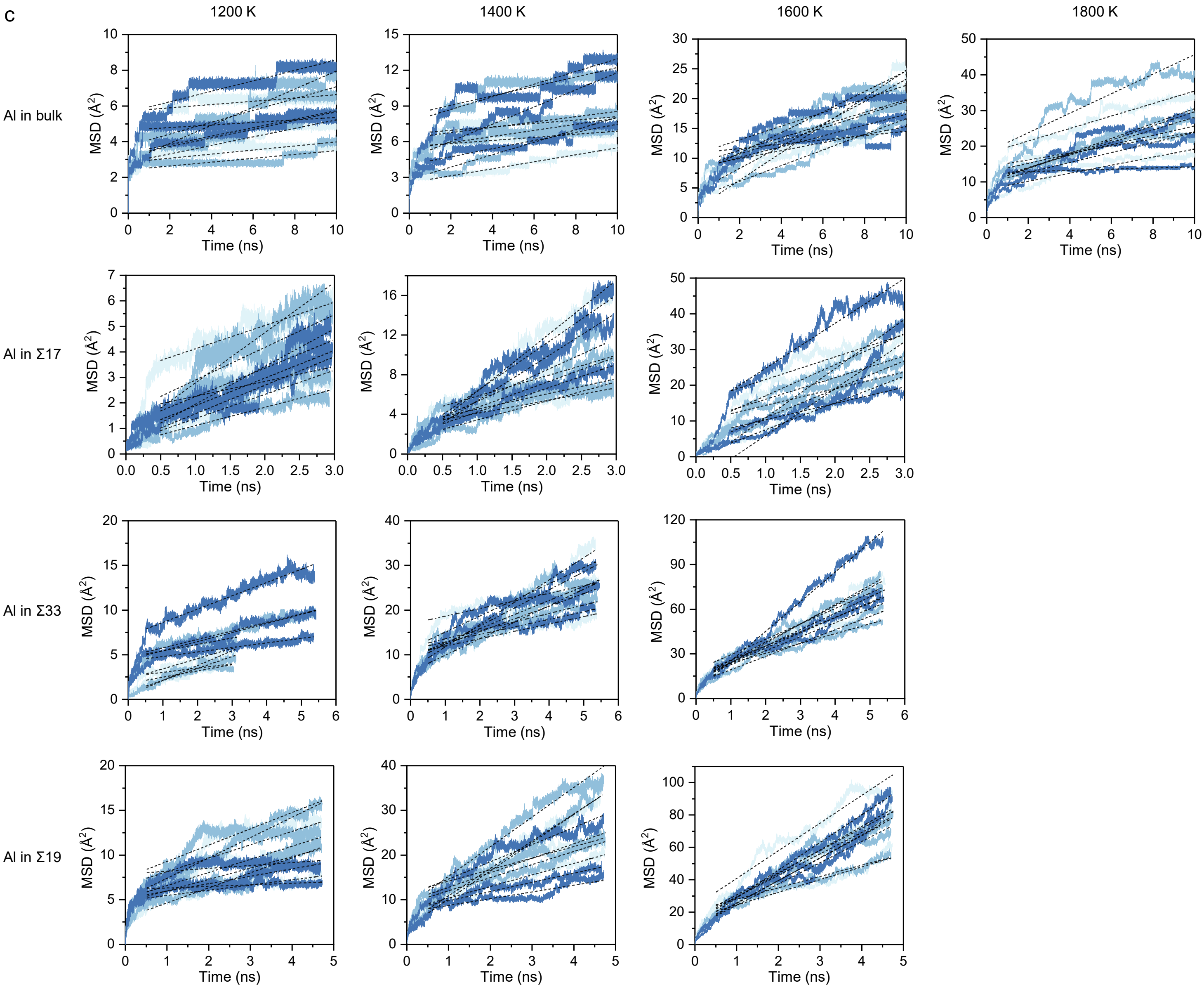
c
1200 K
1400 K
1600 K
1800 K
Al in bulk
Al in Σ17
Al in Σ33
Al in Σ19
MSD (Å2)
Time (ns)

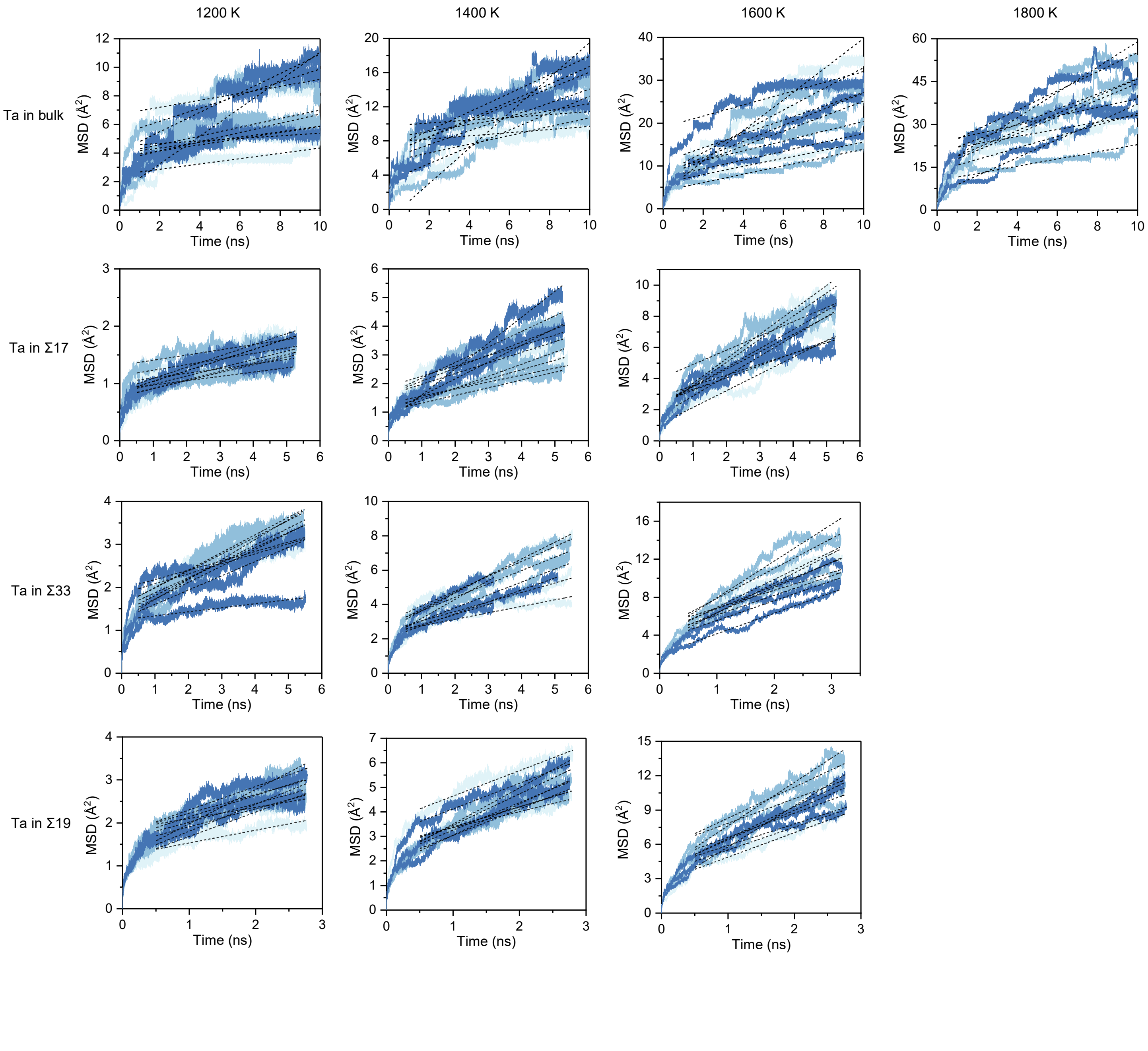
1200 K
1400 K
1600 K
1800 K
Ta in bulk
Ta in Σ17
Ta in Σ33
Ta in Σ19
MSD (Å²)
Time (ns)

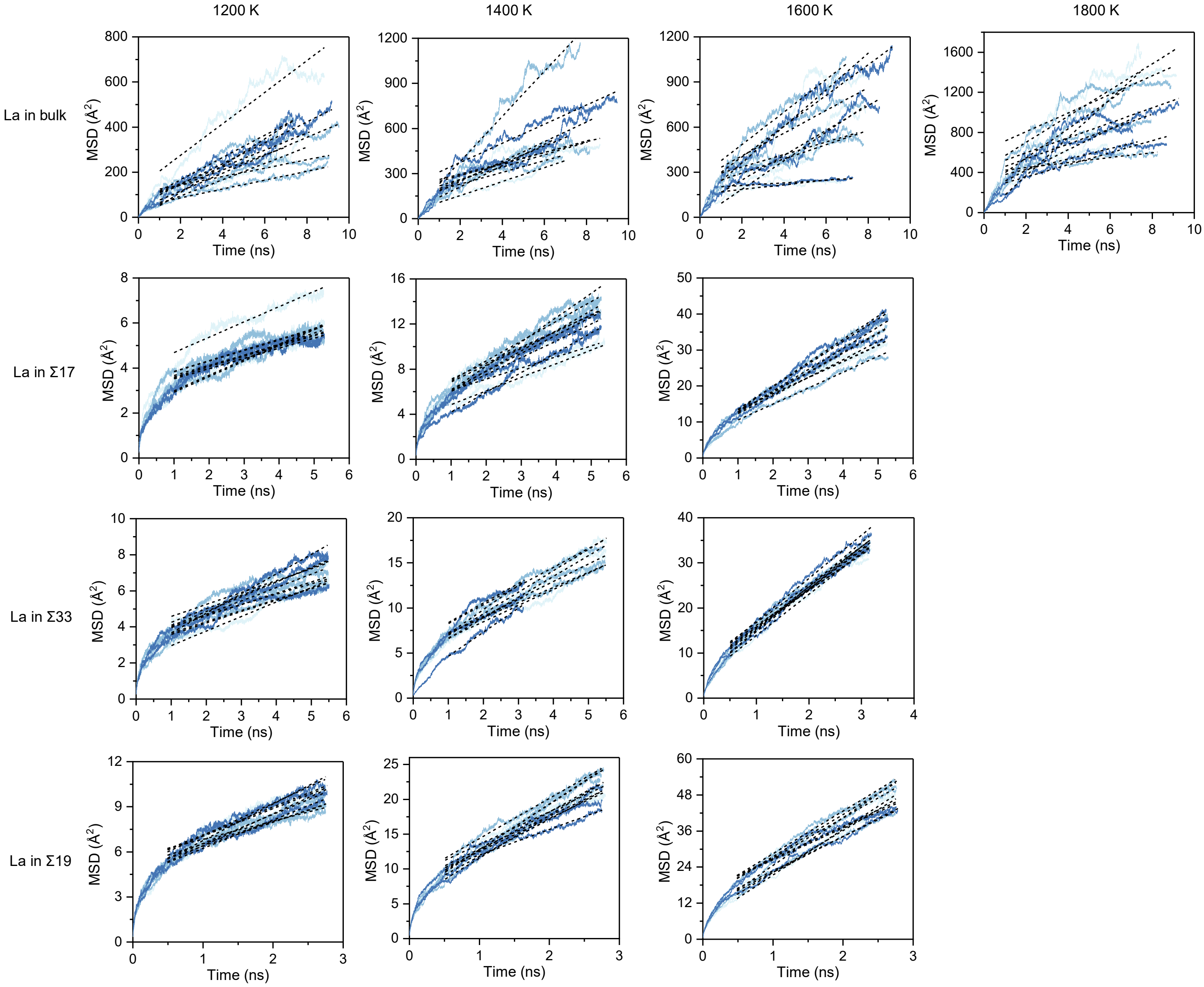

1200 K
1400 K
1600 K
1800 K
La in bulk
La in Σ17
La in Σ33
La in Σ19
MSD (Å²)
Time (ns)

d

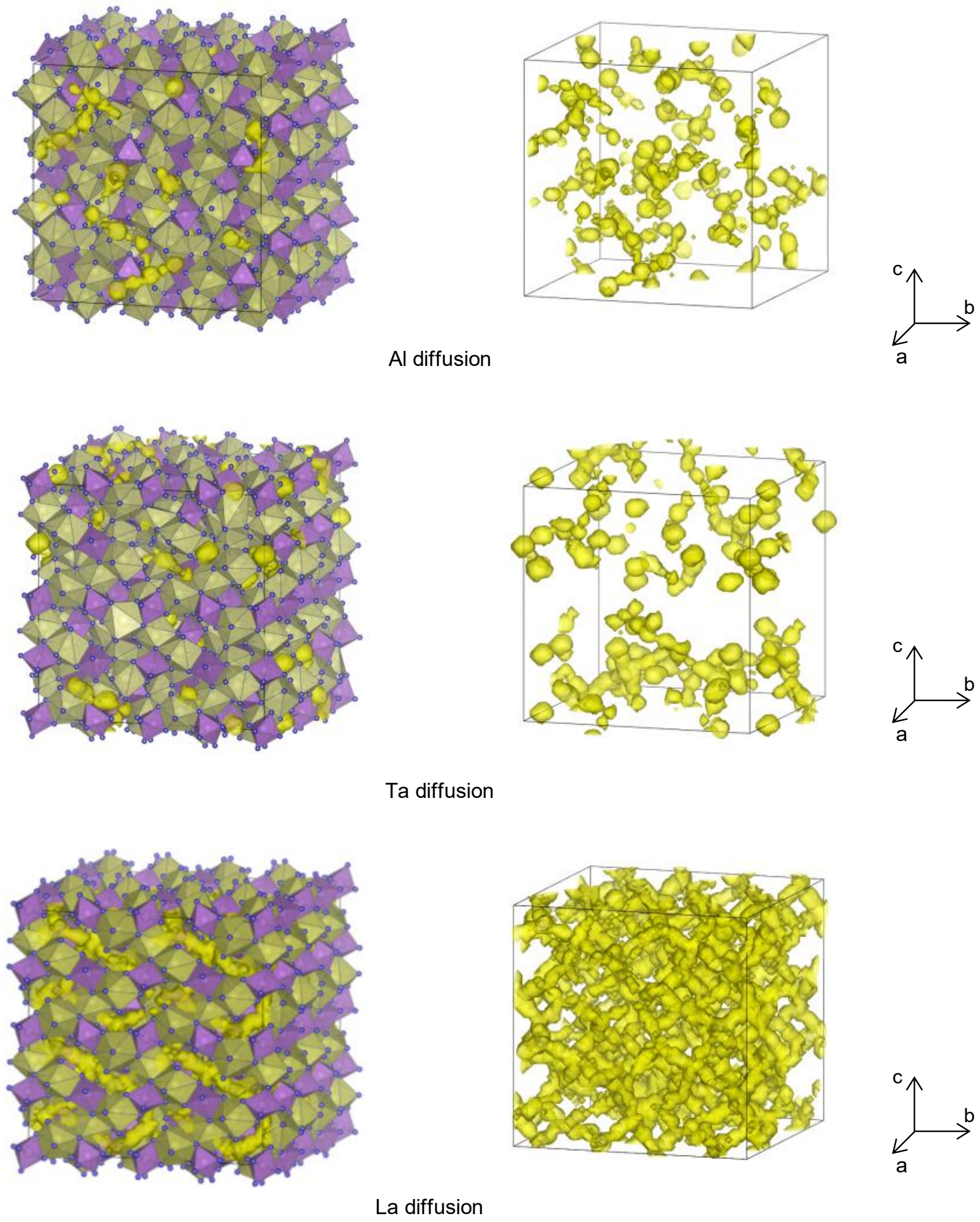
c
b
a
Al diffusion
Ta diffusion
La diffusion

e

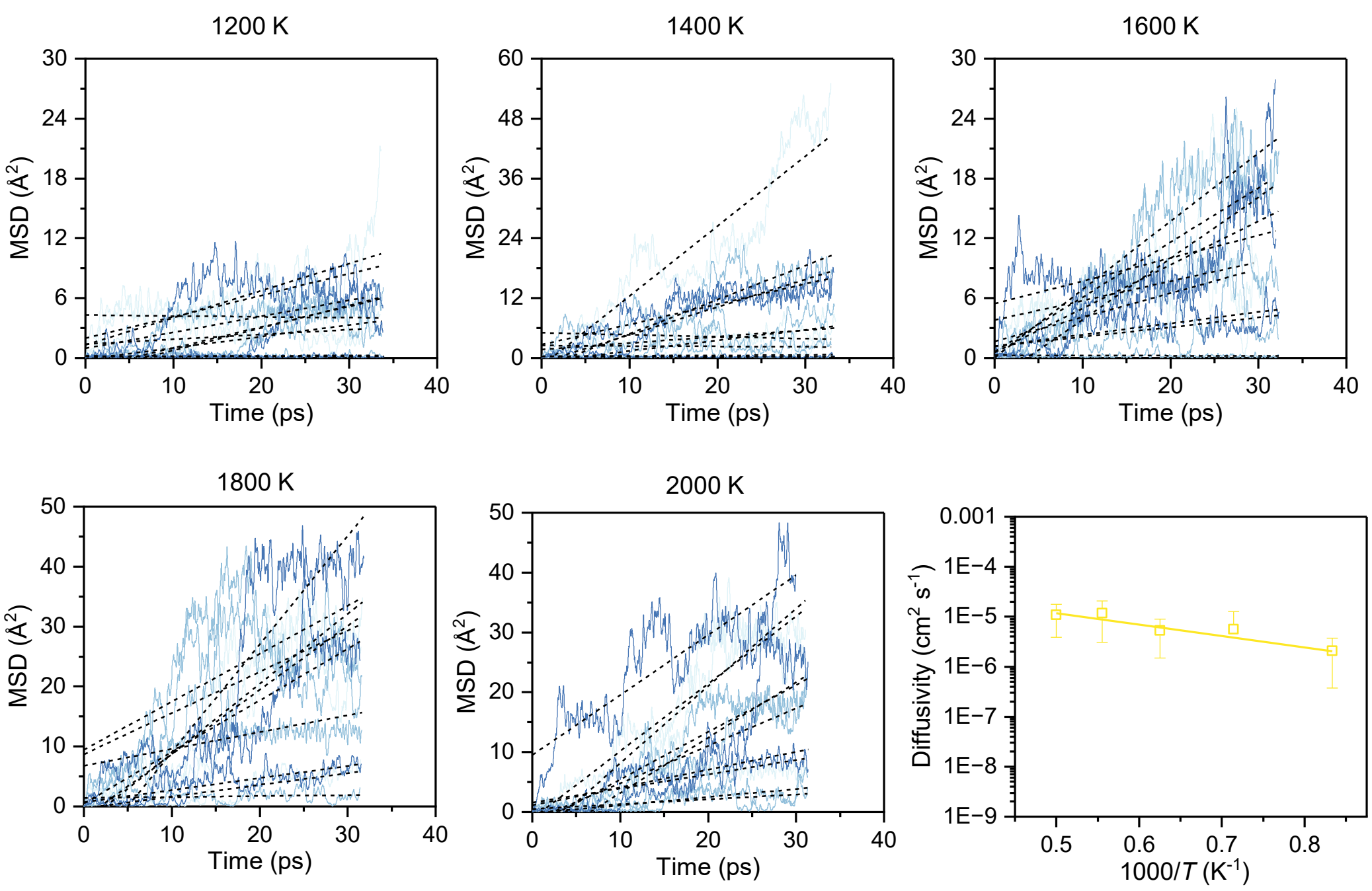
1200 K
1400 K
1600 K
1800 K
2000 K
MSD (Å²)
Time (ps)
Diffusivity (cm² s⁻¹)
1000/T (K⁻¹)

**Supplementary Fig. 3 | FAST/SPS. a**, Equipment schematic diagram. This technique operates within an integrated system. It allows for precise control of sintering parameters and real-time monitoring of the sample status and evolution during processing. The pressed powder is heated directly in a graphite die. Since the sample is confined in a cylindrical die, the deformation of the sample occurs only in the z-axis direction, resulting in the displacement of the sample. The chamber is continuously evacuated, and the pressure change within the chamber reflects the gas exhausting from the sample during sintering. Both the sample displacement and chamber pressure can be recorded to analyze the sintering behavior of ceramics. After the mother powder is loaded, the pressure in the chamber is evacuated to below 100 Pa and an external pressure (up to 58 Mpa) is then applied to the sample, leading to an initial increase in displacement. The sample is subsequently heated to 400 °C within seconds as the starting temperature, during which a peak is recorded on the pressure curve as the gas within the sample is expelled due to thermal expansion. Afterwards, the sample was heated linearly with 20-80 °C min$^{-1}$ until sintering finish. **b**, Photo of the die during high temperature sintering. **c**, Sintering examples of $Na_{3.4}Zr_2Si_{2.4}P_{0.6}O_{12}$ and $Li_{1.3}Al_{0.3}Ti_{1.7}(PO_4)_3$ demonstrating how pressure and displacement curves can be used to understand sintering behavior and determine sintering completion through inflection points. Once the experiment is completed, cooling begins immediately, allowing the sample to cool down to below 100 ℃ within 20 min. The equipment schematic diagram was created using Microsoft PowerPoint.

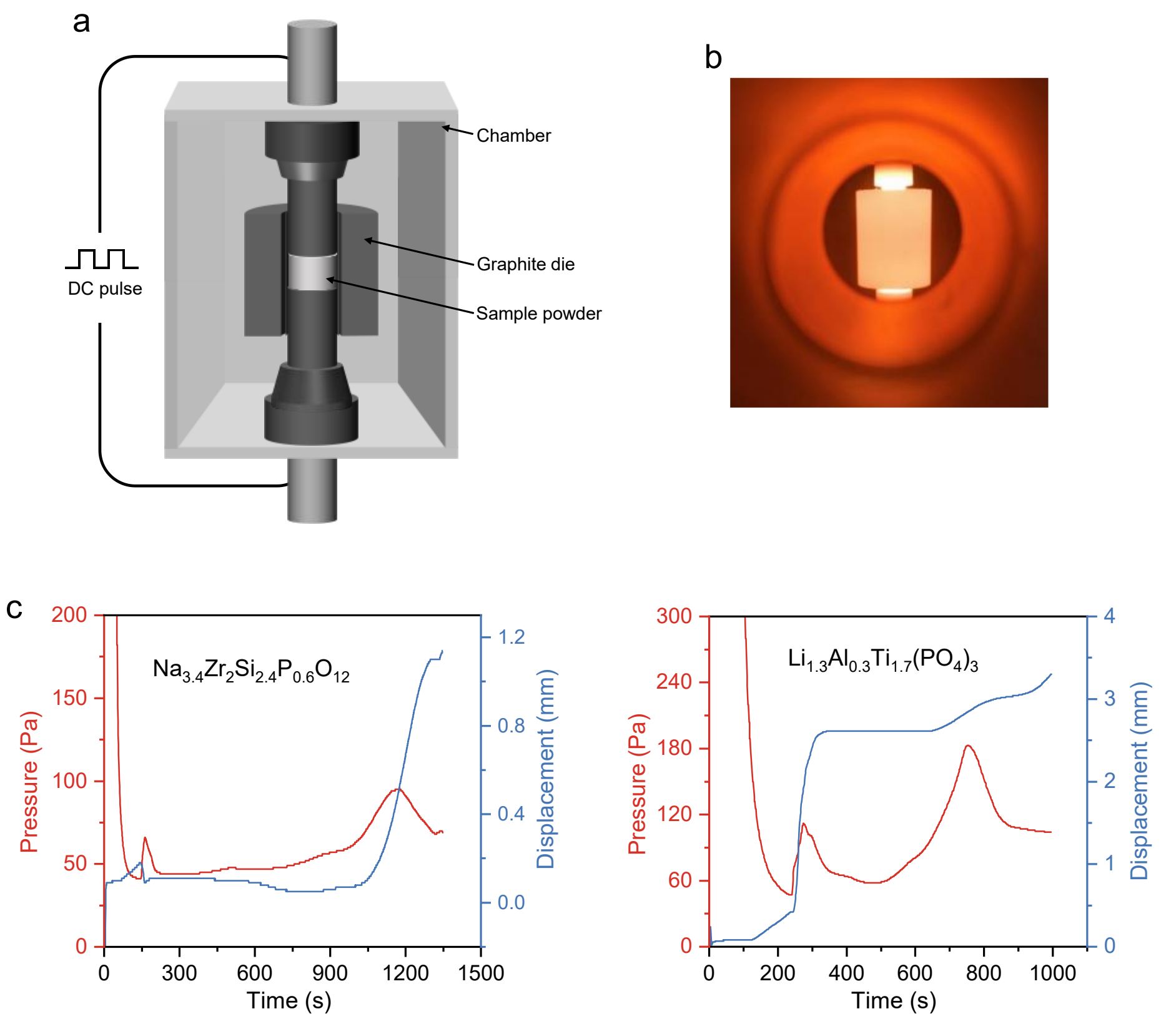

**Supplementary Fig. 4 | Characterizations of SF-LLZO. a**, SXRD refinement of SF-LLZO. The synchrotron diffraction wavelength is 0.4592 Å. **b**, Elements valence analysis. A comparison of SF-LLZO and S-LLZO reveals that all elemental spectra overlap significantly, with no observable peak shifts. This indicates that the elemental valence states in SF-LLZO are identical to those in S-LLZO. **c**, Particle size statistics of SF-LLZO from BSD images and mother powder from PSD measurements. **d**, SEM-EDS images of the cross-section of SF-LLZO. The scale bars are 10 μm. **e**-**h**, Representative HAADF-STEM image and STEM-EDS mappings of the GB of SF-LLZO. Scale bars are 200 nm (**e**), 500 nm (**f**), 2 μm (**g**,**h**). **i**, Representative HAADF-STEM image and STEM-EDS mappings of the GB of annealed SF-LLZO in air at 1000 ℃ for 1h. The scale bars are 3 nm. (**j**) Representative HAADF-STEM image and STEM-EDS mappings of the GB after long time FAST/SPS sintering, with 10 K min-1 heating rate and 1 h dwell time. The scale bars are 300 nm.

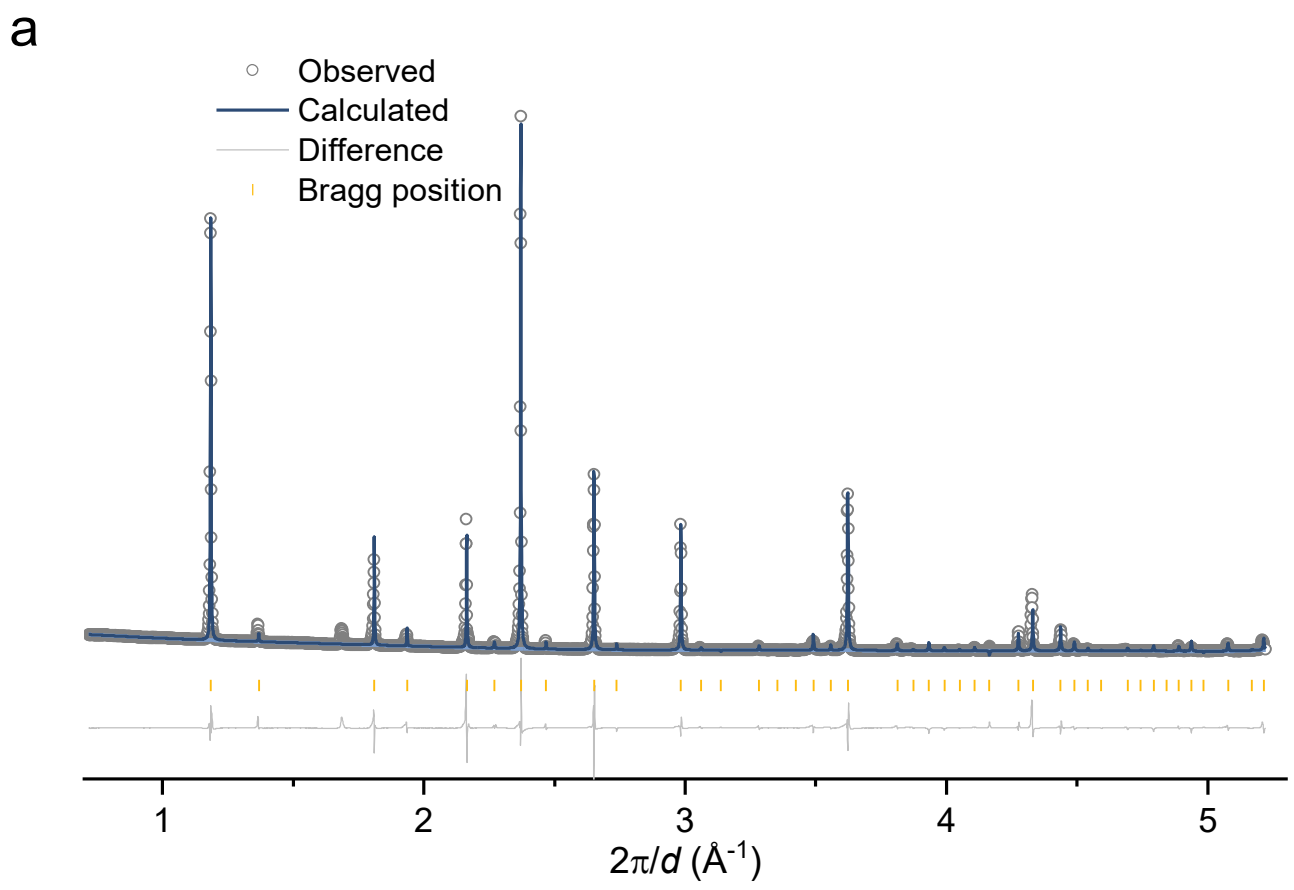


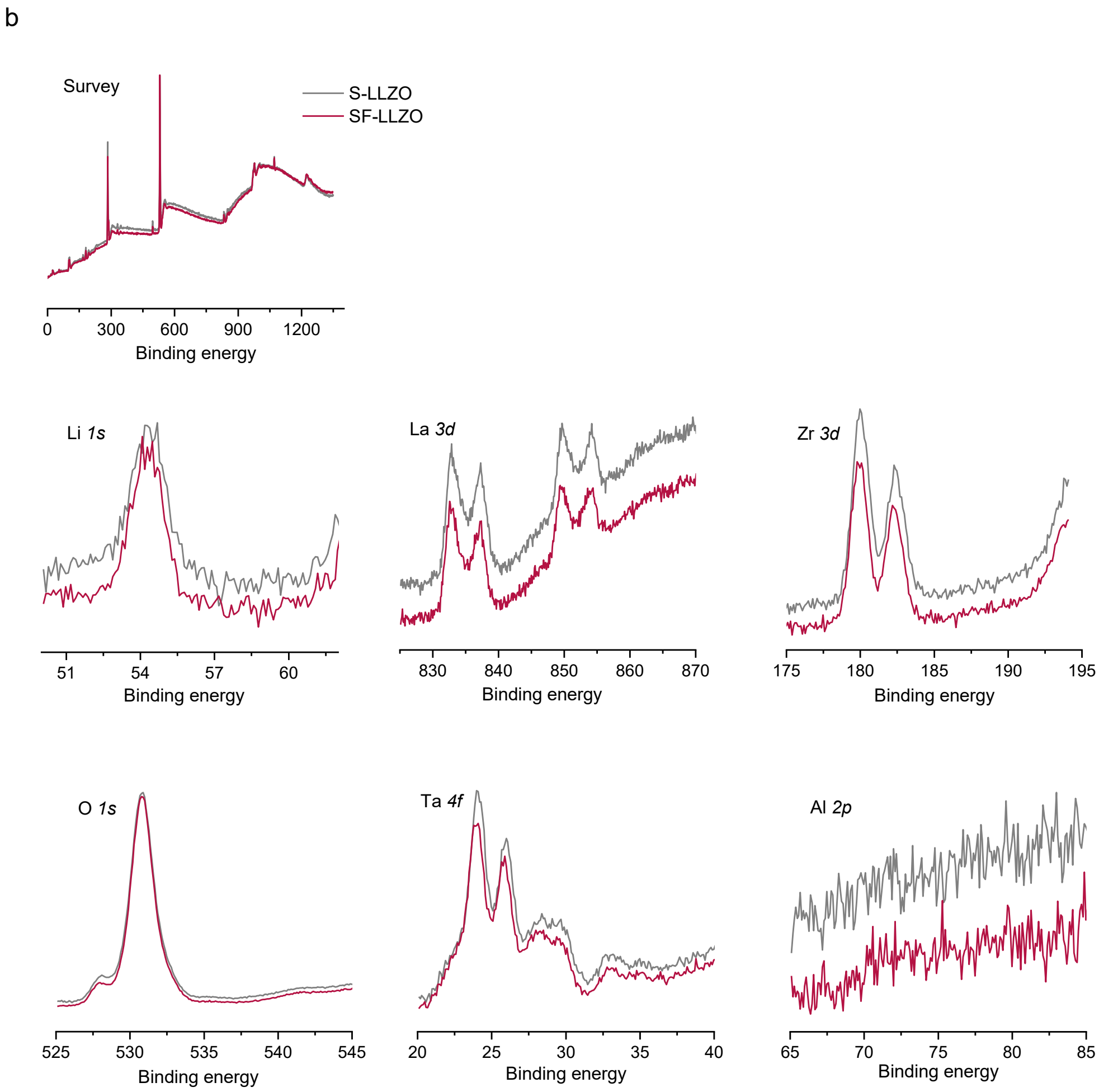

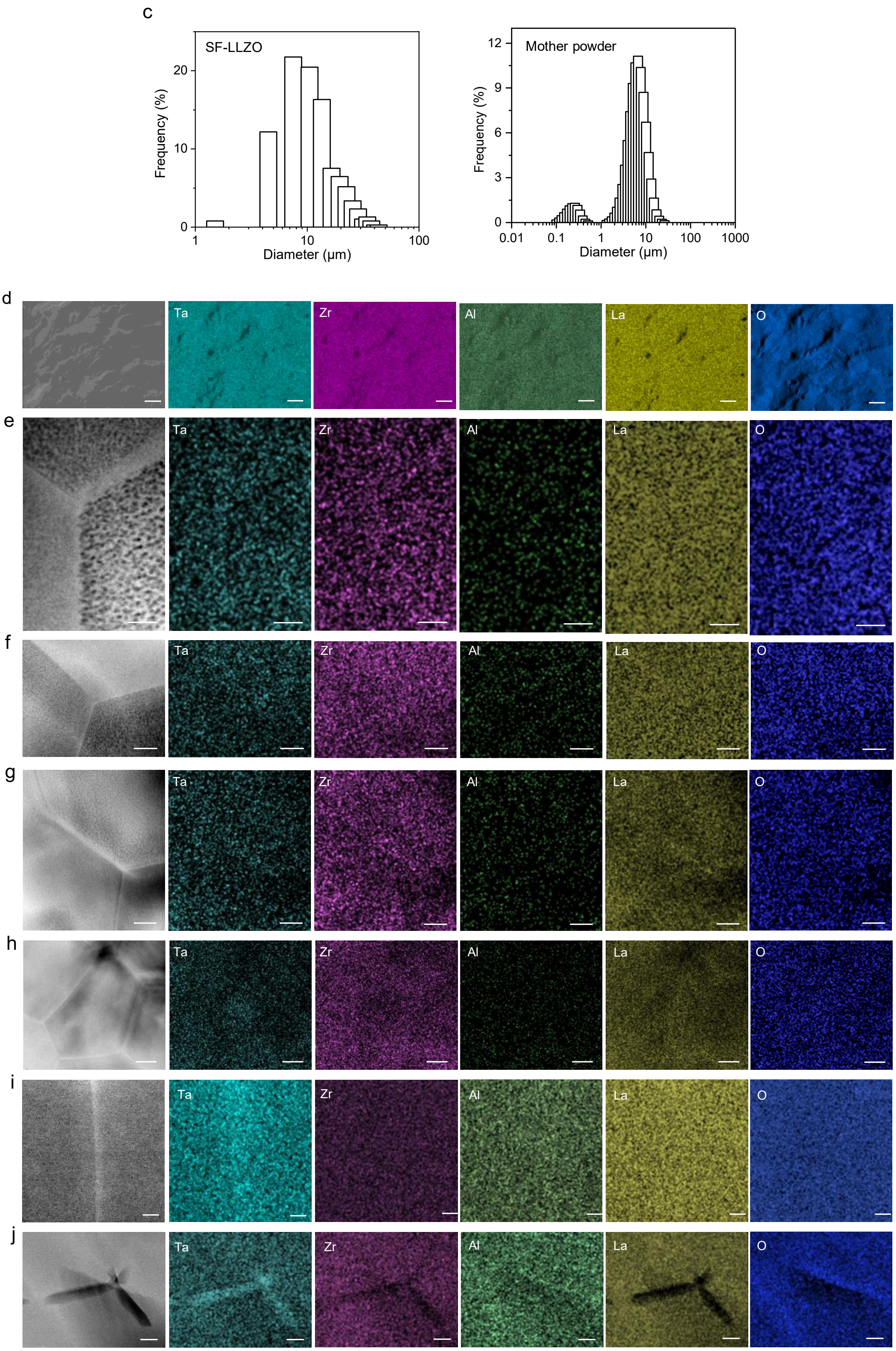
c
SF-LLZO
Frequency (%)
Diameter (μm)
0
10
20
1
10
100
Mother powder
Frequency (%)
Diameter (μm)
0
3
6
9
12
0.01
0.1
1
10
100
1000
d
e
f
g
h
i
j
Ta
Zr
Al
La
O

**Supplementary Fig. 5 | MLFF-MD for Li transport. a**,**b**,**c**, GB Σ17 (530) [001] (**a**), Σ33 (52-2) [011] (**b**), Σ19 (32-5) [111] (**c**), and corresponding Ta concentrations assigned as well as isosurfaces of Li possibilities at 1100 K for 1 ns with Ta segregation and no segregation cases. Green, yellow, cyan, purple, and blue spheres represent Li, La, Ta, Zr, and O, respectively. **d**, Arrhenius plots across temperatures 700, 900 and 1000 K. **e**, RDFs of Zr-Li and Ta-Li. **f**, DFT-calculated chemical potential differences between the GB area and grain interior regions in small GB models: Σ5 (120) [001], Σ3 (1-11) [011], and Σ3 (1-21) [111] with and without Ta doping. The differences were calculated by $\Delta\mu = (\mu_{GB} - \mu_{bulk\ average})$. **g**, DFT-calculated chemical potential differences between cubic and amorphous LLZO structures, with and without Ta doping. The box plot presents the distribution of the 56 Δμ values with Ta doping and 52 Δμ values without Ta doping, showing the mean (as a square), the median (as a line), box (25th–75th percentiles), and whiskers (range of non-outlier data points up to 1.5 × IQR from the box).

a

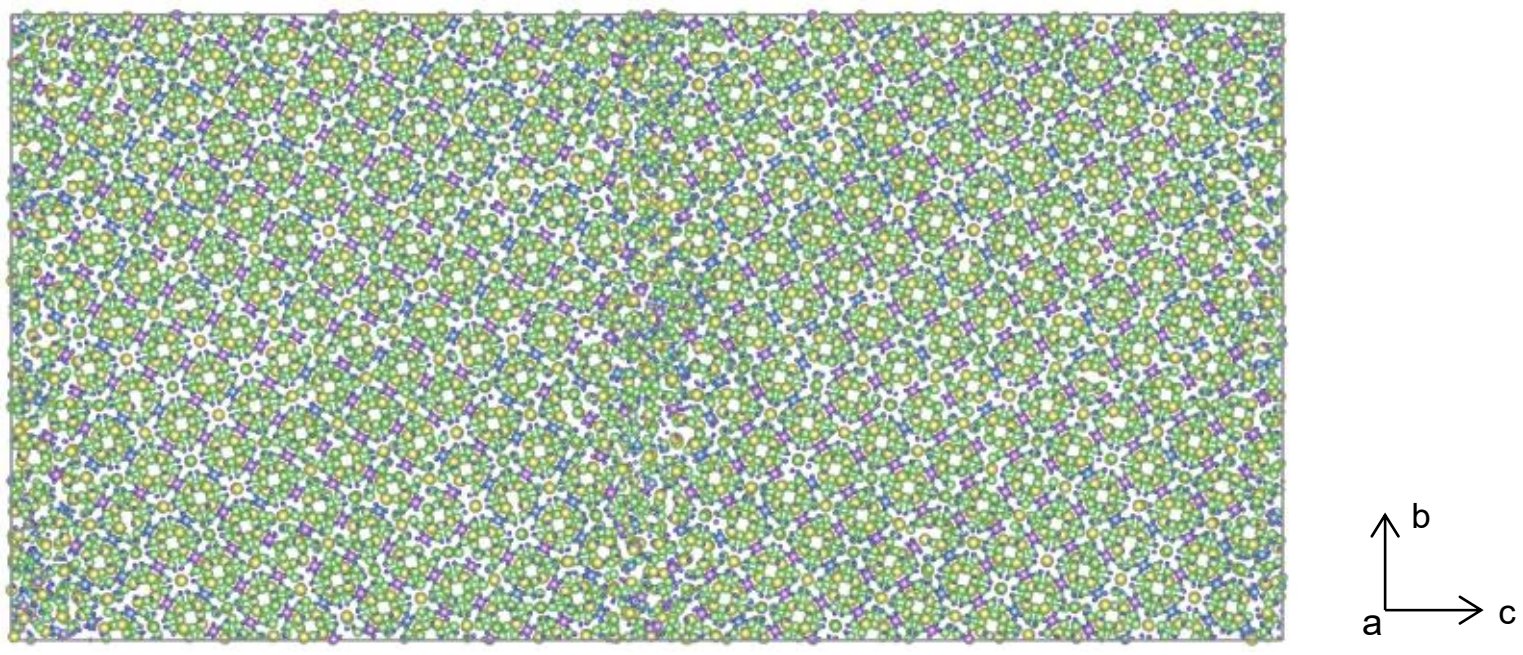


Σ17 (530) [001]

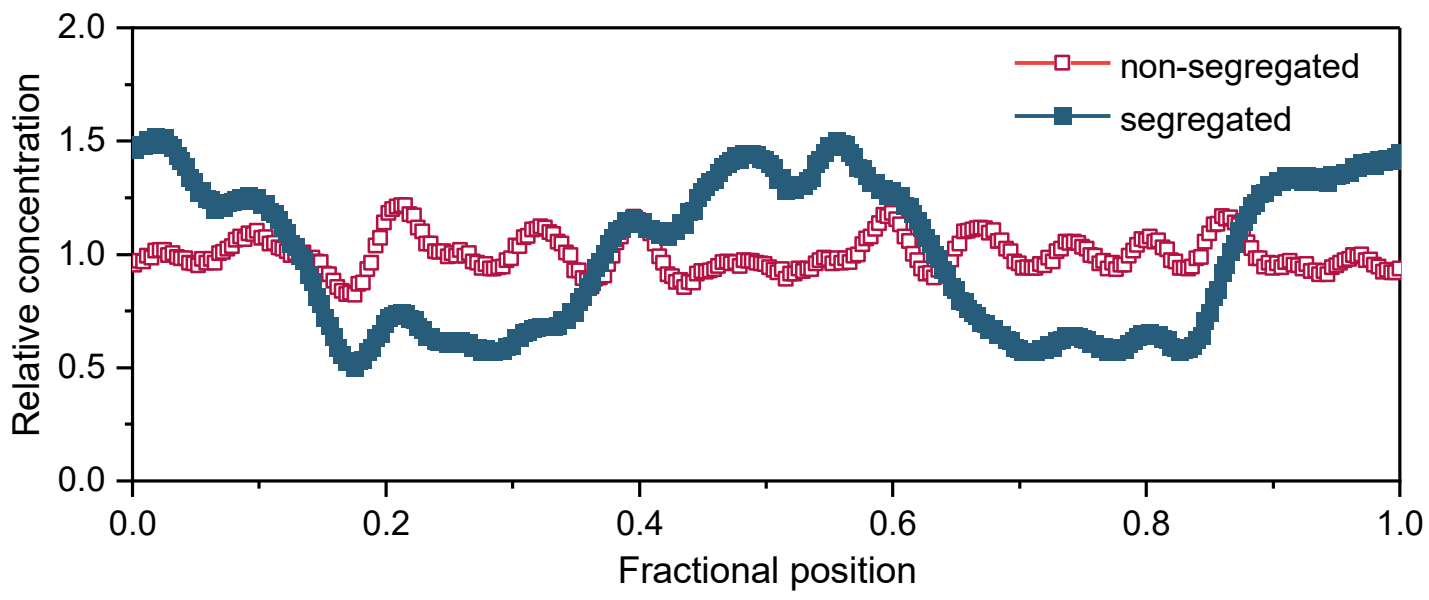


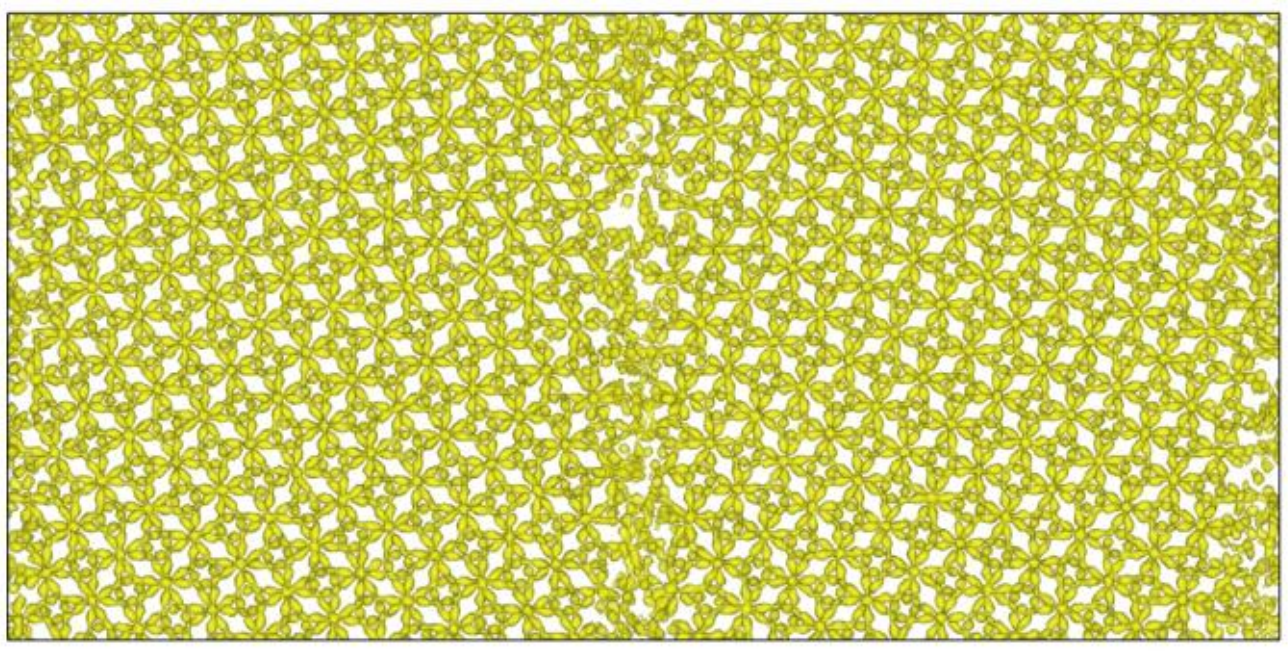

Segregated

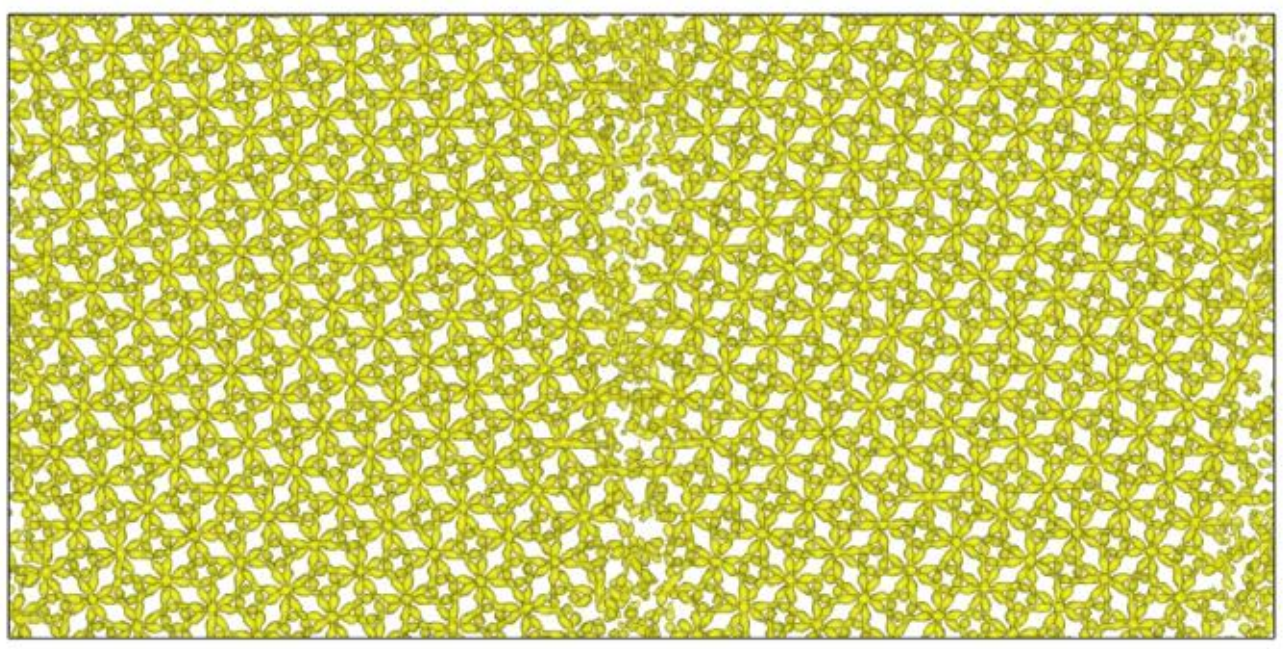

Non-segregated

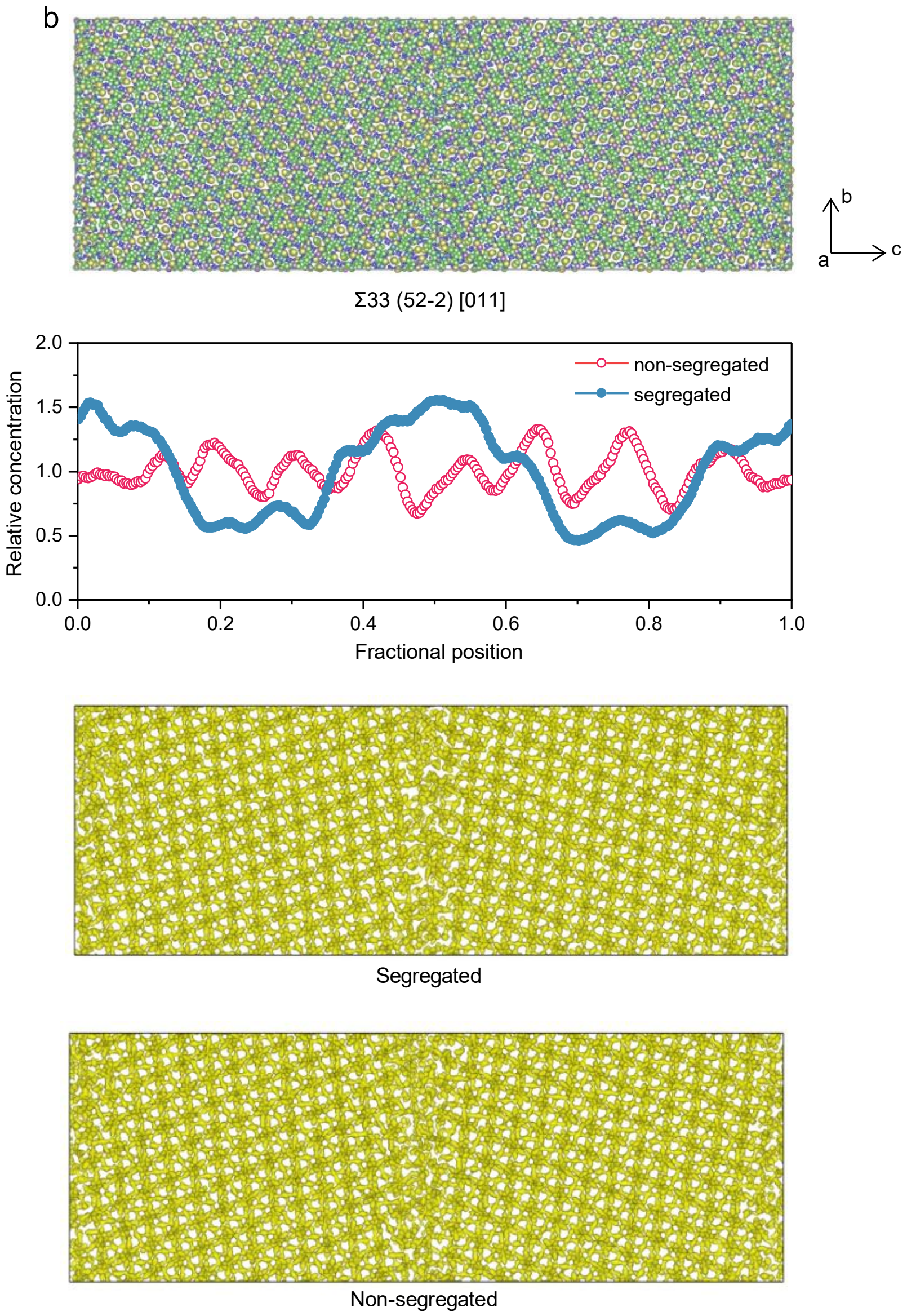
b
b
a
c
Σ33 (52-2) [011]
non-segregated
segregated
Relative concentration
0.0
0.5
1.0
1.5
2.0
0.0
0.2
0.4
0.6
0.8
1.0
Fractional position
Segregated
Non-segregated

c

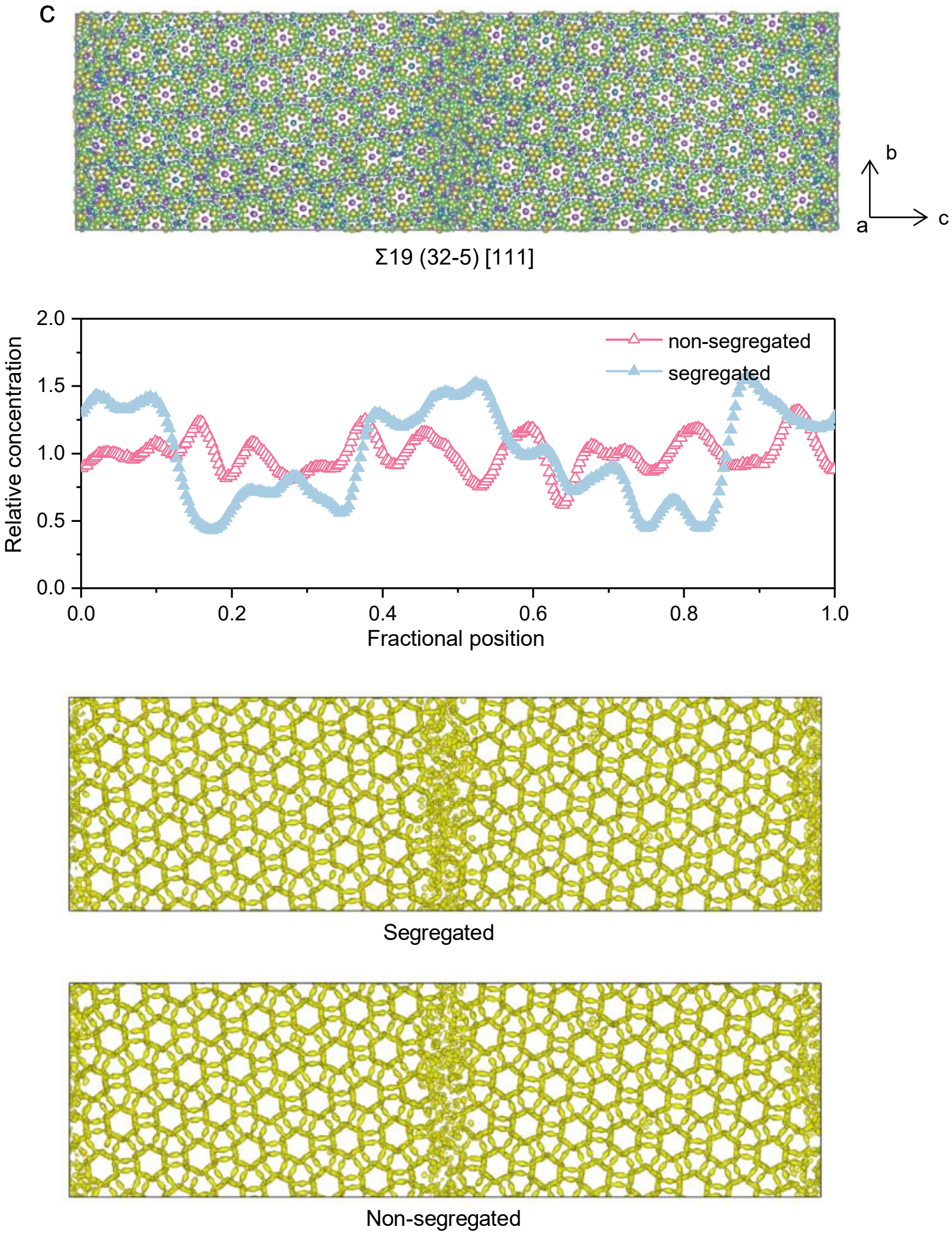
b
a
c
Σ19 (32-5) [111]
2.0
1.5
1.0
0.5
0.0
Relative concentration
0.0
0.2
0.4
0.6
0.8
1.0
Fractional position
non-segregated
segregated
Segregated
Non-segregated

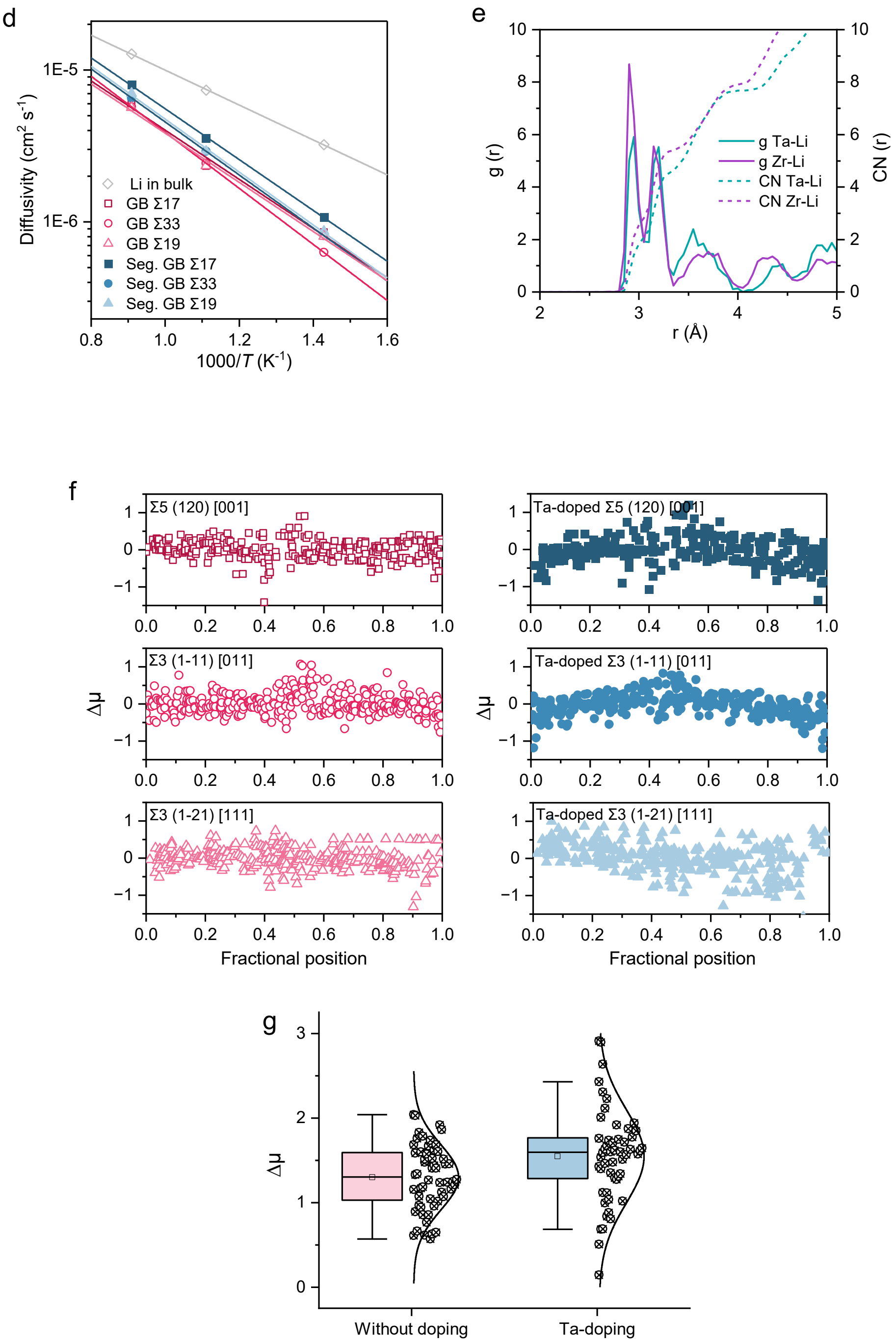
d
Diffusivity (cm² s⁻¹)
1E-5
1E-6
0.8
1.0
1.2
1.4
1.6
1000/T (K⁻¹)
Li in bulk
GB Σ17
GB Σ33
GB Σ19
Seg. GB Σ17
Seg. GB Σ33
Seg. GB Σ19
e
g (r)
CN (r)
r (Å)
g Ta-Li
g Zr-Li
CN Ta-Li
CN Zr-Li
f
Σ5 (120) [001]
Σ3 (1-11) [011]
Σ3 (1-21) [111]
Ta-doped Σ5 (120) [001]
Ta-doped Σ3 (1-11) [011]
Ta-doped Σ3 (1-21) [111]
Δμ
Fractional position
g
Δμ
Without doping
Ta-doping

**Supplementary Fig. 6 | Li-rich compound formation.** **a**, Li metal formation from Al, Ta-doped LLZO in SEM with extended exposure time. Scale bars are 1 μm. With the extended exposure time, Li metal precipitates on the LLZO surface and gradually grows. **b**, Li compounds on Al, Ta-doped LLZO in HAADF-STEM. Scale bar is 40 nm. Black spots were observed along both sides of the GBs and in regions further away. These spots are hypothesized to be Li-containing compounds. This is because specimen preparation can promote Li formation on the LLZO surface, which is then expected to be rapidly oxidized during sample transfer, thereby forming Li-rich compounds. **c**, Li compound formation from Ga-doped LLZO in HAADF-STEM. Scale bar is 5 nm. Li compounds were observed to form along both sides of the GB, similar to the Al, Ta-LLZO. **d-f**, Li compound formation from Ga-doped LLZO in HAADF-STEM (**d**) (Scale bar is 10 nm), enlarged left side (**e**) (Scale bar is 5 nm) and enlarged right side (**f**) (Scale bar is 5 nm). Insets show lattice planes, and yellow and purple spheres represent La and Zr, respectively. The orientations of the grains forming either side of the GB were identified as ⟨111⟩ and ⟨110⟩. More Li compounds were observed on the ⟨111⟩ surface compared to ⟨110⟩, indicating that Li is more readily reduced from ⟨111⟩.

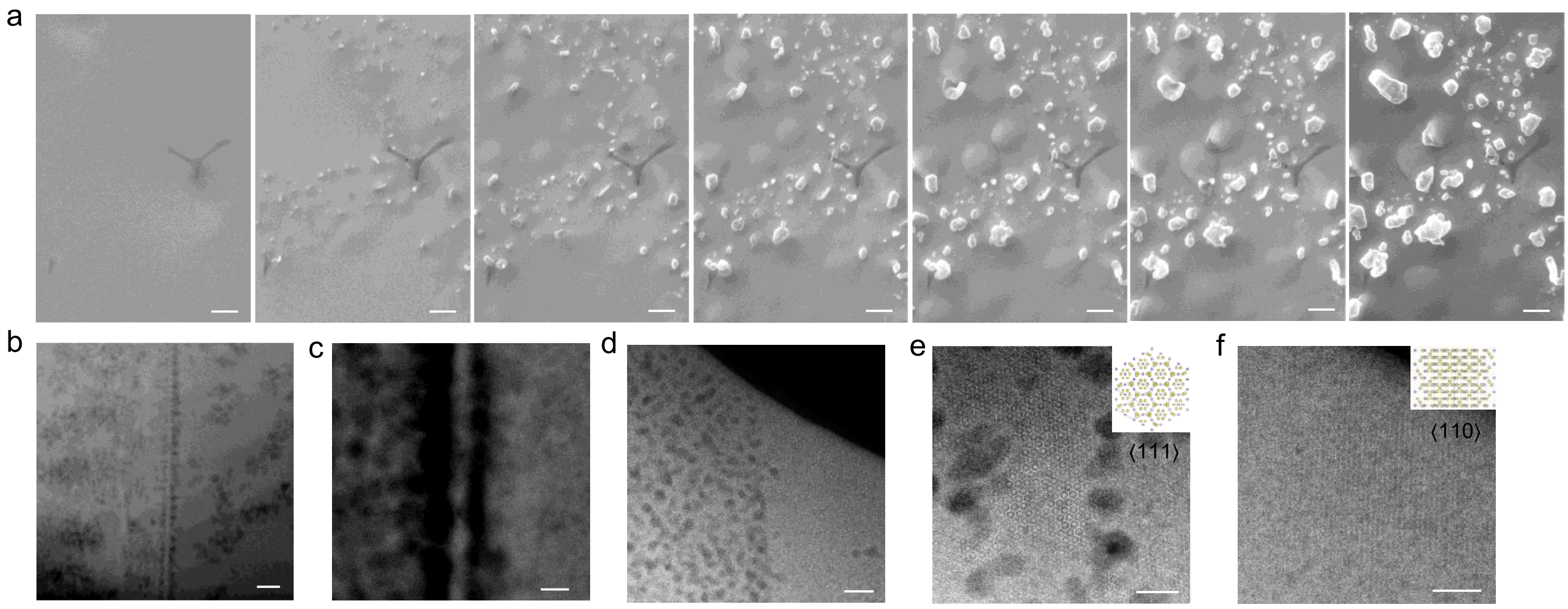

**Supplementary Fig. 7 | MLFF-MD for Li vacancy formation energy. a-c,** GB Σ17 (530) [001] (**a**), Li vacancy formation energies with Ta segregation and no segregation (**b**) and RDFs at bulk and GB (**c**). **d-f,** GB Σ33 (52-2) [011] (**d**), Li vacancy formation energies with Ta segregation and no segregation (**e**) and RDFs at bulk and GB (**f**). (**g**) RDFs at bulk and GB in Σ19 (32-5) [111]. Green, yellow, cyan, purple, and blue spheres represent Li, La, Ta, Zr, and O, respectively.

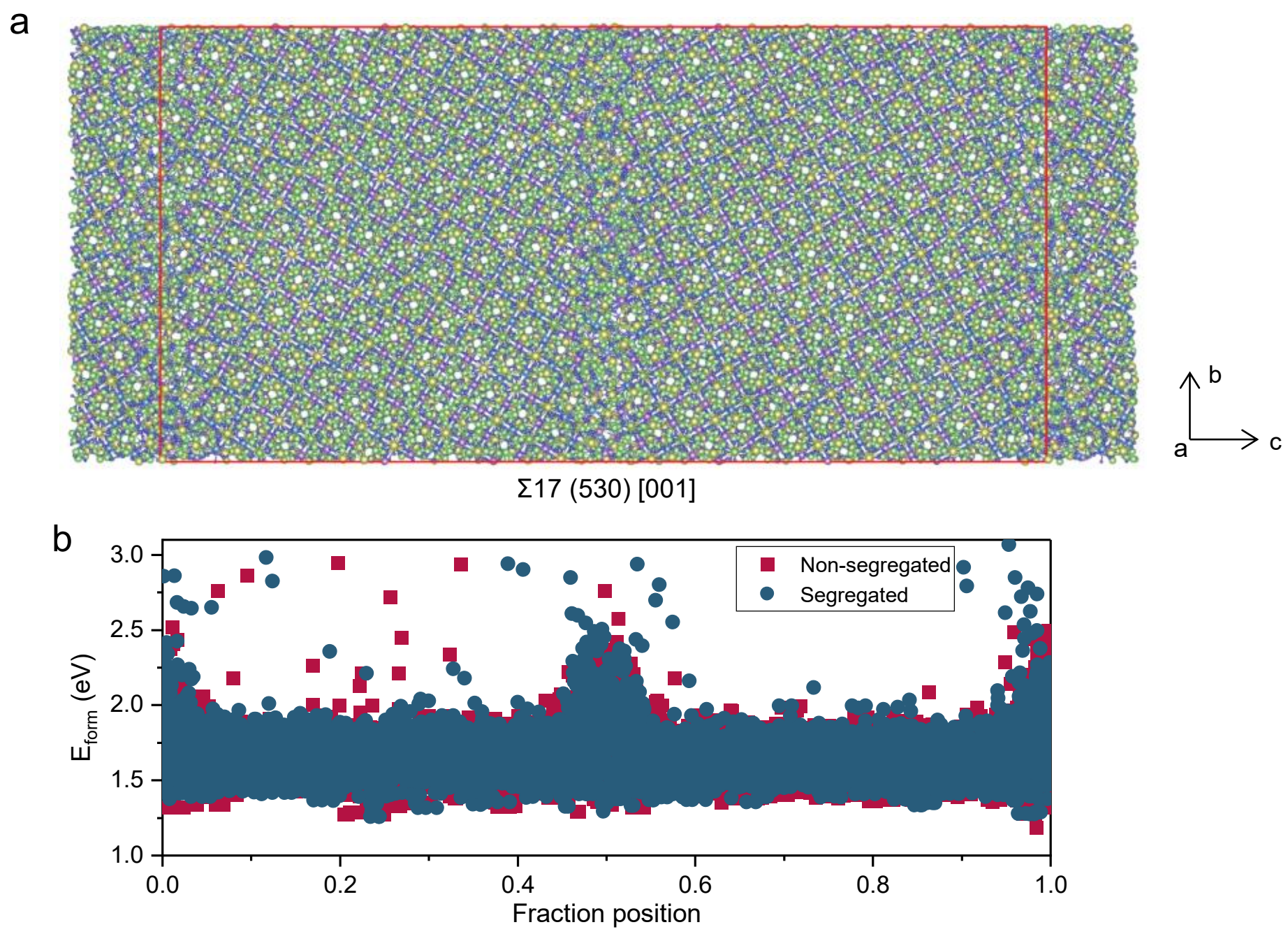

c

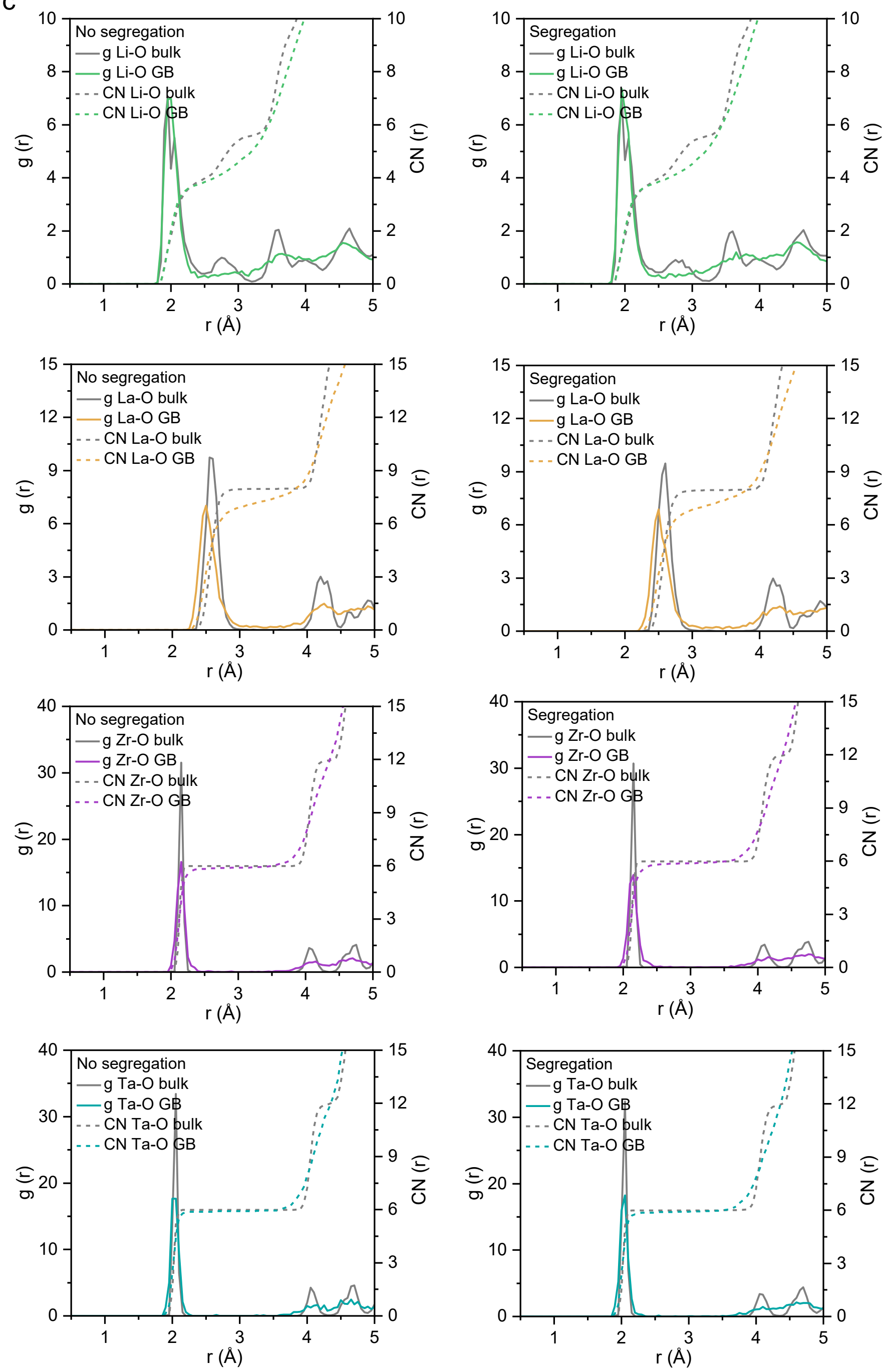
No segregation
g Li-O bulk
g Li-O GB
CN Li-O bulk
CN Li-O GB
Segregation
g Li-O bulk
g Li-O GB
CN Li-O bulk
CN Li-O GB
No segregation
g La-O bulk
g La-O GB
CN La-O bulk
CN La-O GB
Segregation
g La-O bulk
g La-O GB
CN La-O bulk
CN La-O GB
No segregation
g Zr-O bulk
g Zr-O GB
CN Zr-O bulk
CN Zr-O GB
Segregation
g Zr-O bulk
g Zr-O GB
CN Zr-O bulk
CN Zr-O GB
No segregation
g Ta-O bulk
g Ta-O GB
CN Ta-O bulk
CN Ta-O GB
Segregation
g Ta-O bulk
g Ta-O GB
CN Ta-O bulk
CN Ta-O GB
g (r)
CN (r)
r (Å)

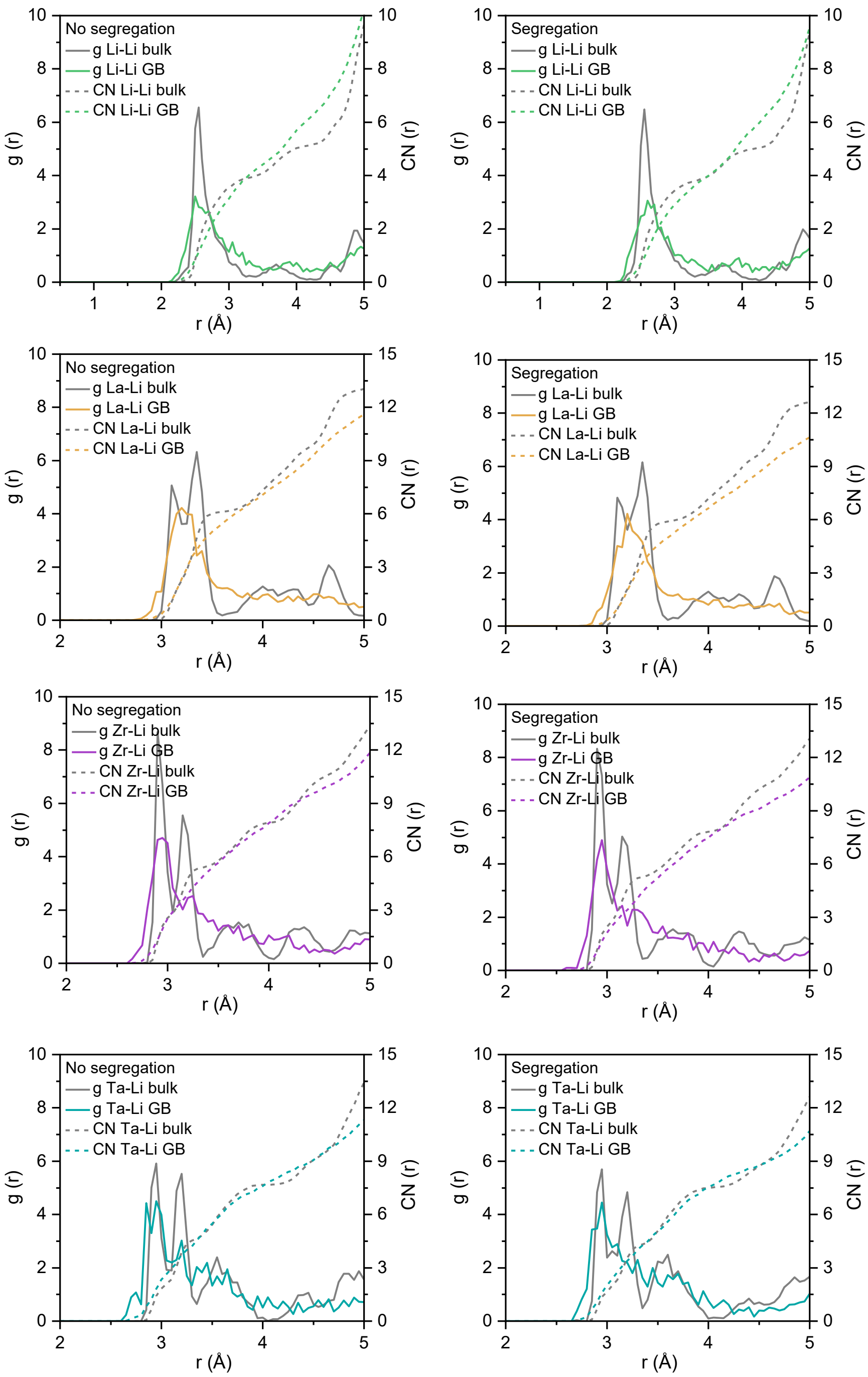
No segregation
g Li-Li bulk
g Li-Li GB
CN Li-Li bulk
CN Li-Li GB
Segregation
g Li-Li bulk
g Li-Li GB
CN Li-Li bulk
CN Li-Li GB
No segregation
g La-Li bulk
g La-Li GB
CN La-Li bulk
CN La-Li GB
Segregation
g La-Li bulk
g La-Li GB
CN La-Li bulk
CN La-Li GB
No segregation
g Zr-Li bulk
g Zr-Li GB
CN Zr-Li bulk
CN Zr-Li GB
Segregation
g Zr-Li bulk
g Zr-Li GB
CN Zr-Li bulk
CN Zr-Li GB
No segregation
g Ta-Li bulk
g Ta-Li GB
CN Ta-Li bulk
CN Ta-Li GB
Segregation
g Ta-Li bulk
g Ta-Li GB
CN Ta-Li bulk
CN Ta-Li GB
g (r)
CN (r)
r (Å)

d

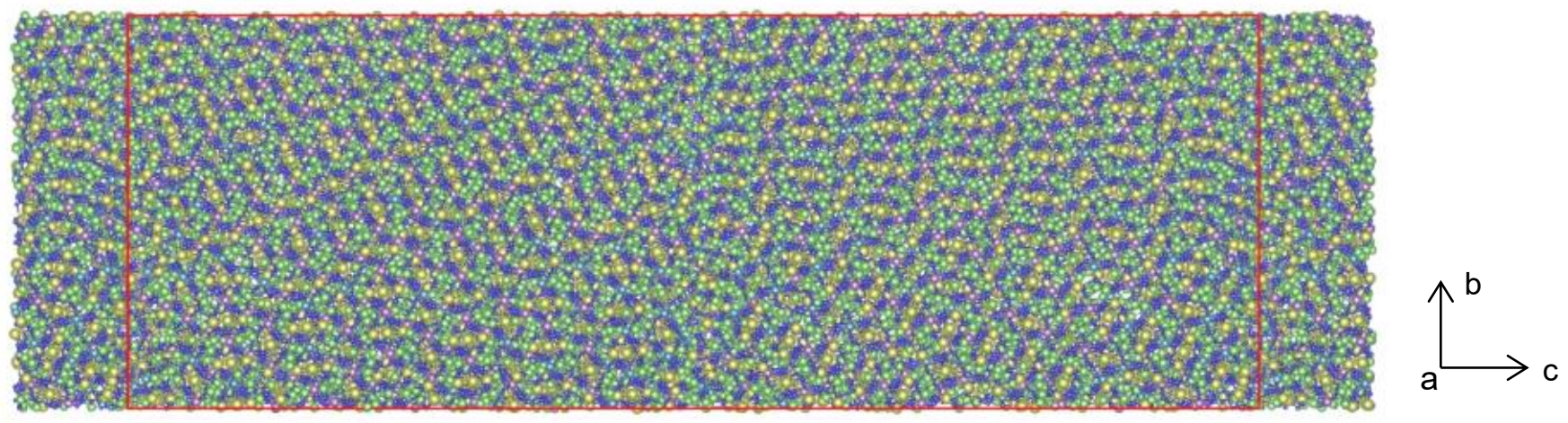


Σ33 (52-2) [011]

e

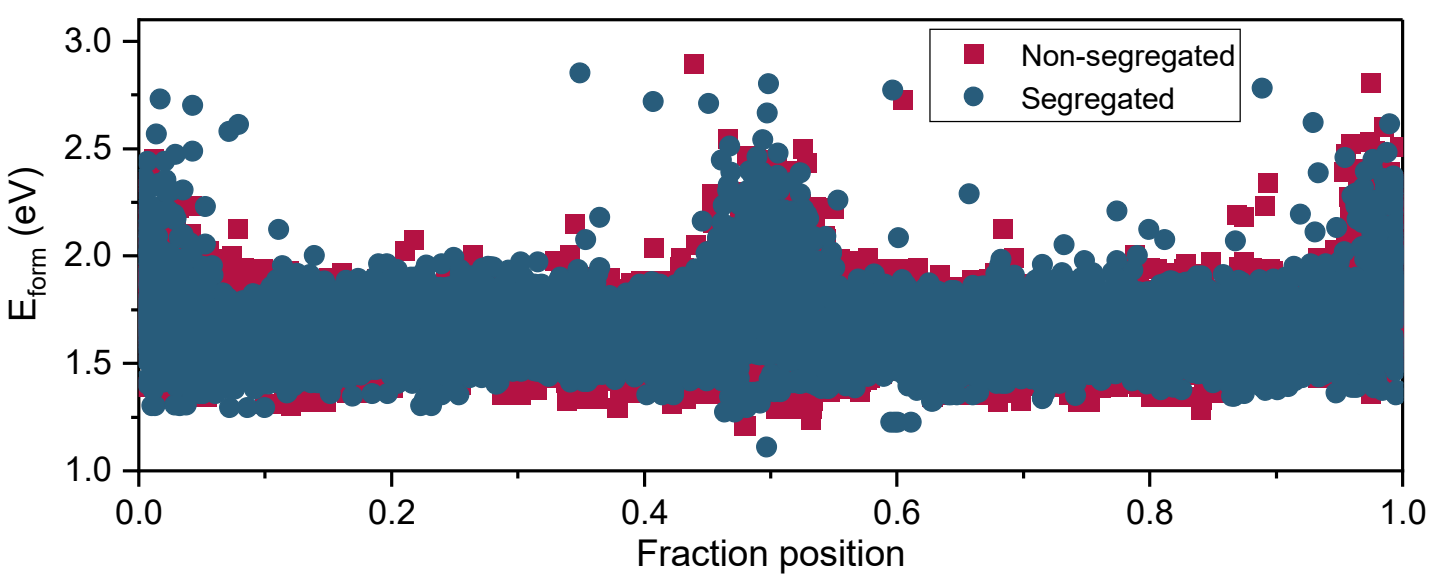

f

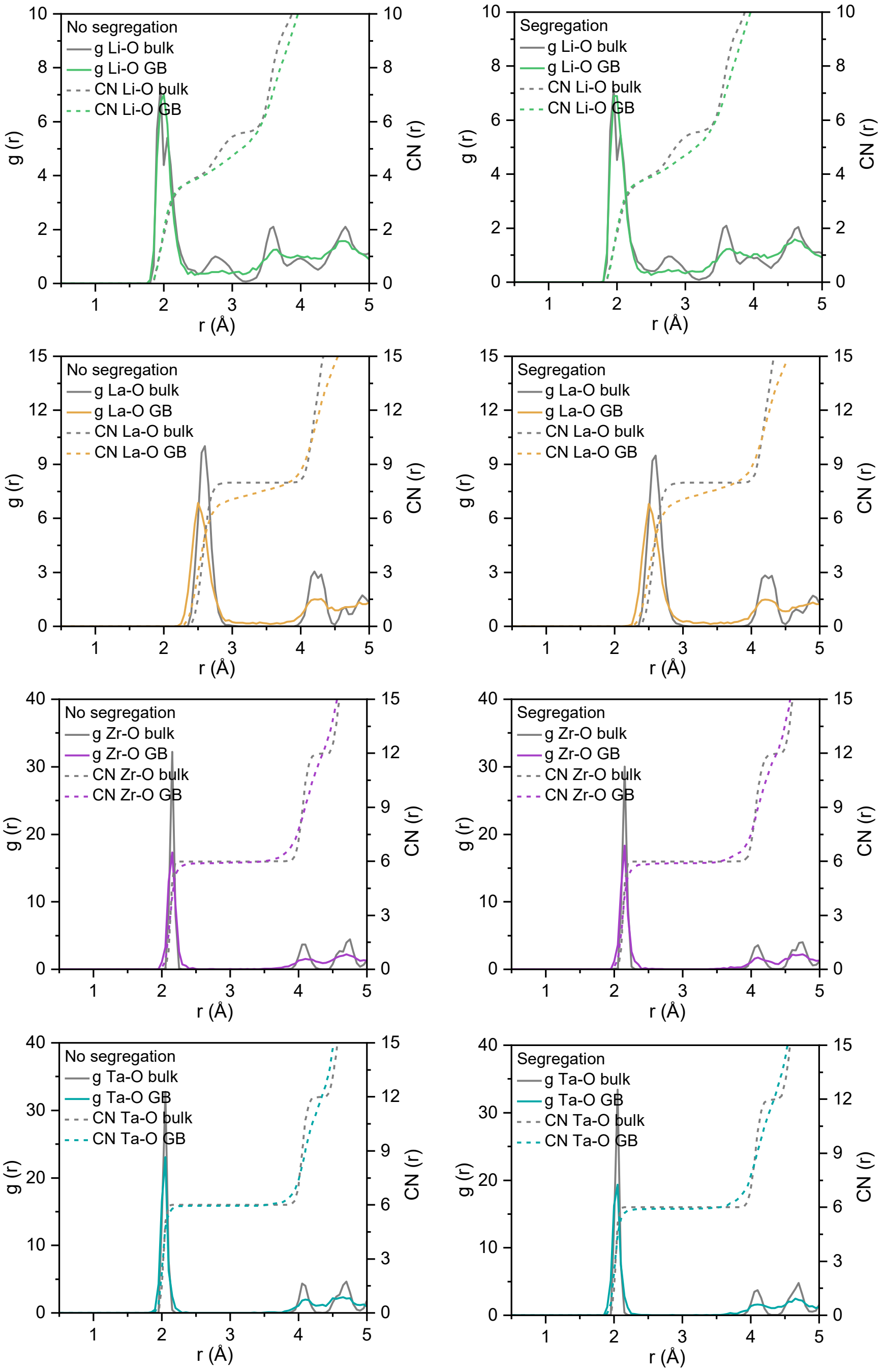

No segregation
g Li-O bulk
g Li-O GB
CN Li-O bulk
CN Li-O GB
Segregation
No segregation
g La-O bulk
g La-O GB
CN La-O bulk
CN La-O GB
No segregation
g Zr-O bulk
g Zr-O GB
CN Zr-O bulk
CN Zr-O GB
No segregation
g Ta-O bulk
g Ta-O GB
CN Ta-O bulk
CN Ta-O GB
g (r)
CN (r)
r (Å)

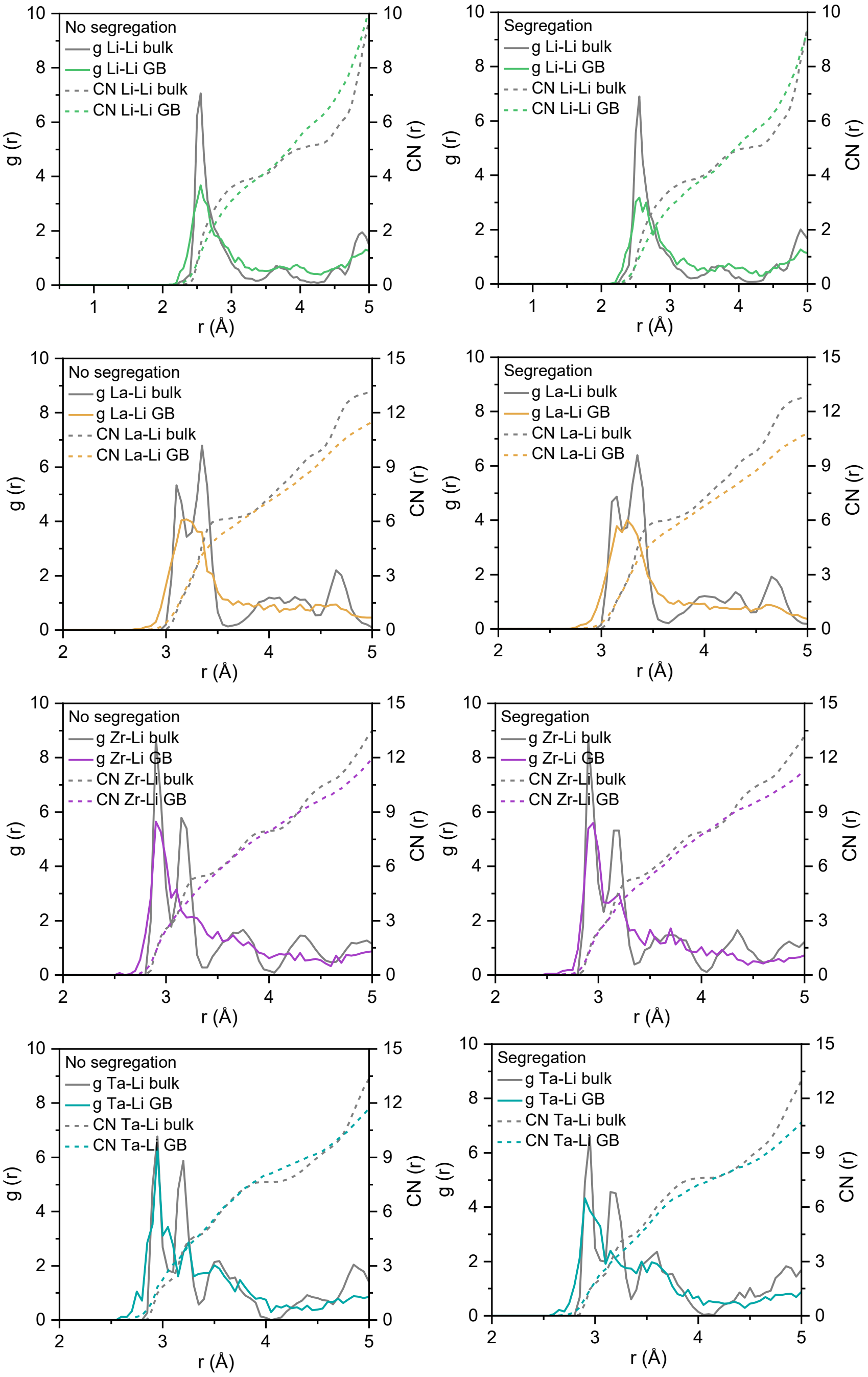
No segregation
g Li-Li bulk
g Li-Li GB
CN Li-Li bulk
CN Li-Li GB
Segregation
g Li-Li bulk
g Li-Li GB
CN Li-Li bulk
CN Li-Li GB
No segregation
g La-Li bulk
g La-Li GB
CN La-Li bulk
CN La-Li GB
Segregation
g La-Li bulk
g La-Li GB
CN La-Li bulk
CN La-Li GB
No segregation
g Zr-Li bulk
g Zr-Li GB
CN Zr-Li bulk
CN Zr-Li GB
Segregation
g Zr-Li bulk
g Zr-Li GB
CN Zr-Li bulk
CN Zr-Li GB
No segregation
g Ta-Li bulk
g Ta-Li GB
CN Ta-Li bulk
CN Ta-Li GB
Segregation
g Ta-Li bulk
g Ta-Li GB
CN Ta-Li bulk
CN Ta-Li GB
g (r)
CN (r)
r (Å)

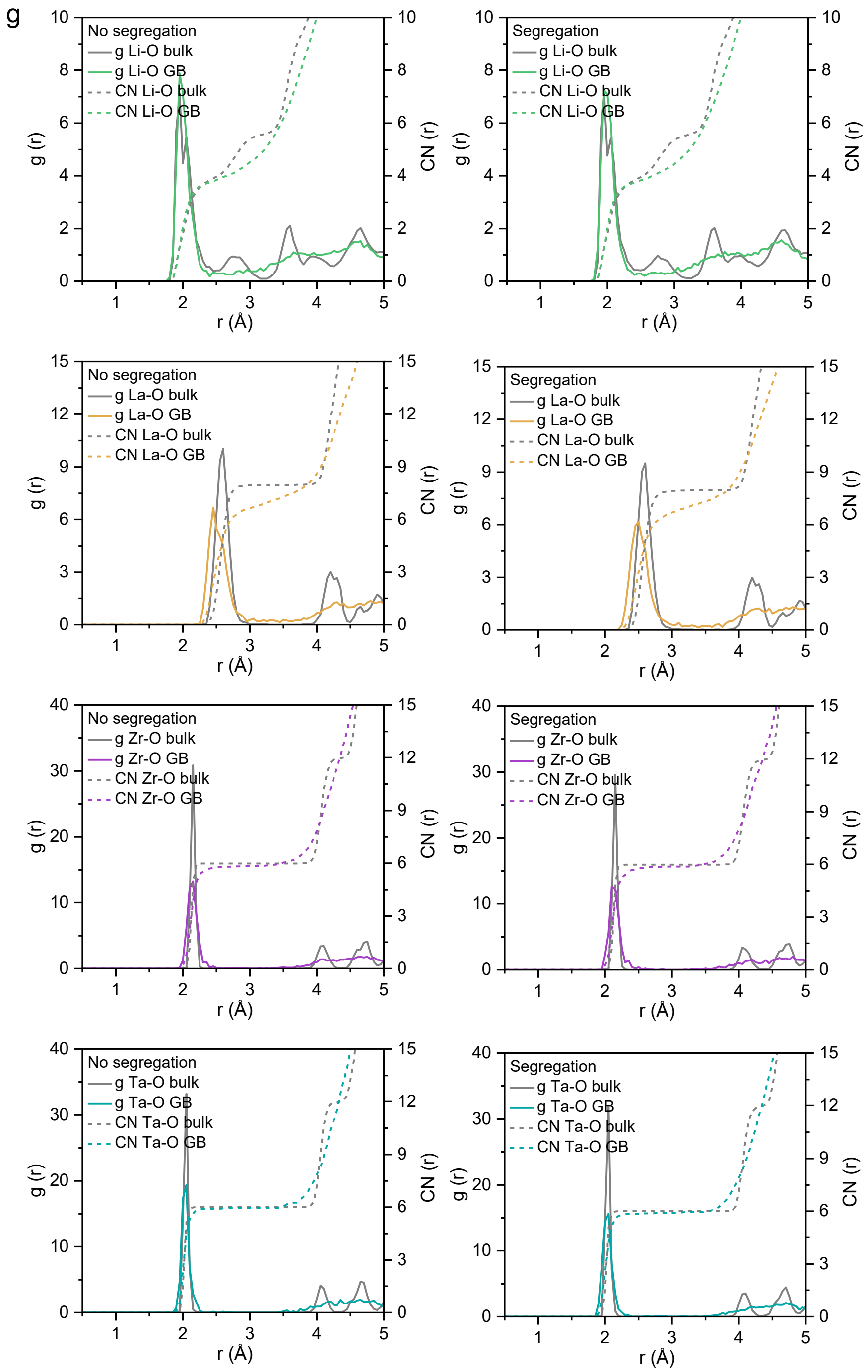

g
No segregation
g Li-O bulk
g Li-O GB
CN Li-O bulk
CN Li-O GB
Segregation
g Li-O bulk
g Li-O GB
CN Li-O bulk
CN Li-O GB
No segregation
g La-O bulk
g La-O GB
CN La-O bulk
CN La-O GB
Segregation
g La-O bulk
g La-O GB
CN La-O bulk
CN La-O GB
No segregation
g Zr-O bulk
g Zr-O GB
CN Zr-O bulk
CN Zr-O GB
Segregation
g Zr-O bulk
g Zr-O GB
CN Zr-O bulk
CN Zr-O GB
No segregation
g Ta-O bulk
g Ta-O GB
CN Ta-O bulk
CN Ta-O GB
Segregation
g Ta-O bulk
g Ta-O GB
CN Ta-O bulk
CN Ta-O GB
g (r)
CN (r)
r (Å)

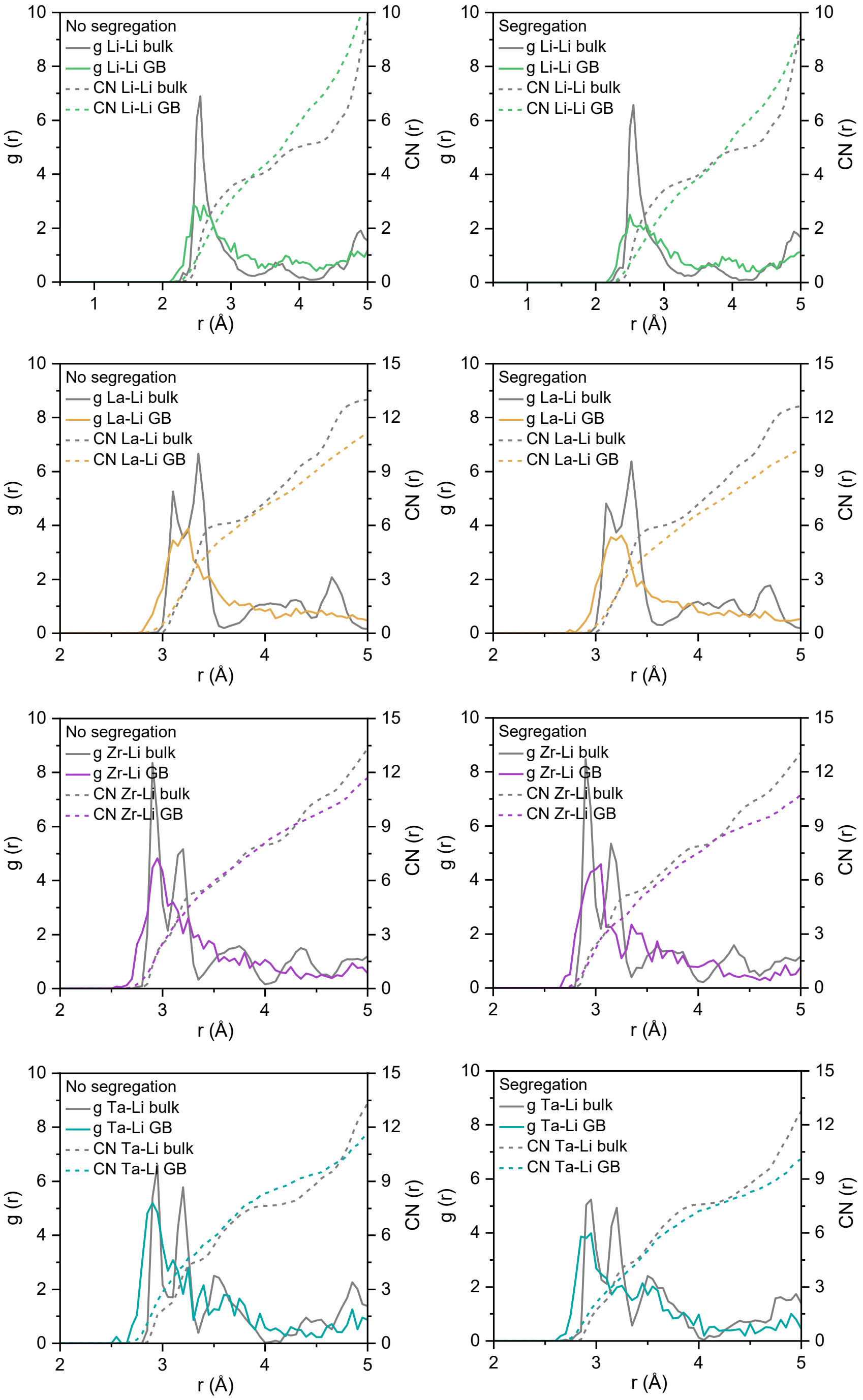

No segregation
g Li-Li bulk
g Li-Li GB
CN Li-Li bulk
CN Li-Li GB
Segregation
g Li-Li bulk
g Li-Li GB
CN Li-Li bulk
CN Li-Li GB
No segregation
g La-Li bulk
g La-Li GB
CN La-Li bulk
CN La-Li GB
Segregation
g La-Li bulk
g La-Li GB
CN La-Li bulk
CN La-Li GB
No segregation
g Zr-Li bulk
g Zr-Li GB
CN Zr-Li bulk
CN Zr-Li GB
Segregation
g Zr-Li bulk
g Zr-Li GB
CN Zr-Li bulk
CN Zr-Li GB
No segregation
g Ta-Li bulk
g Ta-Li GB
CN Ta-Li bulk
CN Ta-Li GB
Segregation
g Ta-Li bulk
g Ta-Li GB
CN Ta-Li bulk
CN Ta-Li GB
g (r)
CN (r)
r (Å)

**Supplementary Fig. 8 | Post-mortem EDS analysis after dendrite penetration. a**,**b**, Local cross-sectional (**a**), and fractured (**b**) EDS mappings at cracks after dendrite penetrating SF-LLZO. Scale bars are 50 μm (**a**) and 10 μm (**b**). Post-mortem analysis of batteries cycled at room temperature revealed complete dendrite penetration through the pellets after 500 plating/stripping cycles with 2 h per cycle at current densities of 0.5 mA cm$^{-2}$ for S-LLZO and 1 mA cm$^{-2}$ for SF-LLZO. Batteries for post-mortem analysis were disassembled after 500 plating/stripping cycles with 2 h per cycle at current densities of 0.5 mA cm$^{-2}$ for S-LLZO and 1 mA cm$^{-2}$ for SF-LLZO at 25 ℃.

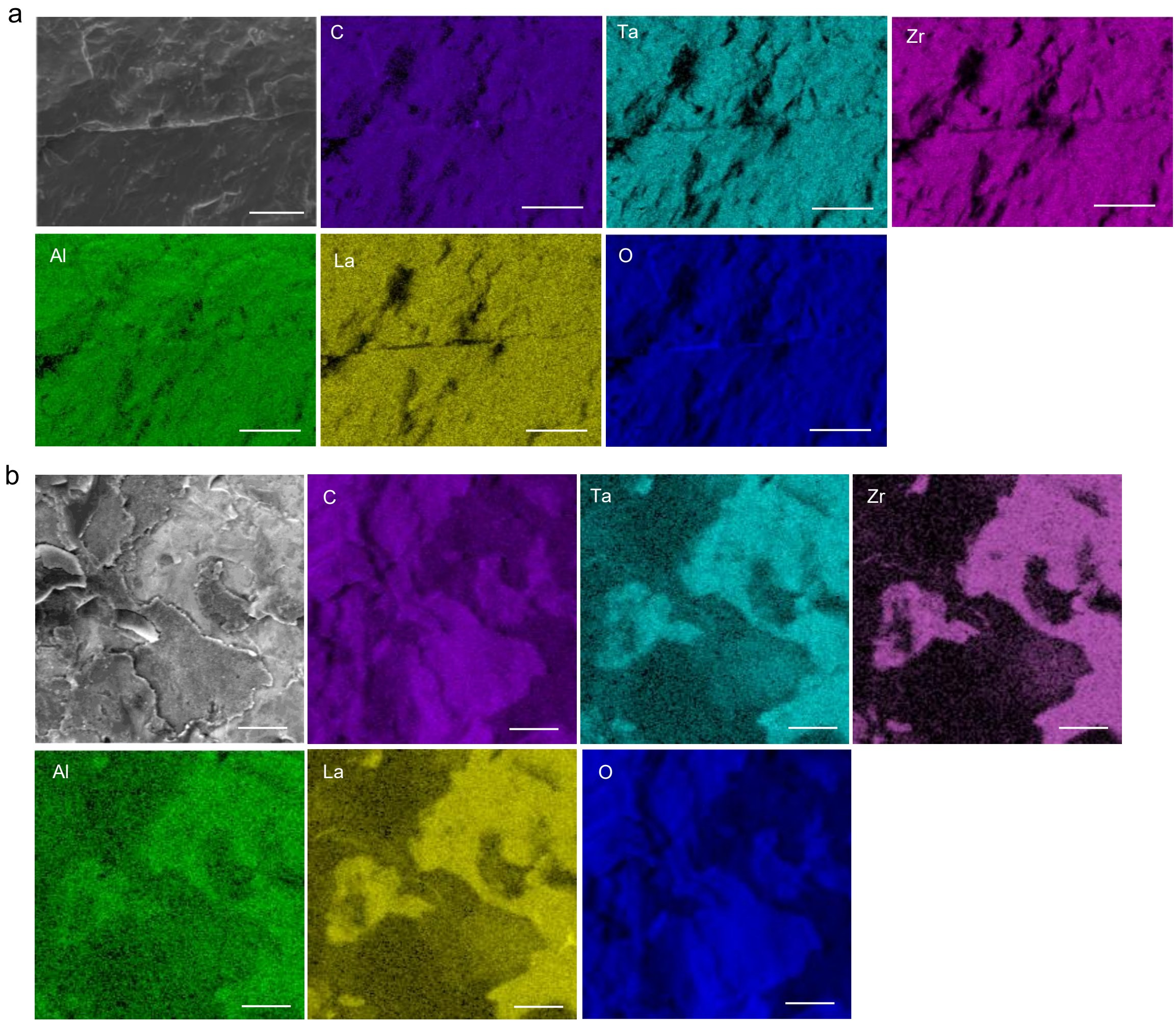

**Supplementary Fig. 9 | Battery performance. a**,**b**, Rate performance of full cells incorporating SF-LLZO (**a**) and S-LLZO (**b**) pellets tested at 25 ℃. Full cell constructed with high-voltage $LiCoO_2$ as the positive electrode (areal density: 10.7 mg cm$^{-2}$, loading: 96.4%), LLZO pellet as the separator, and Li metal as the negative electrode. A liquid electrolyte (1.0 M $LiPF_6$ in EC:DMC:EMC = 1:1:1 by volume with 1.0% VC) was applied at the positive electrode-separator interface to ensure proper contact. The voltage window was set from 3 to 4.45 V, with a 1C rate defined as 170 mA g$^{-1}$. **e**, Long-term cycling performance of SF-LLZO pellets at 0.1 C (3–4.45 V) and 1 C (3–4.3 V) at 25 ℃. The batteries were tested in coin cells without applying additional pressure, and the cells was pressed by the spring component.

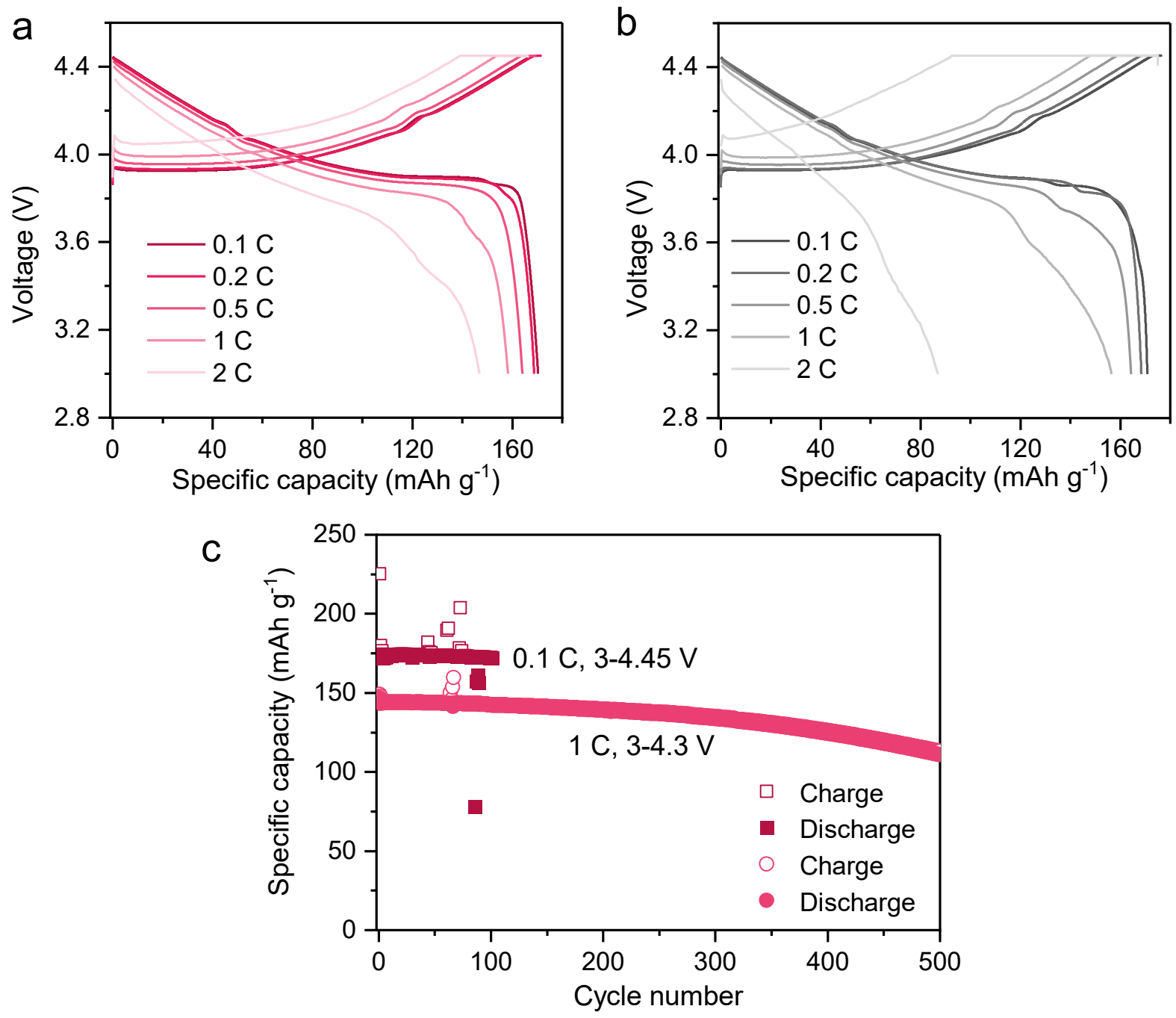

**Supplementary Fig. 10 | MLFF validation.** Comparison of energy (top left) and force components (top right) between DFT calculations and predictions by the ML potential for Al-doped LLZO ($R^2$ values are also shown), and the energy error (bottom left) and force RMSE (bottom right) as a function of simulation time for MD snapshots at 2000 K for (**a**,**c**,**e**,**g**) cubic and (**b**,**d**,**f**,**h**) amorphous $[Li_{7-3x}Al_x]La_3Zr_2O_{12}$ with $x$ = 0, 0.25, 0.5, and 0.75, respectively, with a unit cell for each system used for training. The error is defined as the difference in energy or force component values between ML prediction and DFT calculation. The force RMSE of each snapshot (i.e., at each time) was calculated using errors of 3N force components (where N is the number of atoms). MD snapshots were sampled every 1 ps. The black solid line is a guideline, and the red line shows the position of RMSE for 100 ps. Comparison of energy (top left) and force components (top right) between DFT calculations and predictions by the ML potential for Ta-doped LLZO ($R^2$ values are also shown), and the energy error (bottom left) and force RMSE (bottom right) as a function of simulation time for MD snapshots at 2000 K for (**i**,**j**,**k**,**l**) cubic and (**m**,**n**,**o**,**p**) amorphous $Li_{7-x}La_3[Zr_{2-x}Ta_x]O_{12}$ with $x$ = 0, 0.25, 0.5, and 0.75, respectively, with a unit cell for each system used for training. The error is defined as the difference in energy or force component values between ML prediction and DFT calculation. The force RMSE of each snapshot (i.e., at each time) was calculated using errors of 3N force components (where N is the number of atoms). MD snapshots were sampled every 1 ps. The black solid line is a guideline, and the red line shows the position of RMSE for 100 ps.

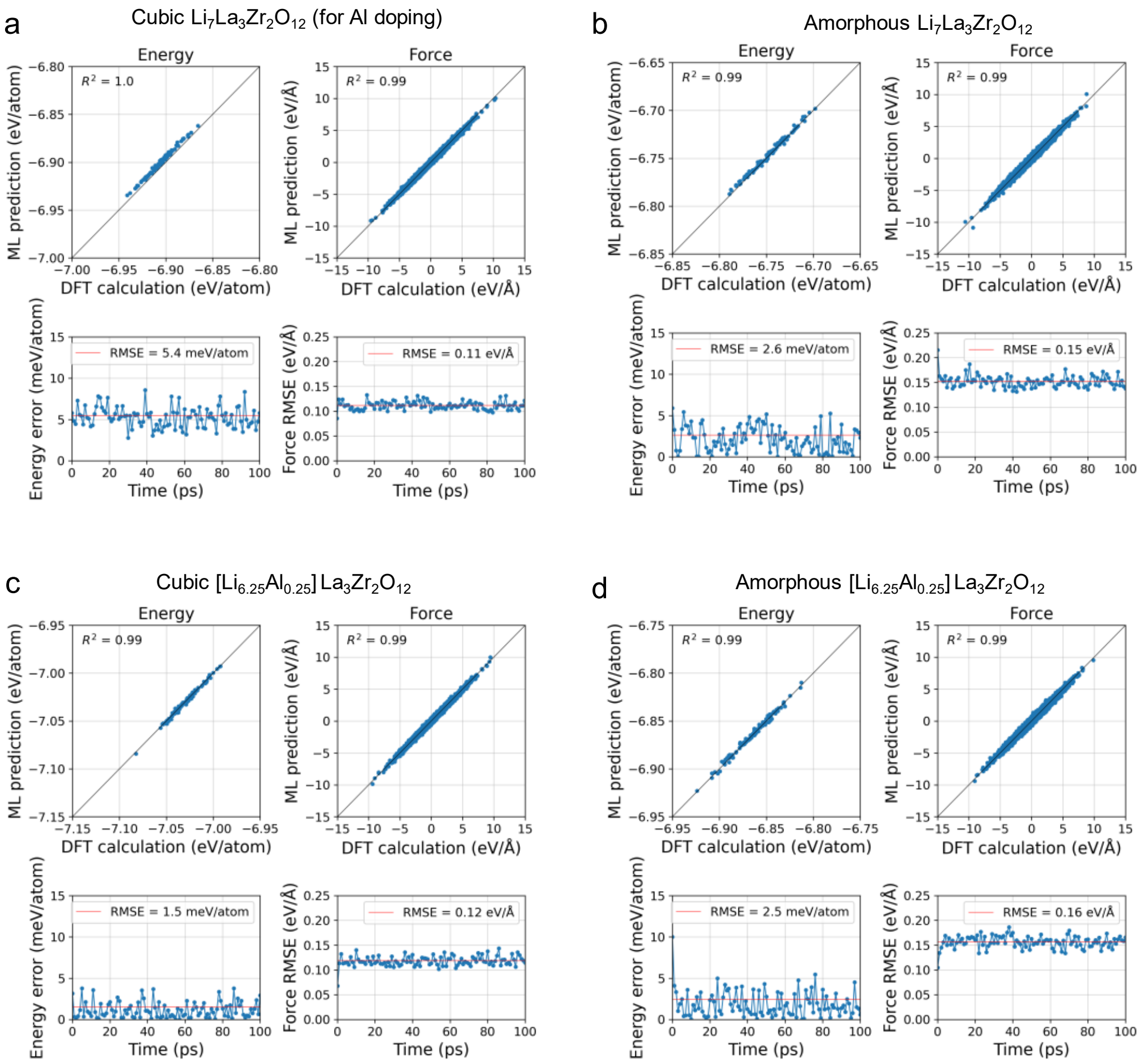

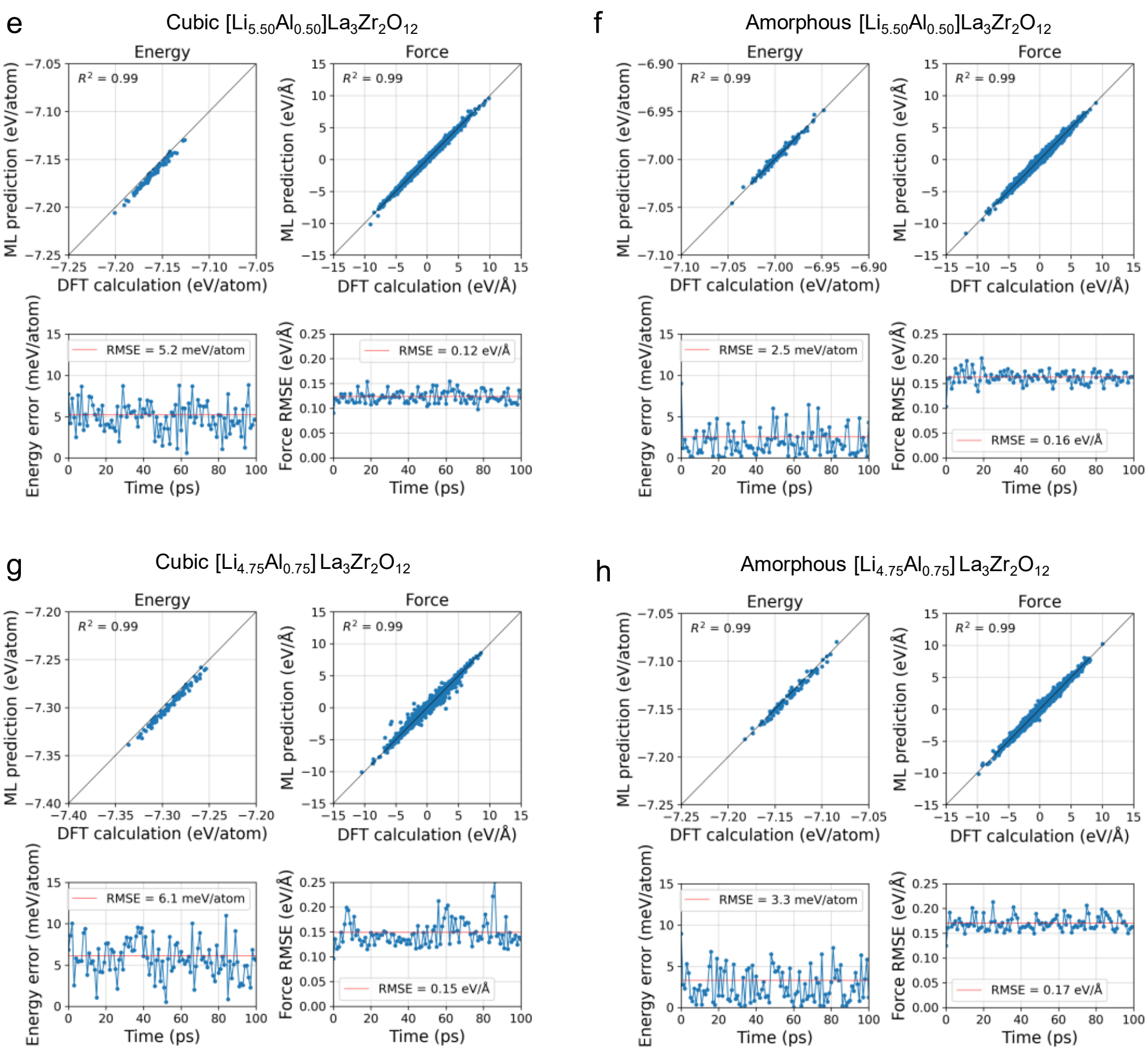
e
Cubic [Li5.50Al0.50]La3Zr2O12
Energy
Force
R2 = 0.99
ML prediction (eV/atom)
DFT calculation (eV/atom)
ML prediction (eV/Å)
DFT calculation (eV/Å)
RMSE = 5.2 meV/atom
RMSE = 0.12 eV/Å
Energy error (meV/atom)
Force RMSE (eV/Å)
Time (ps)
f
Amorphous [Li5.50Al0.50]La3Zr2O12
RMSE = 2.5 meV/atom
RMSE = 0.16 eV/Å
g
Cubic [Li4.75Al0.75] La3Zr2O12
RMSE = 6.1 meV/atom
RMSE = 0.15 eV/Å
h
Amorphous [Li4.75Al0.75] La3Zr2O12
RMSE = 3.3 meV/atom
RMSE = 0.17 eV/Å

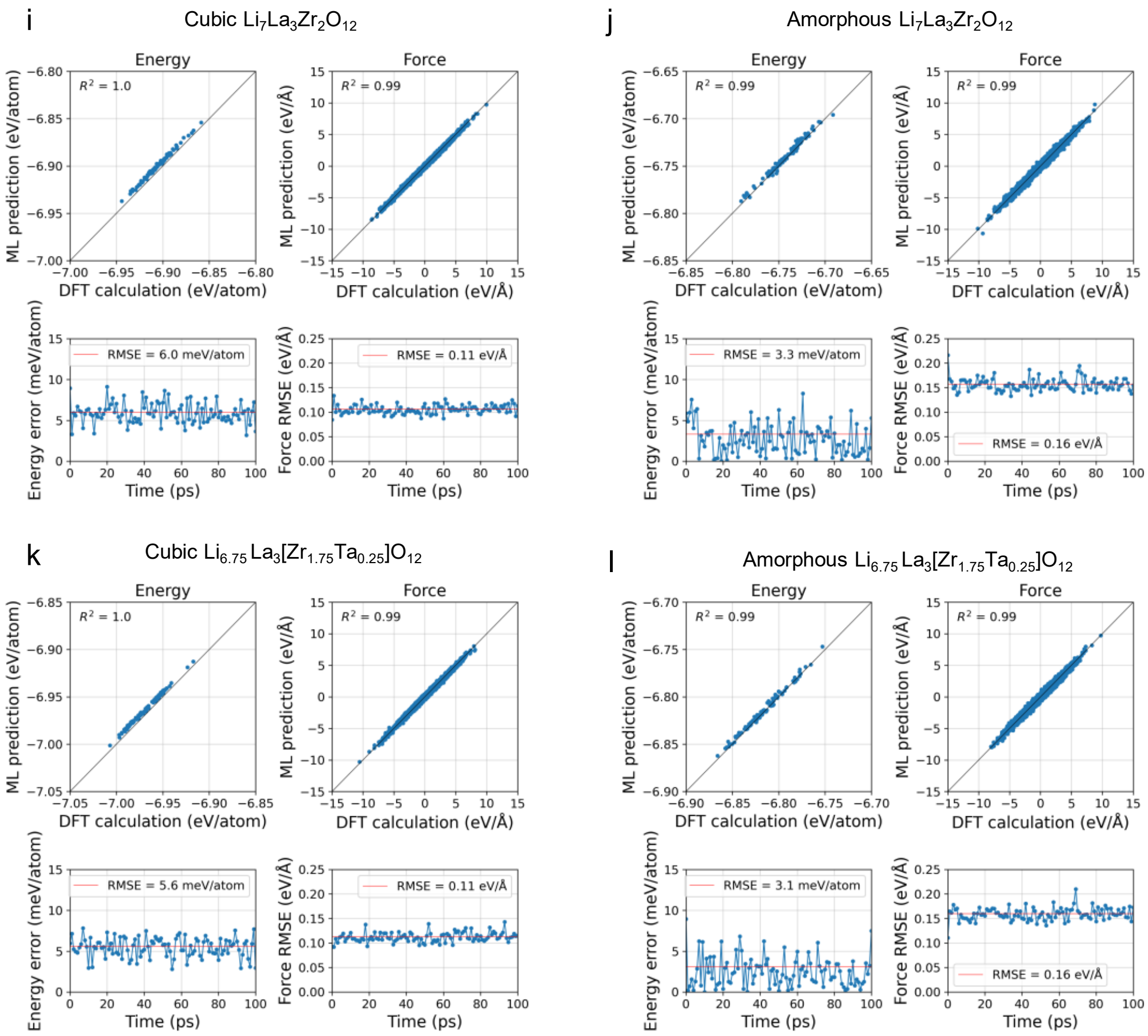
i
Cubic Li7La3Zr2O12
Energy
R² = 1.0
ML prediction (eV/atom)
DFT calculation (eV/atom)
Force
R² = 0.99
ML prediction (eV/Å)
DFT calculation (eV/Å)
RMSE = 6.0 meV/atom
Energy error (meV/atom)
Time (ps)
RMSE = 0.11 eV/Å
Force RMSE (eV/Å)
Time (ps)
j
Amorphous Li7La3Zr2O12
Energy
R² = 0.99
ML prediction (eV/atom)
DFT calculation (eV/atom)
Force
R² = 0.99
ML prediction (eV/Å)
DFT calculation (eV/Å)
RMSE = 3.3 meV/atom
Energy error (meV/atom)
Time (ps)
RMSE = 0.16 eV/Å
Force RMSE (eV/Å)
Time (ps)
k
Cubic Li6.75La3[Zr1.75Ta0.25]O12
Energy
R² = 1.0
ML prediction (eV/atom)
DFT calculation (eV/atom)
Force
R² = 0.99
ML prediction (eV/Å)
DFT calculation (eV/Å)
RMSE = 5.6 meV/atom
Energy error (meV/atom)
Time (ps)
RMSE = 0.11 eV/Å
Force RMSE (eV/Å)
Time (ps)
l
Amorphous Li6.75La3[Zr1.75Ta0.25]O12
Energy
R² = 0.99
ML prediction (eV/atom)
DFT calculation (eV/atom)
Force
R² = 0.99
ML prediction (eV/Å)
DFT calculation (eV/Å)
RMSE = 3.1 meV/atom
Energy error (meV/atom)
Time (ps)
RMSE = 0.16 eV/Å
Force RMSE (eV/Å)
Time (ps)

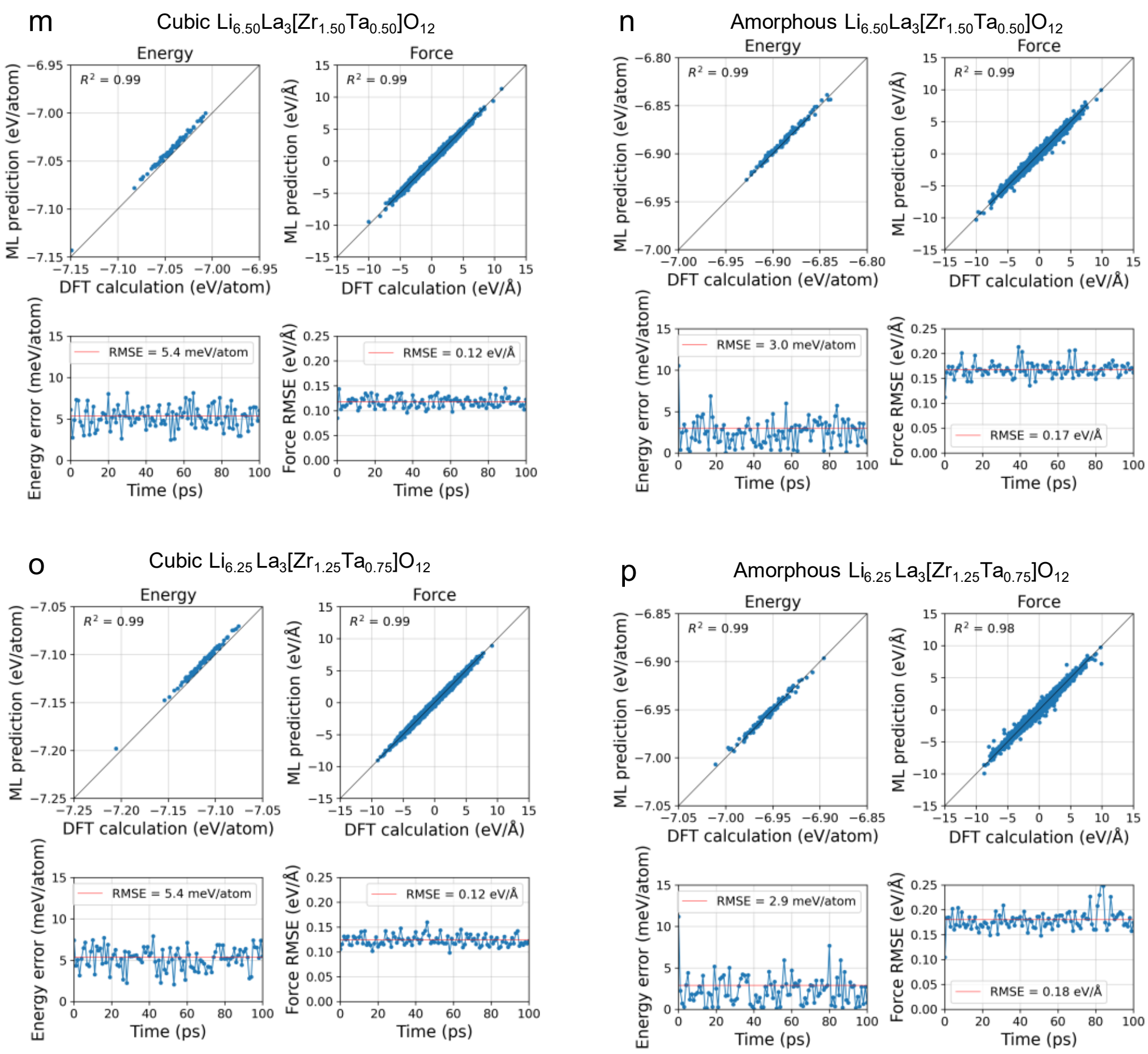
m
Cubic Li6.50La3[Zr1.50Ta0.50]O12
Energy
Force
R2 = 0.99
ML prediction (eV/atom)
DFT calculation (eV/atom)
ML prediction (eV/Å)
DFT calculation (eV/Å)
RMSE = 5.4 meV/atom
RMSE = 0.12 eV/Å
Energy error (meV/atom)
Force RMSE (eV/Å)
Time (ps)
n
Amorphous Li6.50La3[Zr1.50Ta0.50]O12
Energy
Force
R2 = 0.99
ML prediction (eV/atom)
DFT calculation (eV/atom)
ML prediction (eV/Å)
DFT calculation (eV/Å)
RMSE = 3.0 meV/atom
RMSE = 0.17 eV/Å
Energy error (meV/atom)
Force RMSE (eV/Å)
Time (ps)
o
Cubic Li6.25La3[Zr1.25Ta0.75]O12
Energy
Force
R2 = 0.99
ML prediction (eV/atom)
DFT calculation (eV/atom)
ML prediction (eV/Å)
DFT calculation (eV/Å)
RMSE = 5.4 meV/atom
RMSE = 0.12 eV/Å
Energy error (meV/atom)
Force RMSE (eV/Å)
Time (ps)
p
Amorphous Li6.25La3[Zr1.25Ta0.75]O12
Energy
Force
R2 = 0.99
R2 = 0.98
ML prediction (eV/atom)
DFT calculation (eV/atom)
ML prediction (eV/Å)
DFT calculation (eV/Å)
RMSE = 2.9 meV/atom
RMSE = 0.18 eV/Å
Energy error (meV/atom)
Force RMSE (eV/Å)
Time (ps)

**Supplementary Tab. 1 | Formation energies.** Formation energies of Al, Ta and La in cubic and amorphous LLZO were calculated using elemental metals as reference, with the average energy obtained from 10 random doped structures for Al and Ta and 24 vacancy sites for La.

| | | Cubic | Amorphous | Difference ($\Delta E_{form}$) |
|---|---|---|---|---|
| $E_{form}$ (eV/dopant) | Al | 2.12 | 1.17 | -0.95 |
| | Ta | 2.28 | 2.41 | +0.12 |
| | La | 12.34 | 9.83 | +2.50 |

**Supplementary Tab. 2 | MLFF-MD for Ta and Al migration.** Activation energies of Al, Ta and La migrating in bulk and GB Σ17 (530) [001], Σ33 (52-2) [011] and Σ19 (32-5) [111].

| | Ea of Al (eV) | Ea Ta (eV) | Ea La (eV) |
|---|---|---|---|
| Bulk | 0.645 | 0.619 | 0.248 |
| Σ17 (530) [001] | 0.831 | 0.882 | 0.941 |
| Σ33 (52-2) [011] | 0.974 | 0.873 | 1.007 |
| Σ19 (32-5) [111] | 0.989 | 0.673 | 0.793 |

**Supplementary Tab. 3 | Comparison of conductivity.** Comparison of conductivity as a function of density and sintering methods. CS is conventional sintering, HPS is hot press sintering and SPS is spark plasma sintering. All reported conductivities in the table were measured near room temperature

| Methods | Relative density | Conductivity (mS/cm) | Chemical formula or dopant | Ref. |
|---|---|---|---|---|
| CS | 93 | 0.0567 | Al | 1 |
| CS | 92.9 | 0.246 | $Li_{7.06}La_3Zr_{1.94}Sm_{0.06}O_{12}$ | 2 |
| CS | 92 | 0.08 | $Li_7La_3(ZrNb)O_{12}$ | 3 |
| CS | 96 | 0.31 | $Li_{6.4}La_3Zr_{1.4}Ta_{0.6}O_{12}$ | 4 |
| CS | 94 | 0.467 | $Li_7La_3Zr_{0.5}Nb_{0.5}Ta_{0.5}Hf_{0.5}O_{12}$ | 5 |
| CS | 97.4 | 0.694 | Ta | 6 |
| CS | 92.8 | 0.509 | Mo | 7 |

| | | | | |
|---|---|---|---|---|
| CS | 94.5 | 1.36 | Ga | 8 |
| CS | 94 | 0.3 | Eu | 9 |
| CS | 90 | 0.5 | Ta | 10 |
| CS | 91.9 | 0.23 | Sb | 11 |
| CS | 93.0 | 0.353 | Ta, Sb | 11 |
| CS | 92.7 | 0.5 | $Li_{6.4}La_3Zr_{1.4}Ta_{0.6}O_{12}$ | 11 |
| CS | 92.3 | 0.448 | $Li_{6.4}La_3Zr_{1.4}Nb_{0.3}Ta_{0.3}O_{12}$ | 11 |
| CS | 92.0 | 0.266 | $Li_{6.4}La_3Zr_{1.4}Nb_{0.6}O_{12}$ | 11 |
| CS | 92 | 0.3 | Ta | 12 |
| CS | 94 | 0.522 | Nb | 13 |
| CS | 97 | 0.7 | $Li_{6.5}La_3Zr_{1.5}Nb_{0.5}O_{12}$ | 14 |
| CS | 91.7 | 0.73 | Ta | 15 |
| CS | 92.7 | 0.63 | Ta | 15 |
| CS | 92.5 | 0.69 | Ta | 15 |
| CS | 92.8 | 0.46 | Ta | 15 |
| CS | 92.8 | 0.47 | Ta | 15 |
| CS | 93.6 | 0.40 | Ta | 15 |
| CS | 94.1 | 0.28 | Ta | 15 |
| CS | 95.0 | 0.35 | Ta | 15 |
| CS | 93.8 | 0.034 | Ta | 15 |
| CS | 96 | 0.645 | Ta | 16 |
| CS | 98 | 0.5 | Al | 17 |
| CS | 95 | 0.46 | Al | 18 |
| CS | 94 | 0.2 | Al | 19 |
| CS | 97 | 1.09 | Ta | 20 |
| CS | 92 | 0.1 | Ta | 21 |
| CS | 97 | 0.517 | Ta | 22 |
| CS | 92.1 | 0.33 | Al | 23 |
| CS | 92.9 | 0.82 | Fe | 23 |
| CS | 94.4 | 1.31 | Ga | 23 |
| CS | 94.1 | 0.668 | Si | 24 |
| CS | 97.54 | 0.618 | $Li_{6.4}La_3Zr_{1.4}Ta_{0.6}O_{12}$ | 25 |
| CS | 94 | 1.2 | $Li_{6.4}Ga_{0.2}La_3Zr_2O_{12}$ | 26 |
| CS | 97 | 0.6 | Nb | 27 |
| CS | 96 | 0.448 | Al | 28 |
| CS | 96 | 0.647 | $Li_{6.4}La_3Zr_{1.4}Ta_{0.6}O_{12}$ | 29 |
| CS | 97.5 | 1.24 | $Li_{6.4}Ga_{0.2}La_3Zr_2O_{12}$ | 30 |
| CS | 95.4 | 1.09 | $Li_{6.4}Ga_{0.2}La_3Zr_2O_{12}$ | 31 |
| CS | 98 | 1 | $Li_{6.4}La_3Zr_{1.4}Ta_{0.6}O_{12}$ | 32 |
| CS | 97 | 0.85 | $Li_{6.5}La_3Zr_{1.5}Ta_{0.5}O_{12}$ | 33 |

| | | | | |
|---|---|---|---|---|
| CS | 94.25 | 0.45 | Al | 34 |
| CS | 96.2 | 0.853 | Ta | 35 |
| CS | 90 | 0.36 | $Li_{6.8}La_{2.95}Ca_{0.05}Zr_{1.75}Nb_{0.25}O_{12}$ | 36 |
| CS | 93.4 | 0.87 | Ga | 37 |
| CS | 94.1 | 1.46 | Ga | 37 |
| CS | 96.3 | 1.12 | Ga | 37 |
| CS | 95.1 | 0.71 | Ga | 37 |
| CS | 92.8 | 0.57 | Ga | 37 |
| HPS | 98 | 0.34 | $Li_{6.19}Al_{0.27}La_3Zr_2O_{12}$ | 38 |
| HPS | 98 | 1.2–1.75 | Ga | 39 |
| HPS | 97 | 0.46 | $Li_{6.25}La_3Zr_2Al_{0.25}O_{12}$ | 40 |
| HPS | 91 | 0.35 | $Li_{6.25}La_3Zr_2Ga_{0.25}O_{12}$ | 41 |
| HPS | 99 | 0.37 | $Li_{5.95}La_3Zr_2Al_{0.35}O_{12}$ | 42 |
| HPS | 97.5 | 1.13 | $Li_{6.55}Ga_{0.15}La_3Zr_2O_{12}$ | 43 |
| HPS | 97 | 0.5 | $Li_{6.25}Al_{0.25}La_3Zr_2O_{12}$ | 44 |
| HPS | 98 | 0.52 | $Li_{6.25}Al_{0.25}La_3Zr_2O_{12}$ | 45 |
| HPS | 98 | 0.4 | $Li_{6.28}Al_{0.24}La_3Zr_2O_{12}$ | 46 |
| HPS | 99.7 | 0.61 | Ta | 47 |
| HPS | 99 | 0.74 | $Li_7La_3Zr_{1.5}Nb_{0.5}O_{12}$ | 48 |
| HPS | 99.4 | 0.57 | $Li_{6.25}Al_{0.25}La_3Zr_2O_{12}$ | 49 |
| HPS | 99 | 0.84 | $Li_{6.6}La_3Zr_{1.6}Ta_{0.4}O_{12}$ | 50 |
| HPS | 97 | 0.4 | Ta | 51 |
| HPS | 97.3 | 1.3 | Ga | 52 |
| HPS | 99.5 | 0.66 | Ta | 53 |
| SPS | 99.8 | 0.57 | Al | 54 |
| SPS | 99 | 0.31 | $Li_{6.25}Al_{0.25}La_3Zr_2O_{12}$ | 55 |
| SPS | 95 | 0.643 | $Li_{6.65}La_{2.95}Sr_{0.05}Zr_{1.8}Mo_{0.2}O_{12}$ | 56 |
| SPS | 99.8 | 0.651 | Al | 57 |
| SPS | 93 | 0.33 | Al | 58 |
| SPS | 95.5 | 0.69 | Ta | 59 |
| SPS | 99 | 1.1 | $Li_{6.6}La_3Zr_{1.6}Ta_{0.4}O_{12}$ | 60 |
| SPS | 96 | 0.917 | $Li_{6.4}La_3Zr_{1.4}Ta_{0.6}O_{12}$ | 61 |
| SPS | 99.2 | 0.48 | $Li_{6.75}La_3Al_{0.25}Zr_2O_{12}$ | 62 |
| SPS | 96 | 0.16 | Al | 63 |

**Supplementary Tab. 4 | Quantitative statistical analysis of excess elements.** Grain boundary excess values (in atoms/nm$^2$) and measurement detection limits for S-LLZO and SF-LLZO, measured by STEM/EDS. The aluminum concentration was too low to be quantitatively analyzed with EDS. The three box method has been used to quantify the grain boundary segregation in each sample[64-65]. More than 20 GBs were analyzed for the SF-LLZO, and five for the S-LLZO. The average GB excess values for each element are shown in the table, along with the detection limit of the method for each element. The standard error of the mean was used to calculate errors for the GB excess values.

| Materials | Element | GB excess | Detection limit |
|---|---|---|---|
| S-LLZO | La | -8.0 ± 9.3 | 0.4 |
| | Ta | 2.5 ± 0.5 | 0.2 |
| | Zr | -2.3 ± 0.4 | 0.1 |
| SF-LLZO | La | 0.4 ± 0.2 | 0.1 |
| | Ta | 0.3 ± 0.2 | 0.1 |
| | Zr | 0.2 ± 0.1 | 0.05 |

**Supplementary Tab. 5 | Impedance fitting results.** Equivalent circuit diagram and fitting results and errors for impedance fitting.

| Samples | Equivalent circuits | Elements | Fit values | Fit errors |
|---|---|---|---|---|
| S-LLZO | 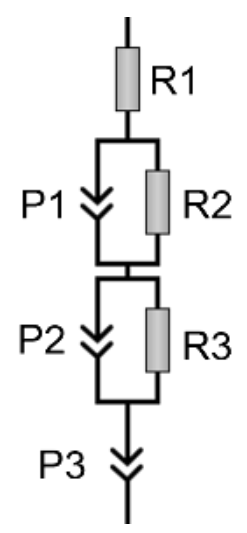  | R1 | 52.1 | 8.8% |
| | | R2 | 471 | 1.5% |
| | | CPE Q1 | 8.05E-11 | 15% |
| | | CPE Alpha1 | 1 | 0.96% |
| | | R3 | 86.2 | 14% |
| | | CPE Q2 | 3.64E-8 | 20% |
| | | CPE Alpha2 | 0.958 | 6.7% |
| | | CPE Q3 | 3.99E-6 | 13% |
| | | CPE Alpha3 | 0.634 | 2.2% |
| SF-LLZO | 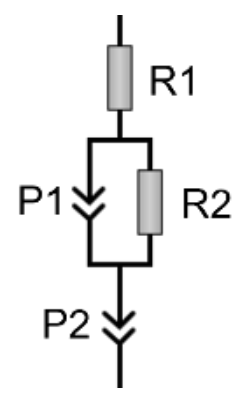  | R1 | 25.5 | 5.4% |
| | | R2 | 537 | 2.6% |
| | | CPE Q1 | 4.57E-11 | 13% |
| | | CPE Alpha1 | 0.984 | 1% |
| | | CPE Q2 | 3.91E-6 | 2.5% |
| | | CPE Alpha2 | 0.735 | 0.4% |
| | | R1 | 35.3 | 13% |

| | | | | |
|---|---|---|---|---|
| Annealed SF-LLZO | 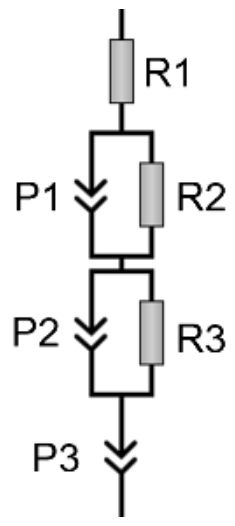 | R2 | 447 | 1.6% |
| | | CPE Q1 | 8.7E-11 | 15% |
| | | CPE Alpha1 | 0.994 | 0.97% |
| | | R3 | 104 | 4.5% |
| | | CPE Q2 | 1.98E-8 | 38% |
| | | CPE Alpha2 | 0.977 | 3.2% |
| | | CPE Q3 | 1.26E-6 | 2.2% |
| | | CPE Alpha3 | 0.782 | 0.33% |

**Supplementary Tab. 6 | Comparison of activation energies.** Comparison of activation energies of Li migrating in bulk and GBs with and without Ta segregation.

| | No segregation (eV) | Ta segregation (eV) |
|---|---|---|
| Bulk | 0.23 | |
| Σ17 (530) [001] | 0.32 | 0.33 |
| Σ33 (52-2) [011] | 0.37 | 0.35 |
| Σ19 (32-5) [111] | 0.32 | 0.35 |

**Supplementary Tab. 7 | Continuum simulation parameters.**

| Parameter | Value |
|---|---|
| Li bulk concentration | 6.45 per unit cell |
| σ | $7.3*10^{-4}$ S/cm |
| $\varepsilon_r$ | 120[66] |
| GB width | 1.6 nm |

**Supplementary Tab. 8 | Comparison of stripping/plating capacities.** Comparison of reported CCDs and CCD capacities of LLZOs, as well as total accumulated capacities during galvanostatic cycling. The CCD performance compared in Fig. 5c of manuscript was measured at near room temperature.

| Materials | CCD ($mA\ cm^{-2}$) | CCD test time (h) | Accumulated cycling capacities ($mAh\ g^{-1}$) | Modifications | Tem. (℃) | Ref. |
|---|---|---|---|---|---|---|
| $Li_{6.76}Al_{0.24}La_3Zr_2O_{12}$ | 0.35 | 0.5 | ~6.25 | | 25 | 67 |
| $Li_{6.45}Al_{0.05}La_3Zr_{1.6}Ta_{0.4}O_{12}$ | 0.3185 | 0.5 | | | 50 | 68 |
| $Li_{6.25}La_3Zr_2Al_{0.25}O_{12}$ | 0.93 | 0.215 | | | 25 | 69 |
| $Li_{6.25}La_3Zr_2Al_{0.25}O_{12}$ | 0.05 | 0.3 | | | 30 | 40 |
| Ta-doped LLZO | 0.44 | 0.5 | | | RT | 70 |
| $Li_{6.1}Ga_{0.3}La_3Zr_2O_{12}$ | 0.32 | 0.6 | 3 | | 25 | 71 |
| $Li_{6.5}La_3Zr_{1.5}Ta_{0.5}O_{12}$ | 0.5 | 0.5 | ~25 | | RT | 72 |
| $Li_{6.5}La_3Zr_{1.5}Ta_{0.5}O_{12}$ | 0.8 | 0.3125 | | | RT | 73 |
| $Li_{6.5}La_3Zr_{1.5}Ta_{0.5}O_{12}$ | 0.9 | 0.222 | | | RT | 74 |
| $Li_{6.5}La_3Zr_{1.5}Ta_{0.5}O_{12}$ | 6.0 | 0.0333 | 702 | | 60 | 74 |
| Al-doped LLZO | 0.134 | 0.5 | | | RT | 75 |
| $Li_{6.5}La_3Zr_{1.5}Ta_{0.5}O_{12}$ | 0.1 | 0.5 | | | 25 | 76 |
| $Li_{6.5}La_3Zr_{1.5}Ta_{0.5}O_{12}$ | 0.6 | 0.667 | | | 25 | 77 |
| $Li_{6.25}Al_{0.25}La_3Zr_2O_{12}$ | 0.6 | 0.5 | | | RT | 49 |
| Al-doped LLZO | 0.62 | 0.1 | | | RT | 78 |
| | | | ~260 | | 70 | 78 |
| $Li_{6.4}Ga_{0.2}La_3Zr_2O_{12}$ | 0.28 | 0.333 | | | 20 | 79 |
| $Li_{6.5}La_3Zr_{1.5}Ta_{0.5}O_{12}$ | 0.4 | 0.5 | ~7.5 | | 25 | 80 |
| $Li_{6.25}Ga_{0.25}La_3Zr_2O_{12}$ | 0.55 | 0.5 | | | | 81 |
| $Li_{6.1}Ga_{0.3}La_3Zr_2O_{12}$ | 0.58 | 0.4 | 6 | | RT | 82 |
| Al-doped LLZO | 12.5 | 0.08 | 125 | | 75 | 83 |
| Ta-doped LLZO | 1.15 | 0.5 | ~56.25 | | 25 | 29 |
| Al-doped LLZO | 0.64 | 0.156 | ~28.75 | | RT | 84 |
| $Li_{6.25}Ga_{0.25}La_3Zr_2O_{12}$ | 0.7 | 0.5 | 100 | | RT | 85 |
| Ta-doped LLZO | 0.1 | 0.5 | 15 | | RT | 86 |
| $Li_{6.6}La_3Zr_{1.6}Ta_{0.4}O_{12}$ | 0.7 | 0.5 | 6 | | RT | 87 |
| Ta-doped LLZO | 1.1 | 0.18 | 150.4 | GB engineering (LiF) | RT | 88 |
| $Li_{6.1}Ga_{0.3}La_3Zr_2O_{12}$ | | | 15 | GB engineering ($Li_2O$-$B_2O_3$-$Al_2O_3$) | RT | 89 |
| $Li_{6.4}La_3Zr_{1.4}Ta_{0.6}O_{12}$ | 3.2 | 0.03125 | | GB engineering ($ZrO_2$) and surface modification (W) | RT | 90 |
| $Li_{5.9}Ga_{0.2}La_3Zr_{1.5}Ta_{0.5}O_{12}$ | 0.5 | 0.5 | 100 | GB engineering ($LiGaO_2$) | RT | 91 |
| Ta-doped LLZO | 1.1 | 0.5 | 300 | GB engineering ($Li_3BO_3$) | RT | 92 |
| $Li_{6.5}La_3Zr_{1.5}Ta_{0.5}O_{12}$ | 2.12 | 0.5 | 75 | GB engineering ($La_2O_3$) and surface modification (Au) | RT | 93 |

| | | | | | | |
|---|---|---|---|---|---|---|
| $Li_{6.4}La_3Zr_{1.4}Ta_{0.6}O_{12}$ | 0.9 | 0.5 | 75 | GB engineering ($Li_2O$) and surface modification (Au) | RT | 32 |
| Ta-doped LLZO | 1.1 | 0.25 | 75 | GB engineering ($TiO_2$) and surface modification (Au) | RT | 94 |
| $Li_{6.5}La_3Zr_{1.5}Ta_{0.5}O_{12}$ | 1.4 | 0.5 | 300 | GB engineering ($Li_6Zr_2O_7$) and surface modification (Au) | RT | 95 |
| $Li_{6.5}La_3Zr_{1.5}Ta_{0.5}O_{12}$ | 0.6 | 0.62 | | GB engineering ($Li_2O$) | RT | 77 |
| F-doped LLZO | 1.8 | 0.125 | 130 | GB engineering ($LiAlO_2$) | RT | 96 |
| $Li_{6.5}La_3Zr_{1.6}Ta_{0.4}O_{12}$ | 0.45 | 0.125 | 50 | GB engineering ($LiAlO_2$) | 30 | 97 |
| $Li_{6.4}La_3Zr_{1.4-x}Mn_xTa_{0.6}O_{12}$ | 0.3 | 1 | | GB engineering ($La_4LiMnO_8$) | RT | 98 |
| $Li_{6.5}La_3Zr_{1.5}Ta_{0.5}O_{12}$ | 1.8 | 0.5 | 300 | GB engineering ($Li_2TiO_3$) and surface modification (Au) | RT | 80 |
| Ta-doped LLZO | 1.5 | 0.167 | 150 | GB engineering ($LiGaO_2$) | 30 | 99 |
| $Li_{6.25}Al_{0.25}La_3Zr_2O_{12}$ | 0.1 | 0.5 | 21 | GB engineering (LiF, $Li_xAlO_y$) | RT | 100 |
| $Li_{6.4}La_3Zr_{1.4}Ta_{0.6}O_{12}$ | 2.7 | 0.037 | 300 | GB engineering (Li/$BaTiO_3$) | RT | 101 |

**Supplementary Tab. 9 | MLFF validation.** Energy (meV/atom) and force (eV/Å) errors of snapshots from MLMD simulations at 2000 K versus DFT. "Unseen" indicates that the ML model did not learn the systems. The doping level is indicated by the stoichiometry x in $[Li_{7-3x}Al_x]La_3Zr_2O_{12}$ and $Li_{7-x}La_3[Zr_{2-x}Ta_x]O_{12}$. Values in parenthesis are $R^2$ (see Supplementary Fig. 8 for detailed data).

| System type | | Energy | Force |
|---|---|---|---|
| Al-LLZO Cubic | *x* = 0 | 5.4 (1.0) | 0.11 (0.99) |
| | *x* = 0.25 | 1.5 (0.99) | 0.12 (0.99) |
| | *x* = 0.50 | 5.2 (0.99) | 0.12 (0.99) |
| | *x* = 0.75 | 6.1 (0.99) | 0.15 (0.99) |
| Al-LLZO Amorphous | *x* = 0 | 2.6 (0.99) | 0.15 (0.99) |
| | *x* = 0.25 | 2.5 (0.99) | 0.16 (0.99) |
| | *x* = 0.50 | 2.5 (0.99) | 0.16 (0.99) |
| | *x* = 0.75 | 3.3 (0.99) | 0.17 (0.99) |
| Ta-LLZO Cubic | *x* = 0 | 6.0 (1.0) | 0.11 (0.99) |
| | *x* = 0.25 | 5.6 (1.0) | 0.11 (0.99) |
| | *x* = 0.50 | 5.4 (0.99) | 0.12 (0.99) |
| | *x* = 0.75 | 5.4 (0.99) | 0.12 (0.99) |
| Ta-LLZO Amorphous | *x* = 0 | 3.3 (0.99) | 0.16 (0.99) |
| | *x* = 0.25 | 3.1 (0.99) | 0.16 (0.99) |
| | *x* = 0.50 | 3.0 (0.99) | 0.17 (0.99) |
| | *x* = 0.75 | 2.9 (0.99) | 0.18 (0.98) |

**References**


1. Zhao, P. *et al.* A novel method for preparation of high dense tetragonal $Li_7La_3Zr_2O_{12}$. *J. Power Sources* **344**, 56-61 (2017).
2. Wang, X. *et al.* High lithium ionic conductivity of garnet-type oxide $Li_{7+x}La_3Zr_{2-x}Sm_xO_{12}$ (x = 0-0.1) ceramics. *Mater. Lett.* **231**, 43-46 (2018).
3. Rosero-Navarro, N. C., Yamashita, T., Miura, A., Higuchi, M. & Tadanaga, K. Effect of Sintering Additives on Relative Density and Li-ion Conductivity of Nb-Doped $Li_7La_3ZrO_{12}$ Solid Electrolyte. *J. Am. Ceram. Soc.* **100**, 276-285 (2017).
4. Zhang, K. *et al.* Unveiling the roles of alumina as a sintering aid in Li-Garnet solid electrolyte. *Int. J. Energy Res.* **44**, 9177-9184 (2020).
5. Fu, Z. & Ferguson, J. Processing and characterization of an $Li_7La_3Zr_{0.5}Nb_{0.5}Ta_{0.5}Hf_{0.5}O_{12}$ high-entropy Li-garnet electrolyte. *J. Am. Ceram. Soc.* **105**, 6175-6183 (2022).
6. Zheng, H., Li, G., Liu, H., Wu, Y. & Duan, H. Influence of dry- and wet-milled LLZTO particles on the sintered pellets. *J. Am. Ceram. Soc.* **106**, 274-284 (2023).
7. Liu, X. *et al.* High lithium ionic conductivity in the garnet-type oxide $Li_{7-2x}La_3Zr_{2-x}Mo_xO_{12}$ (x=0-0.3) ceramics by sol-gel method. *J. Am. Ceram. Soc.* **100**, 1527-1533 (2017).
8. Xiang, X. *et al.* Effect of $Li_2O$ excess in $Li_{6.4}Ga_{0.2}La_3Zr_2O_{12}$ electrolyte for all-solid-state Li-ion batteries. *Int. J. Appl. Ceram. Technol.* **20**, 321-328 (2023).
9. Salimkhani, H., Erdem, E., Alkan Gursel, S. & Yurum, A. Unveiling the presence of mixed oxidation states of Europium in $Li_{7+\delta}Eu_xLa_{3-\delta}Zr_{2-\delta}O_{12-\delta}$ garnet and its impact on the Li-ion conductivity. *J. Am. Ceram. Soc.* **104**, 4257-4271 (2021).
10. Weller, J. M., Dopilka, A. & Chan, C. K. Observation of Elemental Inhomogeneity and Its Impact on Ionic Conductivity in Li-Conducting Garnets Prepared with Different Synthesis Methods. *Adv. Energy Sust. Res.* **2**, 2000109 (2021).
11. Xiang, X. *et al.* Dual regulation of $Li^+$ migration of $Li_{6.4}La_3Zr_{1.4}M_{0.6}O_{12}$ (M = Sb, Ta, Nb) by bottleneck size and bond length of M-O. *J. Am. Ceram. Soc.* **103**, 2483-2490 (2020).
12. Murugan, R., Thangadurai, V. & Weppner, W. Fast Lithium Ion Conduction in Garnet-Type $Li_7La_3Zr_2O_{12}$. *Angew. Chem. Int. Ed.* **46**, 7778-7781 (2007).
13. Zhao, P. *et al.* Garnet-like $Li_{7-x}La_3Zr_{2-x}Nb_xO_{12}$ (x = 0-0.7) solid state electrolytes enhanced by self-consolidation strategy. *J. Eur. Ceram. Soc.* **38**, 5454-5462 (2018).
14. Lu, W., Wang, T., Xue, M. & Zhang, C. Improved $Li_{6.5}La_3Zr_{1.5}Nb_{0.5}O_{12}$ electrolyte and effects of atmosphere exposure on conductivities. *J. Power Sources* **497**, 229845 (2021).
15. Wang, Y. & Lai, W. High Ionic Conductivity Lithium Garnet Oxides of $Li_{7-x}La_3Zr_{2-x}Ta_xO_{12}$ Compositions. *Electrochem. Solid State Lett.* **15**, A68 (2012).
16. Xia, W. *et al.* Reaction mechanisms of lithium garnet pellets in ambient air: The effect of humidity and $CO_2$. *J. Am. Ceram. Soc.* **100**, 2832-2839 (2017).
17. Dubey, R. *et al.* Building a Better Li-Garnet Solid Electrolyte/Metallic Li Interface with Antimony. *Adv. Energy Mater.* **11**, 2102086 (2021).
18. Krauskopf, T., Mogwitz, B., Rosenbach, C., Zeier, W. G. & Janek, J. Diffusion Limitation of Lithium Metal and Li-Mg Alloy Anodes on LLZO Type Solid Electrolytes as a Function of Temperature and Pressure. *Adv. Energy Mater.* **9**, 1902568 (2019).
19. Yi, E., Wang, W., Kieffer, J. & Laine, R. M. Flame made nanoparticles permit processing

of dense, flexible, $Li^+$ conducting ceramic electrolyte thin films of cubic-$Li_7La_3Zr_2O_{12}$ (c-LLZO). *J. Mater. Chem. A* **4**, 12947-12954 (2016).

20. Lu, Y. et al. An in situ element permeation constructed high endurance Li-LLZO interface at high current densities. *J. Mater. Chem. A* **6**, 18853-18858 (2018).
21. Shen, F., Dixit, M. B., Xiao, X. & Hatzell, K. B. Effect of Pore Connectivity on Li Dendrite Propagation within LLZO Electrolytes Observed with Synchrotron X-ray Tomography. *ACS Energy Lett.* **3**, 1056-1061 (2018).
22. Huang, X. *et al.* Preparation of dense Ta-LLZO/MgO composite Li-ion solid electrolyte: Sintering, microstructure, performance and the role of MgO. *J. Energy Chem.* **39**, 8-16 (2019).
23. Xiang, X. *et al.* Crystal structure and lithium ionic transport behavior of Li site doped $Li_7La_3Zr_2O_{12}$. *J. Eur. Ceram. Soc.* **40**, 3065-3071 (2020).
24. Ma, X. *et al.* Garnet Si-$Li_7La_3Zr_2O_{12}$ electrolyte with a durable, low resistance interface layer for all-solid-state lithium metal batteries. *J. Power Sources* **453**, 227881 (2020).
25. Shen, F. *et al.* A Simple and Highly Efficient Method toward High-Density Garnet-Type LLZTO Solid-State Electrolyte. *ACS Appl. Mater. Interfaces* **12**, 30313-30319 (2020).
26. Huang, X. *et al.* Synthesis of Ga-doped $Li_7La_3Zr_2O_{12}$ solid electrolyte with high $Li^+$ ion conductivity. *Ceram. Int.* **47**, 2123-2130 (2021).
27. Huang, X., Song, Z., Xiu, T., Badding, M. E. & Wen, Z. Sintering, micro-structure and $Li^+$ conductivity of $Li_{7-x}La_3Zr_{2-x}Nb_xO_{12}$/MgO (x = 0.2-0.7) Li-Garnet composite ceramics. *Ceram. Int.* **45**, 56-63 (2019).
28. Xia, W. *et al.* Ionic Conductivity and Air Stability of Al-Doped $Li_7La_3Zr_2O_{12}$ Sintered in Alumina and Pt Crucibles. *ACS Appl. Mater. Interfaces* **8**, 5335-5342 (2016).
29. Huang, X. *et al.* None-Mother-Powder Method to Prepare Dense Li-Garnet Solid Electrolytes with High Critical Current Density. *ACS Appl. Energy Mater.* **1**, 5355-5365 (2018).
30. Su, J. *et al.* Overcoming the abnormal grain growth in Ga-doped $Li_7La_3Zr_2O_{12}$ to enhance the electrochemical stability against Li metal. *Ceram. Int.* **45**, 14991-14996 (2019).
31. Xiang, X., Chen, F., Shen, Q., Zhang, L. & Chen, C. Effect of the lithium ion concentration on the lithium ion conductivity of Ga-doped LLZO. *Mater. Res. Express* **6**, 085546 (2019).
32. Huang, X. *et al.* Manipulating $Li_2O$ atmosphere for sintering dense $Li_7La_3Zr_2O_{12}$ solid electrolyte. *Energy Storage Mater.* **22**, 207-217 (2019).
33. Yang, L. *et al.* Rapid sintering method for highly conductive $Li_7La_3Zr_2O_{12}$ ceramic electrolyte. *Ceram. Int.* **46**, 10917-10924 (2020).
34. Xu, B. *et al.* Multistep sintering to synthesize fast lithium garnets. *J. Power Sources* **302**, 291-297 (2016).
35. Ren, Y., Shen, Y., Lin, Y. & Nan, C.-W. Microstructure Manipulation for Enhancing the Resistance of Garnet-Type Solid Electrolytes to "Short Circuit" by Li Metal Anodes. *ACS Appl. Mater. Interfaces* **11**, 5928-5937 (2019).
36. Ohta, S. *et al.* Co-sinterable lithium garnet-type oxide electrolyte with cathode for all-solid-state lithium ion battery. *J. Power Sources* **265**, 40-44 (2014).
37. Wu, J.-F. *et al.* Gallium-Doped $Li_7La_3Zr_2O_{12}$ Garnet-Type Electrolytes with High

Lithium-Ion Conductivity. *ACS Appl. Mater. Interfaces* **9**, 1542-1552 (2017).
38. Kim, Y. *et al.* The Effect of Relative Density on the Mechanical Properties of Hot-Pressed Cubic $Li_7La_3Zr_2O_{12}$. *J. Am. Ceram. Soc.* **99**, 1367-1374 (2016).
39. Yang, S. H. *et al.* Ionic conductivity of Ga-doped LLZO prepared using Couette-Taylor reactor for all-solid lithium batteries. *J. Ind. Eng. Chem.* **56**, 422-427 (2017).
40. Sharafi, A., Meyer, H. M., Nanda, J., Wolfenstine, J. & Sakamoto, J. Characterizing the Li-$Li_7La_3Zr_2O_{12}$ interface stability and kinetics as a function of temperature and current density. *J. Power Sources* **302**, 135-139 (2016).
41. Wolfenstine, J., Ratchford, J., Rangasamy, E., Sakamoto, J. & Allen, J. L. Synthesis and high Li-ion conductivity of Ga-stabilized cubic $Li_7La_3Zr_2O_{12}$. *Mater. Chem. Phys.* **134**, 571-575 (2012).
42. David, I. N., Thompson, T., Wolfenstine, J., Allen, J. L. & Sakamoto, J. Microstructure and Li-Ion Conductivity of Hot-Pressed Cubic $Li_7La_3Zr_2O_{12}$. *J. Am. Ceram. Soc.* **98**, 1209-1214 (2015).
43. Qin, S. *et al.* Extremely dense microstructure and enhanced ionic conductivity in hot-isostatic pressing treated cubic garnet-type solid electrolyte of $Ga_2O_3$-doped $Li_7La_3Zr_2O_{12}$. *Funct. Mater. Lett.* **11**, 1850029 (2018).
44. Sharafi, A. et al. Impact of air exposure and surface chemistry on Li-$Li_7La_3Zr_2O_{12}$ interfacial resistance. *J. Mater. Chem. A* **5**, 13475-13487 (2017).
45. Schmidt, R. D. & Sakamoto, J. In-situ, non-destructive acoustic characterization of solid state electrolyte cells. *J. Power Sources* **324**, 126-133 (2016).
46. Tenhaeff, W. E. *et al.* Resolving the Grain Boundary and Lattice Impedance of Hot-Pressed $Li_7La_3Zr_2O_{12}$ Garnet Electrolytes. *ChemElectroChem* **1**, 375-378 (2014).
47. Zhang, J., Li, J., Zhai, H., Tan, G. & Tang, X. One-Step Processing of Soft Electrolyte/Metallic Lithium Interface for High-Performance Solid-State Lithium Batteries. *ACS Appl. Energy Mater.* **3**, 6139-6145 (2020).
48. Lee, H. C., Oh, N. R., Yoo, A. R., Kim, Y. & Sakamoto, J. Preparation of a $Li_7La_3Zr_{1.5}Nb_{0.5}O_{12}$ Garnet Solid Electrolyte Ceramic by using Sol-gel Powder Synthesis and Hot Pressing and Its Characterization. *J. Korean Phys. Soc.* **73**, 1535-1540 (2018).
49. Sharafi, A., Haslam, C. G., Kerns, R. D., Wolfenstine, J. & Sakamoto, J. Controlling and correlating the effect of grain size with the mechanical and electrochemical properties of $Li_7La_3Zr_2O_{12}$ solid-state electrolyte. *J. Mater. Chem. A* **5**, 21491-21504 (2017).
50. Nonemacher, J. F. *et al.* Fracture toughness of single grains and polycrystalline $Li_7La_3Zr_2O_{12}$ electrolyte material based on a pillar splitting method. *J. Eur. Ceram. Soc.* **40**, 3057-3064 (2020).
51. Thompson, T. *et al.* A Tale of Two Sites: On Defining the Carrier Concentration in Garnet-Based Ionic Conductors for Advanced Li Batteries. *Adv. Energy Mater.* **5**, 1500096 (2015).
52. Bernuy-Lopez, C. *et al.* Atmosphere Controlled Processing of Ga-Substituted Garnets for High Li-Ion Conductivity Ceramics. *Chem. Mater.* **26**, 3610-3617 (2014).
53. Zhang, J. *et al.* Transformation of Undesired $Li_2CO_3$ into Lithiophilic Layer Via Double Replacement Reaction for Garnet Electrolyte Engineering. *Energy Environ. Mater.* **5**, 962-968 (2022).

54. Zhang, Y., Chen, F., Tu, R., Shen, Q. & Zhang, L. Field assisted sintering of dense Al-substituted cubic phase $Li_7La_3Zr_2O_{12}$ solid electrolytes. *J. Power Sources* **268**, 960-964 (2014).
55. Sazvar, A., Sarpoolaky, H. & Golmohammad, M. The effects of electric field on physical properties of LLZO made by flash sintering method. *Solid State Ion.* **386**, 116054 (2022).
56. Zhou, X. *et al.* $Sr^{2+}$ and $Mo^{6+}$ co-doped $Li_7La_3Zr_2O_{12}$ with superior ionic conductivity. *J. Alloys Compd.* **891**, 161906 (2022).
57. Zhang, Y. *et al.* High-purity and high-density cubic phase of $Li_7La_3Zr_2O_{12}$ solid electrolytes by controlling surface/volume ratio and sintering pressure. *Electrochim. Acta* **359**, 136965 (2020).
58. Botros, M., Djenadic, R., Clemens, O., Möller, M. & Hahn, H. Field assisted sintering of fine-grained $Li_{7-3x}La_3Zr_2Al_xO_{12}$ solid electrolyte and the influence of the microstructure on the electrochemical performance. *J. Power Sources* **309**, 108-115 (2016).
59. Yamada, H., Ito, T. & Hongahally Basappa, R. Sintering Mechanisms of High-Performance Garnet-type Solid Electrolyte Densified by Spark Plasma Sintering. *Electrochim. Acta* **222**, 648-656 (2016).
60. Dong, Z. *et al.* Dual Substitution and Spark Plasma Sintering to Improve Ionic Conductivity of Garnet $Li_7La_3Zr_2O_{12}$. *Nanomaterials* **9**, 721 (2019).
61. Zhao, B. *et al.* Interfacial modification enabled room temperature solid-state lithium-metal batteries. *Ionics* **27**, 1569-1578 (2021).
62. Zhao, X., Zhang, H., Yuan, Y., Ren, Y. & Wang, N. Ultra-fast and stable extraction of Li metal from seawater. *Chem. Commun.* **56**, 1577-1580 (2020).
63. Castillo, A. *et al.* Bulk Li mobility enhancement in Spark Plasma Sintered $Li_{(7-3x)}Al_xLa_3Zr_2O_{12}$ garnet. *Ceram. Int.* **44**, 18844-18850 (2018).
64. H. Sternlicht, S.A. Bojarski, G.S. Rohrer, W.D. Kaplan, Quantitative differences in the Y grain boundary excess at boundaries delimiting large and small grains in Y doped $Al_2O_3$, *J. Eur. Ceram. Soc.* **38**, 1829–1835 (2018).
65. K. Syed, M. Xu, K.K. Ohtaki, D. Kok, K.K. Karandikar, O.A. Graeve, W.J. Bowman, M.L. Mecartney, Correlations of grain boundary segregation to sintering techniques in a three-phase ceramic, *Materialia* **14**, 100890 (2020).
66. Posch, P. et al. Ion dynamics in Al-Stabilized $Li_7La_3Zr_2O_{12}$ single crystals-Macroscopic transport and the elementary steps of ion hopping. *Energy Storage Mater.* **24**, 220-228 (2020).
67. Dussart, T. *et al.* Critical Current Density Limitation of LLZO Solid Electrolyte: Microstructure vs Interface. *J. Electrochem. Soc.* **168**, 120550 (2021).
68. Rosen, M. *et al.* Controlling the lithium proton exchange of LLZO to enable reproducible processing and performance optimization. *J. Mater. Chem. A* **9**, 4831-4840 (2021).
69. Wang, M., Wolfenstine, J. B. & Sakamoto, J. Temperature dependent flux balance of the $Li/Li_7La_3Zr_2O_{12}$ interface. *Electrochim. Acta* **296**, 842-847 (2019).
70. Chen, S. *et al.* The Influence of Surface Chemistry on Critical Current Density for Garnet Electrolyte. *Adv. Funct. Mater.* **32**, 2113318 (2022).
71. Sim, J.-W. *et al.* Controlling Dendrite Formation for Long-Term Cyclability and Fast $Li^+$ Diffusion Kinetics in Solid-State Electrolyte $Li_7La_3Zr_2O_{12}$. *Chem. Mater.* **35**, 6538-6548

(2023).
72. Cai, M. *et al.* In-situ constructed lithium-salt lithiophilic layer inducing bi-functional interphase for stable LLZO/Li interface. *Energy Storage Mater.* **47**, 61-69 (2022).
73. Valle, J. M. & Sakamoto, J. The effect of lanthanoid defects on anionic solvation of Li in $Li_{6.5}La_{2+x}Zr_{1.5}Ta_{0.5}O_{12}$ from x = 0 to x = 1.2 garnet. *Solid State Ion.* **345**, 115170 (2020).
74. Taylor, N. J. *et al.* Demonstration of high current densities and extended cycling in the garnet $Li_7La_3Zr_2O_{12}$ solid electrolyte. *J. Power Sources* **396**, 314-318 (2018).
75. Cheng, L. *et al.* Effect of Surface Microstructure on Electrochemical Performance of Garnet Solid Electrolytes. *ACS Appl. Mater. Interfaces* **7**, 2073-2081 (2015).
76. Basappa, R. H., Ito, T. & Yamada, H. Contact between Garnet-Type Solid Electrolyte and Lithium Metal Anode: Influence on Charge Transfer Resistance and Short Circuit Prevention. *J. Electrochem. Soc.* **164**, A666 (2017).
77. Hongahally Basappa, R. *et al.* Grain boundary modification to suppress lithium penetration through garnet-type solid electrolyte. *J. Power Sources* **363**, 145-152 (2017).
78. Zhang, H. *et al.* Unveiling Surface Chemistry of Ultrafast-Sintered LLZO Solid-State Electrolytes for High-Performance Li-Garnet Solid-State Batteries. *Chem. Mater.* **36**, 11254-11263 (2024).
79. Flatscher, F., Philipp, M., Ganschow, S., Wilkening, H. M. R. & Rettenwander, D. The natural critical current density limit for $Li_7La_3Zr_2O_{12}$ garnets. *J. Mater. Chem. A* **8**, 15782-15788 (2020).
80. Zheng, C. *et al.* Grain Boundary Engineering Enabled High-Performance Garnet-Type Electrolyte for Lithium Dendrite Free Lithium Metal Batteries. *Small Methods* **6**, 2200667 (2022).
81. Gao, M. *et al.* Constructing a Multifunctional Interlayer toward Ultra-High Critical Current Density for Garnet-Based Solid-State Lithium Batteries. *Adv. Funct. Mater.* **33**, 2300319 (2023).
82. Lee, R.-H. & Lee, S.-H. Regulating the grain size of garnet type $Ga_{0.3}Li_{6.1}La_3Zr_2O_{12}$ for ultrafast $Li^+$ diffusion and enhanced cycling performance. *J. Energy Storage* **72**, 107421 (2023).
83. Okur, F. *et al.* Ultrafast Sintering of Dense $Li_7La_3Zr_2O_{12}$ Membranes for Li Metal All-Solid-State Batteries. *Adv. Sci.* **12**, 2412370 (2025).
84. Zhang, H. *et al.* On High-Temperature Thermal Cleaning of $Li_7La_3Zr_2O_{12}$ Solid-State Electrolytes. *ACS Appl. Energy Mater.* **6**, 6972-6980 (2023).
85. Zhao, G., Luo, C. & Hua, Q. Enhanced comprehensive performance of the LLZO series solid electrolyte via multifunctional additive. *J. Eur. Ceram. Soc.* **44**, 2251-2260 (2024).
86. Guo, J. & Chan, C. K. Lithium Dendrite Propagation in Ta-Doped $Li_7La_3Zr_2O_{12}$ (LLZTO): Comparison of Reactively Sintered Pyrochlore-to-Garnet vs LLZTO by Solid-State Reaction and Conventional Sintering. *ACS Appl. Mater. Interfaces* **16**, 4519-4529 (2024).
87. Tsai, C.-L. *et al.* $Li_7La_3Zr_2O_{12}$ Interface Modification for Li Dendrite Prevention. *ACS Appl. Mater. Interfaces* **8**, 10617-10626 (2016).
88. Xiang, X. et al. Constructing electron-blocking grain boundaries in garnet to suppress lithium dendrite growth. *J. Adv. Ceram.* **13**, 166-175 (2024).

89. Lee, R.-H. et al. Tailoring the grain boundary structure and chemistry of the dendrite-free garnet solid electrolyte $Li_{6.1}Ga_{0.3}La_3Zr_2O_{12}$. *NPG Asia Mater.* **16**, 42 (2024).

90. Raj, V. et al. Grain boundary zirconia-modified garnet solid-state electrolyte. *Nat. Mater.* (2025). https://doi.org/10.1038/s41563-025-02374-9

91. Jeong, W. et al. Tailoring grain boundary structures and chemistry of $Li_7La_3Zr_2O_{12}$ solid electrolytes for enhanced air stability. *Energy Storage Mater.* **54**, 543-552 (2023).

92. Du, C. et al. Constructing a three-dimensional continuous grain boundary with lithium ion conductivity and electron blocking property in LLZO to suppress lithium dendrites. *J. Alloys Compd.* **1003**, 175769 (2024).

93. Qin, Z. et al. Grain Boundary Engineering in Ta-Doped Garnet-Type Electrolyte for Lithium Dendrite Suppression. *ACS Appl. Mater. Interfaces* **14**, 40959-40966 (2022).

94. Luo, P. et al. $TiO_2$-Induced Conversion Reaction Eliminating $Li_2CO_3$ and Pores/Voids Inside Garnet Electrolyte for Lithium–Metal Batteries. *Adv. Funct. Mater.* **33**, 2302299 (2023).

95. Zheng, C. et al. Grain boundary modification in garnet electrolyte to suppress lithium dendrite growth. *Chem. Eng. J.* **411**, 128508 (2021).

96. Biao, J. et al. Inhibiting Formation and Reduction of $Li_2CO_3$ to $LiC_x$ at Grain Boundaries in Garnet Electrolytes to Prevent Li Penetration. *Adv. Mater.* **35**, 2208951 (2023).

97. Wang, C.-C. et al. Grain boundary complexion modification for interface stability in garnet based solid-state Li batteries. *J. Power Sources* **602**, 234394 (2024).

98. Altus, S. J. et al. Impact of Secondary Phases on the Electrochemical Performance of $Li_{6.4}La_3Zr_{1.4}Ta_{0.6}O_{12}$ Garnet Solid Electrolytes through Grain Boundary Engineering. *Chem. Mater.* **37**, 7136-7146 (2025).

99. Liu, J. et al. $LiGaO_2$-mediated grain boundary engineering in Ta-doped $Li_7La_3Zr_2O_{12}$ solid electrolyte. *Mater. Chem. Front.* (2026). https://doi.org/10.1039/D5QM00736D

100. Lee, J. et al. Uniformization of Electron Distribution at Grain Boundaries via Work Function Adjusting Interlayer to Prevent Li-Filament Formation Within Solid Electrolytes. *Small* **21**, 2412799 (2025).

101. Xiong, B.-Q. et al. Field-responsive grain boundary against dendrite penetration for all-solid-state batteries. *Energy Environ. Sci.* **17**, 6707-6716 (2024).
