# Supplementary material for: Elimination of detrimental grain boundary segregation in garnets
Source: Nat Commun. 2026 Jun 29;17:8667. doi: 10.1038/s41467-026-74887-z (PMC13490533; doi:10.1038/s41467-026-74887-z)
Supplement: Supplementary file 2 [file 41467_2026_74887_MOESM2_ESM.pdf]

<u>Description of Additional Supplementary Files</u>

Supplementary data 1: Twenty grain boundary models for segregation energy calculations.

Supplementary data 2: The files of certain frames during the MLFF-MD simulation process.

Supplementary data 3: Three small grain boundary models for electronic structure and chemical potential calculations.
