# Supplementary material for: Elimination of detrimental grain boundary segregation in garnets
Source: Nat Commun. 2026 Jun 29;17:8667. doi: 10.1038/s41467-026-74887-z (PMC13490533; doi:10.1038/s41467-026-74887-z)
Supplement: Supplementary file 3 [file 41467_2026_74887_MOESM4_ESM.pdf]

nature portfolio

Peer Review File

Elimination of Detrimental Grain Boundary Segregation in Garnets

Corresponding Author: Professor Dina Fattakhova-Rohlfing

This file contains all reviewer reports in order by version, followed by all author rebuttals in order by version.

Version 0:

Reviewer comments:

Reviewer #1

(Remarks to the Author)
This manuscript presents a comprehensive investigation of grain-boundary chemistry and transport in doped LLZO solid electrolytes. The authors propose a kinetic strategy to suppress detrimental elemental segregation during sintering. The topic is timely and relevant to the solid-state battery community. The dataset is substantial, and several observations (e.g., the formation of a transparent oxide ceramic pellet) are intriguing. I recommend publication in Nature Communications after the authors address the following concerns:
1. The manuscript focuses on Al, Ta, and Zr as representative species to investigate segregation behaviors. However, the motivation for choosing these particular elements is not sufficiently articulated. Please clarify (i) what scientific or practical considerations guided the selection (e.g., common dopants in LLZO, charge compensation mechanisms, ionic radii/valence, known segregation tendencies), and (ii) whether the observed segregation trends can be rationalized in terms of simple descriptors. In particular, it would be helpful if the authors could provide qualitative insights—without requiring rigorous derivations—on (a) thermodynamic propensity for GB occupation (e.g., segregation energy trends vs. ionic size/charge, local coordination preference, elastic misfit, bonding character), and (b) kinetic propensity for migration toward GBs (e.g., diffusivity trends, defect association, vacancy-mediated transport, impact of sintering temperature/time). Even a semi-quantitative discussion or a descriptor-based framework would significantly improve the mechanistic clarity and general value of the work.
2. I do not agree that comparing band-gap values alone is sufficient to infer electronic conductivity, unless the band gap remains large enough (e.g., >3 eV) such that the material remains strongly insulating and conductivity changes are dominated by intrinsic carriers. In LLZO grain boundaries, early experimental evidence suggests that the local band gap can be substantially smaller. For instance, the prior study "Local electronic structure variation resulting in Li 'filament' formation within solid electrolytes" reports a GB band gap possibly below ~2 eV. Please reconcile this with the present experimental and computational results. Specifically, the authors should explain potential sources of discrepancy, such as differences in GB structure/chemistry, dopant distribution, sample processing, measurement modality, or computational setup (e.g., functional choice, treatment of disorder, presence/absence of defects).
3. Beyond Li nucleation at grain boundaries, fracture mechanics can strongly impact Li|LLZO interfacial stability. The enhanced CCD observed for SF-LLZO could potentially arise, at least in part, from improved densification (reduced porosity and crack initiation sites), rather than solely from changes in GB chemistry/electronic leakage. Moreover, Li metal itself undergoes cyclic mechanical fatigue during repeated plating/stripping, which can exacerbate contact loss and local current focusing. The authors should expand the discussion to consider these mechanical contributions and, where possible, provide post-mortem evidence such as cross-sectional SEM of Li/LLZO interface.
4. I am wondering whether the proposed kinetic strategy is broadly applicable to various ceramic materials. Can authors provide additional evidence supporting extension beyond LLZO.
5. In the galvanostatic cycling profiles of Li symmetric cells, cell using SF-LLZO demonstrates a lower overpotential than its counterpart. Please provide an explanation.

Reviewer #2

(Remarks to the Author)
This study reports the detrimental effects and underlying mechanisms of metal segregation at grain boundaries in LLZO

solid-state electrolytes. Based on these mechanistic insights, a universal strategy is proposed to achieve segregation-free grain boundaries via a rapid, precisely controlled sintering protocol that exploits the onset of solid-state softening, identified by an inflection in the densification curve. However, before the work can be considered for publication in Nature Communications, several issues need to be addressed.

1. Do any metal compounds form at the grain boundaries when segregation occurs?
2. In line 127, the authors state: “These dark regions are assumed to be Li-rich phases, which appear dark in HAADF imaging due to the low atomic number of Li and are probably formed during post-treatment.” However, given the very low atomic number of Li (Z = 3), its contribution to HAADF contrast is negligible, making Li-rich phases difficult to directly identify by HAADF imaging alone.
3. How did the authors confirm the formation of a lithium-rich phase, aside from HAADF imaging?
4. Why can LLZO with nearly no grain boundaries be prepared by FAST/SPS? Can such grain-boundary-free LLZO be considered a single crystal with some preferred orientation?

Reviewer #3

(Remarks to the Author)
This work elucidated the structural origins and electrochemical consequences of grain boundary segregation in Garnet $Li_7La_3Zr_2O_{12}$ (LLZO) electrolyte. Accordingly, a universal strategy was developed to achieve segregation-free grain boundaries through a rapid yet precisely controlled sintering protocol. However, significant flaws exist in the rigor of experimental design, the thoroughness of data analysis, and the reliability of conclusions, undermining the paper's scientific integrity and credibility. Therefore, the existing article must undergo thorough and rigorous revision to address these deficiencies.
1. The author determines the “solid softening point” based on the inflection point of the displacement curve as the criterion for the sintering endpoint, claiming this strategy is applicable to various ceramic electrolytes. However, the definition of the sintering endpoint in the paper is ambiguous and lacks sufficient theoretical support. Furthermore, materials with differing chemical compositions, powder particle sizes, and doping types will inevitably exhibit distinct softening behaviors (i.e., inflection point temperatures and displacement change rates). How can one “pre-determine” or “real-time judge” the inflection point for new materials based on limited initial experimental data to demonstrate the predictability and universality of this strategy?
2. This study lacks critical characterization of the dendrite failure mechanism in symmetric cell testing. Following CCD testing and cycling, the authors recorded only voltage signals. Voltage signals alone cannot determine which stage of dendrite growth is inhibited. Cross-sectional SEM images of the cycled battery must be provided to directly observe whether dendrites grow along grain boundaries, through grains, or nucleate at the electrode/electrolyte interface. Otherwise, improved CCD and cycle life cannot be directly attributed to “inhibited lithium nucleation along grain boundaries.”
3. The evidence chain supporting the “no segregation” conclusion in the article is incomplete. The authors hastily claim “no elemental segregation” based solely on HAADF-STEM/EDS mapping of one or a few grain boundaries, rendering the conclusion extremely hasty and highly unscientific. The paper neither specifies the detection limits of the EDS mapping nor provides details on the specific grain boundary observation areas or the number of grain boundaries examined. It offers no detailed description of the distribution within grain boundaries, and the statistical sample size is severely inadequate. Therefore, the conclusion of no segregation cannot be blindly drawn. This demonstrates a lack of rigor in the paper.
4. The article lacks comparison with existing advanced grain boundary engineering strategies. Numerous reports in recent literature have successfully enhanced LLZO performance through “grain boundary engineering” approaches such as grain boundary doping and second-phase modification (NPG Asia Mater 2024 16(1), 1884-4049; Nat. Mater. 2025, 1476-1122; Nat Commun 2025 16(1), 2041-1723). The authors' work essentially focuses on “avoiding the formation of harmful grain boundaries.” However, the paper only compares results with unmodified LLZO, neglecting representative data points from recent studies that achieved superior performance through active “grain boundary engineering.” Such comparisons merely demonstrate the advantages of conventional grain boundary modification without highlighting the innovation of the grain boundary modification in this paper. Therefore, the paper should not only contrast with “unmodified” LLZO but also discuss recent boundary engineering approaches. This would emphasize the relative advantages of the presented method in terms of performance, process complexity, and cost.
5. The analysis of ion conductivity activation energy in the paper lacks depth. The authors point out that the total activation energy of SF-LLZO (0.427 eV) is nearly identical to the grain activation energy of S-LLZO (0.429 eV), using this similarity as evidence that grain boundary impedance is negligible in SF-LLZO. However, for polycrystalline ceramics, the total activation energy represents a weighted average of bulk and grain boundary conduction pathways. Directly equating the total activation energy to the grain boundary activation energy is overly simplistic. More precise activation energy data should be obtained through more accurate models or by performing a more refined distribution of relaxation time (DRT) analysis of the EIS data.
6. The core comparative experiments are missing from the article. The most critical performance comparison throughout the paper (between S-LLZO and SF-LLZO) relies on samples made from identical powders but using different sintering techniques (conventional sintering and FAST/SPS). The inherent electric field, pressure, and reducing atmosphere of FAST/SPS differ from conventional sintering, and these factors themselves may independently influence grain boundary structure and performance. A critical control experiment must be added: prepare a “FAST/SPS-slow firing” sample using the same FAST/SPS equipment but simulating conventional sintering with a ‘slow’ heating ramp and extended soak time. Only by comparing with this control sample can the performance improvement be attributed to “time reduction/kinetic suppression” rather than other effects inherent to the FAST/SPS technology itself.

Version 1:

Reviewer comments:

Reviewer #1

(Remarks to the Author)
Authors have carefully revised the paper, which contains interesting results. I recommend it for publication without revision.

Reviewer #2

(Remarks to the Author)
The authors have addressed the issues raised by the reviewer. The current version of the manuscript is suitable for acceptance.

Reviewer #3

(Remarks to the Author)
concerns have been well addressed.

Open Access This Peer Review File is licensed under a Creative Commons Attribution 4.0 International License, which permits use, sharing, adaptation, distribution and reproduction in any medium or format, as long as you give appropriate credit to the original author(s) and the source, provide a link to the Creative Commons license, and indicate if changes were made.
In cases where reviewers are anonymous, credit should be given to 'Anonymous Referee' and the source.
The images or other third party material in this Peer Review File are included in the article's Creative Commons license, unless indicated otherwise in a credit line to the material. If material is not included in the article's Creative Commons license and your intended use is not permitted by statutory regulation or exceeds the permitted use, you will need to obtain permission directly from the copyright holder.
To view a copy of this license, visit https://creativecommons.org/licenses/by/4.0/

Elemental Segregation at LLZO Grain Boundaries: Eliminating Its Detrimental Role in Conductivity and Lithium Nucleation (NCOMMS-25-92836-T)

Response to Reviewers' Comments

Reviewer #1

Remarks to the Author:

This manuscript presents a comprehensive investigation of grain-boundary chemistry and transport in doped LLZO solid electrolytes. The authors propose a kinetic strategy to suppress detrimental elemental segregation during sintering. The topic is timely and relevant to the solid-state battery community. The dataset is substantial, and several observations (e.g., the formation of a transparent oxide ceramic pellet) are intriguing. I recommend publication in Nature Communications after the authors address the following concerns:

Author Response

We are grateful to the reviewer for the positive feedback and numerous constructive suggestions, which have contributed to improving the quality of the manuscript. A detailed response to the comments can be found below. The changes are highlighted in yellow in the revised version of the manuscript.

Comment 1

The manuscript focuses on Al, Ta, and Zr as representative species to investigate segregation behaviors. However, the motivation for choosing these particular elements is not sufficiently articulated. Please clarify (i) what scientific or practical considerations guided the selection (e.g., common dopants in LLZO, charge compensation mechanisms, ionic radii/valence, known segregation tendencies),

Response 1

We thank the reviewer for this insightful comment. The choice of Ta- and Al-codoped LLZO as the main compound for investigating segregation effects was based on several considerations. The most important of those were the proven effectiveness of these dopants in improving the ionic conductivity of LLZO and the importance of this material for the development of practical batteries. Among the commonly reported dopants, only Ta- and Ga-doped LLZO have achieved ionic conductivity above 1 mS cm^{-1}. However, Ga-doped LLZO is known to be unstable in contact with Li metal (J. Mater. Chem. A, 2022, 10, 10998-11009) and thermodynamically unstable during co-sintering with cathode active materials (CAMs) (Energy Mater. 2025, 5, http://dx.doi.org/10.20517/energymater.2025.174). Unlike Ga doped LLZO, the Ta-doped LLZO exhibits excellent reduction stability towards Li metal as well as good

thermodynamic stability with various CAMs, including $LiCoO_2$ and NCM, making it suitable as an anolyte, catholyte, and separator material in various cell designs. Therefore, Ta was selected as the primary dopant. A small amount of Al was additionally introduced to stabilize the cubic phase and to facilitate densification during sintering (J. Power Sources, 2011, 196, 8683-8687). We have expanded the relevant discussion in the manuscript to more clearly justify the choice of dopants.

Change to manuscript (pages 4-5)

"Doping with various elements such as Al, Ta, Ga, Nb, Hf, and Mo has been extensively used as an effective means to stabilize the cubic phase at room temperature and improve the performance of LLZO in different parts of the cell[4,5]. Among the commonly used dopants, only Ta- and Ga-doped LLZO have achieved ionic conductivity above 1 mS cm^{-1}. However, Ga-doped LLZO is known to be unstable in contact with Li metal and thermodynamically unstable during co-sintering with cathode active materials (CAMs)[34,35]. In contrast to Ga, Ta-doped LLZO exhibits excellent reduction stability towards Li metal as well as good thermodynamic stability with various CAMs, including $LiCoO_2$ and NCM, making it suitable as an anolyte, catholyte, and separator material in various cell designs. Based on these considerations, this work focuses primarily on Ta-doped LLZO ($Li_{6.45}Al_{0.05}La_3Zr_{1.6}Ta_{0.4}O_{12}$), with a small amount of Al additionally introduced to promote densification during sintering[35], which was shown to be the most promising composition for the use in practical all-solid-state oxide batteries."

Comment 2

Please clarify (ii) whether the observed segregation trends can be rationalized in terms of simple descriptors. In particular, it would be helpful if the authors could provide qualitative insights—without requiring rigorous derivations—on (a) thermodynamic propensity for GB occupation (e.g., segregation energy trends vs. ionic size/charge, local coordination preference, elastic misfit, bonding character), and (b) kinetic propensity for migration toward GBs (e.g., diffusivity trends, defect association, vacancy-mediated transport, impact of sintering temperature/time). Even a semi-quantitative discussion or a descriptor-based framework would significantly improve the mechanistic clarity and general value of the work.

Response 2

We fully agree with the reviewer that the establishment of a simplified descriptor-based framework would be helpful in guiding the material design. However, for the reasons explained below, we have not yet fully succeeded in creating such a simplified framework for LLZO.

As far as thermodynamic considerations are concerned, simplified thermodynamic descriptors have indeed been successfully used to rationalize segregation behavior in several alloy systems and a limited number of well-studied inorganic materials, such as perovskites and Al_2O_3. The descriptors used included elastic mismatch due to atomic

size differences, electrostatic interactions related to dopant charge states, or other classical parameters correlating with GB energetics (Prog. Mater. Sci., 2004, 49, 109-208, J. Am. Ceram. Soc., 1990, 73, 3407-3415). It is important to note that such frameworks are typically built upon extensive statistical analyses of large TEM/EDS datasets collected over decades. Despite intensive studies of LLZO as a solid electrolyte, the number of experimentally confirmed segregation cases remains very limited. To our knowledge, direct segregation toward GB has been observed for only a few elements, such as Al and Ta. This scarcity is mainly due to the extreme sensitivity of LLZO to electron beam irradiation, which makes reliable EDS characterization particularly difficult and requires stringent control of experimental conditions. In our previous attempts, even cryo-TEM was unable to sufficiently reduce beam damage. Considering only the currently available Al and Ta data, our MLFF-MD results (Supplementary Fig. 7) indicate that the differences in local coordination preference between these dopants are not pronounced enough to serve as a robust thermodynamic descriptor. From an electrostatic perspective, both Al^{3+} and Ta^{5+} are aliovalent dopants with a higher valence than Li^{+} and Zr^{4+}, which may suggest a negatively charged grain-boundary core. This interpretation is qualitatively consistent with previous reports indicating Li accumulation at GBs (J. Power Sources, 2022, 539, 231417). However, we believe that proposing a general descriptor based on such a limited dataset would not be statistically and physically robust enough.

Regarding kinetic considerations, we have provided a semi-quantitative, descriptor-based discussion to rationalize segregation in terms of diffusivity trends, defect association/vacancy-mediated transport, and the impact of sintering temperature and time. Specifically, our DFT results show that Ta and La can transiently occupy Li sites via Frenkel-type defect formation at elevated temperatures, enabling their migration through Li-ion channels (i.e., a defect-assisted and vacancy-mediated transport pathway). MLFF-MD simulations further quantify the diffusivity trends at sintering-relevant temperatures and show that Al, Ta, and La can diffuse rapidly along the Li-ion channels in cubic LLZO, with La exhibiting significantly higher diffusivity than Al and Ta. This provides a kinetic descriptor that explains why La can still efficiently reach GB regions and show noticeable segregation even with negligible thermodynamic preference, while Ta segregation benefits from both fast transport kinetics and a strong driving force. Moreover, our discussion emphasizes the role of sintering temperature/time: higher temperature activates defect formation and accelerates long-range migration, and longer sintering duration provides sufficient time for dopants to reach GBs and for GB structures to evolve toward thicker/disordered interfacial regions, thereby increasing segregation (pages 8-9 in the revised version of the manuscript).

Overall, this work provides, to our knowledge, the first mechanistic explanation of segregation effects in LLZO using a simplified, descriptor-based framework that links segregation propensity to a small set of thermodynamic and kinetic descriptors. Importantly, the descriptor-guided picture is experimentally validated and offers a practical route to suppress detrimental GB segregation, enabling the reproducible fabrication of segregation-free, highly dense LLZO.

Comment 3

I do not agree that comparing band-gap values alone is sufficient to infer electronic conductivity, unless the band gap remains large enough (e.g., >3 eV) such that the material remains strongly insulating and conductivity changes are dominated by intrinsic carriers. In LLZO grain boundaries, early experimental evidence suggests that the local band gap can be substantially smaller. For instance, the prior study "Local electronic structure variation resulting in Li 'filament' formation within solid electrolytes" reports a GB band gap possibly below ~2 eV. Please reconcile this with the present experimental and computational results. Specifically, the authors should explain potential sources of discrepancy, such as differences in GB structure/chemistry, dopant distribution, sample processing, measurement modality, or computational setup (e.g., functional choice, treatment of disorder, presence/absence of defects).

Response 3

We agree with the reviewer's opinion that the band gap over 3 eV would not be sufficient to ensure adequate electronic conductivity, considering the measured value of about 2 eV reported in the paper mentioned by the reviewer (Nat. Mater. 2021, 20, 1485-1490). We would like to emphasize that the purpose of our DFT calculations is not to make accurate predictions of the electronic structure comparable to experiments, but to investigate the influence of Ta segregation at GBs on the band gap and electronic conductivity. Our results clearly and qualitatively explain this effect in comparison with experimental observations. Regarding the reviewer's inquiry, there are many factors in the DFT calculation that can lead to deviations from the experimental observations. 1) Size limitation in DFT: due to the limitation of the DFT simulation scale, the thickness of the GB models in our study is about 0.6–0.7 nm, while in the real system it is at least one order of magnitude thicker (5–10 nm). Although we have attempted to model GBs more realistically than in previous reports by incorporating the ML potential for perturbation and equilibration of GB structures with more than 1000 atoms, it is likely that the size difference leads to differences in electronic properties. 2) Statistical sampling problem: again, due to size limitation in DFT, we have investigated three simple coherent symmetric tilt GB models with low sigma numbers and low-index surfaces and rotation axes, whereas real LLZO samples have diverse GBs with a wide distribution of misorientation angles. These less well-defined GB structures would have smaller local band gaps. 3) Composition variations and chemical defects: our small models do not necessarily exhibit compositional variations with deviations from stoichiometry in the GB regions that would alter the local band gaps at the GBs. Furthermore, chemical defects at the GBs would add extra defect states between the band gap and facilitate the excitation of electrons with significantly lower activation energies. In short, we expect thicker and less well-defined GB structures with local composition variations and chemical defects to have smaller band gaps than the model GBs used in the DFT calculations. We have included a corresponding discussion in the manuscript.

Change to manuscript (page 19)

"Previous theoretical and experimental studies have shown that GBs with higher electronic conductivity facilitate dendrite initiation and propagation[13,18]. To evaluate the influence of elemental segregation on electronic conductivity, a 100-hour DC polarization experiment was performed (Fig. 4d). The results show that SF-LLZO exhibits electronic conductivity that is approximately one order of magnitude lower than that of segregated samples, including S-LLZO and air-annealed SF-LLZO, and that the presence of GBs actually correlates with increased electronic conductivity of polycrystalline LLZO at the macroscopic level. The reported experimental results of other groups, such as Liu et al., show that LLZO GBs can have a markedly reduced bandgap (1–3 eV), in sharp contrast to the grain interior (5.7–5.8 eV)[13]. We used DFT calculations to evaluate how GB segregation affects the bandgap, using the generalized gradient approximation (GGA) level of theory. As shown in Fig. 4e, bulk LLZO has a band gap of 4.35 eV, while the band gap at GBs is about 0.6–0.9 eV lower. With Ta segregation, the average band gap decreases by a further 0.2–0.3 eV, with the maximum decrease reaching 0.6 eV. Therefore, our calculations support experimental observation by Liu et al. regarding the higher electronic conductivity of GBs. In addition to dopant segregation, other GB effects that were not explicitly considered in our DFT calculations may also contribute to the observed difference in electronic conductivity. For example, differences in GB thickness, such as the thinner GBs in SF-LLZO compared to S-LLZO, could influence the local electronic structure and therefore affect the electronic conductivity. The higher electronic conductivity of GBs together with the higher concentration of Li-ions at the GB edges will fertilize the reduction of Li-ions and dendrite formation along the GBs. It should be noted that the present DFT analysis is intended to provide a qualitative assessment within a consistent computational framework and to offer mechanistic insights into GB electronic conductivity, rather than to quantitatively predict absolute bandgap values, given the intrinsic structural complexity of GBs and the limitations associated with the choice of exchange-correlation functional."

Comment 4

Beyond Li nucleation at grain boundaries, fracture mechanics can strongly impact Li|LLZO interfacial stability. The enhanced CCD observed for SF-LLZO could potentially arise, at least in part, from improved densification (reduced porosity and crack initiation sites), rather than solely from changes in GB chemistry/electronic leakage. Moreover, Li metal itself undergoes cyclic mechanical fatigue during repeated plating/stripping, which can exacerbate contact loss and local current focusing. The authors should expand the discussion to consider these mechanical contributions and, where possible, provide post-mortem evidence such as cross-sectional SEM of Li/LLZO interface.

Response 4

We thank the reviewer for the constructive comments, which prompted us to perform

additional post-mortem characterization of segregated and segregation-free LLZO separator pellets after cell short circuiting. We expanded the respective discussion in the manuscript with the results of SEM and EDS analysis:

Change to manuscript (page 22)

Post-mortem analysis of the cells after a hard short circuit also shows that segregated and segregation-free LLZO pellets exhibit remarkable differences in Li penetration mechanisms. In S-LLZO pellets, dark areas can be observed on the surface where dendrites form[21] (Fig. 5d). Local cross-sectional SEM images of this area show that Li grows along the GBs and forms interconnected filaments (Fig. 5e). This is consistent with the reported bulk-mediated dendrite growth mode, where Li nucleation and interconnection occur along the GBs[13]. In contrast, SF-LLZO exhibits a distinctly different dendrite growth behavior, in which Li dendrites penetration is accompanied by crack formation (Fig. 5f and g). The cracks run either slanted or almost perpendicular to the surface through the entire pellet thickness without following the microstructure of the LLZO grains. The cracks are filled with lithium, which can be seen along the crack length and along the fractured surface (Fig. 5h-j and Supplementary Fig. 8), as confirmed by EDS mapping, where C and O are enriched (Li was oxidized to Li_2CO_3 during sample transfer), while Zr and La (LLZO) are absent (Supplementary Fig. 8). This behavior is very similar to the performance reported for single-crystal LLZO[53], suggesting a surface-initiated dendrite growth mechanism in which dendrites propagate from one electrode to the other through SF-LLZO separator, while the high stress concentrated at the dendrite tips promotes crack initiation and development[54,55]. The distinctly different dendrite propagation behavior can be attributed to the differences in the structure of segregated and segregation-free GB discussed above, including differences in the local distribution of Li-ions and electronic conductivity. We therefore propose that the dendrite tolerance of polycrystalline LLZO separators is mainly determined by the morphology (relative density) and electronic conductivity of the GBs, the latter being strongly influenced by the dopants and the element segregation. After eliminating element segregation in SF-LLZO, the dendrite tolerance is largely determined by the mechanical properties (mainly fracture toughness) of the LLZO crystal structure and is close to maximum value expected for this material. A further increase in CCD would primarily require optimization of the mechanical properties of the polycrystalline LLZO scaffold and improving its ability to relief the stress occurring during Li plating, with research directions including optimization of particle size/particle size distribution and GB modification to improve the total ductility.

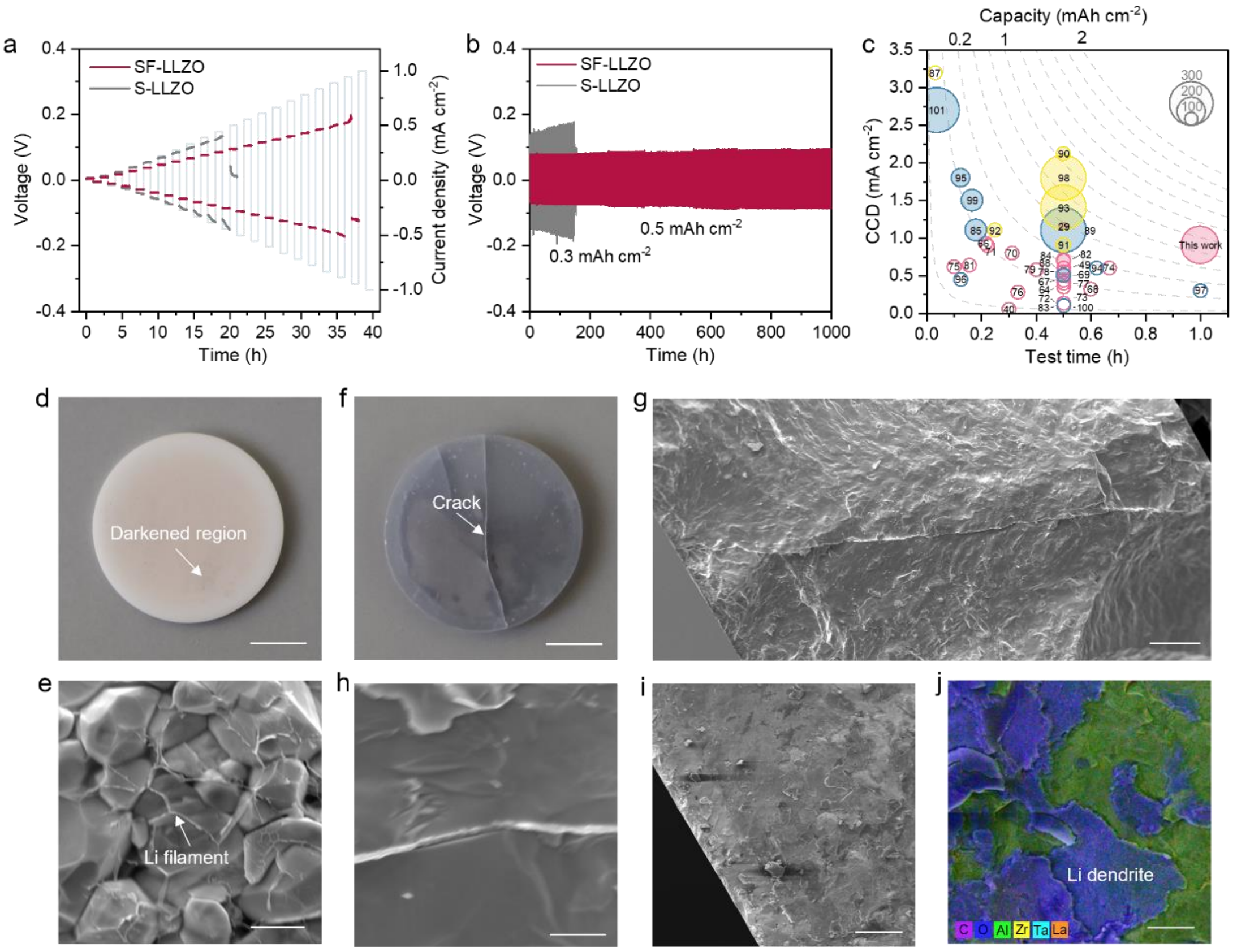

“**Fig. 5 | Dendrite tolerance assessment. a**,**b**, CCD (**a**) and galvanostatic cycling (**b**) measurements of S- and SF-LLZO pellets at RT. **c**, Comparison of reported CCDs and CCD capacities of LLZOs, together with total accumulated capacities during galvanostatic cycling (bubbles). Pink, blue, and yellow bubbles denote pristine, GB-engineered, and both GB- and surface-modified LLZO, respectively. Filled bubbles indicate accumulated capacities and open bubbles indicate <50 mAh cm^{-2} or not provided. Current densities are normalized to the effective Li/LLZO contact area. References are listed in Supplementary Table 7. **d**,**e**, Post-mortem analysis after dendrite penetrating S-LLZO (removing one Li electrode), shown by an optical top-view image (**d**) and local cross-sectional SEM images at dark regions (**e**). Scale bars are 3 mm (**d**), 5 μm (**e**). **f-j**, Post-mortem analysis after dendrite penetrating SF-LLZO (removing one Li electrode), shown by an optical top-view image (**f**), local cross-sectional (**g**,**h**), and fractured SEM image and EDS overlay mapping at cracks (**i**,**j**). Scale bars are 3 mm (**f**), 100 μm (**g**), 5 μm (**h**), 50 μm (**i**) and 10 μm (**j**).”

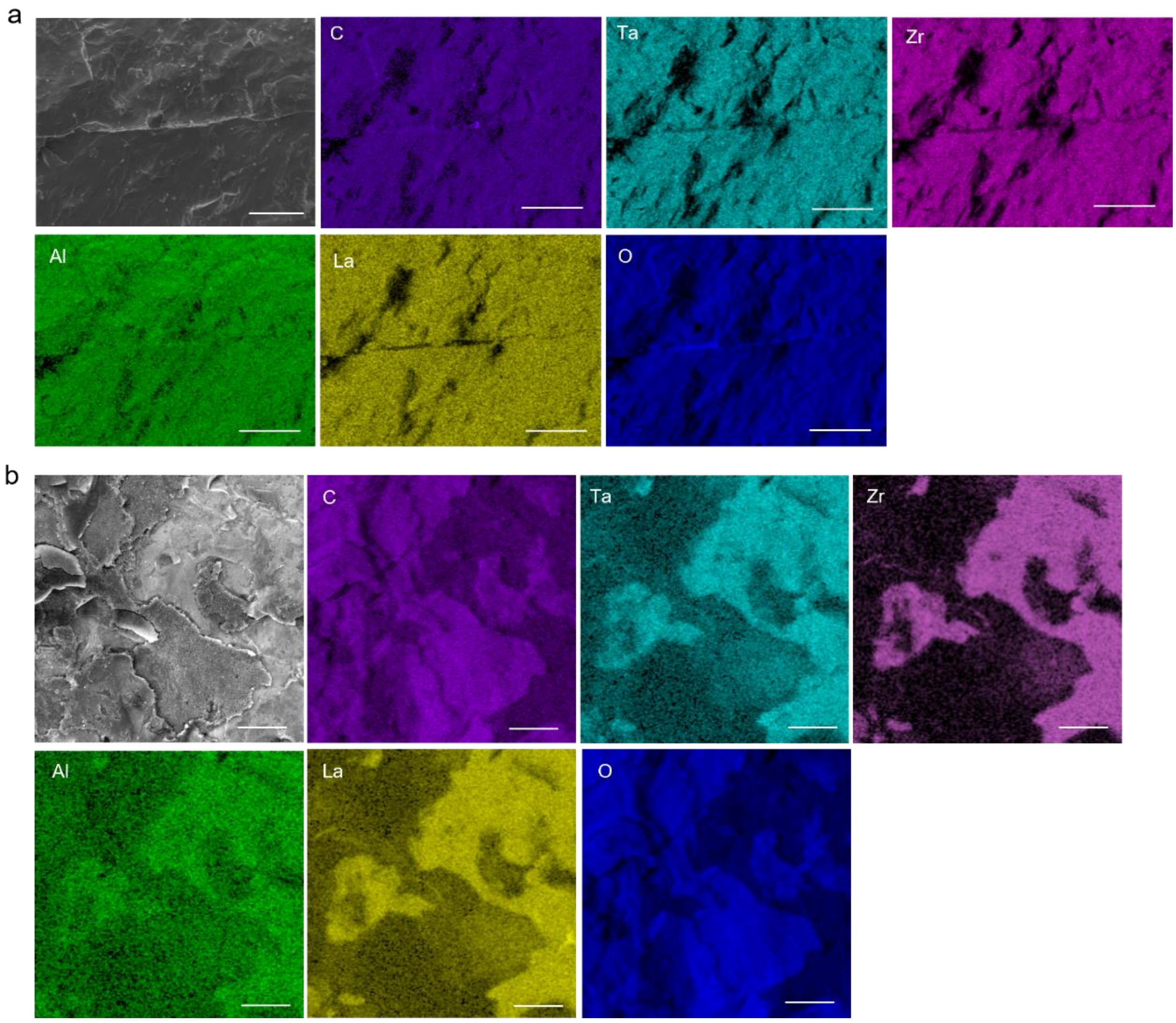

“**Supplementary Fig. 8 | Post-mortem analysis after dendrite penetration. a**,**b**, Local cross-sectional (**a**), and fractured (**b**) EDS mappings at cracks after dendrite penetrating SF-LLZO. Scale bars are 50 μm (**a**) and 10 μm (**b**).”

Comment 5

I am wondering whether the proposed kinetic strategy is broadly applicable to various ceramic materials. Can authors provide additional evidence supporting extension beyond LLZO.

Response 5

We believe that this strategy is not only effective for LLZO, but can also be applied to other ceramic materials, as has been demonstrated in recent studies. For example, it has been shown that rapid sintering suppresses Y segregation at grain boundaries in $BaZr_{0.7}Ce_{0.2}Y_{0.1}O_{3-\delta}$ (Adv. Energy Mater. 2024, 15, 2404410, doi.org/10.1002/aenm.202404410), and it has been reported that a fast sintering strategy mitigates Ti segregation at grain boundaries in $SrTiO_3$ (J. Eur. Ceram. Soc. 2023, 43, 6925–6933). However, it should be noted that the correlation between segregation at grain boundaries and functionality in different applications may vary for different materials, and that the mechanism of this correlation should be investigated

individually for each material and application. For example, in this paper (Adv. Energy Mater. 2024, 2404410), the authors pointed out that the segregation of acceptor dopants at GBs may actually favor proton transport in proton-conducting ceramics due to the reduction of positive space charge potential.

We can say with certainty that our processing strategy is universal in achieving highly dense ceramics across different compositions and that all resulting materials have a relative density of over 99%. However, understanding the segregation mechanism and its effects on material functionality would require in-depth characterization of the structure and properties of all these materials, which we have not yet performed.

Comment 6

In the galvanostatic cycling profiles of Li symmetric cells, cell using SF-LLZO demonstrates a lower overpotential than its counterpart. Please provide an explanation.

Response 6

A lower overpotential of SF-LLZO can originate from several factors. First, SF-LLZO has significantly higher ionic conductivity than S-LLZO, resulting in reduced ohmic polarization. Second, SF-LLZO has a much higher density, so its surface in contact with Li metal is expected to be more compact, whereas the surface of S-LLZO may contain residual pores. This difference is likely to result in a larger effective contact area for SF-LLZO, reducing the interfacial resistance. Furthermore, we assume that the absence of local composition variations on the SF-LLZO surface leads to a more homogeneous structure of the Li/LLZO interface and thus to more uniform Li plating/stripping during cycling, which may further reduce the interfacial resistance and contribute to lower overpotential. We have incorporated these considerations into the revised manuscript, as summarized below:

Change to manuscript (page 21)

"Galvanostatic cycling demonstrates outstanding stability of SF-LLZO over 1000 hours, in stark contrast to S-LLZO, which fails after 150 hours, accompanied by increasing overpotentials indicating uneven Li plating/stripping (Fig. 5b). Symmetrical cells with SF-LLZO separators consistently exhibit lower polarization than cells with S-LLZO, primarily due to the higher ionic conductivity of SF-LLZO pellets and thus greatly reduced ohmic losses, resulting in an ohmic voltage drop of approximately 0.05 V. In addition, the denser surface morphology of SF-LLZO pellets likely allows for more effective contact with the Li metal, reducing interfacial resistance and resulting in more homogeneous Li plating/stripping."

Reviewer #2

Remarks to the Author:

This study reports the detrimental effects and underlying mechanisms of metal segregation at grain boundaries in LLZO solid-state electrolytes. Based on these mechanistic insights, a universal strategy is proposed to achieve segregation-free grain boundaries via a rapid, precisely controlled sintering protocol that exploits the onset of solid-state softening, identified by an inflection in the densification curve. However, before the work can be considered for publication in Nature Communications, several issues need to be addressed.

Author response

We are grateful to the reviewer for the positive feedback and numerous constructive suggestions, which have contributed to improving the quality of the manuscript. A detailed response to the comments can be found below. The changes are highlighted in yellow in the revised version of the manuscript.

Comment 1

Do any metal compounds form at the grain boundaries when segregation occurs?

Response 1

We thank the reviewer for the insightful comments. In general, dopants with ionic radii and valence states comparable to those of the host species are preferred to ensure high solubility in the crystalline structure, such as Ta^{5+} (64 pm) comparable to Zr^{4+} (72 pm) in Ta-doped LLZO. Even for Al, whose ionic radius and charge state (Al^{3+}, 53.5 pm) differ substantially from those of Li^{+} (76 pm), previous studies have reported a solubility limit in $Li_{7-x}Al_xLa_3Zr_2O_{12}$ of up to ~0.4 (J. Mater. Chem. A 2022, 10, 21955-21972). In our composition ($Li_{6.45}Al_{0.05}La_3Zr_{1.6}Ta_{0.4}O_{12}$), the Al content is very low, making the formation of Al- or Ta-containing secondary phases at the GBs unlikely.

Furthermore, a secondary phase forming at the GBs would typically lead to a pronounced deterioration in ionic conductivity. In contrast, the ionic conductivity of our segregated LLZO remains ~0.6 mS cm^{-1} (Figure 2b, manuscript), which is comparable to the typical level reported for conventionally sintered LLZO with similar compositions. This observation suggests that no significant phase transformation occurs at the GBs. In addition, HAADF-STEM images show no obvious contrast difference between the GB core and the bulk region (Figure 1b, manuscript), further supporting the absence of distinct secondary phase formation at the GBs.

Comment 2

In line 127, the authors state: “These dark regions are assumed to be Li-rich phases, which appear dark in HAADF imaging due to the low atomic number of Li and are

probably formed during post-treatment." However, given the very low atomic number of Li (Z = 3), its contribution to HAADF contrast is negligible, making Li-rich phases difficult to directly identify by HAADF imaging alone.

Response 2

We thank the reviewer for bringing up this important point. As the reviewer correctly states, Li will contribute little to contrast in HAADF images, where image intensity scales with the square of atomic number. We posit here that the dark features are lithium-rich compounds precisely because they appear dark, indicating a region of lower average atomic mass along the optic axis. The best way to conclusively prove that these features are lithium rich is to utilize electron energy loss spectroscopy (EELS). Unfortunately, our samples contain Ta, which has an EELS edge which directly obscures any lithium signal which may be present. Therefore, we speculate that Li precipitation occurs due to surface charging of the sample during sample preparation. Li metal is highly reactive and can easily react with atmosphere during transport to form Li-containing compounds such as Li_2O, Li_3N and $LiCO_3$. In such cases, the heavier element (e.g., C, N and O) can produce a more pronounced HAADF contrast and thus indirectly affect the observed intensity. For example, Peng et al. reported Li growth on the LLZO surface induced by electron-beam charging during in situ TEM observation (Sci. Adv. 2023, 9, eabq3285), As shown in Figure 1 (below). Another possible scenario is Li precipitation may cause local structural collapse or nano porosity on LLZO, which could also lead to locally reduced HAADF contrast and thus dark features in the images. We have revised the corresponding discussion in manuscript.

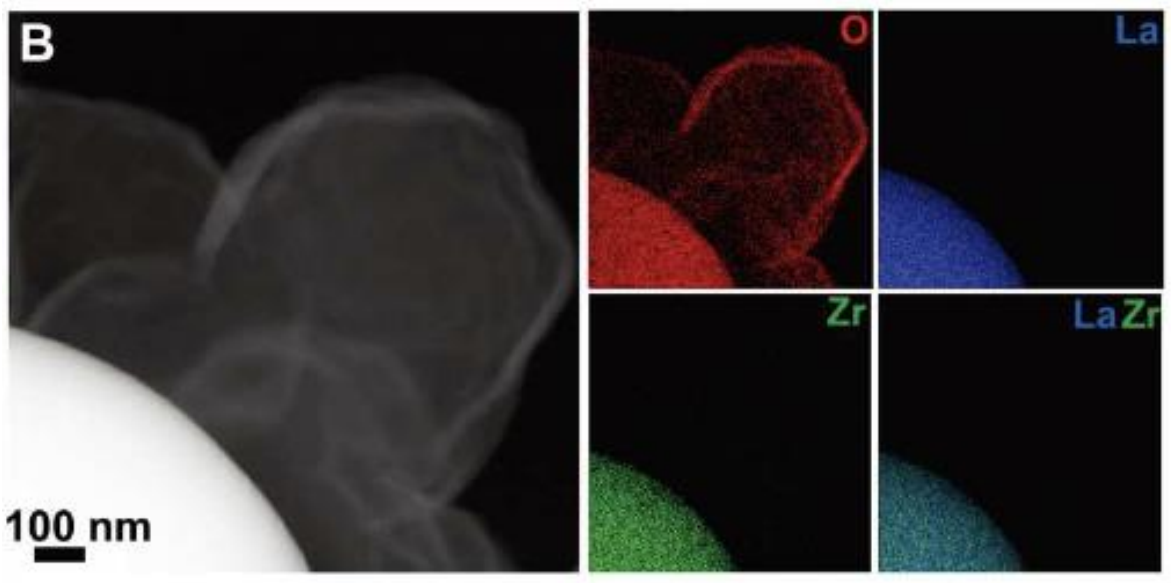

Figure 1. Representative HAADF-STEM image and the corresponding elemental maps of La, Zr, and O using Super-X EDS at cryogenic temperature (Sci. Adv. 2023, 9, eabq3285).

Changes to manuscript (pages 5 and 16)

"High-angle annular dark field scanning transmission electron microscopy (HAADF-STEM) shows the microstructure of a representative GB with a distinct GB core and dark contrast spots along the edges (Fig. 1b). These dark regions are hypothesized to be Li compounds (see discussion in a later section)."

"STEM characterizations of conventionally sintered LLZO doped with Al, Ta, or Ga

reveal the presence of dark spots along the GB edges in HAADF images (Fig. 1b and Supplementary Fig. 6b, c). We hypothesize that during specimen preparation (e.g., ion milling), surface charging of the sample promotes the precipitation of Li on the LLZO surface. The deposited Li is then likely to be rapidly oxidized, forming Li-containing compounds (e.g., Li_2O or Li_2CO_3) during sample transfer. These Li containing compounds consist of relatively light elements, or Li precipitation may induce local structural collapse and nano porosity, both of which would lead to reduced HAADF contrast and thus appear as dark spots in the images."

Comment 3

How did the authors confirm the formation of a lithium-rich phase, aside from HAADF imaging?

Response 3

We thank the reviewer for this important comment. In addition to HAADF-STEM contrast, we relied on the analysis of experimental results from various techniques in conjunction with the interpretation of similar observations in previously published studies.

In the initial phase of this work, we observed Li growth on the LLZO surface during SEM imaging, as shown in the Supplementary Fig. 6a. A similar effect has been described in previous studies (e.g., Joule, 2019, 3, 2030–2049 and Sci. Adv. 2023, 9, eabq3285). Actually this is a well-known phenomenon in LLZO. Since LLZO is an electronic insulator, it tends to become surface-charged under electron beam irradiation, which can induce Li^+ reduction and subsequent Li deposition. It should be noted that such Li growth is not observed under all conditions and strongly depends on factors such as the Li content in LLZO and the imaging parameters (e.g., accelerating voltage, beam current, and dwell time). During subsequent TEM characterization of the same samples, we also observed dark contrast spots on the LLZO surface. This phenomenon is very similar to the in-situ TEM observation described in Sci. Adv. 2023, 9, eabq3285 (Figure 1). In our work, the dark spots appeared more frequently near both sides of the GBs and resembled the Li precipitation behavior reported in Joule, 2019, 3, 2030-2049 (Figure 2, shown below), where the preferential occurrence of Li metal next to GBs during electron microscopy analysis was described. Furthermore, the occurrence of these deposits during TEM observation in our work shows a dependence on the crystallographic orientation of LLZO (Supplementary Fig. 6d-f).

Based on these observations, we suggest that the dark spots are likely induced by surface charging during TEM imaging and/or sample preparation processes, which promotes local Li precipitation onto the LLZO surface. It is expected that the deposited Li will be rapidly oxidized by the residual gases in the TEM chamber and/or during sample transfer, leading to the formation of Li-containing compounds.

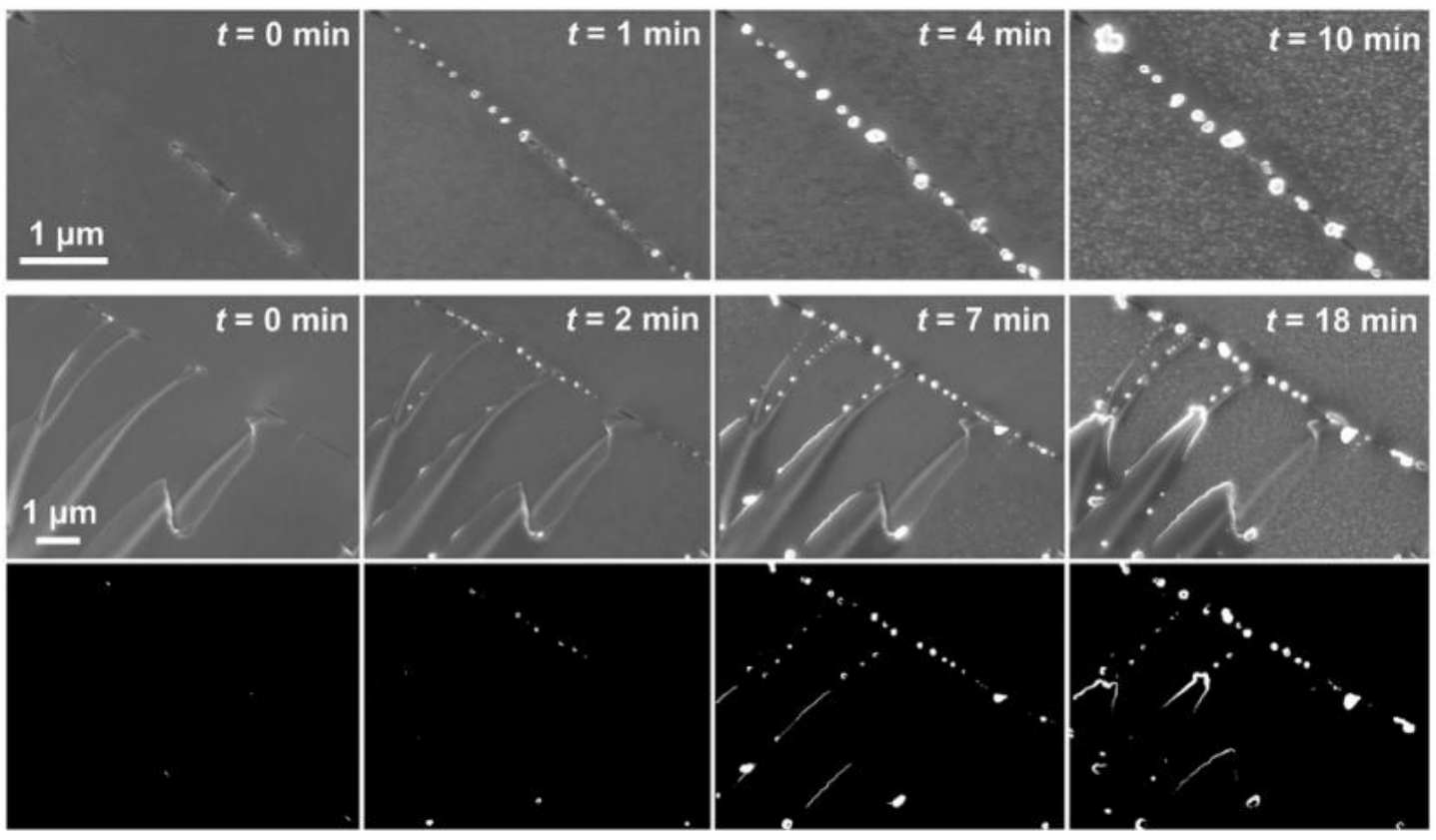

Figure 2. In situ lithium electrodeposition by electron injection (Joule, 2019, 3, 2030–2049).

We agree that the rationale for identifying these features as a Li-rich phase was not sufficiently explained in the main text and was only briefly mentioned in the caption of the respective figure. We have therefore revised and expanded the relevant discussion in the manuscript to improve clarity and enable readers to better understand our interpretation, as explained in more detail below.

Changes to manuscript (pages 5 and 16)

"High-angle annular dark field scanning transmission electron microscopy (HAADF-STEM) shows the microstructure of a representative GB with a distinct GB core and dark contrast spots along the edges (Fig. 1b). These dark regions are hypothesized to be Li compounds (see discussion in a later section)."

"STEM characterizations of conventionally sintered LLZO doped with Al, Ta, or Ga reveal the presence of dark spots along the GB edges in HAADF images (Fig. 1b and Supplementary Fig. 6b, c). We hypothesize that during specimen preparation (e.g., ion milling), surface charging of the sample promotes the precipitation of Li on the LLZO surface. The deposited Li is then likely to be rapidly oxidized, forming Li-containing compounds (e.g., Li_2O or Li_2CO_3) during sample transfer. These Li containing compounds consist of relatively light elements, or Li precipitation may induce local structural collapse and nano porosity, both of which would lead to reduced HAADF contrast and thus appear as dark spots in the images."

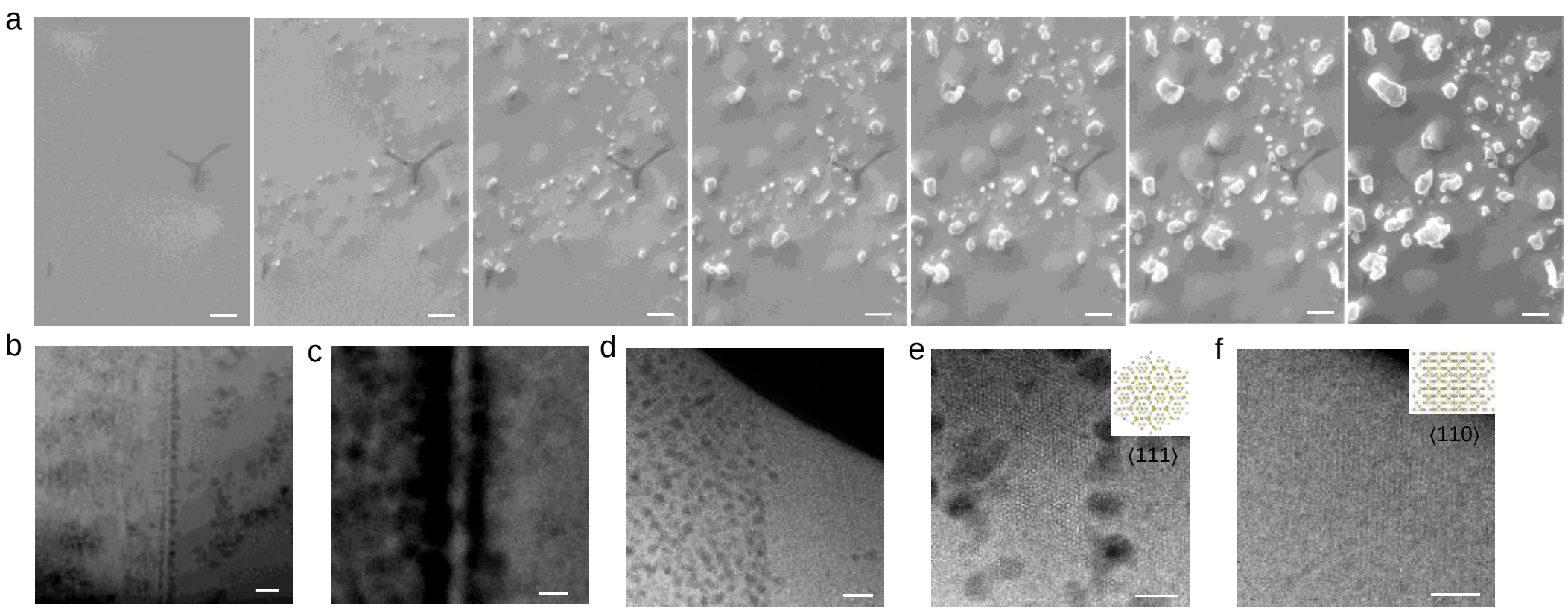

"**Supplementary Fig. 6** | **Li-rich compound formation.** **a**, Li metal formation from Al, Ta-doped LLZO in SEM with extended exposure time. Scale bars are 1 μm. With the extended exposure time, Li metal precipitates on the LLZO surface and gradually grows. **b**, Li compounds on Al, Ta-doped LLZO in HAADF-STEM. Scale bar is 40 nm. Black spots were observed along both sides of the GBs and in regions further away. These spots are hypothesized to be Li-containing compounds. This is because specimen preparation can promote Li formation on the LLZO surface, which is then expected to be rapidly oxidized during sample transfer, thereby forming Li-rich compounds. **c**, Li compound formation from Ga-doped LLZO in HAADF-STEM. Scale bar is 5 nm. Li compounds were observed to form along both sides of the GB, similar to the Al, Ta-LLZO. **d**-**f**, Li compound formation from Ga-doped LLZO in HAADF-STEM (**d**) (Scale bar is 10 nm), enlarged left side (**e**) (Scale bar is 5 nm) and enlarged right side (**f**) (Scale bar is 5 nm). Insets show lattice planes, and yellow and purple spheres represent La and Zr, respectively. The orientations of the grains forming either side of the GB were identified as ⟨ 111⟩ and ⟨110 ⟩. More Li compounds were observed on the ⟨111 ⟩ surface compared to ⟨110⟩, indicating that Li is more readily reduced from ⟨111⟩."

Comment 4

Why can LLZO with nearly no grain boundaries be prepared by FAST/SPS? Can such grain-boundary-free LLZO be considered a single crystal with some preferred orientation?

Response 4

By using the FAST/SPS process with an inflection point-guided sintering protocol described in our manuscript, we were able to obtain LLZO pellets with an extremely high relative density and effectively suppressed detrimental GB segregation. Electrochemical impedance spectroscopy (EIS) measurements of this material show only one arc due to ion transport in bulk LLZO material and virtually no GB impedance, so it effectively behaves like a single crystal. Nevertheless, the material remains polycrystalline and shows no obvious preferred orientation, as confirmed by the EBSD analysis (Figure 3).

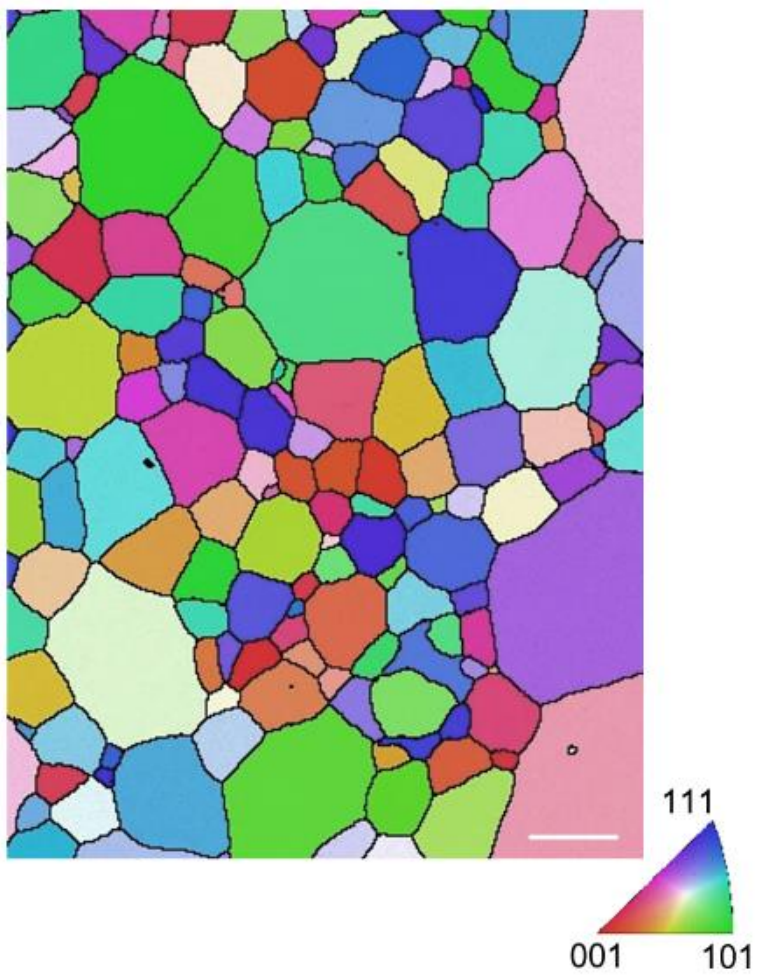

Figure 3. Representative EBSD images of SF-LLZO. (Figure 2d, manuscript)

Reviewer #3

Remarks to the Author:

This work elucidated the structural origins and electrochemical consequences of grain boundary segregation in Garnet $Li_7La_3Zr_2O_{12}$ (LLZO) electrolyte. Accordingly, a universal strategy was developed to achieve segregation-free grain boundaries through a rapid yet precisely controlled sintering protocol. However, significant flaws exist in the rigor of experimental design, the thoroughness of data analysis, and the reliability of conclusions, undermining the paper's scientific integrity and credibility. Therefore, the existing article must undergo thorough and rigorous revision to address these deficiencies.

Author response

We are grateful to the reviewer for the positive feedback and numerous constructive suggestions, which have contributed to improving the quality of the manuscript. A detailed response to the comments can be found below. The changes are highlighted in yellow in the revised version of the manuscript.

Comment 1

The author determines the “solid softening point” based on the inflection point of the displacement curve as the criterion for the sintering endpoint, claiming this strategy is applicable to various ceramic electrolytes. However, the definition of the sintering endpoint in the paper is ambiguous and lacks sufficient theoretical support. Furthermore, materials with differing chemical compositions, powder particle sizes, and doping types will inevitably exhibit distinct softening behaviors (i.e., inflection point temperatures and displacement change rates). How can one “pre-determine” or “real-time judge” the inflection point for new materials based on limited initial experimental data to

demonstrate the predictability and universality of this strategy?

Response 1

We thank the reviewer for the insightful comments. We would like to clarify that the method described in our manuscript does not introduce a new sintering theory, but rather provides a phenomenological and process-oriented strategy for obtaining highly densified ceramics by utilizing the onset of solid softening and viscous deformation at elevated temperatures.

We agree that different materials, compositions, and powder particle sizes may exhibit different softening behaviors and thus different critical temperatures, which are difficult to predict a priori. It is important that sample displacement is monitored in real time during the sintering process so that we can continuously track the densification behavior and stop the sintering process at the desired stage, followed by immediate rapid cooling.

In practice, although the exact solid-softening point is not known in advance, the approximate melting point of a given ceramic material is usually available, and the solid-softening point typically occurs below the melting point. During densification, gases trapped between the particles are expelled. Accordingly, a pronounced pressure peak can be observed in the chamber pressure curve (red curves in Figure 4 below for three representative ceramics). After this peak, the curve approaches an inflection point. Meanwhile, the displacement rate gradually decreases toward zero as densification saturates and then increases again once the softened sample begins to undergo extrusion/flow deformation. Therefore, as soon as we detect that the displacement rate begins to increase, we immediately stop heating and pressurization and switch the program to the cooling stage. In other words, the program is stopped shortly after the inflection point to obtain segregation-free LLZO. It is noteworthy that this inflection point is clearly recognizable, as both the first and second derivatives of the displacement curve approach zero at this point, allowing for reliable identification during real-time monitoring. Therefore, this strategy does not rely on a predetermined sintering temperature and can be widely applied to various materials to achieve rapid densification.

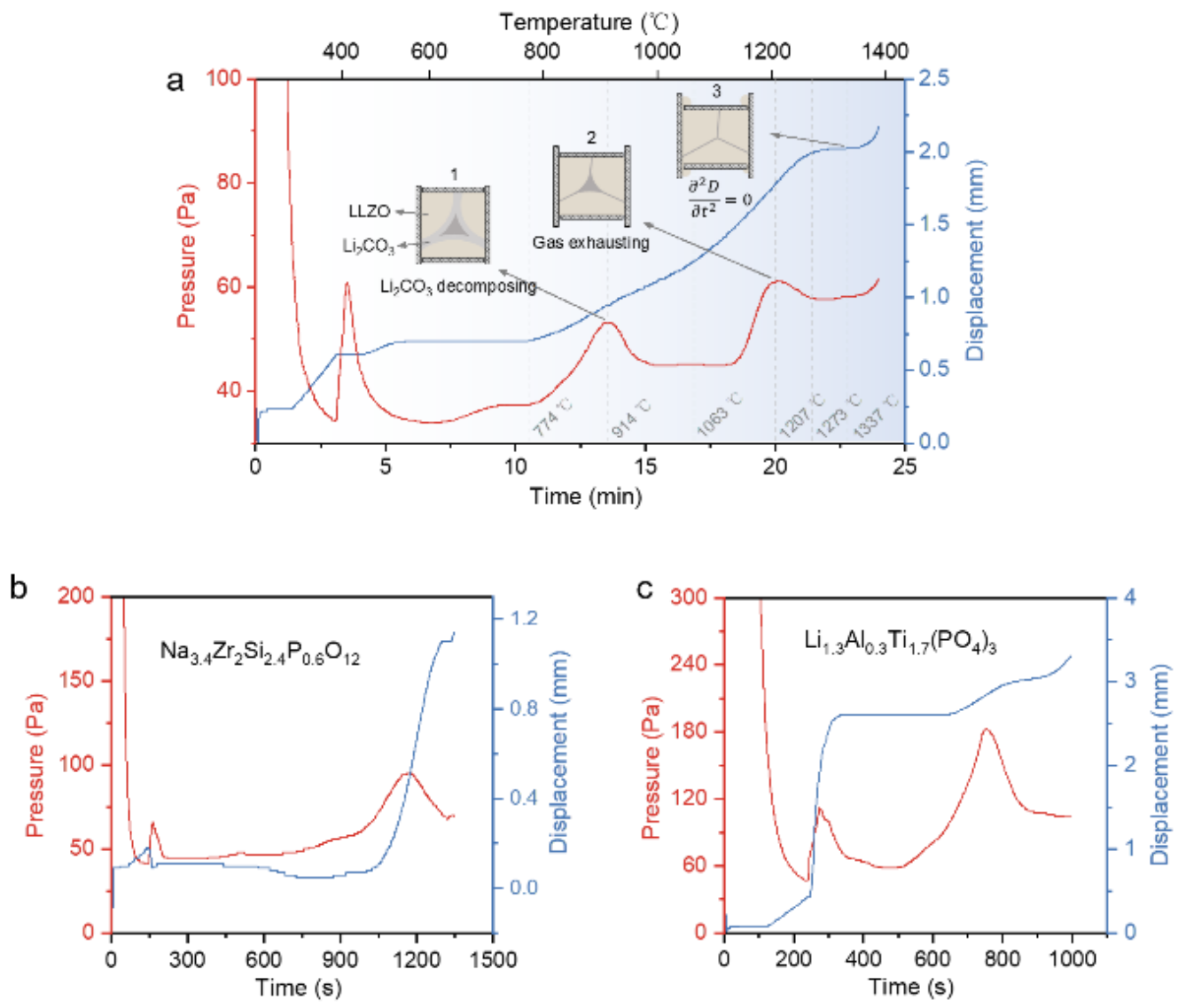

Figure 4. Displacement and chamber pressure records of LLZO (a) and other two electrolyte ceramics (b and c) during FAST/SPS process

We have revised the relevant section in the manuscript to clarify that the samples were obtained not exactly at the inflection point, but immediately after it:

Change to manuscript (page 10)

"The process exhibits a mathematically identifiable inflection point ($\partial^2 \Delta D \ / \ \partial t^2 = 0$, where D is the displacement) after the gas has completely escaped. This point represents the critical softening point of the solid phase and can be determined individually for different ceramic powders with different compositions (Supplementary Fig. 3c). In our sintering protocol, the inflection point is used as a practical indicator of the optimal endpoint, and the sintering process is terminated immediately after this point to obtain nearly full dense pellets."

Comment 2

This study lacks critical characterization of the dendrite failure mechanism in symmetric cell testing. Following CCD testing and cycling, the authors recorded only voltage signals. Voltage signals alone cannot determine which stage of dendrite growth is inhibited. Cross-sectional SEM images of the cycled battery must be provided to directly observe whether dendrites grow along grain boundaries, through grains, or nucleate at the electrode/electrolyte interface. Otherwise, improved CCD and cycle life cannot be directly attributed to "inhibited lithium nucleation along grain boundaries."

Response 2

We thank the reviewer for the constructive comment. We fully agree that a post-mortem analysis is essential, as it can provide the most direct evidence to support our conclusions. In the revised manuscript, we have included the results of the post-mortem SEM characterization of the cross sections of SF-LLZO and S-LLZO after dendrite penetration, as shown in the figures below. The results strongly support the Li growth

mechanisms discussed in manuscript. In SF-LLZO, Li dendrite penetration is accompanied by the formation of cracks that extend across the entire thickness of the pellet without following the microstructure of the LLZO grains, which is similar to the performance of single-crystal LLZO. This behavior differs significantly from S-LLZO, where Li dendrite grows as filaments along the GBs, consistent with the Li nucleation and growth along GBs proposed by Liu et. al (Nat. Mater. 2021, 20, 1485-1490). A detailed discussion is included in the revised version:

Chang to manuscript (page 22)

Post-mortem analysis of the cells after a hard short circuit also shows that segregated and segregation-free LLZO pellets exhibit remarkable differences in Li penetration mechanisms. In S-LLZO pellets, dark areas can be observed on the surface where dendrites form[21] (Fig. 5d). Local cross-sectional SEM images of this area show that Li grows along the GBs and forms interconnected filaments (Fig. 5e). This is consistent with the reported bulk-mediated dendrite growth mode, where Li nucleation and interconnection occur along the GBs[13]. In contrast, SF-LLZO exhibits a distinctly different dendrite growth behavior, in which Li dendrites penetration is accompanied by crack formation (Fig. 5f and g). The cracks run either slanted or almost perpendicular to the surface through the entire pellet thickness without following the microstructure of the LLZO grains. The cracks are filled with lithium, which can be seen along the crack length and along the fractured surface (Fig. 5h-j and Supplementary Fig. 8), as confirmed by EDS mapping, where C and O are enriched (Li was oxidized to Li_2CO_3 during sample transfer), while Zr and La (LLZO) are absent (Supplementary Fig. 8). This behavior is very similar to the performance reported for single-crystal LLZO[53], suggesting a surface-initiated dendrite growth mechanism in which dendrites propagate from one electrode to the other through SF-LLZO separator, while the high stress concentrated at the dendrite tips promotes crack initiation and development[54,55]. The distinctly different dendrite propagation behavior can be attributed to the differences in the structure of segregated and segregation-free GB discussed above, including differences in the local distribution of Li-ions and electronic conductivity. We therefore propose that the dendrite tolerance of polycrystalline LLZO separators is mainly determined by the morphology (relative density) and electronic conductivity of the GBs, the latter being strongly influenced by the dopants and the element segregation. After eliminating element segregation in SF-LLZO, the dendrite tolerance is largely determined by the mechanical properties (mainly fracture toughness) of the LLZO crystal structure and is close to maximum value expected for this material. A further increase in CCD would primarily require optimization of the mechanical properties of the polycrystalline LLZO scaffold and improving its ability to relief the stress occurring during Li plating, with research directions including optimization of particle size/particle size distribution and GB modification to improve the total ductility.

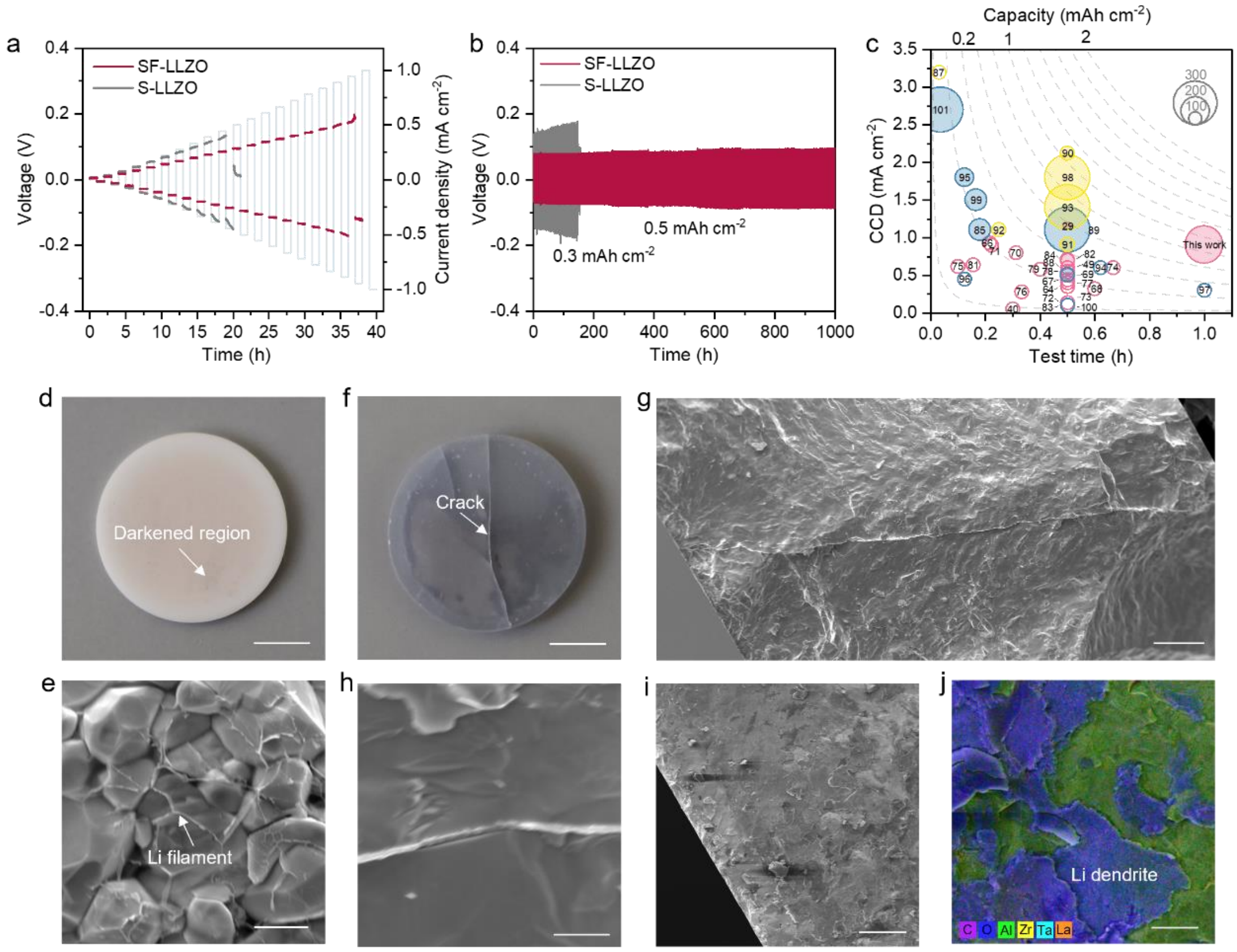

“**Fig. 5 | Dendrite tolerance assessment. a**,**b**, CCD (**a**) and galvanostatic cycling (**b**) measurements of S- and SF-LLZO pellets at RT. **c**, Comparison of reported CCDs and CCD capacities of LLZOs, together with total accumulated capacities during galvanostatic cycling (bubbles). Pink, blue, and yellow bubbles denote pristine, GB-engineered, and both GB- and surface-modified LLZO, respectively. Filled bubbles indicate accumulated capacities and open bubbles indicate <50 mAh cm^{-2} or not provided. Current densities are normalized to the effective Li/LLZO contact area. References are listed in Supplementary Table 7. **d**,**e**, Post-mortem analysis after dendrite penetrating S-LLZO (removing one Li electrode), shown by an optical top-view image (**d**) and local cross-sectional SEM images at dark regions (**e**). Scale bars are 3 mm (**d**), 5 μm (**e**). **f-j**, Post-mortem analysis after dendrite penetrating SF-LLZO (removing one Li electrode), shown by an optical top-view image (**f**), local cross-sectional (**g**,**h**), and fractured SEM image and EDS overlay mapping at cracks (**j**,**j**). Scale bars are 3 mm (**f**), 100 μm (**g**), 5 μm (**h**), 50 μm (**i**) and 10 μm (**j**).”

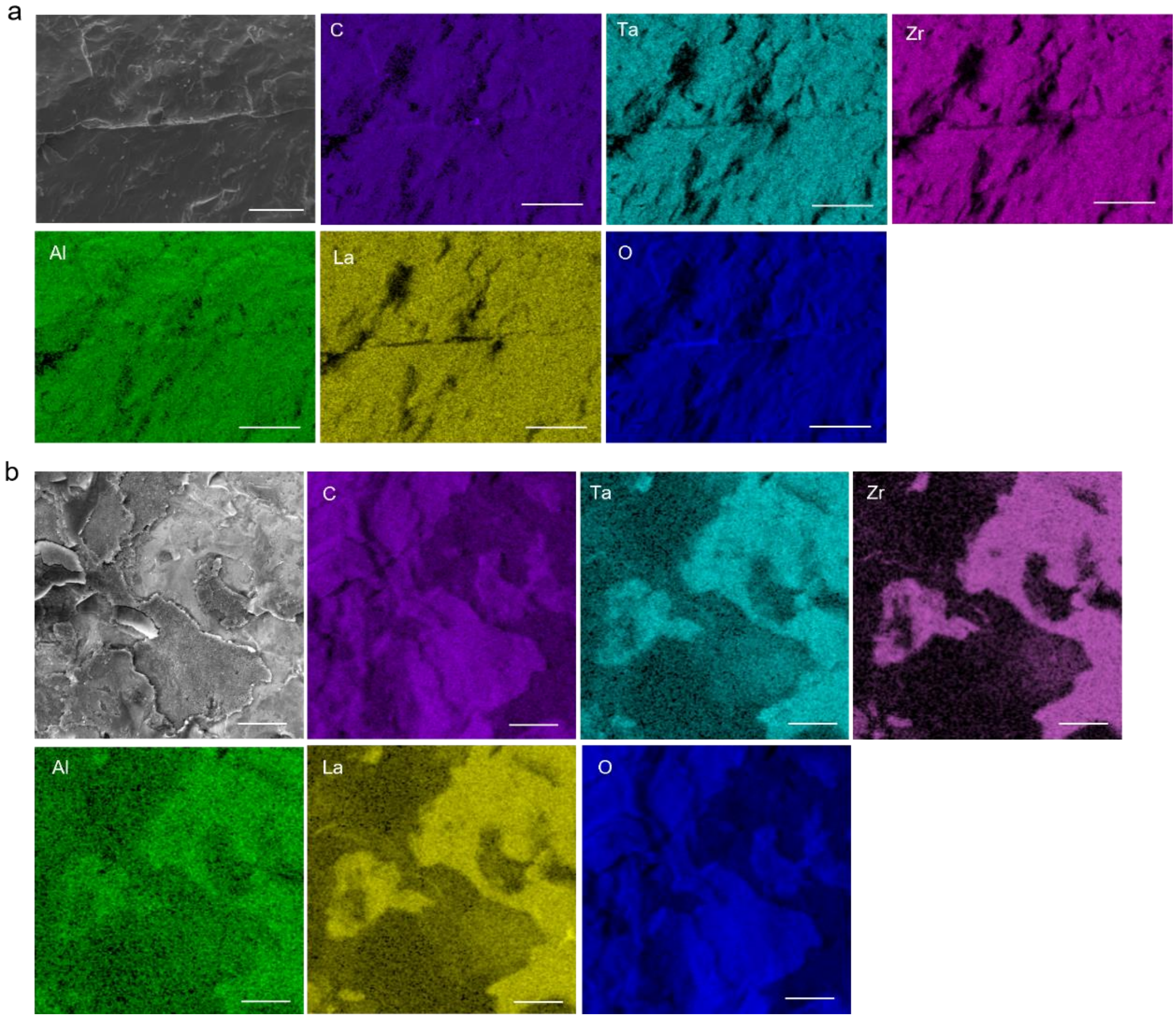

"**Supplementary Fig. 8 | Post-mortem analysis after dendrite penetration. a**,**b**, Local cross-sectional (**a**), and fractured (**b**) EDS mappings at cracks after dendrite penetrating SF-LLZO. Scale bars are 50 μm (**a**) and 10 μm (**b**)."

Comment 3

The evidence chain supporting the "no segregation" conclusion in the article is incomplete. The authors hastily claim "no elemental segregation" based solely on HAADF-STEM/EDS mapping of one or a few grain boundaries, rendering the conclusion extremely hasty and highly unscientific. The paper neither specifies the detection limits of the EDS mapping nor provides details on the specific grain boundary observation areas or the number of grain boundaries examined. It offers no detailed description of the distribution within grain boundaries, and the statistical sample size is severely inadequate. Therefore, the conclusion of no segregation cannot be blindly drawn. This demonstrates a lack of rigor in the paper.

Response 3

We agree with the reviewer that further statistical analysis is needed to support our claims. We performed this analysis using the three-box method for all grain boundary

scans collected for the two samples to quantify the grain boundary excess of La, Ta, and Zr. This method also provides the necessary sensitivity and shows that the results are statistically significant. In addition, we provide additional EDS images for more GBs for SF-LLZO. This data has been added to the supplementary information as described below.

Change to supplementary information (page36, supplementary)

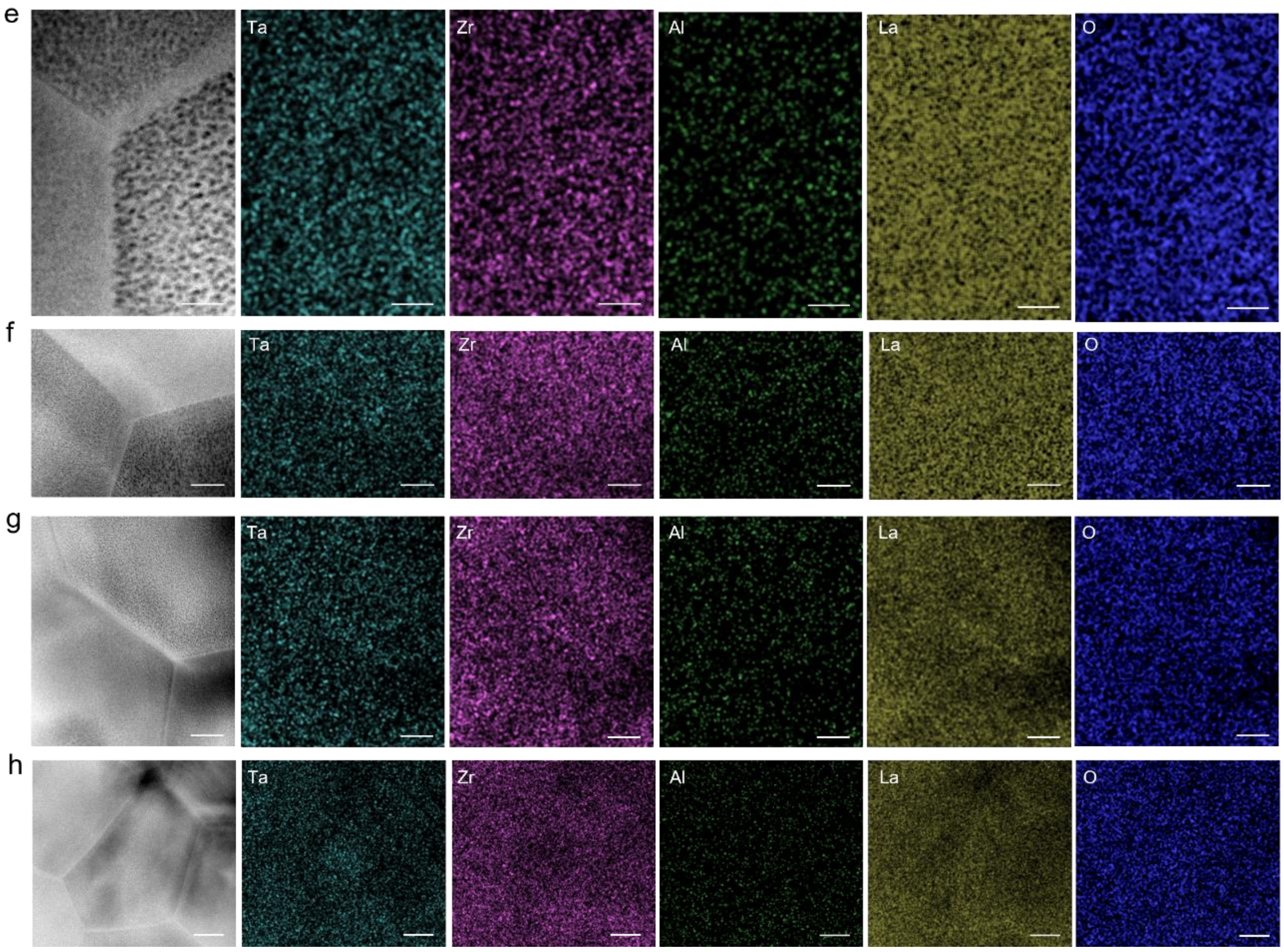

Supplementary Fig. 4 | Characterizations of SF-LLZO. e-h, Representative HAADF-STEM image and STEM-EDS mappings of the GB of SF-LLZO. Scale bars are 200 nm (**e**), 500 nm (**f**), 2 µm (**g**,**h**).

Supplementary Tab. 4 | Quantitative statistical analysis of excess elements. Grain boundary excess values (in atoms/nm^2) and measurement detection limits for S-LLZO and SF-LLZO, measured by STEM/EDS. The aluminum concentration was too low to be quantitatively analyzed with EDS. The three box method has been used to quantify the grain boundary segregation in each sample (J. Eur. Ceram. Soc., 2018, 38, 1829-1835; Materialia, 2020, 14, 100890). More than 20 GBs were analyzed for the SF-LLZO, and five for the conventionally sintered sample. The average GB excess values for each element are shown in the table, along with the detection limit of the method for each element. The standard error of the mean was used to calculate errors for the GB excess values.

Materials	Element	GB excess	Detection limit
S-LLZO	La	-8.0 ± 9.3	0.4
	Ta	2.5 ± 0.5	0.2
	Zr	-2.3 ± 0.4	0.1
SF-LLZO	La	0.4 ± 0.2	0.1
	Ta	0.3 ± 0.2	0.1
	Zr	0.2 ± 0.1	0.05

Furthermore, changes were made to the main text to correct linguistic inaccuracies. The description of segregation behavior was changed to “no statistically significant segregation is detected within the measurement accuracy”. This sentence more accurately expresses that it cannot be claimed that ‘no segregation’ exists, but only that no statistically significant segregation was observed.

Additional evidence for suppressed segregation in SF-LLZO at the macroscopic level is provided by the EIS results, as shown in Figure 5 below. It is noteworthy that SF-LLZO does not exhibit a discernible GB impedance arc. However, after annealing, when segregation reappears (TEM data in supplementary Fig. 4e), a distinct GB-related impedance arc becomes visible, indicating the appearance of GB resistance.

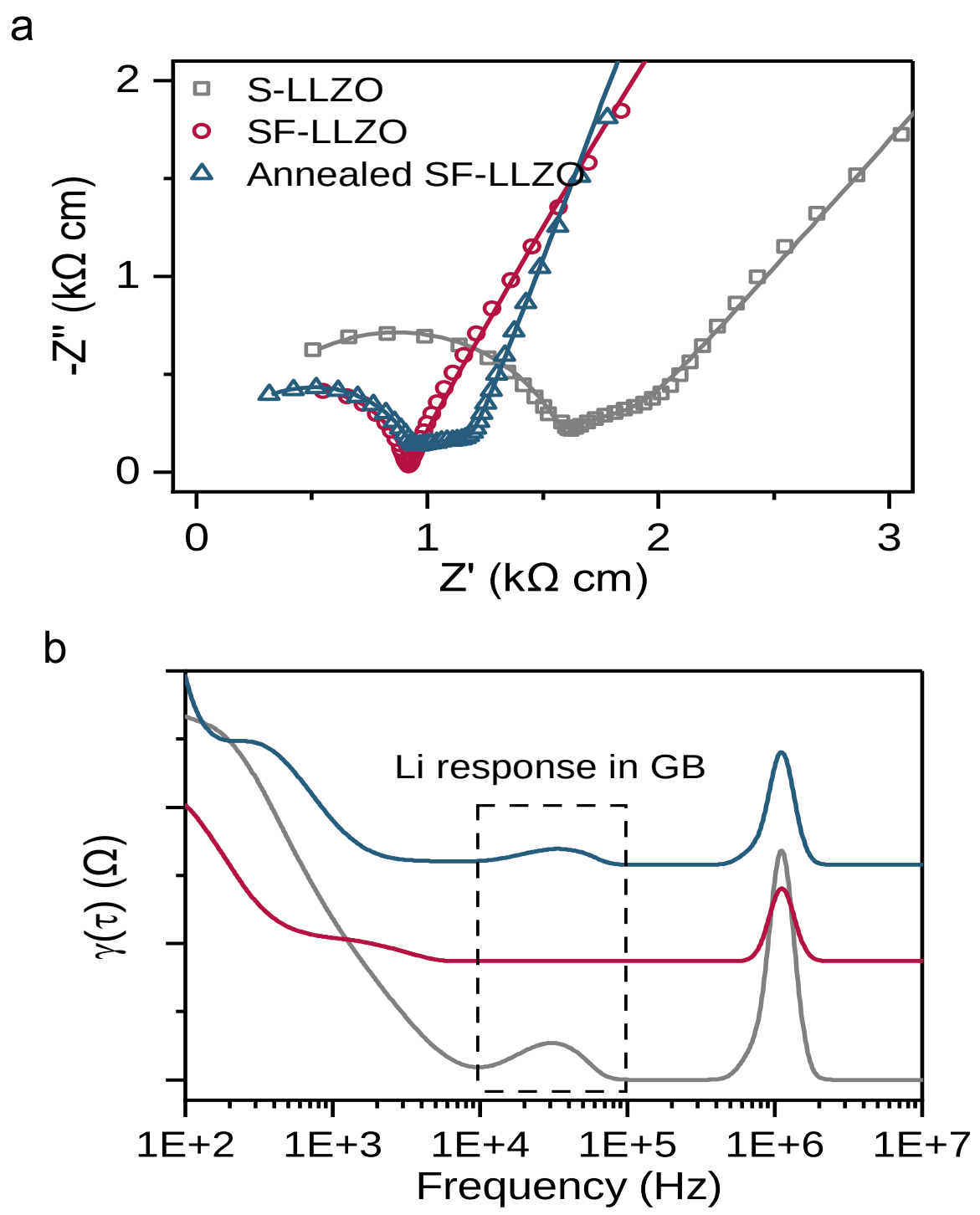

Figure 5. EIS (a) and corresponding DRT analysis (b) of S-, SF- and annealed SF-LLZO measured from 7 MHz to 1 Hz at RT. (Figure 3a, b, manuscript)

In addition, our MLFF-MD simulations suggest that the thermodynamic driving forces for dopant segregation across different GB configurations exhibit a converging (narrowly distributed) trend, as shown in Figure 6 below. Therefore, although TEM is inherently a local characterization technique, observations at a limited number of representative GBs are nevertheless meaningful and can provide relevant insights into general segregation behavior.

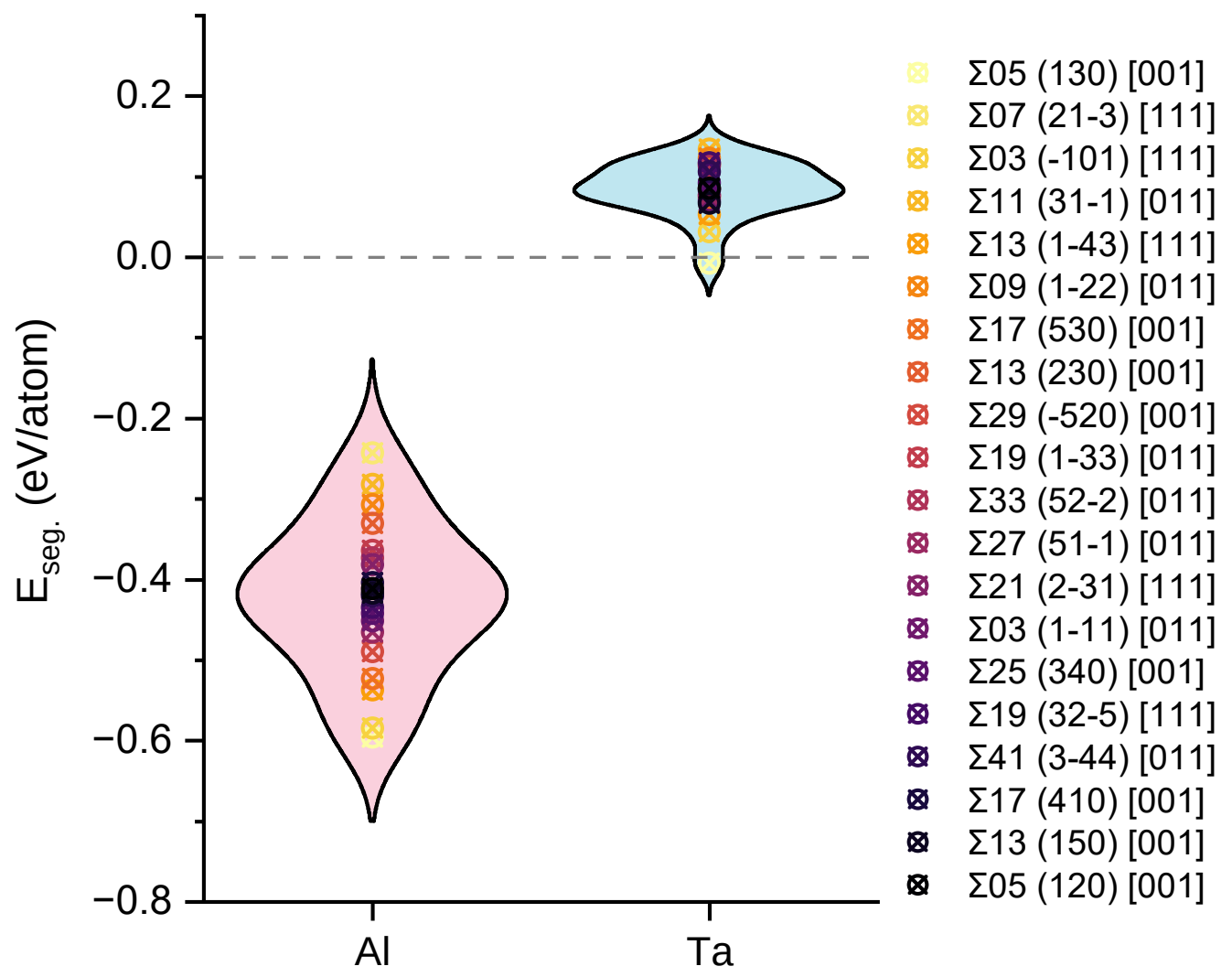

Figure 6. Statistical results of MLFF-MD simulation on segregation energies of Al and Ta at 20 GBs. Each segregation energy was calculated across 50 randomly doped structures. (Figure 1d, manuscript)

Comment 4

The article lacks comparison with existing advanced grain boundary engineering strategies. Numerous reports in recent literature have successfully enhanced LLZO performance through "grain boundary engineering" approaches such as grain boundary doping and second-phase modification (NPG Asia Mater 2024 16(1), 1884-4049; Nat. Mater. 2025, 1476-1122; Nat Commun 2025 16(1), 2041-1723). The authors' work essentially focuses on "avoiding the formation of harmful grain boundaries." However, the paper only compares results with unmodified LLZO, neglecting representative data points from recent studies that achieved superior performance through active "grain boundary engineering." Such comparisons merely demonstrate the advantages of conventional grain boundary modification without highlighting the innovation of the grain boundary modification in this paper. Therefore, the paper should not only contrast with "unmodified" LLZO but also discuss recent boundary engineering approaches. This would emphasize the relative advantages of the presented method in terms of performance, process complexity, and cost.

Response 4

We thank the reviewer for this constructive suggestion. We have added the references

suggested by the reviewer as well as several representative studies on grain boundary engineering from recent years. It is important to note that the CCD performance of LLZO is determined not only by the chemistry or structure of the grain boundaries, but is also significantly influenced by the properties of Li|LLZO interface. For example, in a recent paper (Nat. Mater. 2025, 1476–1122), the authors modified the LLZO surface with W, enabling a CCD of 1.4 mA cm^{-2} even for unmodified LLZO. The duration of the CCD test, also referred to as capacity, also has a decisive influence on the CCD. Therefore, in our updated CCD comparison, we have retained the test time and explicitly labeled the modification strategies used in individual literature reports, as well as provided a more comprehensive discussion of the presented method in terms of electrochemical performance, process complexity, and cost. The revised figure, table, and corresponding manuscript text are shown below.

Change to manuscript and supplementary (page 21, 22, manuscript; page 38, supplementary)

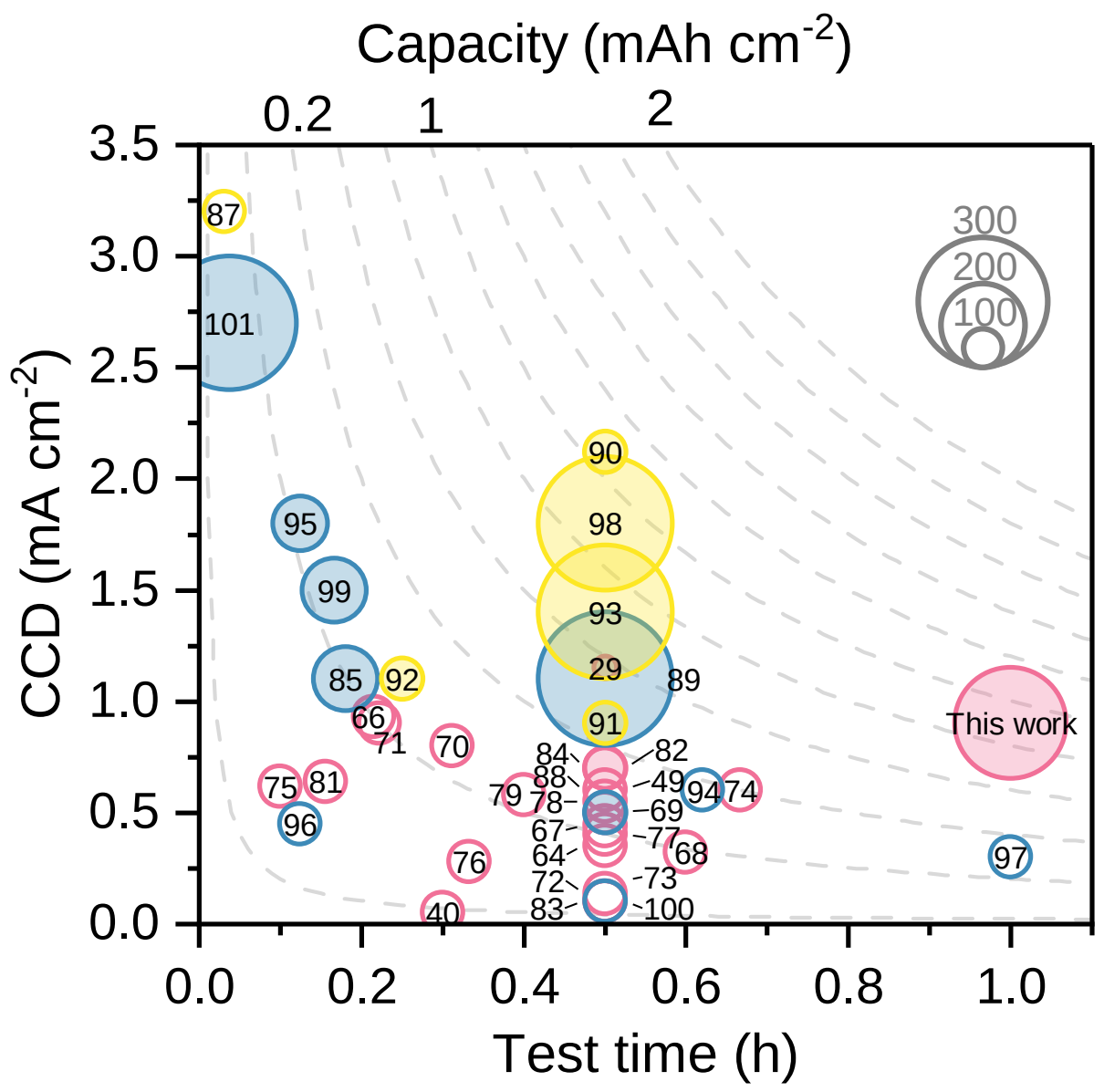

"Figure 5c. Comparison of reported CCDs and CCD capacities of LLZOs, together with total accumulated capacities during galvanostatic cycling (bubbles). Pink, blue, and yellow bubbles denote pristine, GB-engineered, and both GB- and surface-modified LLZO, respectively. Filled bubbles indicate accumulated capacities and open bubbles indicate <50 mAh cm^{-2} or not provided. Current densities are normalized to the effective Li/LLZO contact area. References are listed in Supplementary Tab. 7."

"**Supplementary Tab. 7 | Comparison of stripping/plating capacities.** Comparison of reported CCDs and CCD capacities of pristine (unmodified) LLZOs, as well as total accumulated capacities during galvanostatic cycling."

Materials	CCD (mA cm^{-2})	CCD test time (h)	Accumulated cycling capacities (mAh g^{-1})	Modifications	Temp (℃)	Ref.
$Li_{6.76}Al_{0.24}La_3Zr_2O_{12}$	0.35	0.5	~6.25		25	64
$Li_{6.45}Al_{0.05}La_3Zr_{1.6}Ta_{0.4}O_{12}$	0.3185	0.5			50	65
$Li_{6.25}La_3Zr_2Al_{0.25}O_{12}$	0.93	0.215			25	66
$Li_{6.25}La_3Zr_2Al_{0.25}O_{12}$	0.05	0.3			30	40
Ta-doped LLZO	0.44	0.5			RT	67
$Li_{6.1}Ga_{0.3}La_3Zr_2O_{12}$	0.32	0.6	3		25	68
$Li_{6.5}La_3Zr_{1.5}Ta_{0.5}O_{12}$	0.5	0.5	~25		RT	69
$Li_{6.5}La_3Zr_{1.5}Ta_{0.5}O_{12}$	0.8	0.3125			RT	70
$Li_{6.5}La_3Zr_{1.5}Ta_{0.5}O_{12}$	0.9	0.222			RT	71
$Li_{6.5}La_3Zr_{1.5}Ta_{0.5}O_{12}$	6.0	0.0333	702		60	71
Al-doped LLZO	0.134	0.5			RT	72
$Li_{6.5}La_3Zr_{1.5}Ta_{0.5}O_{12}$	0.1	0.5			25	73
$Li_{6.5}La_3Zr_{1.5}Ta_{0.5}O_{12}$	0.6	0.667			25	74
$Li_{6.25}Al_{0.25}La_3Zr_2O_{12}$	0.6	0.5			RT	49
Al-doped LLZO	0.62	0.1			RT	75
			~260		70	75
$Li_{6.4}Ga_{0.2}La_3Zr_2O_{12}$	0.28	0.333			20	76
$Li_{6.5}La_3Zr_{1.5}Ta_{0.5}O_{12}$	0.4	0.5	~7.5		25	77
$Li_{6.25}Ga_{0.25}La_3Zr_2O_{12}$	0.55	0.5				78
$Li_{6.1}Ga_{0.3}La_3Zr_2O_{12}$	0.58	0.4	6		RT	79
Al-doped LLZO	12.5	0.08	125		75	80
Ta-doped LLZO	1.15	0.5	~56.25		25	29
Al-doped LLZO	0.64	0.156	~28.75		RT	81
$Li_{6.25}Ga_{0.25}La_3Zr_2O_{12}$	0.7	0.5	100		RT	82
Ta-doped LLZO	0.1	0.5	15		RT	83

$Li_{6.6}La_3Zr_{1.6}Ta_{0.4}O_{12}$	0.7	0.5	6		RT	84
Ta-doped LLZO	1.1	0.18	150.4	GB engineering (LiF)	RT	85
$Li_{6.1}Ga_{0.3}La_3Zr_2O_{12}$			15	GB engineering (Li_2O-B_2O_3-Al_2O_3)	RT	86
$Li_{6.4}La_3Zr_{1.4}Ta_{0.6}O_{12}$	3.2	0.03125		GB engineering (ZrO_2) and surface modification (W)	RT	87
$Li_{5.9}Ga_{0.2}La_3Zr_{1.5}Ta_{0.5}O_{12}$	0.5	0.5	100	GB engineering ($LiGaO_2$)	RT	88
Ta-doped LLZO	1.1	0.5	300	GB engineering (Li_3BO_3)	RT	89
$Li_{6.5}La_3Zr_{1.5}Ta_{0.5}O_{12}$	2.12	0.5	75	GB engineering (La_2O_3) and surface modification (Au)	RT	90
$Li_{6.4}La_3Zr_{1.4}Ta_{0.6}O_{12}$	0.9	0.5	75	GB engineering (Li_2O) and surface modification (Au)	RT	91
Ta-doped LLZO	1.1	0.25	75	GB engineering (TiO_2) and surface modification (Au)	RT	92
$Li_{6.5}La_3Zr_{1.5}Ta_{0.5}O_{12}$	1.4	0.5	300	GB engineering ($Li_6Zr_2O_7$) and surface modification (Au)	RT	93
$Li_{6.5}La_3Zr_{1.5}Ta_{0.5}O_{12}$	0.6	0.62		GB engineering (Li_2O)	RT	94
F-doped LLZO	1.8	0.125	130	GB engineering	RT	95

				($LiAlO_2$)		
$Li_{6.5}La_3Zr_{1.6}Ta_{0.4}O_{12}$	0.45	0.125	50	GB engineering ($LiAlO_2$)	30	96
$Li_{6.4}La_3Zr_{1.4-x}Mn_xTa_{0.6}O_{12}$	0.3	1		GB engineering (La_4LiMnO_8	RT	97
$Li_{6.5}La_3Zr_{1.5}Ta_{0.5}O_{12}$	1.8	0.5	300	GB engineering (Li_2TiO_3) and surface modification (Au)	RT	98
Ta-doped LLZO	1.5	0.167	150	GB engineering ($LiGaO_2$)	30	99
$Li_{6.25}Al_{0.25}La_3Zr_2O_{12}$	0.1	0.5	21	GB engineering (LiF, Li_xAlO_y)	RT	100
$Li_{6.4}La_3Zr_{1.4}Ta_{0.6}O_{12}$	2.7	0.037	300	GB engineering ($Li/BaTiO_3$)	RT	101

"Compared with state-of-the-art reports, SF-LLZO outperforms most pristine LLZO electrolytes (Fig. 5c and Supplementary Tab. 7). Previous studies have shown that introducing electronically insulating phases at GBs (e.g., LiF or ZrO_2) via GB engineering, or employing lithiophilic interlayers to modify the Li|LLZO interface, can significantly improve the CCD performance (Fig. 5c). Notably, even when benchmarked against these advanced modification strategies, SF-LLZO still exhibits promising CCD capacity and cumulative capacity during galvanostatic cycling (Fig. 5c). Moreover, SF-LLZO is achieved through an ultrashort high-temperature process without introducing additional modification materials, offering clear advantages in terms of energy cost and process simplicity."

Comment 5

The analysis of ion conductivity activation energy in the paper lacks depth. The authors point out that the total activation energy of SF-LLZO (0.427 eV) is nearly identical to the grain activation energy of S-LLZO (0.429 eV), using this similarity as evidence that grain boundary impedance is negligible in SF-LLZO. However, for polycrystalline ceramics, the total activation energy represents a weighted average of bulk and grain boundary conduction pathways. Directly equating the total activation energy to the grain boundary activation energy is overly simplistic. More precise activation energy data should be obtained through more accurate models or by performing a more refined distribution of relaxation time (DRT) analysis of the EIS data.

Response 5

As recommended, before analyzing the activation energies, we first compared the EIS spectra of SF-LLZO and S-LLZO and further performed DRT analysis to examine their GB resistance differences (Figure 3a, b, manuscript). We fitted and separated the bulk and GB contributions. In addition, the extracted capacitance values were used to distinguish the GB-related response (page 12, manuscript). It is noteworthy that no discernible semicircle associated with the GB resistance can be resolved in the EIS spectra of SF-LLZO. Consistent with this, the DRT analysis (Figure 3b, manuscript) shows no peak in the frequency range typically associated with Li-ion transport across GBs. Therefore, we conclude that the GB impedance in SF-LLZO is nearly identical to the bulk response (i.e., the GB contribution is negligible within the detection limit).

Since the GB impedance of SF-LLZO cannot be reliably separated by either EIS fitting or DRT (probably falling below the experimental resolution), we determined only the total activation energy of SF-LLZO in the subsequent activation energy analysis. In contrast, for S-LLZO, both bulk and GB impedance arcs are clearly detectable, allowing us to extract the activation energies of the grain and GB contributions separately. The total activation energy of SF-LLZO (0.427 eV) is nearly identical to the bulk activation energy of S-LLZO (0.429 eV), which serves as additional indirect evidence for the minimal GB resistance in SF-LLZO.

Comment 6

The core comparative experiments are missing from the article. The most critical performance comparison throughout the paper (between S-LLZO and SF-LLZO) relies on samples made from identical powders but using different sintering techniques (conventional sintering and FAST/SPS). The inherent electric field, pressure, and reducing atmosphere of FAST/SPS differ from conventional sintering, and these factors themselves may independently influence grain boundary structure and performance. A critical control experiment must be added: prepare a “FAST/SPS-slow firing” sample using the same FAST/SPS equipment but simulating conventional sintering with a ‘slow’ heating ramp and extended soak time. Only by comparing with this control sample can the performance improvement be attributed to “time reduction/kinetic suppression” rather than other effects inherent to the FAST/SPS technology itself.

Response 6

We thank the reviewer for this valuable suggestion. We fully agree that such an experiment would be very informative, as it would allow for a more direct comparison between FAST/SPS and conventional sintering. However, it should be noted that the conditions of conventional sintering and FAST/SPS process differ significantly, so adjustment should be made to avoid additional artifacts and make this comparison more meaningful. Unlike conventional sintering, which is performed in air at ambient pressure, the chamber pressure in the FAST/SPS process is typically maintained at ~40 Pa and argon is introduced as a protective atmosphere to protect the internal graphite

tools. While these conditions favor rapid densification, prolonged FAST/SPS processing can lead to material degradation. Based on our repeated experiments, we have found that the practical upper limit for the FAST/SPS sintering is ~2 h, even under rapid heating conditions; for longer dwell times, XRD analysis revealed phase degradation of LLZO. Therefore, it is not possible to completely reproduce the heating rate and long dwell time used in conventional sintering (typically ~12 h) within the FAST/SPS setup. Instead, we used a slower heating rate (10 K min^{-1}) and a prolonged dwell time of 1 hour in a FAST/SPS process as a reference test to reproduce slow sintering conditions comparable to conventional sintering. Annealing of the SF-LLZO sample already described in our manuscript was used as a second reference test (annealed SF-LLZO). The EIS results (Figure 7a, b) clearly show that both reference samples exhibit a GB-related impedance arc that is not present in SF-LLZO. Furthermore, the sample sintered slowly in FAST/SPS shows a secondary phase at the GBs, which can be distinguished by the distinctly dark regions in the HAADF image (Figure 7c). The results obtained provide additional experimental evidence that the segregation behavior is highly time-dependent and is kinetically controlled under prolonged thermal exposure.

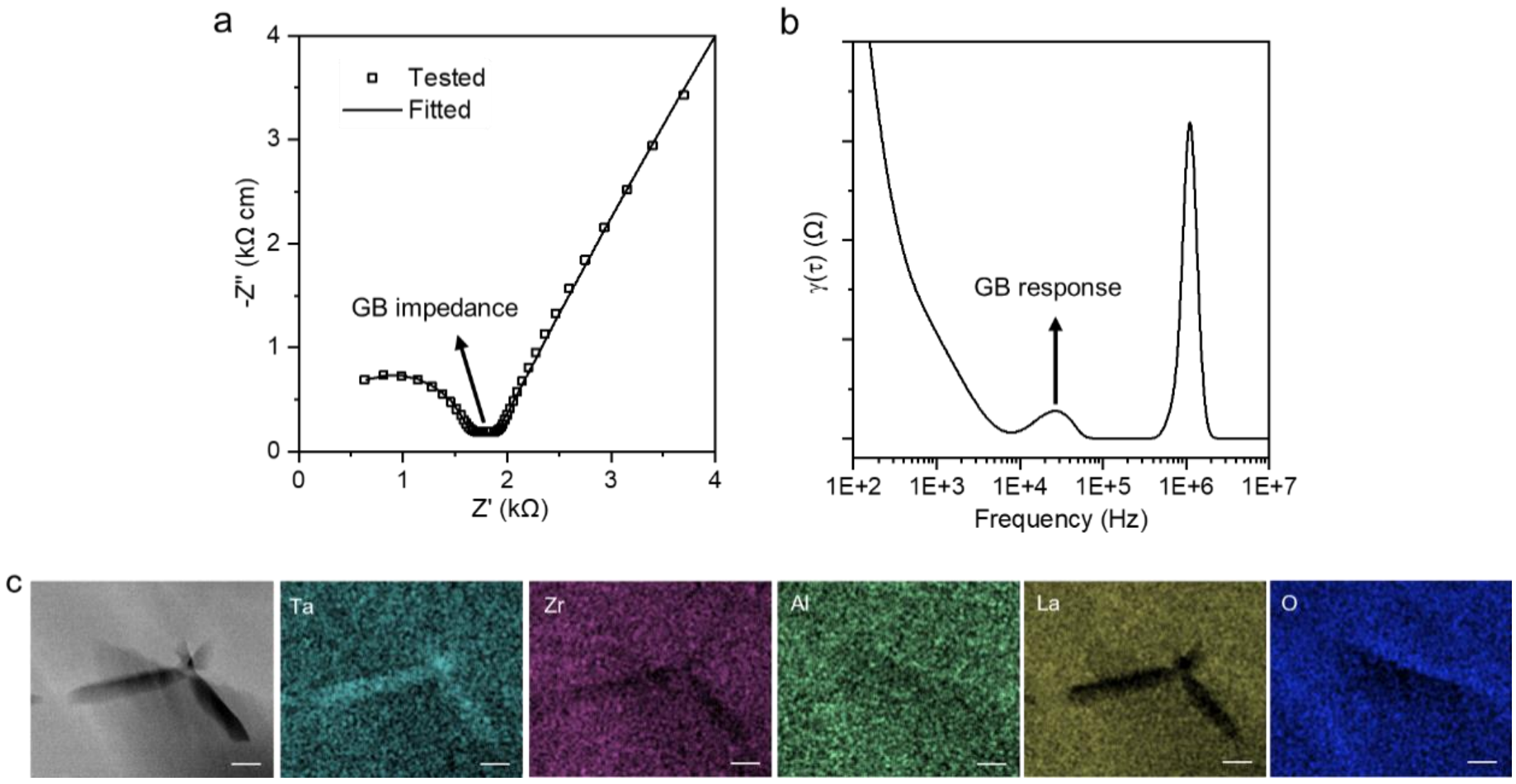

Figure 7. EIS (a) and DRT (b) analysis of LLZO pellet prepared by FAST/SPS via a slow heating (10 K/min) and 1 h dwelling time. (c) Representative HAADF-STEM image and STEM-EDS mappings of the GB during long time FAST/SPS sintering, with 10 K min-1 heating rate and 1 h dwell time. The scale bars are 300 nm.

We would also like to emphasize that our conclusion regarding the kinetic suppression of segregation is not based solely on computational results, but is supported by the combined result of simulations, EIS/DRT analysis, and the recurrence of segregation after post-annealing treatment.

Finally, we would like to point out that a kinetic mechanism for suppressing segregation by rapid sintering has also been demonstrated in other ceramic systems. Zahler et al. reported that fast sintering can suppress Ti segregation in $SrTiO_3$ (J. Eur. Ceram. Soc.

2023, 43, 6925-6933). More recently, Kindelmann et al. showed that rapid FAST/SPS sintering can effectively suppress Y segregation at GBs in $BaZr_{0.7}Ce_{0.2}Y_{0.1}O_{3-\delta}$ (Adv. Energy Mater. 2024, 2404410). In addition, they further designed systematic annealing treatments with different temperatures and durations, which showed that the extent of Y segregation increases with increasing annealing temperature and longer annealing time, which can be considered a strong indication of kinetically controlled segregation.

Change to manuscript (page12)

HAADF-STEM reveals a thin and clean GB (Fig. 2e and Supplementary Fig. 4e-h), and the corresponding STEM-EDS mapping further confirms that no statistically significant segregation is detected within the measurement accuracy (Supplementary Table 4). When the SF-LLZO sample is annealed again in a conventional sintering process, slight Ta segregation can be observed, confirming the kinetic control of element segregation (Supplementary Fig. 4i). In addition, the sample synthesized by FAST/SPS under a slower heating rate (10 K min^{-1}) and a prolonged holding time (1 h) produced a secondary phase, which can be distinguished by the distinctly dark regions in the HAADF image (Supplementary Fig. 4j).
